# Discovery of Stereoselective Targeted Covalent Inhibitors of the RAB27-Effector Protein–Protein Interaction

**DOI:** 10.1021/jacs.6c08927

**Published:** 2026-08-03

**Authors:** Elena De Vita, Adam M. Thomas, Delia Brustur, Montse Tersa, Rita Petracca, Shradda R. Vadodaria, Deborah A. Briggs, Jack W. Houghton, Thomas Lanyon-Hogg, Gregory B. Craven, R. Marc L. Morgan, Alan Armstrong, David J. Mann, Katharine Lodge, Alistair N. Hume, Ernesto Cota, Edward W. Tate

**Affiliations:** † Department of Chemistry, 4615 Imperial College London, London W12 0BZ, U.K.; ‡ Centre for Molecular Cell Biology, Department of Biochemistry, School of Biological and Behavioural Sciences, 4617 Queen Mary University of London, London E1 4NS, U.K.; § Department of Life Sciences, Imperial College London, London W12 0BZ, U.K.; ∥ School of Life Sciences, 6123 University of Nottingham, Nottingham NG7 2UH, U.K.; ⊥ ZoBio B.V., J.H. Oortweg 19, 2333 CH Leiden, Netherlands; # National Heart & Lung Institute, Imperial College London, Hammersmith Campus, London W12 0NN, U.K.

## Abstract

RAB27A and RAB27B are homologous small GTPases that regulate intracellular vesicle trafficking, orchestrating endocytic and exocytic processes that affect cellular communication, immune responses, and the dynamics of the cellular microenvironment. Through their interactions with effector proteins, RAB27A/B plays roles in tumor metastasis and chronic inflammation. However, pharmacological modulation of their activity faces challenges typical of small GTPases, including a lack of well-defined pockets outside the conserved GTP binding site and large RAB27-effector protein–protein interaction (PPI) surfaces. Here, we present the discovery and development of the first cell-active, rationally designed covalent inhibitors of the RAB27-effector interaction, targeting a non-conserved cysteine residue flanking the PPI interface. An electrophile-first biochemical screen led to a novel class of acrylamide covalent inhibitors, and X-ray crystallography structure-guided design led to optimized inhibitors and probes that enantioselectively target RAB27A/B Cys123 in cells. Potency and selectivity were confirmed through biochemical and cellular assays, including chemical proteomics and phenotype recapitulation in melanocytes, alongside a matched inactive enantioprobe control. In contrast, a previously reported compound, Nexinhib-20, was found to be cytotoxic and to exert its activity through non-selective reactivity. This work provides the first toolbox of cell-active chemical probes for RAB27 that can be used in future studies to shed light on the function of this protein and its potential as a therapeutic target.

## Graphical abstract

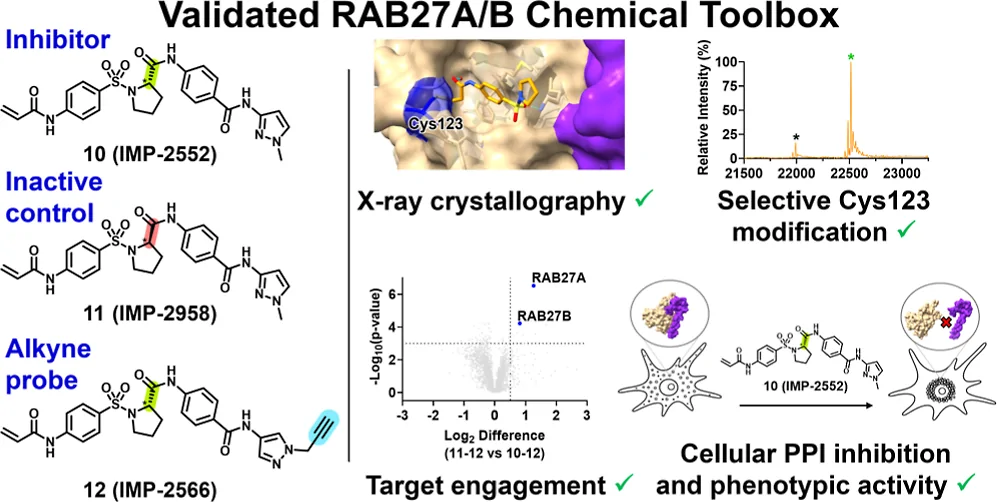

## Introduction

RAB27A and B are small GTPases belonging to the Ras-analogin brain (Rab) family, which encompasses more than 60 members of the Ras superfamily that control trafficking and fate of intracellular vesicles.^1^ Human RAB27A and B (hereafter referred to as RAB27A/B) share 72% homology and regulate vesicle trafficking and docking to membranes, with non-redundant roles in vesicle secretion.^2,3^ For example, in melanocytic cells, RAB27A specifically controls the trafficking and secretion of melanosomes, which are intracellular vesicles that shuttle the pigment melanin into keratinocytic skin and hair cells.^4^

The interaction of Rab proteins with membranes is directed by C-terminal geranylgeranylation by Rab geranyl–geranyl transferase and inhibited by Rab GDP dissociation inhibitor, which complexes to geranylgeranylated GDP-bound Rab proteins in the cytosol.^5^ RAB27A/B display low intrinsic GTPase activity, and cycling between active (GTP-bound) and inactive (GDP-bound) states is tightly regulated by GTPase activating proteins and guanosine nucleotide exchange factors. When bound to GTP, RAB27A/B docks to intracellular membranes such as melanosomes or multivesicular bodies and adopts a more rigid conformation that favors binding to specific effector proteins (Figure 1A). The 11 known RAB27A/B effectors belong to three distinct families: synaptotagmin-like proteins (Slp), containing tandem C2 Ca^2+^-binding motifs; the Slac2 family, lacking these C2 motifs; and Munc13-4.^6^ Recently, SPIRE-type actin nucleators were also identified to work as RAB27A/B effectors.^7^ RAB27A/B-effector protein–protein interactions (PPIs) feature an essential binding interface corresponding to a shallow pocket that hosts a lipophilic effector residue pair, typically tryptophan (W) and phenylalanine (F), also known as the “WF pocket” (Figure 1B).^8–12^ Two cysteine residues flank the WF pocket in RAB27A/B, Cys123, and Cys188, which are non-conserved across the rest of the Rab protein family.^13^ In a previous study, we validated covalent fragments binding to both cysteines by X-ray crystallography (**A01** and **B01**, Figure 1C), demonstrating their ligandability.^13^

**Figure 1:**
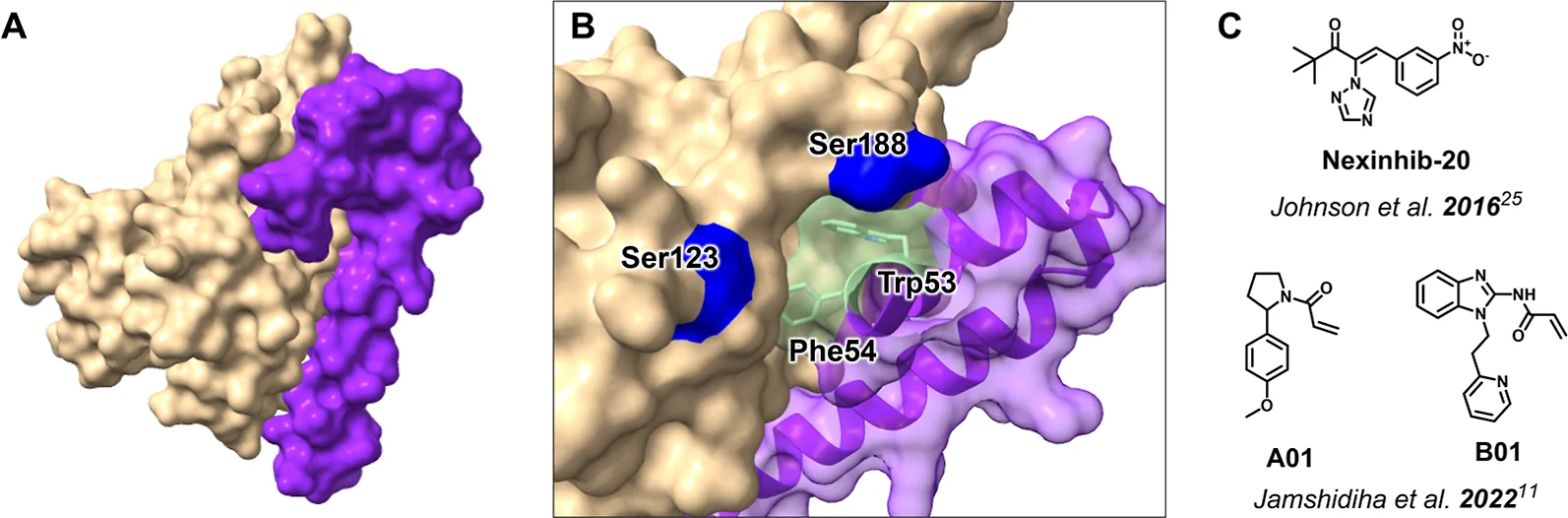
(A) Mouse RAB27A (sand) in complex with the truncated human effector SLP2-A (purple) from PDB:3BC1; (B) WF pocket interacting residues (green) and flanking non-conserved cysteines mutated to serine (blue); and (C) previously reported ligands for RAB27A/B: **Nexinhib-20**,^25^ **A01,** ^11^ and **B01**.^11^

Because of its role in regulating exocytotic processes, RAB27A/B has been implicated in the remodeling of the tumor microenvironment across multiple types of malignancies,^14^ including breast,^15,16^ pancreatic,^17,18^ and colorectal cancers.^19,20^ For example, RAB27A knock-down significantly improves anticancer efficacy of immune-checkpoint inhibitor therapies by reducing exosomal PD-L1, which otherwise impairs T-cell-mediated antitumor surveillance and dampens antibody therapies targeting PD-L1.^21^ These findings suggest that targeting RAB27A/B could disrupt the tumor microenvironment and potentially prevent the establishment of premetastatic niches.^22^ Furthermore, a recent study reported that targeting RAB27A/B could be exploited to facilitate mobilization of immune cells to the brain tissue in brain tumors.^23^ RAB27A has also been found to support the budding process of virus release, for example, for influenza virus, via effectors SYTL1 and SYTL4, suggesting a potential application of RAB27A/B inhibitors in antiviral therapy.^24^

Loss-of-function mutations of RAB27A are viable in humans but result in an autosomal-recessive disorder known as Griscelli type II syndrome, which is characterized by partial albinism and hemophagocytic lymphohistocytosis (HLH).^25^ If identified early, HLH can be treated by bone marrow transplant, while knockout (KO) of RAB27A/B in adult mice is viable with a milder phenotype.^26^ Together, these observations suggest that transient chemical inhibition of the RAB27A/B function in adult patients is likely to carry a low risk of mechanism-based toxicity, motivating the identification of selective inhibitors.

The current lack of high-quality chemical probes hampers efforts to validate RAB27A/B as an actionable target in diseases. Although widely used to study RAB27A/B function, Nexinhib-20, originally reported as a selective, noncovalent RAB27A inhibitor,^27^ contains pan-assay interference (PAINs) motifs, such as a nitro group and a highly conjugated α,β-unsaturated ketone (Figure 1C).

Here, we report the discovery and validation of a novel chemical series of covalent inhibitors that target RAB27A/B Cys123. Through a range of orthogonal biochemical and cellular assays, we demonstrate that optimized inhibitors and probes engage endogenous RAB27A/B in different cell lines and phenocopy RAB27A KO in primary melanocytes through disruption of key effector PPIs. These compounds represent the first example of validated cellular probes for this challenging PPI target alongside matched enantiomeric negative controls.

## Results and Discussion

### Identification of a Synthetically Tractable Acrylamide Fragment with Enhanced Reactivity for RAB27A Cys123

To enable structure-guided discovery and validation of novel RAB27A/B chemical probes, we recently reported a RAB27A recombinant construct termed fRAB27A, which is GppNHp-bound (Gln78Leu locked, stopping intrinsic GTP hydrolysis), lacks the hypervariable prenylated C-terminal region, and is N-terminally fused to a truncated section of the RAB27A/B effector SLP2-A, allowing facile crystallization (Figure 2A,B).^13^ RAB27A and B are highly homologous at the WF pocket; hence, we selected RAB27A as the primary model for biochemical studies. To study modification of a single exposed cysteine, we produced fRAB27A-Cys123 and fRAB27A-Cys188 recombinant constructs, where the number reflects which of the two cysteines is present, while the other cysteine was mutated to a serine to minimize perturbation of the pocket architecture (Figure 2A). Following a pilot screen, we identified two fragments (**A01** and **B01**, Figure 1C) binding at either cysteine, which were validated by X-ray crystallography using our fusion constructs (Figure 2B).^13^ However, these scaffolds were found unsuitable for further development into specific RAB27A/B probes due to challenges associated with analogue synthesis and optimization and because one scaffold had previously emerged from an independent published screening effort.^28^

**Figure 2:**
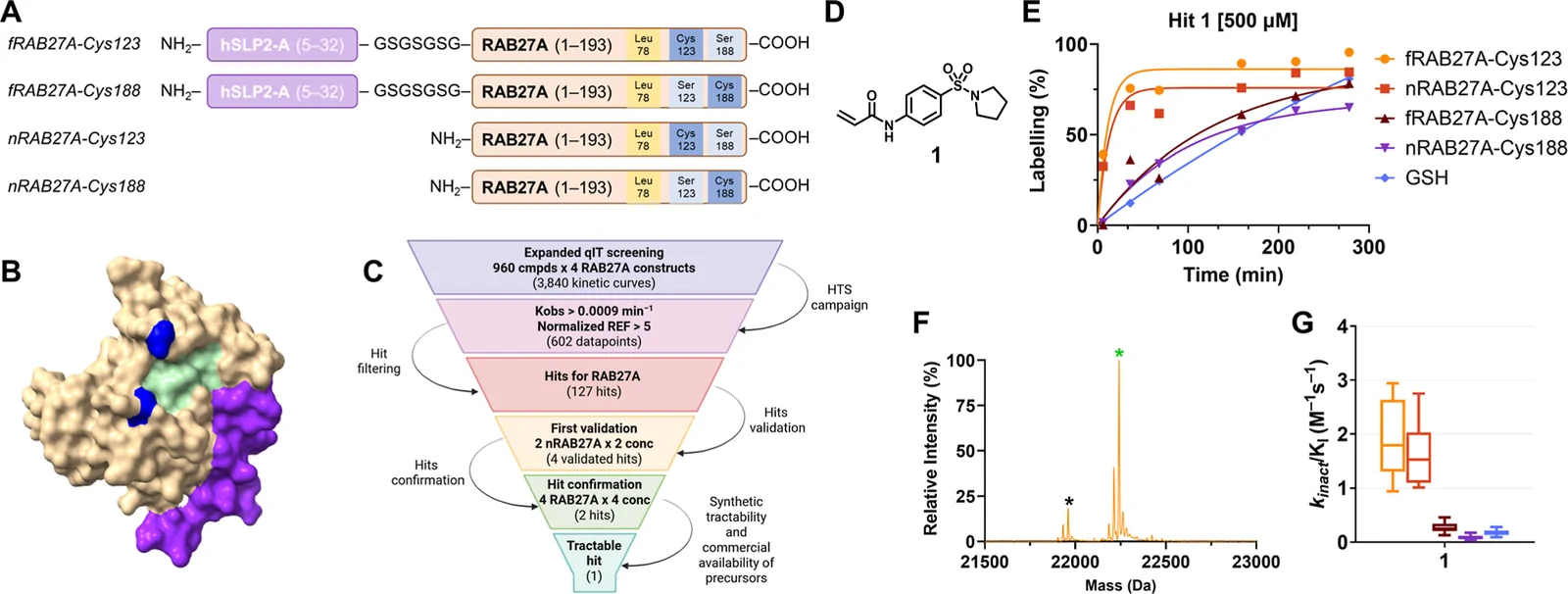
(A) Main RAB27A recombinant constructs used in this work with introduced point mutations, including present Cys;^11^ (B) fusion construct of RAB27A (PDB:7OPP) highlighting the available WF pocket space (green) and the flanking cysteine residues (blue), both mutated to serine in this structure; (C) covalent fragment screening cascade; (D) chemical structure of hit fragment **1**; (E) kinetic covalent modification curves for all thiols by **1** determined via the quantitative irreversible tethering (qIT) assay, normalized as percentage labeling over time; (F) intact protein mass spectrometry (MS) analysis of nRAB27A-Cys123 (black asterisk, MW: 21961 Da) following incubation with **1** (500 μM, 1 h, rt) showing expected covalent adduct formation (green asterisk, MW: 22241 Da); (G) *k*_inact_/*K*_I_ values calculated for **1** from at least 10 independent experiments (for *K*_obs_ graphs, see Figure S2).

We performed an expanded screen using an in-house library of 960 acrylamide fragments with the qIT assay, which measures engagement of a single exposed thiol in the target protein (Figure 2C).^29^ We screened fusion constructs fRAB27A-Cys123 and fRAB27A-Cys188 and non-fusion constructs nRAB27A-Cys123 and nRAB27A-Cys188 lacking the fused sequence of the truncated effector (Figure 2A). Furthermore, we screened the library against glutathione (GSH) as a filter for hyperreactive fragments. Our aim was to identify covalent fragments with enhanced and selective reactivity for either RAB27A Cys123 or Cys188 across both the corresponding fusion and non-fusion constructs, minimizing the risk of false positives while preserving the capacity to crystallize hits in the fusion construct.

Library members were incubated at 500 μM, with each thiol-bearing protein construct or GSH (5 μM) for 24 h at 4 °C, and samples were taken for qIT analysis at regular time points, leading to 3840 kinetic curves across four protein constructs. These data were processed to estimate *k*_obs_ for each construct and filtered by various criteria, including > 5-fold enhanced *k*_obs_ over GSH (Figure 2C, see Supporting Information methods for details). Four compounds were selected for validation in dose–response analyses against all thiols (fRAB27A-Cys123, fRAB27A-Cys188, nRAB27A-Cys123, nRAB27A-Cys188, and GSH). Following assessment of synthetic tractability, we selected hit compound **1** (**IMP-1704**, Figure 2D), which showed enhanced *k*_obs_ for RAB27A-Cys123 (Figure 2E) and single-site modification by intact protein MS (Figure 2F). *k*_inact_/*K*_I_ analysis showed an 18-fold enhanced rate of covalent modification for nRAB27A-Cys123 over nRAB27A-Cys188 and 10-fold over GSH (Figure 2G). Binding to nRAB27B-Cys123 was also confirmed by intact protein MS (Figure S1).

### X-ray Crystallography Reveals Novel IMP-1704/RAB27A Interactions

In order to gain insight into the binding mode of **1** (**IMP-1704**), we solved the X-ray crystallographic structure of ligand-modified fRAB27A-Cys123 to a resolution of 2.1 Å (Figure 3A, PDB: 8P3G, Table S1), revealing that the compound occupies the WF pocket (Figures 3A and S3). The phenyl ring is oriented toward the hydrophobic patch generated by Met93, displacing the Tyr122 outward as previously observed for covalent fragments,^13^ alongside forming a key hydrogen bond between one of the sulfonamide oxygens and Lys11 (Figure 3A). It is possible that this interaction with Lys11 may contribute to the formation of the covalent adduct due to the conjugation between the sulfonamide and acrylamide moieties, e.g., as previously described for covalent KRAS^G12C^ inhibitors.^30^ We next sought to visualize the noncovalent complex formed prior to covalent modification by soaking **1** into crystals of fRAB27A-Ala123 (fRAB27A­[Cys123Ala/Cys188Ser]), revealing a similar noncovalent interaction with Lys11, which orients the acrylamide group toward RAB27A-Cys123 (Figures 3B, S4, PDB: 8P3J, 2.1 Å). Interestingly, an additional noncovalent binding pocket was observed with a second molecule in the vicinity of the WF pocket, but this site is distal to Cys123 and therefore unlikely to be involved in covalent modification (Figure S5). These structures highlighted a viable vector for fragment growth from the pyrrolidine ring, with the potential to extend further into the effector binding site and thereby displace effector-RAB27A PPIs.

**Figure 3:**
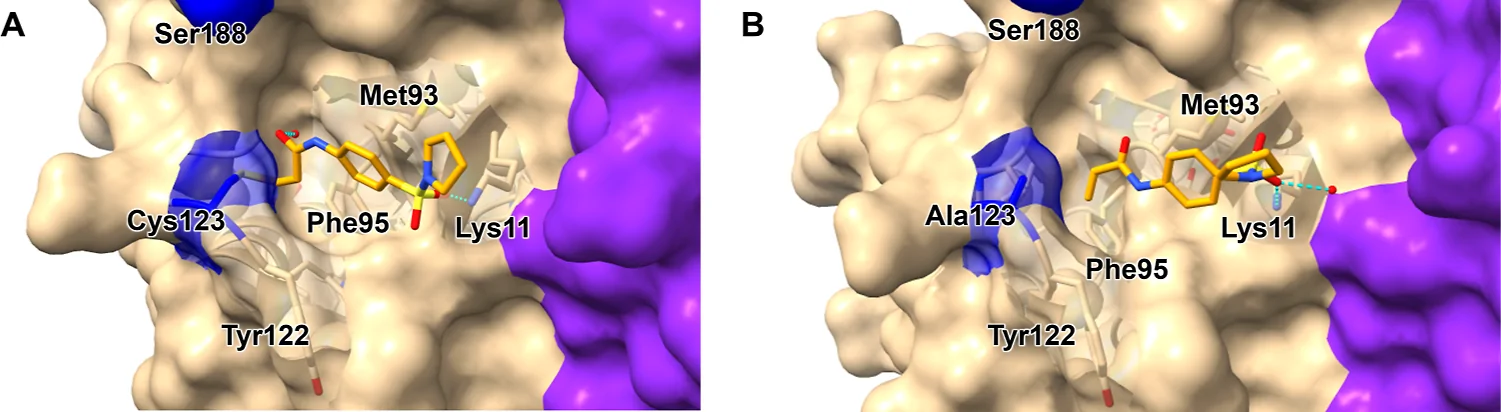
(A) Structure of fRAB27A-Cys123 (PDB: 8P3G) covalently bound to hit **1** (orange), showing key interactions with Lys11; (B) noncovalent complex between fRAB27A-Ala123 and hit **1** (PDB: 8P3J), confirming that the affinity-based orientation favors covalent reaction with Cys123.

### Fragment Growth Identifies Ligands with Enhanced Selective Reactivity for RAB27A Cys123

We next established a fluorescence polarization (FP) assay for rapid analysis of PPI inhibitors at the effector binding site using a novel high-affinity noncovalent cyclic peptide probe for the WF pocket of nRAB27A recently discovered in our laboratories (**IMP-2660**, Figure S6, manuscript in preparation), conjugated to a TAMRA fluorophore. This assay enabled continuous kinetic analysis of **IMP-2660-T** displacement from nRAB27A-Cys123 (*K*_d_ = 221 nM, Figure S6) in 384-well plates and high-throughput determination of *k*_inact_/*K*_I_.

We used this setup to explore the structure–activity relationships (SARs) for the pharmacophore of hit **1** (Figure 4A,B). Consistent with X-ray crystallography, the para-arylsulfonamide moiety was found to be critical for binding, likely contributing to both affinity and reactivity of the series, with analogous amide **2** and meta-sulfonamide **3** being inactive (Figure 4B). Functionalization of the pyrrolidine with a 2-(*S*)-methyl ester (**4**, **IMP-1712**) was well tolerated and led to 2-fold enhancement in modification kinetics, consistent with a conformation leading to enhanced hydrogen-bonding interactions with Lys11 and the backbone of Arg90, as observed by X-ray crystallography for this analogue (Figure 4C). Inversion of the stereochemistry of **4** drastically reduced activity both in FP and intact protein MS assays (**5**, Figure 4B), demonstrating a stereoselective interaction between **4** and RAB27A (Figure 4C).

**Figure 4:**
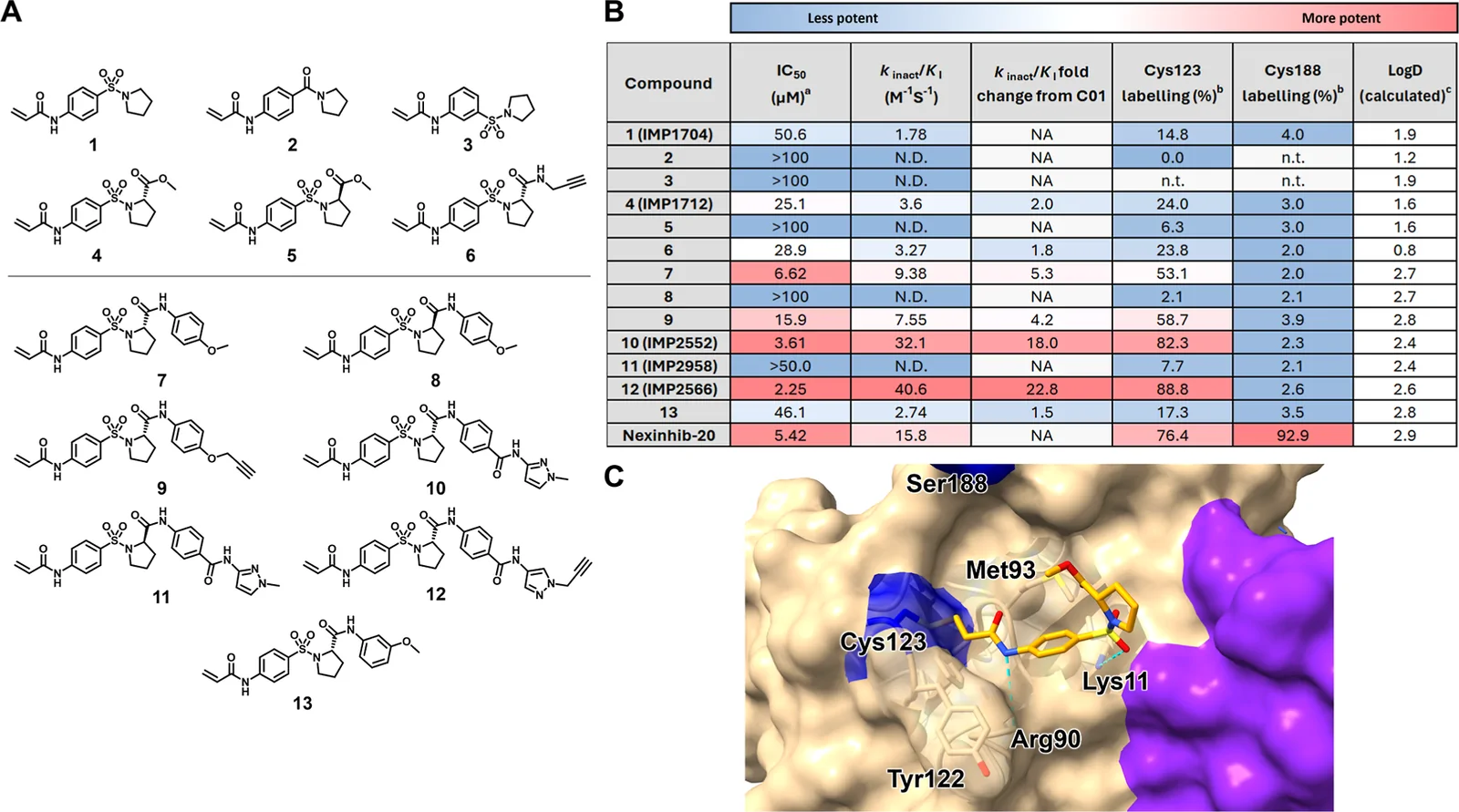
(A) Chemical structure of compounds synthesized for pharmacophore analysis of **1** (top) and fragment growth campaign analogues (bottom); (B) activity values determined by FP and intact protein MS assays. Color scale highlights derivatives from least potent (blue) to most potent (red). a = IC_50_ determined by the FP assay (200 nM nRAB27A-Cys123, 35 nM **IMP-2660-T**) at 2 h; b = percentage labeling determined by intact protein MS following a 1 h incubation of protein (500 nM) with compound (10 μM) at rt; c = LogD calculated by CDD vault during chemical registration using the model by Gedeck et al.;^31^ N.D. = not determined; n.t. = not tested; C) structure of fRAB27A-Cys123 (PDB: 8P3H) covalently bound to **4** (orange), showing key interactions with Lys11 and the backbone of Arg90.

We then proceeded to explore SAR for the corresponding amide analogues of this ester derivative and identified compound **7** with significantly enhanced potency for nRAB27A-Cys123 (>5-fold increased *k*_inact_/*K*_I_ over **1**; Figures 4A,B and S7, S8). Matched enantiomer **8** displayed significantly reduced binding in this assay, consistent with an enantioselective interaction. As an orthogonal method to confirm covalent engagement and Cys123 selectivity, we employed intact protein MS to measure covalent binding against nRAB27A-Cys123 or nRAB27A-Cys188 (Figure 4B). Modification of RAB27A (500 nM) in the presence of excess compound (10 μM) showed clearly favored binding of this series for the Cys123-containing protein, with a single modification event at the expected mass, confirming covalent engagement and Cys123 selectivity (Figures 4B, S9). These results were consistent with the FP data and revealed that selectivity for Cys123 was completely retained over Cys188, demonstrating that the improvement in potency was not driven by intrinsic reactivity of the inhibitors. **Nexinhib-20** (Figure 1C) also exhibited covalent behavior, but in stark contrast displayed no selectivity for Cys123 nor Cys188 (Figures 4B and S7–S9). To further investigate the previously unreported covalent reactivity of **Nexinihib-20**, we performed an LC-MS-based analysis using a physiological concentration of GSH (5 mM, 35 °C, pH 7.4) in phosphate-buffered saline. As expected, **Nexinihib-20** exhibited rapid reactivity, with almost complete consumption after 30 min (Figure S10). In comparison, only 50% of the clinically approved covalent inhibitor **Afatinib** had reacted with GSH after 3 h.

### In-Cell Target Engagement of Endogenous RAB27A/B by Clickable Covalent Probes

To explore whether improved binding in biochemical assays correlated with engagement of endogenous RAB27A/B in cells, we synthesized corresponding alkyne-functionalized analogues of **4** and **7**, **6** and **9**, respectively, and confirmed that these small modifications distal to the acrylamide warhead did not significantly affect covalent modification of recombinant nRAB27A-Cys123 (Figure 4B). We evaluated RAB27A expression in different cancer cell lines and identified triple-negative breast cancer cell line MDA-MB-231 as a suitable model system with the readily detectable RAB27A expression (Figure S11). Incubation of MDA-MB-231 cells with alkyne-tagged probe followed by lysis, ligation to biotin-azide, and analysis by streptavidin mass-shift assay revealed good target engagement by **9**, with substantially higher modification of endogenous RAB27A compared to the less potent probe **6** (Figure 5A) and in a dose-dependent manner as confirmed by immobilized streptavidin pull-down experiments (Figure 5B).

**Figure 5:**
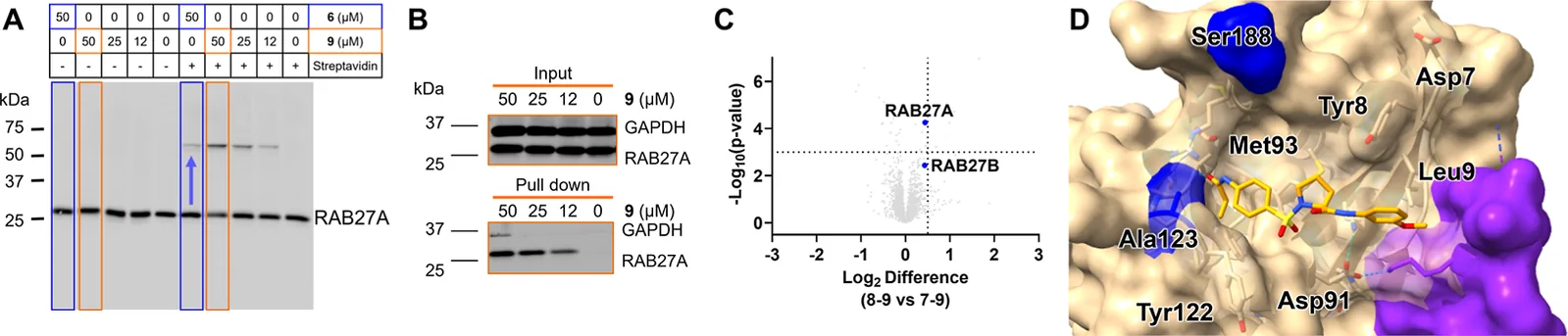
(A) Western blot analysis (α-RAB27A) of the streptavidin-shift assay using alkyne-tagged probes **6** and **9** (2 h incubation); (B) Western blot analysis (α-GAPDH and α-RAB27A) of dose-dependent pull-down of RAB27A following a 2 h treatment with probe **9**, CuAAC chemistry to add a biotin tag and incubation with streptavidin beads; (C) volcano plot for chemical proteomics competition experiments showing the difference (log_2_) between targets competed by **7** and inactive control **8** against probe **9**. Treatment conditions: **7** (20 μM) or inactive control **8** (20 μM) for 2 h followed by **9** (10 μM) for 2 h; (D) noncovalent complex between fRAB27A-Ala123 (sand) and **13** (orange, PDB: 8P3K), showing orientation of the second phenyl ring toward the fused effector region.

Encouraged by the improvement in cellular engagement of RAB27A, we explored the broader in cellulo selectivity of alkyne **9** and corresponding inhibitor **7** by pull-down and competitive chemical proteomics. Incubation with **9** (10 μM) for 2 h showed that RAB27A was significantly enriched in probe-treated samples and was found to be within the top 8.1% of engaged proteins (Figure S15). Consistent with our hypothesis, RAB27B was also found to be significantly enriched in samples treated with probe **9**, albeit with lower engagement, likely due to its lower expression in this cellular system (Figure S12). Competition experiments with a 2 h preincubation with **7** (20 μM) or negative control **8** (20 μM) showed that only the active enantiomer inhibitor **7** competed for engagement of RAB27A/B, while inactive control **8** maintained a similar off-target reactivity profile but did not block RAB27A/B binding by **9** (Figures 5C, S16). These results represent the first example of covalent in-cell target engagement for any Rab protein, providing a benchmark for further optimization of activity and selectivity.

### Structure-Guided Optimization Delivers Potent Stereoselective RAB27A/B Probe IMP-2552 and Inactive Enantiomer Control IMP-2958

With the first cellular RAB27A probes in hand, we set out to improve potency and selectivity by further extending into the effector binding pocket to effectively disrupt RAB27/effector PPIs based on elucidation of the noncovalent binding mode by X-ray crystallography of **13** (Figure 5D). Para-substituted analogue **7**, despite being more potent, did not result in defined crystal structures, likely due to clashes with the fused effector. Elaboration through an amide linker yielded compound **10** (**IMP2552**, Figure 4A), which exhibited enhanced binding kinetics for nRAB27A-Cys123 with a *k*_inact_/*K*_I_ of 32.1 M^–1^ s^–1^ in the competition FP assay (Figures 4B and S7, S8). Selectivity for Cys123 over Cys188 was further corroborated for **10** by intact protein MS analyses, showing a single labeling event for nRAB27A containing both Cys123 and Cys188 (Figures 6A, S9), and for nRAB27B-Cys123 (Figure S1), consistent with the high homology between RAB27A and B in the WF pocket. **IMP-2552** was found to engage both GTP- (active) and GDP-bound (inactive) forms of nRAB27A-Cys123 with equal potency by intact protein MS, suggesting that binding for these compounds at the WF pocket is nucleotide-independent (Figure S13). Using the GSH reactivity assay described above, compound **10** exhibited reactivity comparable to **Afatinib**, with 40% of compound **10** remaining after 3 h (Figure S10). Consistent with the expectation that these optimized analogues would interfere with effector binding, binding to effector fusion construct fRAB27A-Cys123 was reduced relative to nRAB27A-Cys123, and we were unable to determine interactions introduced by the methylated pyrazole ring through X-ray crystallography. Based on the bound trajectory observed for compound **13** (Figure 5D), we hypothesize that additional interactions may be made toward RAB27A, potentially weakening or disrupting existing effector interactions such as between RAB27A Asp91 with SLP2-A Lys14, which support crystal packing in our X-ray models. Importantly, stereoselectivity was maintained in these analogues, with matched enantiomer **11** (**IMP-2958**) showing substantially reduced RAB27A binding in both the FP and intact protein MS assays (Figures 4B and S7, S9), providing an ideal negative control probe for off-target binding in phenotypic assays.

**Figure 6:**
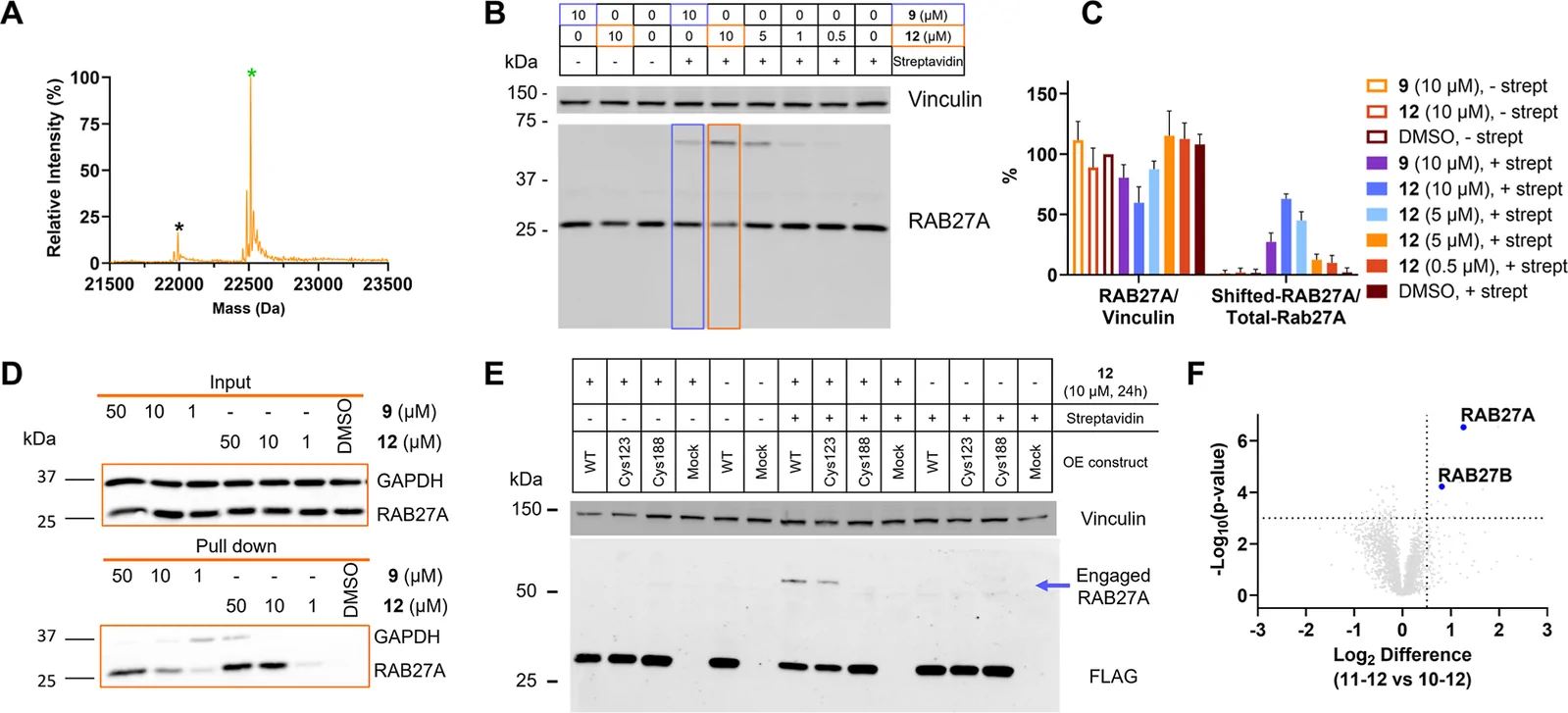
(A) Intact protein MS analysis of nRAB27A-Cys123,Cys188 (1–193, black asterisk, MW: 21991 Da) following incubation with **10** (10 μM, 1 h, rt), showing expected single covalent adduct formation (green asterisk, MW: 22514 Da); (B) streptavidin-shift assay comparing alkyne probes **9** and **12** (24 h incubation); (C) quantification of streptavidin-shift assay shown in B (*n* = 3 independent replicates); (D) Western blot analysis (α-GAPDH and α-RAB27A) of dose-dependent pull-down of RAB27A following a 2 h treatment with probe **12**, CuAAC chemistry to add a biotin tag and incubation with streptavidin beads; (E) Western blot analysis of streptavidin-shift assay using α-FLAG antibody. Importantly, only mutants containing Cys123 are engaged by **12**; hence, the shifted band is only present in WT and Cys123 lanes. WT = FLAG-RAB27A (1–221, P51159); Cys123 = FLAG-RAB27A (1–221, Cys188Ala); Cys188 = FLAG-RAB27A (1–221, Cys123Ala); Mock = no DNA control; (F) volcano plot for chemical proteomics competition experiments showing the difference (log_2_) between targets competed by **10** and inactive control **11** against probe **12**. Treatment conditions: **10** (10 μM) or inactive control **11** (10 μM) for 2 h followed by **12** (5 μM) for 2 h.

In order to assess in cellulo engagement of **10**, a corresponding alkyne-bearing analogue, **12** (**IMP-2566**), was synthesized and validated in the FP and intact protein MS assays (Figures 4B and S7–S9). It should be noted that the pyrazole regioisomers between **10** and **12** differ slightly, owing to the synthetic challenges of synthesizing the propargylated 1,3-isomer. Probe **12** significantly engaged endogenous RAB27A at low micromolar concentrations in MDA-MB-231 cells, reaching ∼50% engagement after 24 h of incubation at 10 μM, as quantified by streptavidin-shift assays (Figure 6B,C) and pull-down experiments (Figure 6D). Evidence from streptavidin-shift analysis of transiently overexpressed FLAG-tagged RAB27A-WT, RAB27A-Cys123­[Cys188Ser], and RAB27A-Cys188­[Cys123Ser] mutants further supported specific Cys123 modification in cells (Figure 6E). We observed that the α-FLAG antibody was less sensitive toward the streptavidin–RAB27A complex, but this could be better visualized with α-RAB27A antibody, which also showed engagement of endogenous RAB27A across all treatment conditions with **12** (Figure S14). Chemical proteomics revealed improved selectivity for RAB27A compared to probe **9**, observing engagement in the top 1.7% of enriched targets across the proteome (Figure S15). Competition experiments once again confirmed stereoselective inhibition of RAB27A/B engagement by **10** but not by **11** (Figures 6F, S16). No other significantly competed protein belonged to the Rab or Rab effector families, minimizing the risk of off-target impacts on vesicle trafficking. No cytotoxicity was observed at concentrations that delivered significant RAB27A/B target engagement in chemoproteomic analyses for these compounds (Figure S17). Overall, the cellular profile of positive and negative control compounds with good off-target overlap (see supporting tables for proteomics) supports the use of these probes in cell-based systems at acceptable concentrations for the study of RAB27A/B-related phenotypes.

### Targeting the WF Pocket with IMP-2552 Disrupts RAB27-Effector Interactions and Phenocopies RAB27A Knockout in Melan-a Melanocytes

In order to test our hypothesis that a targeted covalent WF pocket ligand would inhibit RAB27A/B activity by displacing effectors, we developed a coimmunoprecipitation (Co-IP) assay for transiently expressed GFP-tagged full-length hSLP2-A to evaluate interaction with native RAB27A in cells, employing GFP-trap beads for enrichment. While unperturbed PPIs between hSLP2-A and RAB27A resulted in the expected Co-IP of RAB27A, treatment of cells with either lead inhibitor **10** or alkyne probe **12** significantly blocked RAB27A–SLP2-A interaction at 10 and 20 μM, while the inactive enantiomer control **11** showed no effect up to 20 μM (Figure 7A,B). Taken together with in-cell target engagement data, these results further support the use of these compounds as the first in-cell targeted covalent RAB27A/B PPI inhibitors.

**Figure 7:**
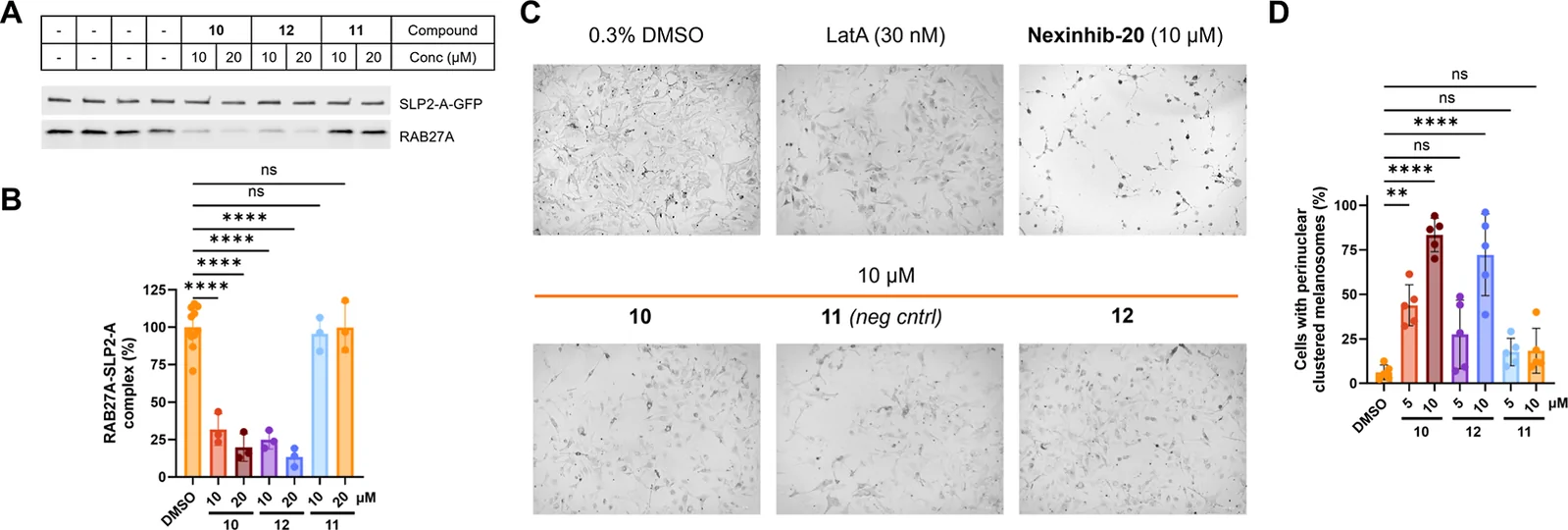
(A) Western blot analysis of GFP-trap Co-IP assays of MDA-MB-231 cells transiently expressing GFP-hSLP2-A. Treatment conditions: **10**, **11**, and **12** (10 and 20 μM, 14 h); (B) quantification of Co-IP experiments shown in A (*n* = 3 independent replicates). ****p < 0.0001, ns = not significant; (C) representative brightfield images of melan-a mouse melanocytes treated with Latrunculin A (30 nM), **Nexinhib-20** (10 μM), **10** (10 μM), **11** (10 μM), or **12** (10 μM) for 24 h; white scale bar is 100 μm; (D) quantification of melanocytes displaying perinuclear melanosomes from assays shown in A (*n* = 5 independent replicates; see montage in Figure S18).

The role of RAB27A in the regulation of skin and hair pigmentation is well established, and loss of function is responsible for the hypopigmentation observed in Griscelli-type II patients. RAB27A knockout (KO) in melanocytes or mutations leading to loss of binding to effector protein SLAC2-A (melanophilin) results in perinuclear accumulation of melanosomes, the specialized transport vesicles that shuttle melanin outside the cell to the epidermis, and cause hypopigmentation in vivo.^32^ This phenotype is fully dependent on the RAB27A activity and can be readily assayed by brightfield microscopy in melanocytes. We treated melan-a mouse melanocytes at increasing probe concentrations for 24 h and quantified cells exhibiting perinuclear accumulation of melanosomes (Figures 7C,D and S18). In agreement with our previous cellular results, both **10** and **12** dose-dependently inhibited melanosome distribution, leading to perinuclear clustering, while enantiomeric inactive compound **11** showed similar distribution of melanosomes to untreated melanocytes (Figure 7C). Consistent with selective RAB27A inhibition, no significant cytotoxicity was observed for compounds **10**, **11**, or **12** at the tested concentrations over the course of the experiment (Figure S19).

**Nexinhib-20** has been widely used in cellular and in vivo studies of RAB27A,^27^ despite a lack of direct evidence for on-target activity. In the melan-a assay, **Nexinhib-20** showed profound cytotoxicity and cell death at 10 μM (Figures 7C, S19), a standard concentration used for this compound in cellular assays.^33^ These data, together with evidence for promiscuous reactivity (Figures 4B and S7–S10) and the presence of PAIN motifs in the structure, suggest that Nexinhib-20-related compounds should not be applied in studies of RAB27A/B. Prior studies, which found Nexinhib compounds unable to recapitulate RAB27A KO, may merit revisiting with bona fide RAB27A/B probes.^34^

## Conclusions

Our work demonstrates the first successful application of a targeted covalent approach to tackle a challenging small Rab GTPase, expanding the scope of covalent inhibition in the Ras superfamily.^35^ Optimized probes engage native RAB27A/B at a non-conserved cysteine residue (Cys123) and consequently inhibit PPIs that drive RAB27A/B cellular activity, producing the first validated chemical probes to study this target, alongside matched enantiomeric negative controls.

Starting from a suitable covalent fragment and a carefully selected vector for elaboration, we were able to identify and develop the first small molecule inhibitors of the RAB27A/effector PPI through structure-guided fragment growth toward the effector interface, supported by a suite of biochemical and cellular assays and X-ray crystallography. Early introduction of a stereocenter of defined configuration essential for specific binding to RAB27A/B allowed the development of matched enantiomer inactive probes as negative controls, enabling comparative analysis in complex cellular models by normalizing for potential off-target effects, as shown in melanocytes. Chemical proteomics studies confirmed that our lead probes, inhibitor **10** (**IMP-2552**) and alkyne probe **12** (**IMP-2566**), but not matched negative control **11** (**IMP-2598**), engage RAB27A and RAB27B in cells and enantioselectively disrupt RAB27 PPIs.

Our results also invalidate **Nexinhib-20** as a putative selective probe for RAB27A/B, inviting great caution in its use in any biological system, in agreement with several previously published studies.^34^

In summary, our work brings together a novel toolbox of chemical probes to advance the study of RAB27A/B biology and its future exploration as an actionable target in diseases from cancer to chronic inflammation. We envision that targeting the WF pocket with these compounds may support systematic mapping of the RAB27A/B interactome at this site and help uncover the context-dependent biology of this GTPase. While these chemical tools exhibit robust target engagement, Rab family selectivity, and phenotypic activity, further optimization to enhance proteome-wide selectivity is ongoing to enable their evaluation in more advanced biological models and ultimately therapeutic contexts. We will be pleased to share these probes with interested laboratories to advance the field.

We anticipate that some of the concepts applied here, for example, the generation of a potent noncovalent peptide probe for high-throughput analysis of covalent modification leading to inhibition of a PPI, may prove useful for other targets. More broadly, we demonstrate a structure-guided “covalent-first” approach to produce cell-active orthosteric small molecule inhibitors for a specific PPI in the context of a very challenging and to date largely intractable target class.

## Supplementary Material



## Notes

X-ray crystallography structures solved for this work have been deposited on the PDB database (PDBs: 8P3G, 8P3H, 8P3J, 8P3K, 8P3l); proteomics data have been deposited on PRIDE (whole proteome data: PXD077793; chemical proteomics data: PXD077741); and supplementary figures and experimental section including organic synthesis procedures, biochemical assays, and biological assays (PDF)

## References

[1] Hutagalung A. H., Novick P. J. (2011). Role of Rab GTPases in Membrane Traffic and Cell Physiology. Physiol. Rev..

[2] Johnson J. L., Brzezinska A. A., Tolmachova T., Munafo D. B., Ellis B. A., Seabra M. C., Hong H., Catz S. D. (2010). Rab27a and Rab27b
Regulate Neutrophil Azurophilic Granule Exocytosis and NADPH Oxidase
Activity by Independent Mechanisms. Traffic.

[3] Ostrowski M., Carmo N. B., Krumeich S., Fanget I., Raposo G., Savina A., Moita C. F., Schauer K., Hume A. N., Freitas R. P., Goud B., Benaroch P., Hacohen N., Fukuda M., Desnos C., Seabra M. C., Darchen F., Amigorena S., Moita L. F., Thery C. (2010). Rab27a and Rab27b Control
Different Steps of the Exosome Secretion Pathway. Nat. Cell Biol..

[4] Hume A. N., Collinson L. M., Rapak A., Gomes A. Q., Hopkins C. R., Seabra M. C. (2001). Rab27a
Regulates the Peripheral Distribution of Melanosomes
in Melanocytes. J. Cell Biol..

[5] Stenmark H. (2009). Rab GTPases
as Coordinators of Vesicle Traffic. Nat. Rev.
Mol. Cell Biol..

[6] Fukuda M. (2005). Versatile
Role of Rab27 in Membrane Trafficking: Focus on the Rab27 Effector
Families. J. Biochem..

[7] Alzahofi N., Welz T., Robinson C. L., Page E. L., Briggs D. A., Stainthorp A. K., Reekes J., Elbe D. A., Straub F., Kallemeijn W. W., Tate E. W., Goff P. S., Sviderskaya E. V., Cantero M., Montoliu L., Nedelec F., Miles A. K., Bailly M., Kerkhoff E., Hume A. N. (2020). Rab27a Co-Ordinates
Actin-Dependent Transport by Controlling Organelle-Associated Motors
and Track Assembly Proteins. Nat. Commun..

[8] Chavas L. M. G., Ihara K., Kawasaki M., Torii S., Uejima T., Kato R., Izumi T., Wakatsuki S. (2008). Elucidation
of Rab27 Recruitment by Its Effectors: Structure of Rab27a Bound to
Exophilin4/Slp2-a. Struct..

[9] Gillingham A. K., Sinka R., Torres I. L., Lilley K. S., Munro S. (2014). Toward a Comprehensive
Map of the Effectors of Rab GTPases. Dev. Cell.

[10] Fukuda M. (2006). Distinct Rab27A
Binding Affinities of Slp2-a and Slac2-a/Melanophilin: Hierarchy of
Rab27A Effectors. Biochem. Biophys. Res. Commun..

[11] Kanno E., Ishibashi K., Kobayashi H., Matsui T., Ohbayashi N., Fukuda M. (2010). Comprehensive Screening for Novel Rab-Binding Proteins
by GST Pull-Down Assay Using 60 Different Mammalian Rabs. Traffic.

[12] Zhao W., Jamshidiha M., Lanyon-Hogg T., Recchi C., Cota E., Tate E. W. (2016). Direct
Targeting of the Ras GTPase Superfamily through
Structure-Based Design. Curr. Top. Med. Chem..

[13] Jamshidiha M., Lanyon-Hogg T., Sutherell C. L., Craven G. B., Tersa M., De Vita E., Brustur D., Pérez-Dorado I., Hassan S., Petracca R., Morgan R. M., Sanz-Hernández M., Norman J. C., Armstrong A., Mann D. J., Cota E., Tate E. W. (2022). Identification
of the First Structurally Validated
Covalent Ligands of the Small GTPase RAB27A. RSC Med. Chem..

[14] Li Z., Fang R., Fang J., He S., Liu T. (2018). Functional
Implications of Rab27 GTPases in Cancer. Cell
Commun. Signaling.

[15] Wang J.-S., Wang F.-B., Zhang Q.-G., Shen Z.-Z., Shao Z.-M. (2008). Enhanced
Expression of Rab27A Gene by Breast Cancer Cells Promoting Invasiveness
and the Metastasis Potential by Secretion of Insulin-Like Growth Factor-II. Mol. Cancer Res..

[16] Hendrix A., Maynard D., Pauwels P., Braems G., Denys H., Van Den Broecke R., Lambert J., Van Belle S., Cocquyt V., Gespach C., Bracke M., Seabra M. C., Gahl W. A., De Wever O., Westbroek W. (2010). Effect of
the Secretory Small GTPase Rab27B on Breast Cancer Growth, Invasion,
and Metastasis. JNCI J. Natl. Cancer Inst..

[17] Kren N., Michaud D., Bagchi S., Greene K., Pylayeva-Gupta Y. (2020). Rab27a Plays
a Dual Role in Metastatic Propensity of Pancreatic Cancer. Sci. Rep..

[18] Pecqueux M., Wende B., Sommer U., Baenke F., Oehme F., Hempel S., Aust D., Distler M., Weitz J., Kahlert C. (2023). RAB27B Expression in Pancreatic Cancer Is Predictive
of Poor Survival but Good Response to Chemotherapy. Cancer Biomarkers.

[19] Li Q., Zhao H., Dong W., Guan N., Hu Y., Zeng Z., Zhang H., Zhang F., Li Q., Yang J., Xiao W. (2022). RAB27A Promotes the Proliferation
and Invasion of Colorectal Cancer Cells. Sci.
Rep..

[20] Feng F., Jiang Y., Lu H., Lu X., Wang S., Wang L., Wei M., Lu W., Du Z., Ye Z., Yang G., Yuan F., Ma Y., Lei X., Lu Z. (2016). Rab27A Mediated by NF-ΚB Promotes the Stemness
of Colon Cancer
Cells via up-Regulation of Cytokine Secretion. OncoTargets.

[21] Ye Z., Xiong Y., Peng W., Wei W., Huang L., Yue J., Zhang C., Lin G., Huang F., Zhang L., Zheng S., Yue J. (2023). Manipulation of PD-L1 Endosomal Trafficking
Promotes Anticancer Immunity. Adv. Sci..

[22] Bobrie A., Krumeich S., Reyal F., Recchi C., Moita L. F., Seabra M. C., Ostrowski M., Théry C. (2012). Rab27a Supports
Exosome-Dependent and -Independent Mechanisms That Modify the Tumor
Microenvironment and Can Promote Tumor Progression. Cancer Res..

[23] Adnani L., Meehan B., Kim M., Choi D., Rudd C. E., Riazalhosseini Y., Rak J. (2025). Immune Cell Infiltration
into Brain
Tumor Microenvironment Is Mediated by Rab27-Regulated Vascular Wall
Integrity. Sci. Adv..

[24] Chen T., Ouyang A., Zou J., Feng Y., Wu C., Jiang M., Tu S., Ding L., Cheng Y., Hu W., Sheng W., Li Y., Jin M., Chen H., Zhou H. (2025). Rab27a Regulates the Transport of Influenza Virus Membrane Proteins
to the Plasma Membrane. Nat. Commun..

[25] Meeths M., Bryceson Y. T., Rudd E., Zheng C., Wood S. M., Ramme K., Beutel K., Hasle H., Heilmann C., Hultenby K., Ljunggren H. G., Fadeel B., Nordenskjöld M., Henter J. I. (2010). Clinical Presentation
of Griscelli Syndrome Type 2
and Spectrum of RAB27A Mutations. Pediatr. Blood
Cancer.

[26] Alexander M., Ramstead A. G., Bauer K. M., Lee S.-H., Runtsch M. C., Wallace J., Huffaker T. B., Larsen D. K., Tolmachova T., Seabra M. C., Round J. L., Ward D. M., O’Connell R. M. (2017). Rab27-Dependent
Exosome Production Inhibits Chronic Inflammation and Enables Acute
Responses to Inflammatory Stimuli. J. Immunol..

[27] Johnson J. L., Ramadass M., He J., Brown S. J., Zhang J., Abgaryan L., Biris N., Gavathiotis E., Rosen H., Catz S. D. (2016). Identification of
Neutrophil Exocytosis
Inhibitors (Nexinhibs), Small Molecule Inhibitors of Neutrophil Exocytosis
and Inflammation: Druggability of the Small GTPase Rab27a. J. Biol. Chem..

[28] Resnick E., Bradley A., Gan J., Douangamath A., Krojer T., Sethi R., Geurink P. P., Aimon A., Amitai G., Bellini D., Bennett J., Fairhead M., Fedorov O., Gabizon R., Gan J., Guo J., Plotnikov A., Reznik N., Ruda G. F., Díaz-Sáez L., Straub V. M., Szommer T., Velupillai S., Zaidman D., Zhang Y., Coker A. R., Dowson C. G., Barr H. M., Wang C., Huber K. V. M., Brennan P. E., Ovaa H., Von Delft F., London N. (2019). Rapid Covalent-Probe
Discovery by Electrophile-Fragment Screening. J. Am. Chem. Soc..

[29] Craven G. B., Affron D. P., Allen C. E., Matthies S., Greener J. G., Morgan R. M. L., Tate E. W., Armstrong A., Mann D. J. (2018). High-Throughput Kinetic Analysis for Target-Directed
Covalent Ligand Discovery. Angew. Chem., Int.
Ed..

[30] Hansen R., Peters U., Babbar A., Chen Y., Feng J., Janes M. R., Li L. S., Ren P., Liu Y., Zarrinkar P. P. (2018). The Reactivity-Driven Biochemical
Mechanism of Covalent
KRAS^G12C^ Inhibitors. Nat. Struct.
Mol. Biol..

[31] Gedeck P., Skolnik S., Rodde S. (2017). ¶ Developing
Collaborative QSAR
Models Without Sharing Structures. J. Chem.
Inf. Model..

[32] Cetica V., Hackmann Y., Grieve S., Sieni E., Ciambotti B., Coniglio M. L., Pende D., Gilmour K., Romagnoli P., Griffiths G. M., Aricò M. (2015). Patients with Griscelli Syndrome
and Normal Pigmentation Identify RAB27A Mutations That Selectively
Disrupt MUNC13–4 Binding. J. Allergy
Clin. Immunol..

[33] Liu W., Cronin C. G., Cao Z., Wang C., Ruan J., Pulikkot S., Hall A., Sun H., Groisman A., Chen Y., Vella A. T., Hu L., Liang B. T., Fan Z. (2022). Nexinhib20 Inhibits Neutrophil Adhesion and Β2 Integrin Activation
by Antagonizing Rac-1-GTP Interaction. J. Immunol..

[34] Poggio M., Hu T., Pai C. C., Chu B., Belair C. D., Chang A., Montabana E., Lang U. E., Fu Q., Fong L., Blelloch R. (2019). Suppression
of Exosomal PD-L1 Induces Systemic Anti-Tumor
Immunity and Memory. Cell.

[35] Amgen Announces First Clinical Data Evaluating Novel Investigational KRASG12C Inhibitor AMG 510 At ASCO 2019. https://www.amgen.com/media/news-releases/2019/06/amgen-announces-first-clinical-data-evaluating-novel-investigational-krasg12c-inhibitor-amg-510-at-asco-2019/(accessed 06 19, 2020).

