# Supplementary material for: Discovery of Stereoselective Targeted Covalent Inhibitors of the RAB27-Effector Protein–Protein Interaction
Source: J Am Chem Soc. 2026 Aug 3;148(38):40880–8. doi: 10.1021/jacs.6c08927 (PMC13632578; doi:10.1021/jacs.6c08927)
Supplement: Supplementary file 1 [file ja6c08927_si_001.pdf]

Supplementary Information

for

Discovery of stereoselective targeted covalent inhibitors of the RAB27-effector protein-protein interaction

Elena De Vita,[1,2,$*] Adam M. Thomas,[1,$] Delia Brustur,[1] Montse Tersa,[3] Rita Petracca,[1] Shradda R. Vadodaria,[1] Deborah A Briggs,[4] Jack W. Houghton,[1] Thomas Lanyon-Hogg,[1] Gregory B. Craven,[1,3] R. Marc L. Morgan,[1,5] Alan Armstrong,[1] David J. Mann,[3] Katharine Lodge,[6] Alistair N. Hume,[4] Ernesto Cota,[3] and Edward W. Tate[1,*]

[$] These authors contributed equally.

[1] Imperial College London, Department of Chemistry, Imperial College London, London, W12 0BZ, UK

[2] Centre for Molecular Cell Biology, Department of Biochemistry, School of Biological and Behavioural Sciences, Queen Mary University of London, London, E1 4NS, UK

[3] Imperial College London, Department of Life Sciences, Imperial College London, London, W12 0BZ, UK

[4] School of Life Sciences, University of Nottingham, Nottingham NG7 2UH, UK

[5] ZoBio B.V., J.H. Oortweg 19, 2333 CH Leiden, Netherlands

[6] National Heart & Lung Institute, Imperial College London, Hammersmith Campus, W12 0NN, UK

*Corresponding authors

e.devita@qmul.ac.uk

e.tate@imperial.ac.uk

Table of contents

- **Supplementary Figures S3**
- **Supplementary Tables S16**
- **Abbreviations S21**
- **Biological Procedures S23**
- **General chemistry experimental S33**
- **NMR figures S50**
- **HPLC Traces S63**
- **Chiral HPLC Traces..S76**
- **References S78**

Supplementary Figures

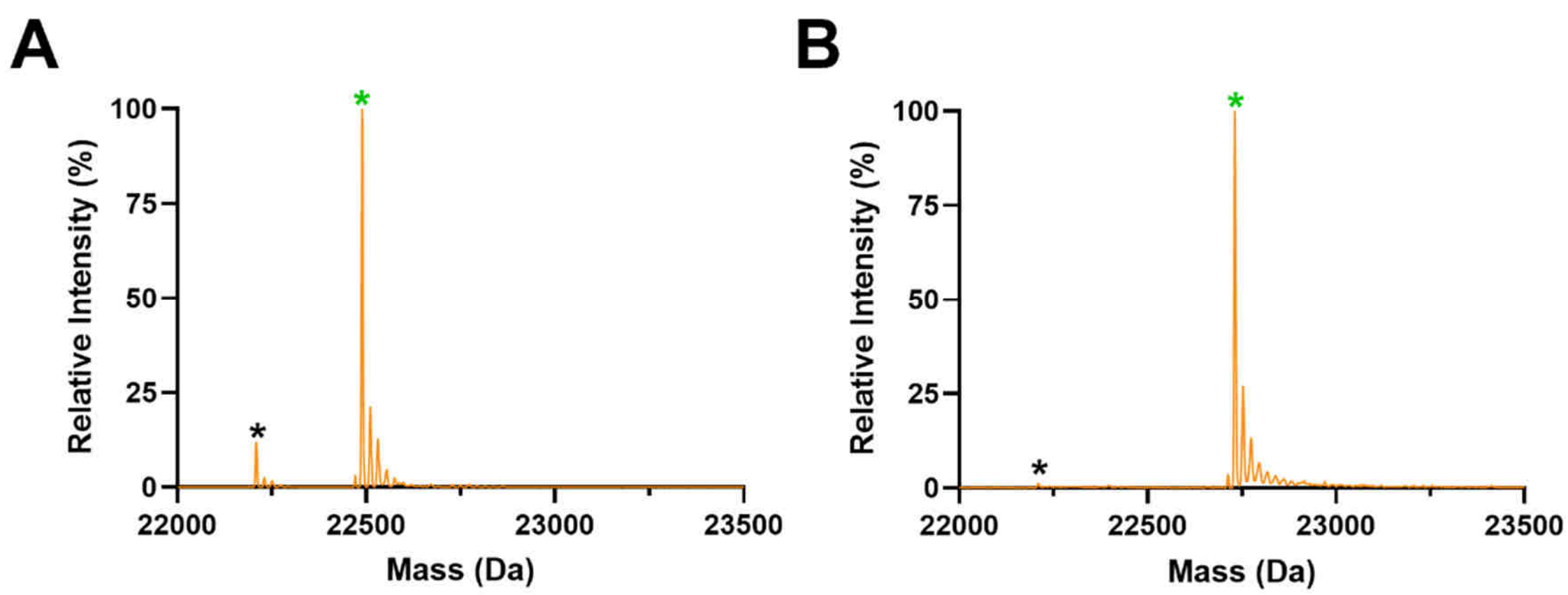

Figure S1. Intact protein MS analysis of 500 µM compound **1** (A) and 10 µM compound **10** (B) incubated for 1 h at rt with 500 nM nRAB27B-Cys123 (black asterisk, MW: 22209 Da) showing expected single covalent adduct formation (green asterisk, A → MW: 22489 Da; B → MW: 22731 Da).

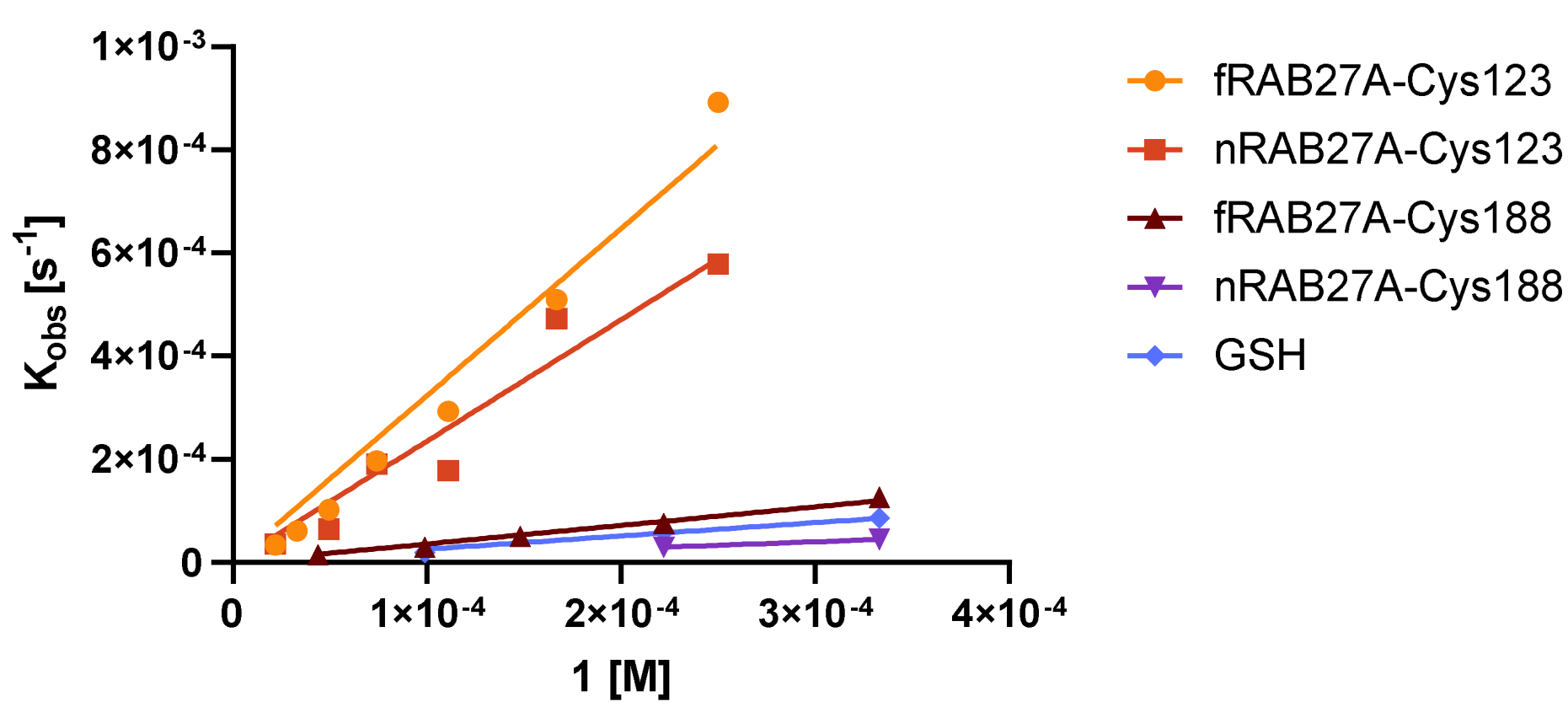

Figure S2. Calculated K_{obs} from dose-response qIT assay for compound **1**.

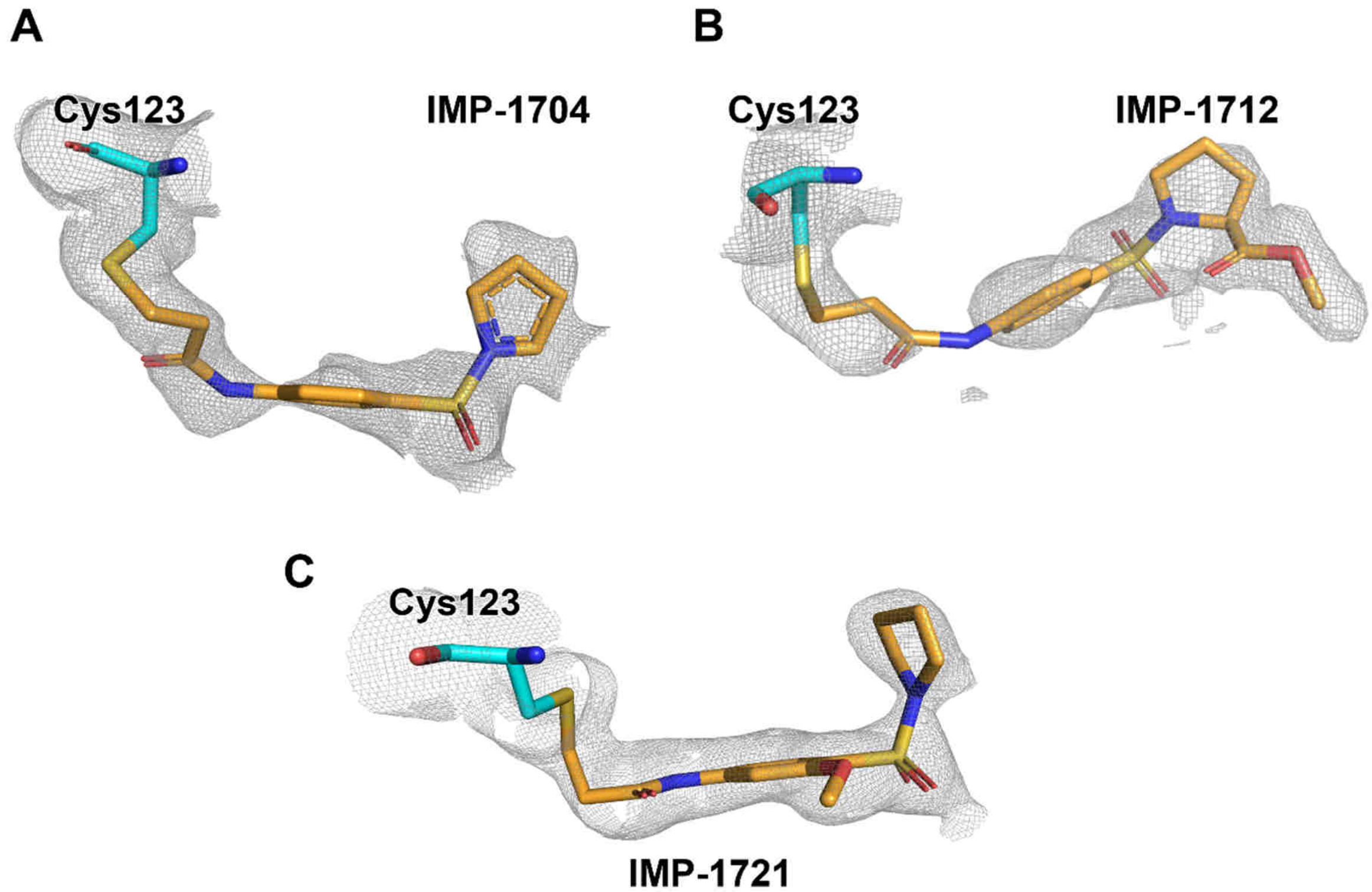

Figure S3. Electron density maps for covalent complexes of fRAB27A-Cys123 solved by X-ray crystallography. 2FO-FC electron density maps (grey) contoured at 1.0 σ for covalently bound ligands (orange) **IMP-1704** (**1**, A), **IMP-1712** (**2**, B) and **IMP-1721** (**14**, C).

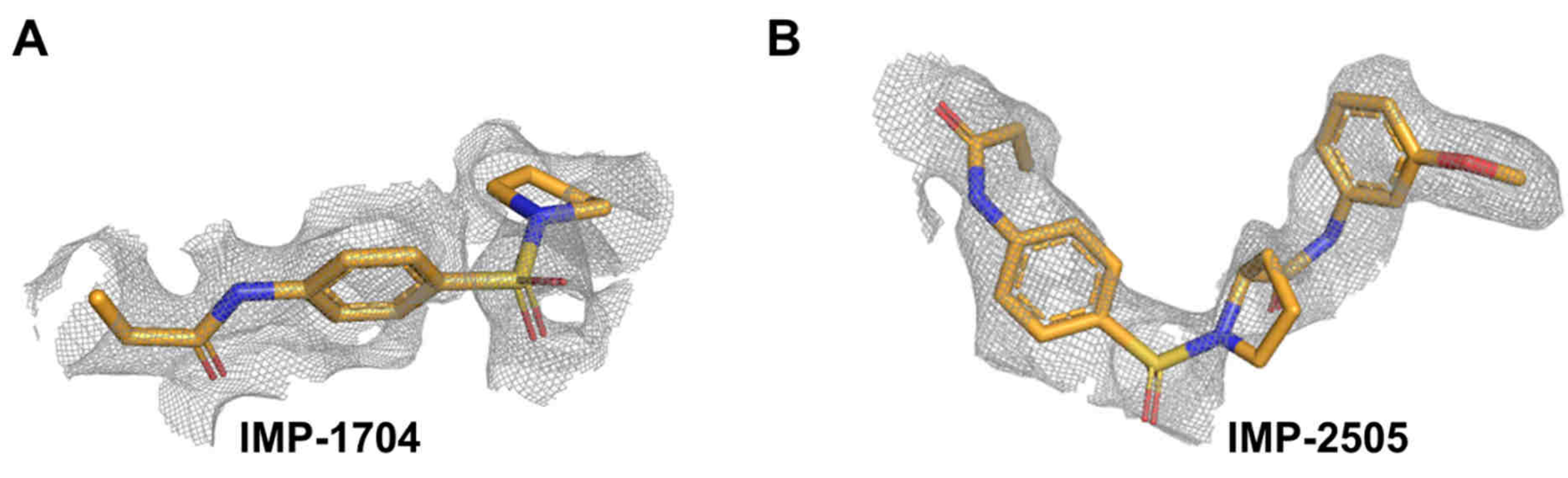

Figure S4. Electron density maps for non-covalent complexes of fRAB27A-Ala123 solved by X-ray crystallography. 2FO-FC electron density maps (grey) contoured at 1.0 σ for non-covalently soaked ligands (orange) **IMP-1704** (**1**, A), and **IMP-2505** (**13**, B).

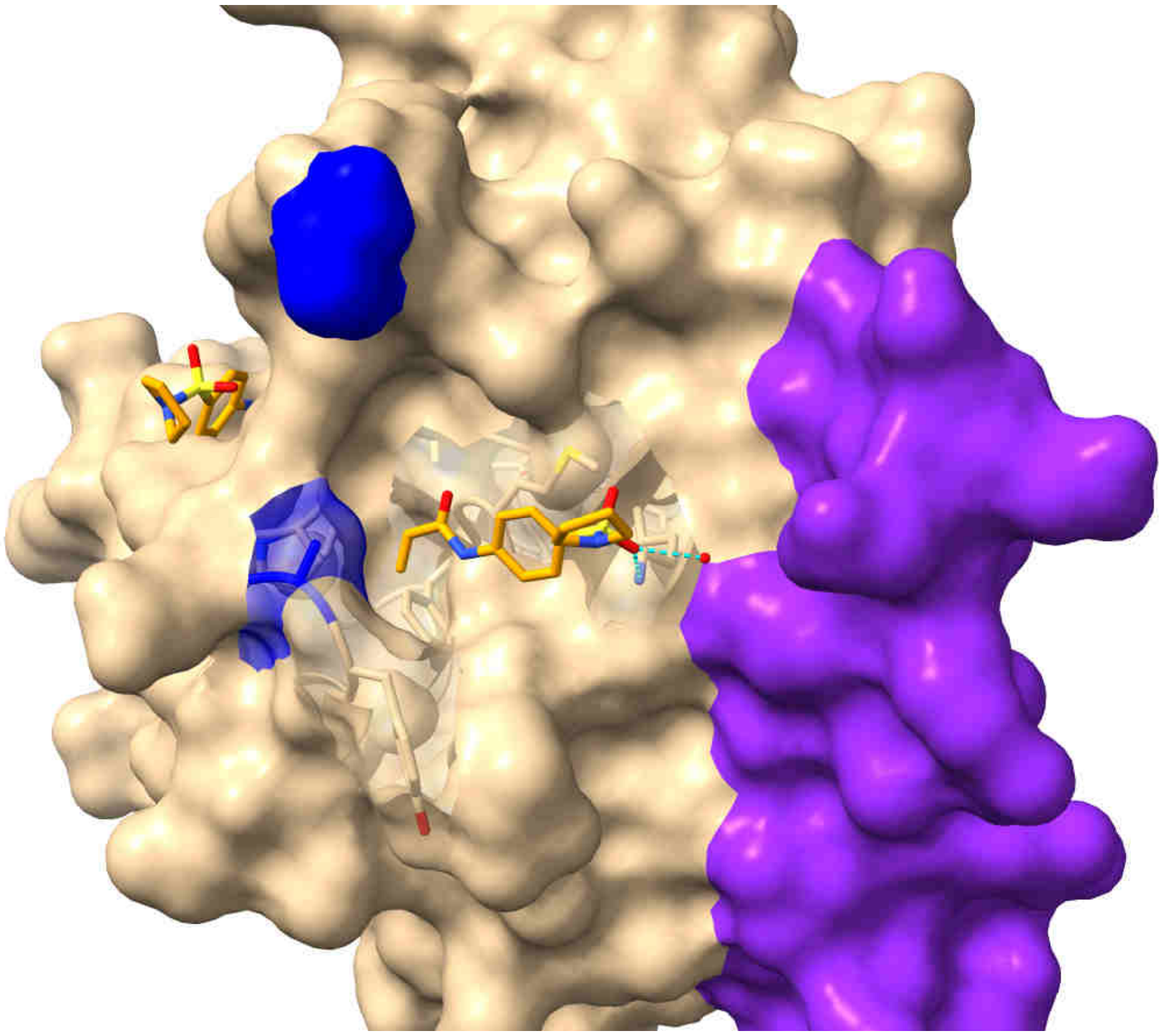

Figure S5. Non-covalent complex between fRAB27A-Ala123 and hit **1** (PDB: 8P3J) showing a second ligand-binding pocket.

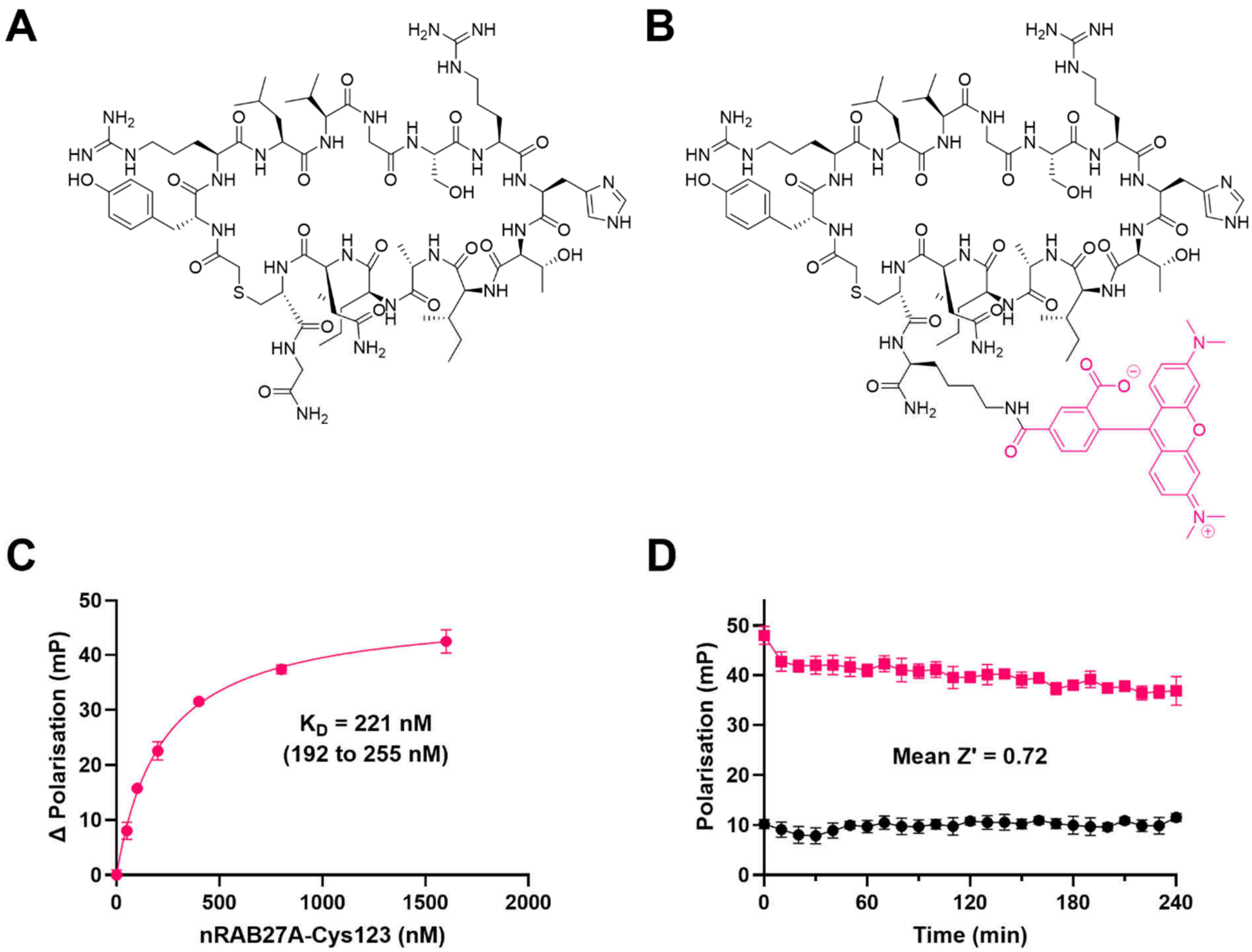

Figure S6. A) Chemical structure of **IMP2660**; B) Chemical structure of **IMP-2660-T**; C) K_D measurement for **IMP-2660-T** via fluorescence polarization, including 95% CI; D) Fluorescence polarization assay window under standard conditions (200 nM nRAB27A-Cys123, 35 nM **IMP-2660-T**).

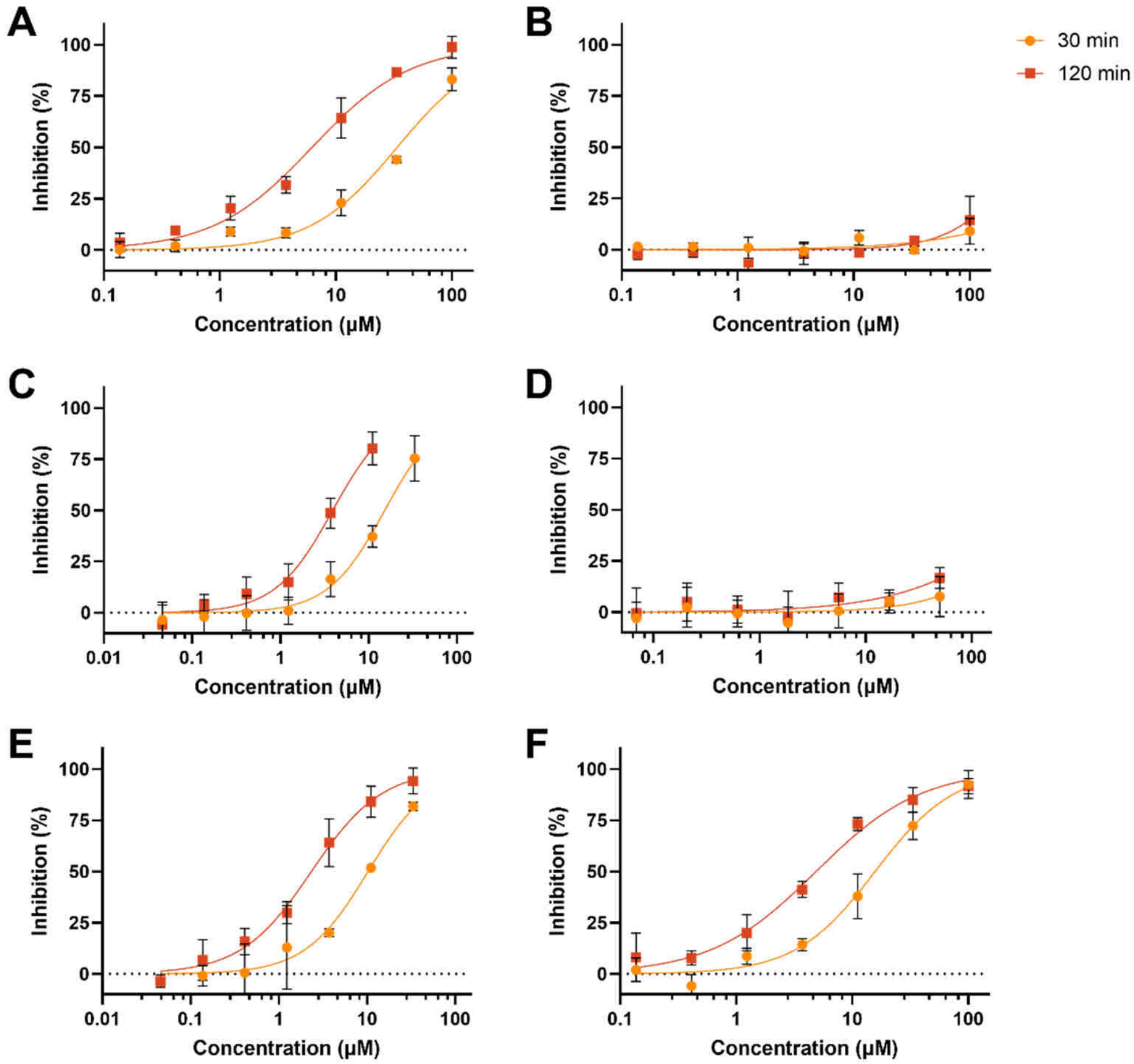

Figure S7. Representative fluorescence polarization (FP) dose-response curves at 30 and 120 min for compounds **7** (A), **8** (B), **10** (C), **11** (D), **12** (E), and **Nexinhib-20** (F). All compounds exhibit time-dependent inhibition, consistent with a covalent mechanism.

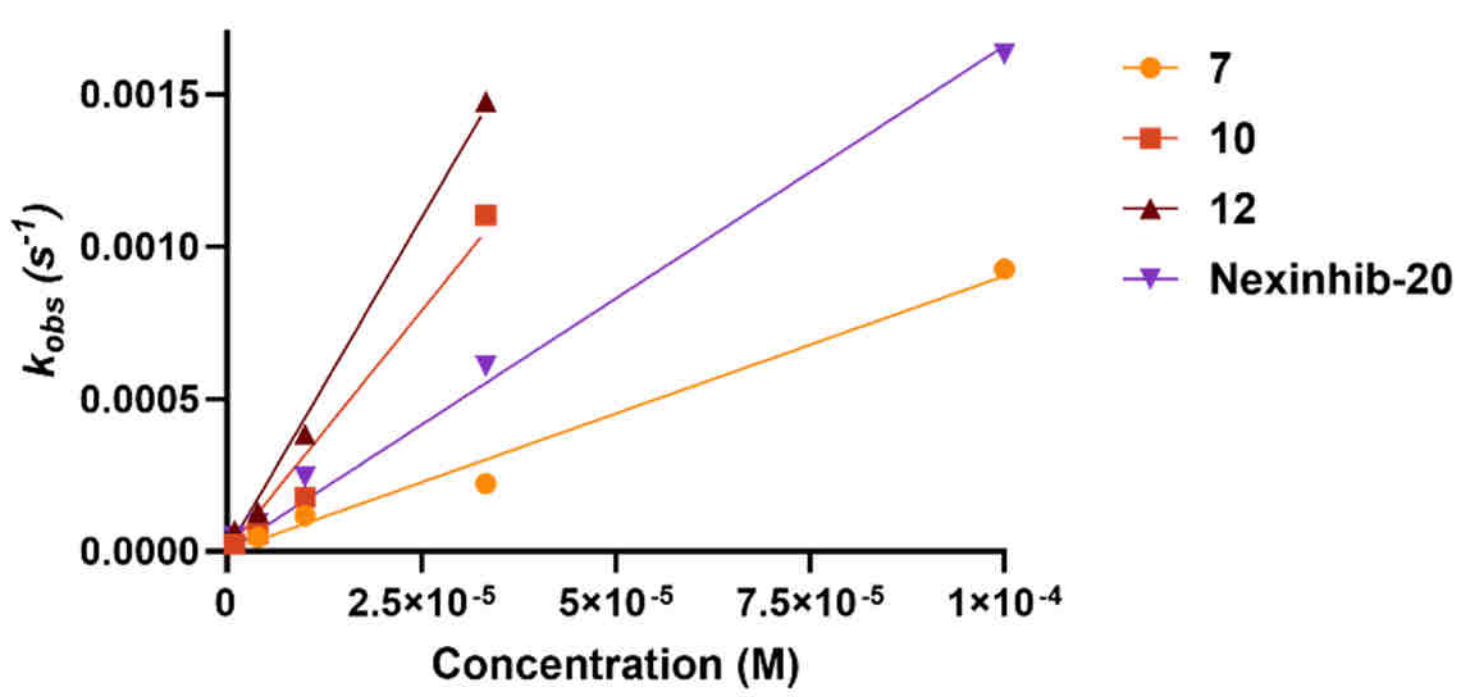

Figure S8. Representative fluorescence polarization (FP) kinetic data for compounds **7** (A), **8** (B), **10** (C), **11** (D), **12** (E), and **Nexinhib-20** (F).

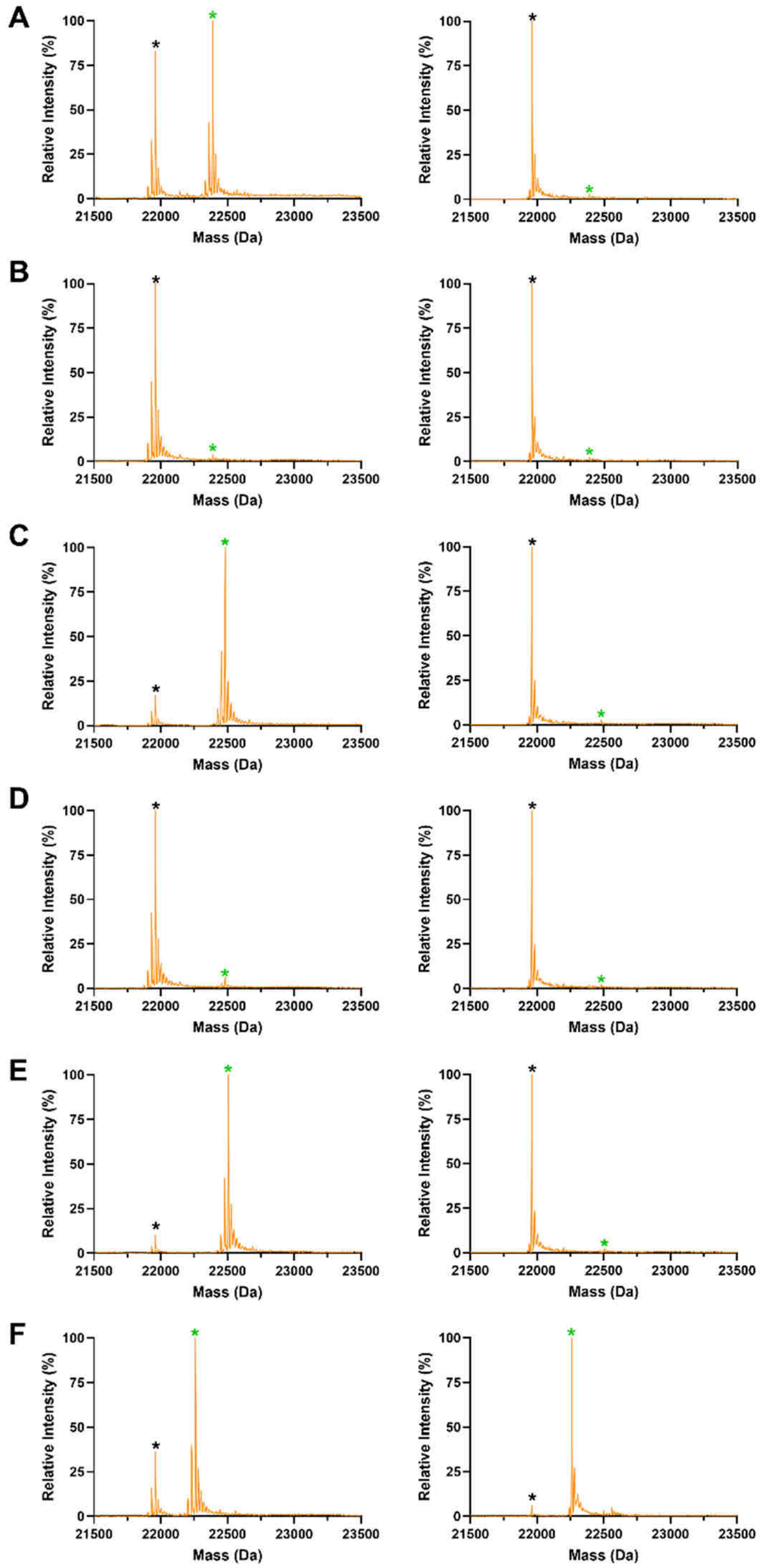

Figure S9. Representative intact protein MS analysis of 10 µM compound **7** (A), **8** (B), **10** (C), **11** (D), **12** (E), and **Nexinhib-20** (F) incubated for 1 h at rt with 500 nM nRAB27A-Cys123 (left) or nRAB27A-Cys188 (right). The black asterix indicates unlabelled protein and the green asterix indicates labelled protein.

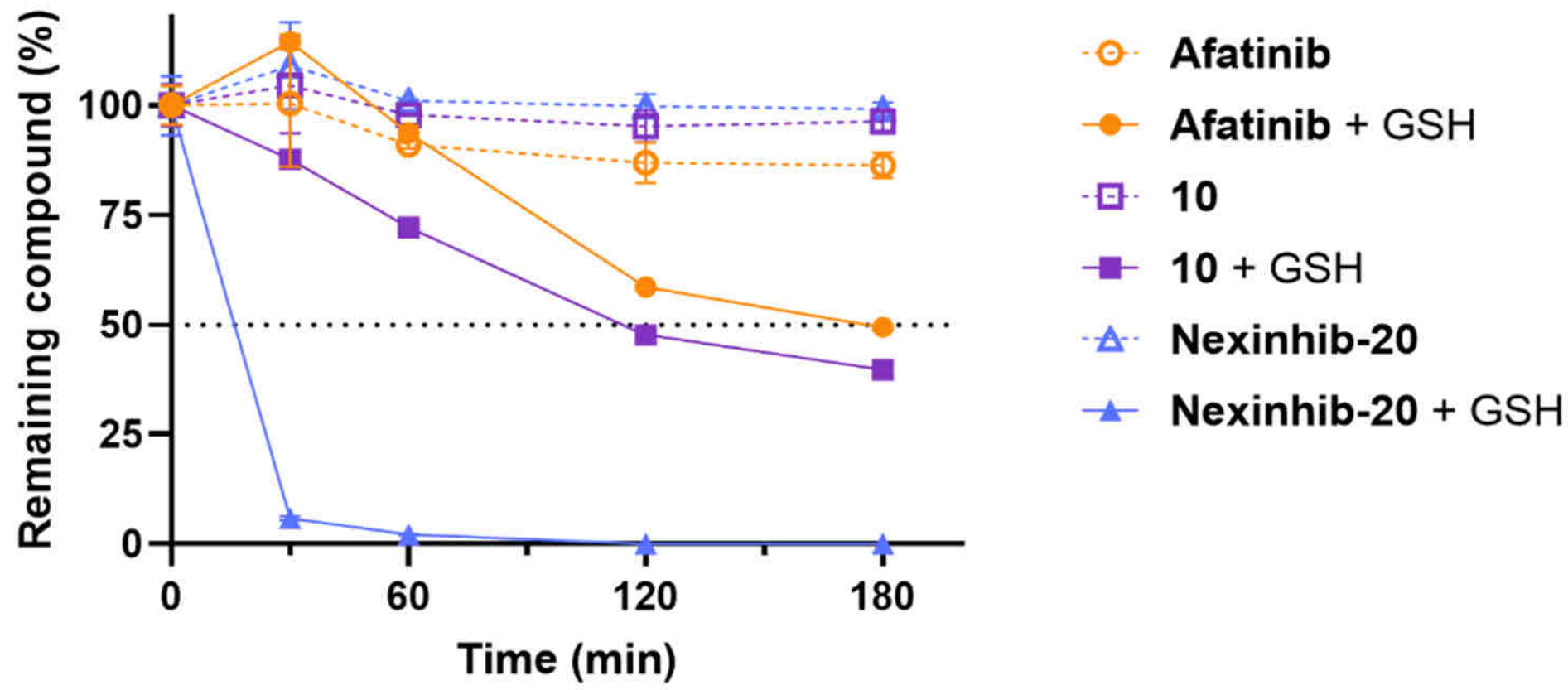

Figure S10. Glutathione (GSH) reactivity analysis for compound **10**, **Nexinhib-20**, and **Afatinib**. Conditions: 5 mM GSH, 35 °C, pH 7.4 in PBS with aliquots measured at the indicated timepoints.

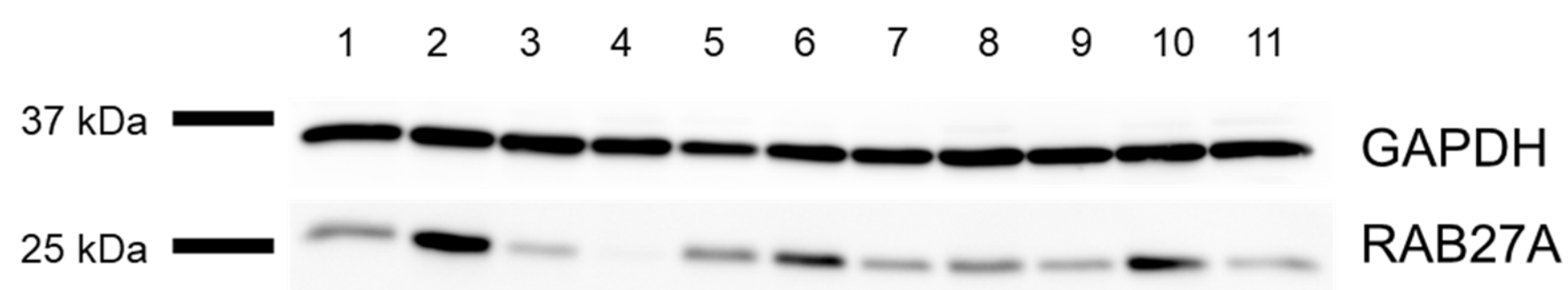

Figure S11. Analysis of RAB27A expression levels in a panel of breast and prostate cell lines by western blotting. *Lane 1* = MCF7; Lane 2 = MDA-MB-231; Lane 3 = SKBR3; Lane 4 = BT474; Lane 5 = T47D; Lane 6 = DU-145; Lane 7 = PC3; Lane 8 = LnCap; Lane 9 = Vcap; Lane 10 = BxPC3; Lane 11 = Capan2.

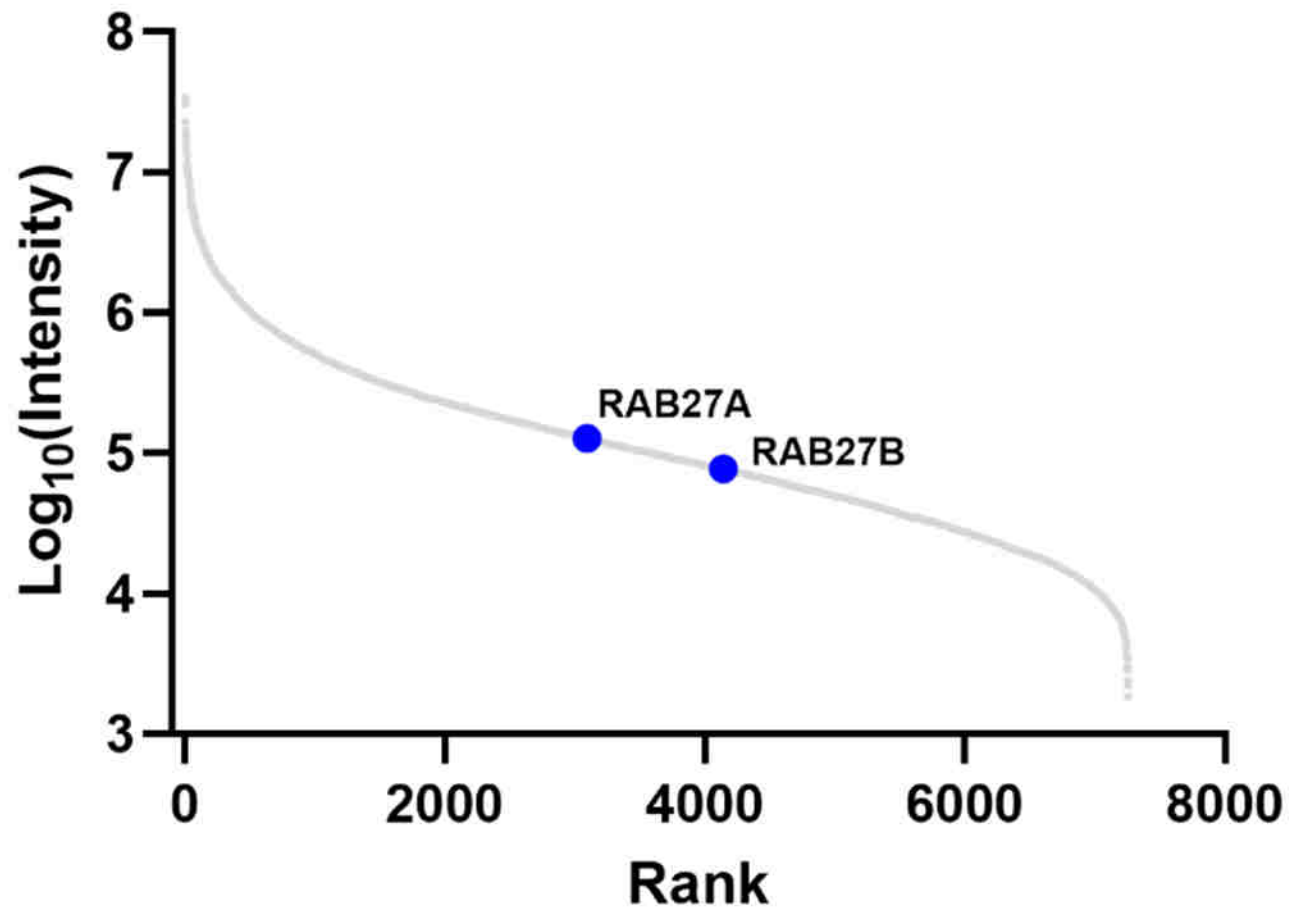

Figure S12. Analysis of RAB27A and RAB27B expression levels by whole proteome analysis in MDA-MB-231 showing moderate expression.

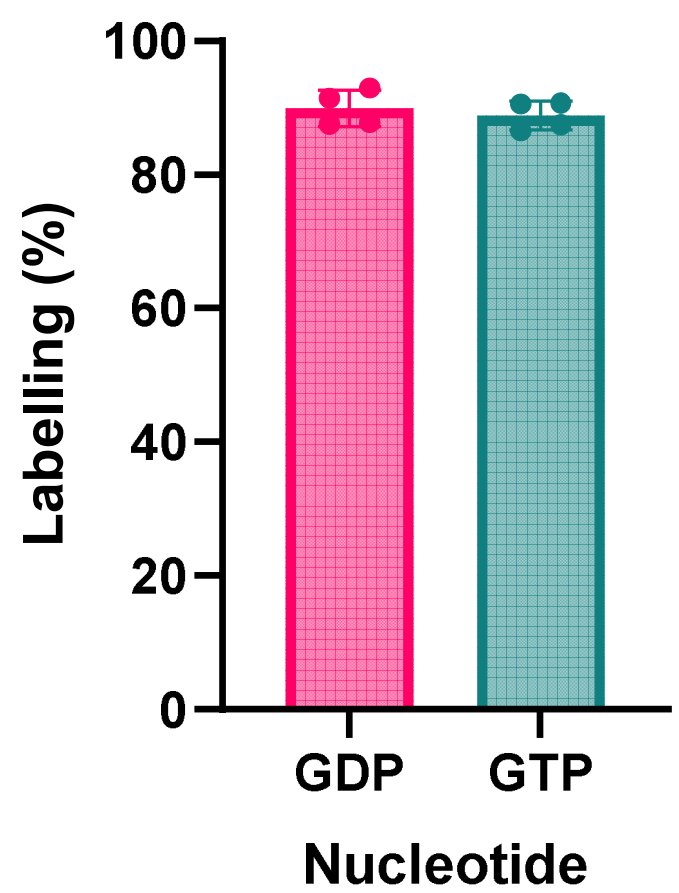

Figure S13. Labelling efficiency for compound **10** against GDP- or GTP-bound nRAB27A-Cys123(L78) determined by intact protein MS.

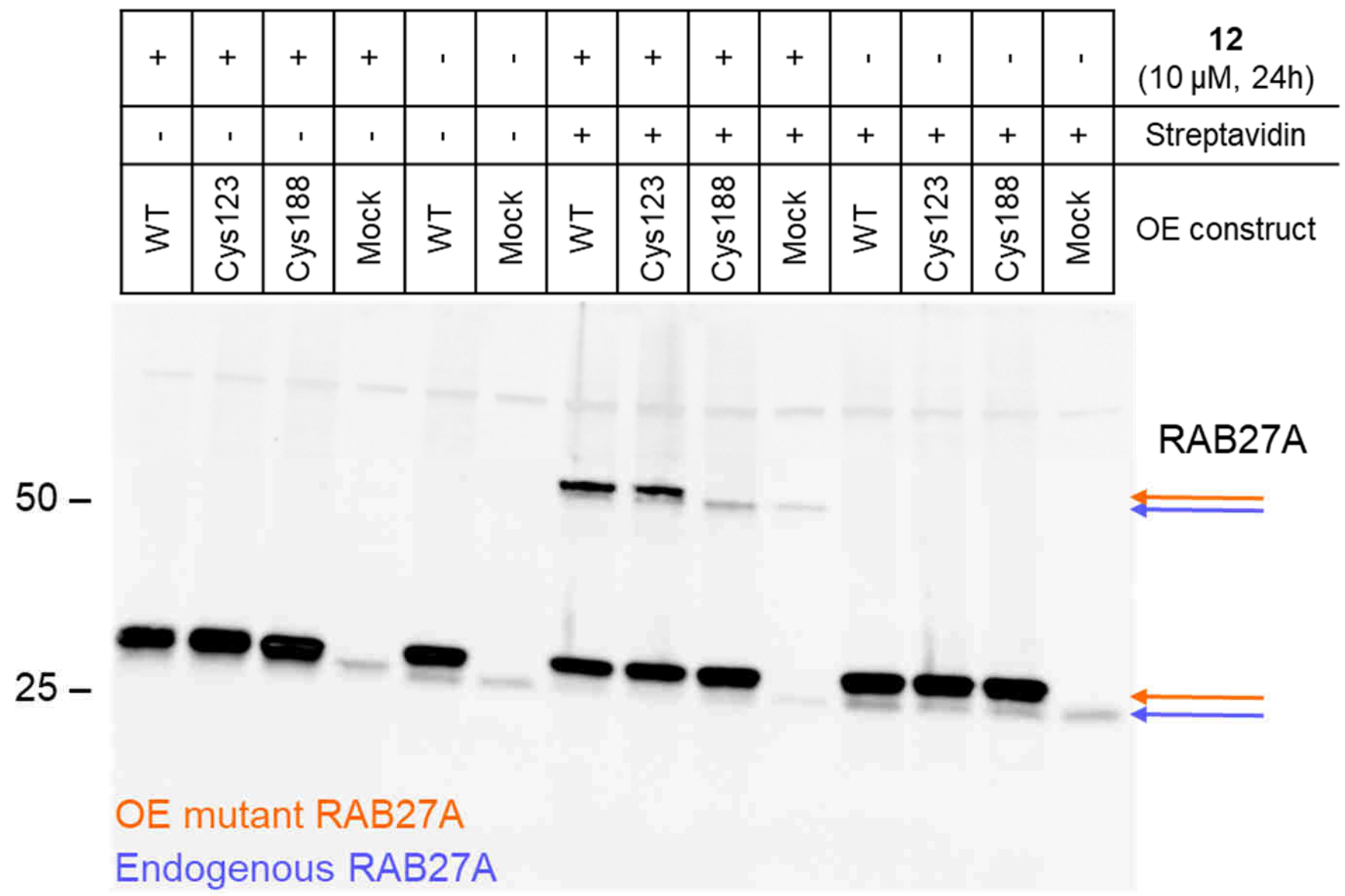

Figure S14. Western blot analysis of streptavidin-shift assay using α-RAB27A antibody. Two close bands corresponding to endogenous RAB27A (lower) and the overexpressed (OE) FLAG-tagged mutant specified (upper band) are observed. Only mutants containing Cys123 are engaged by **12**, hence the OE mutant shifted band is only appearing in WT and Cys123 lanes, while engaged endogenous RAB27A is present in all treated samples. This is the same blot as in Figure 6E after stripping. WT= FLAG-RAB27A (1–221, **P51159**); Cys123= FLAG-RAB27A (1–221, **Cys188Ala**); Cys188= FLAG-RAB27A (1–221, **Cys123Ala**); Mock= no DNA control.

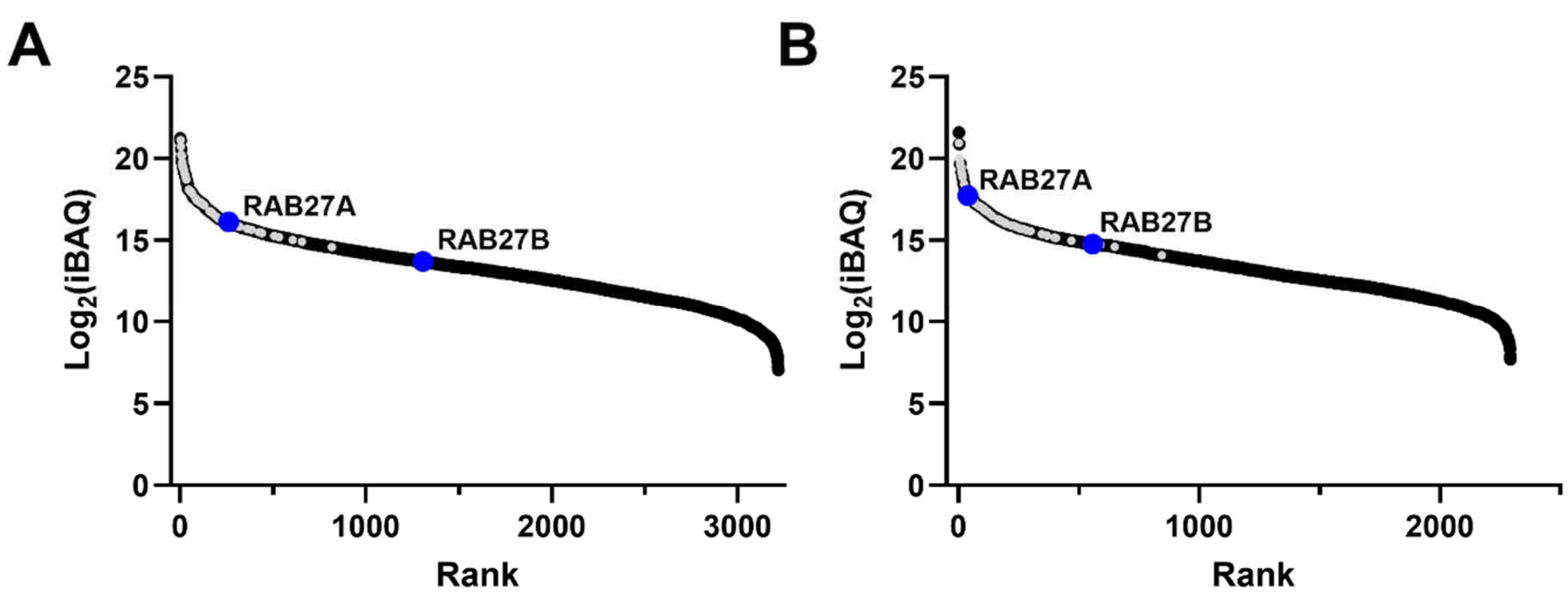

Figure S15. iBAQ-normalized S-plots showing the ranked enrichment of protein targets engaged by probes **9** (A) and **12** (B) in chemical proteomics experiments. Proteins shown in grey were enriched in probe treated samples and also detected in DMSO-treated controls, likely reflecting non-specific, sticky, or highly abundant proteins rather than true targets.

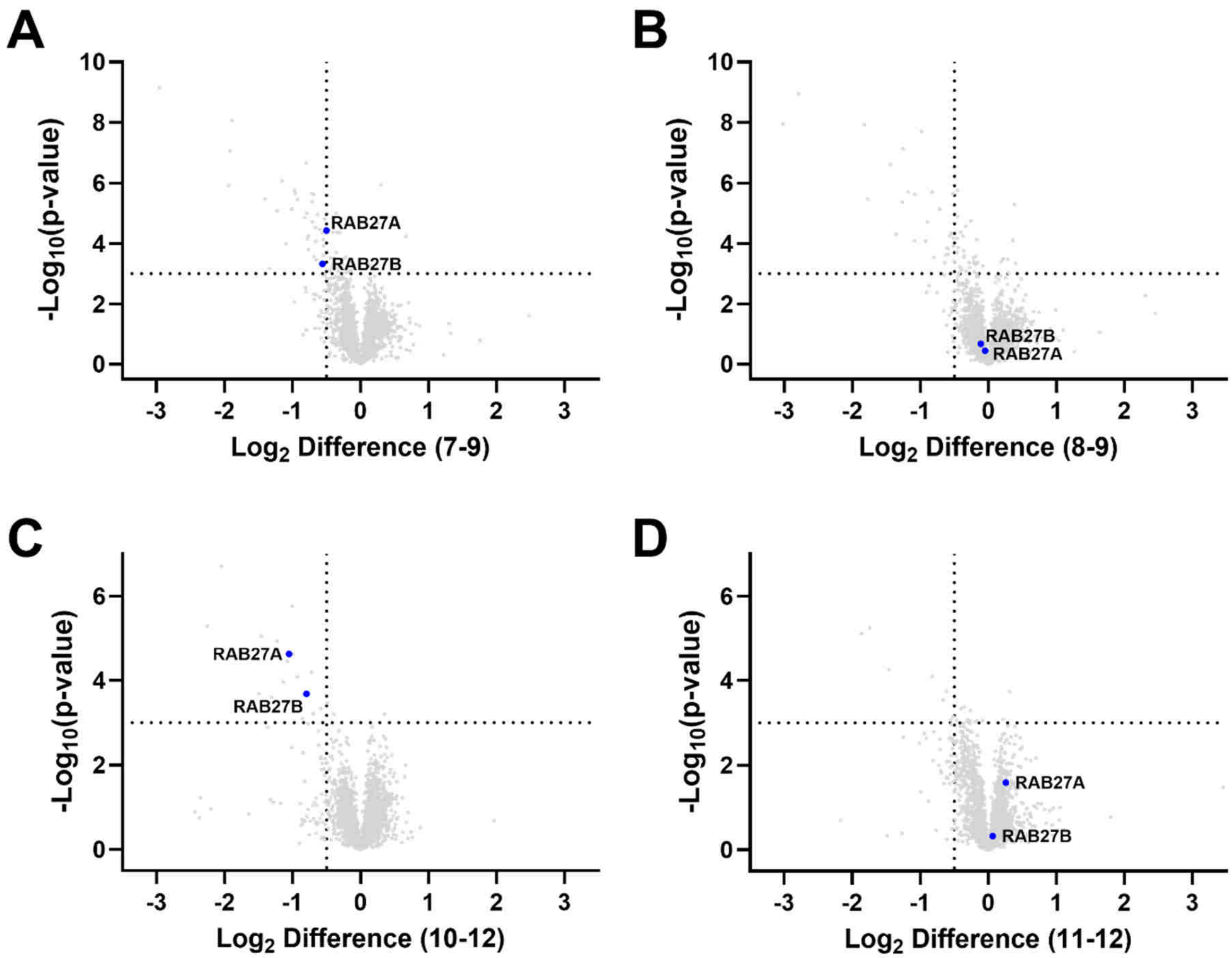

Figure S16. Volcano plots for chemical proteomics competition experiments showing the difference between targets competed by 20 µM **7** (A) or inactive control **8** (B) against 10 µM probe **9**, and targets competed by 10 µM **10** (C) or inactive control **11** (D) against 5 µM probe **12**. Treatment conditions: Parent or inactive control incubated for 2 h followed by probe incubation for 2 h.

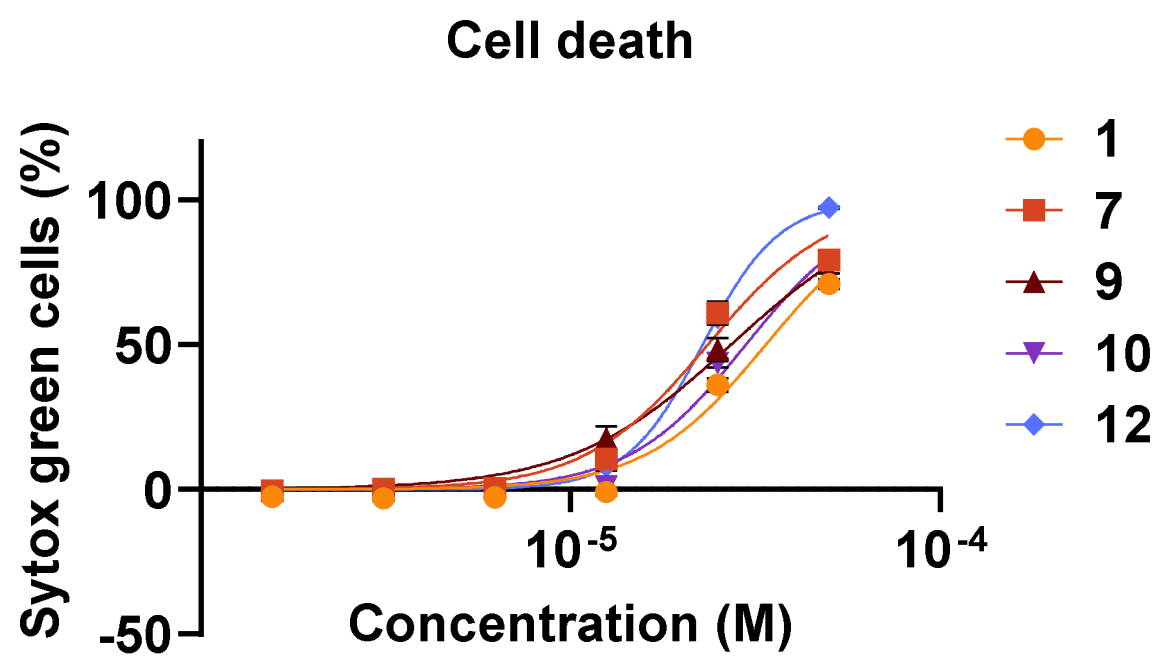

Figure S17. Cell death analysis following 72 h treatment in MDA-MB-231 cells with compounds obtained via Incucyte® live-cell imaging using sytox green to count death cells.

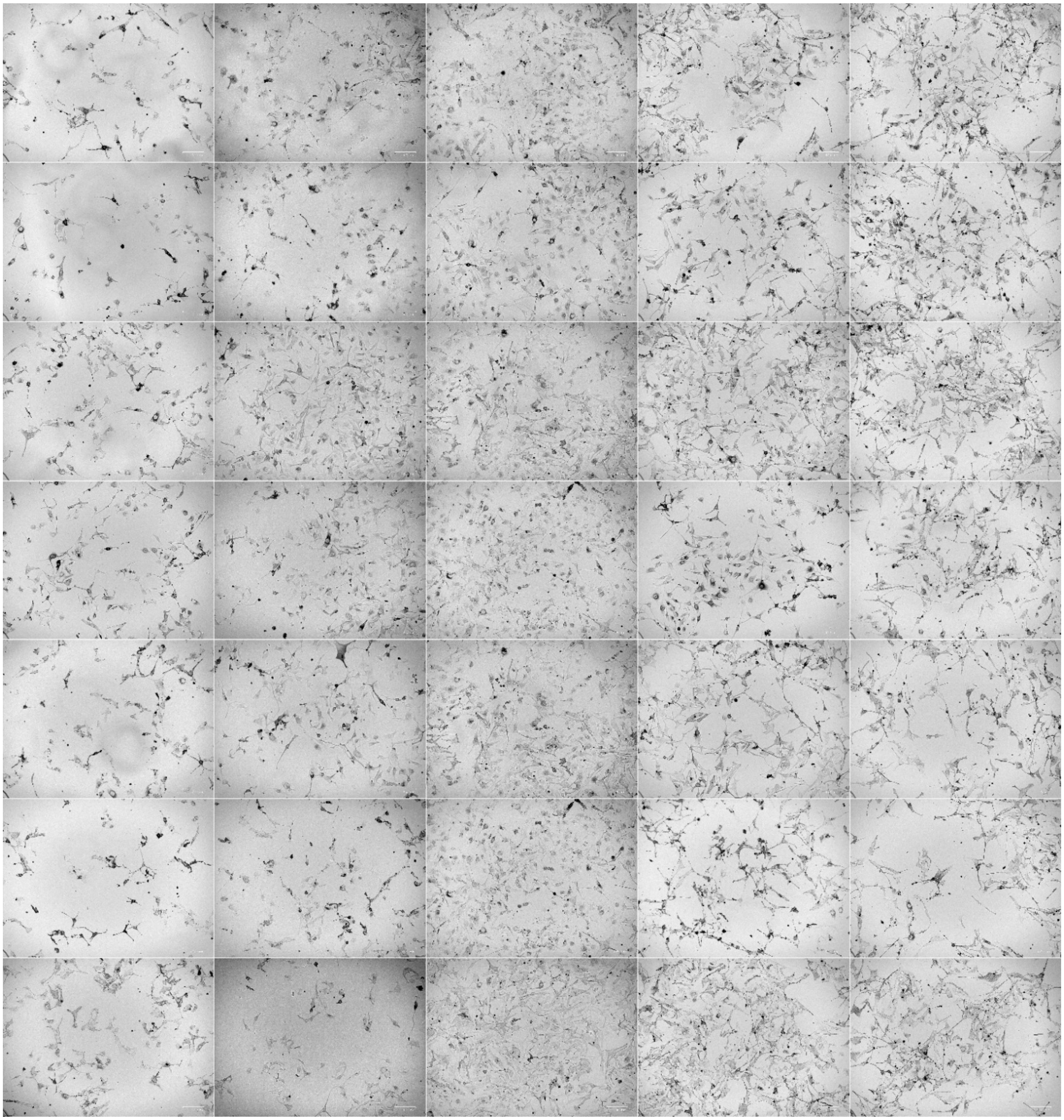

Figure S18. Montage of mouse melanocyte images used for quantification analyses shown in Figure 7B. Conditions array is displayed in the table below for the 5 biological replicates:

**Cmpd 10, 5 µM**	**R #1**	**R #2**	**R #3**	**R #4**	**R #5**
**Cmpd 10, 10 µM**	**R #1**	**R #2**	**R #3**	**R #4**	**R #5**
**Cmpd 12, 5 µM**	**R #1**	**R #2**	**R #3**	**R #4**	**R #5**
**Cmpd 12, 10 µM**	**R #1**	**R #2**	**R #3**	**R #4**	**R #5**
**Cmpd 11, 5 µM**	**R #1**	**R #2**	**R #3**	**R #4**	**R #5**
**Cmpd 11, 10 µM**	**R #1**	**R #2**	**R #3**	**R #4**	**R #5**
**DMSO**	**R #1**	**R #2**	**R #3**	**R #4**	**R #5**

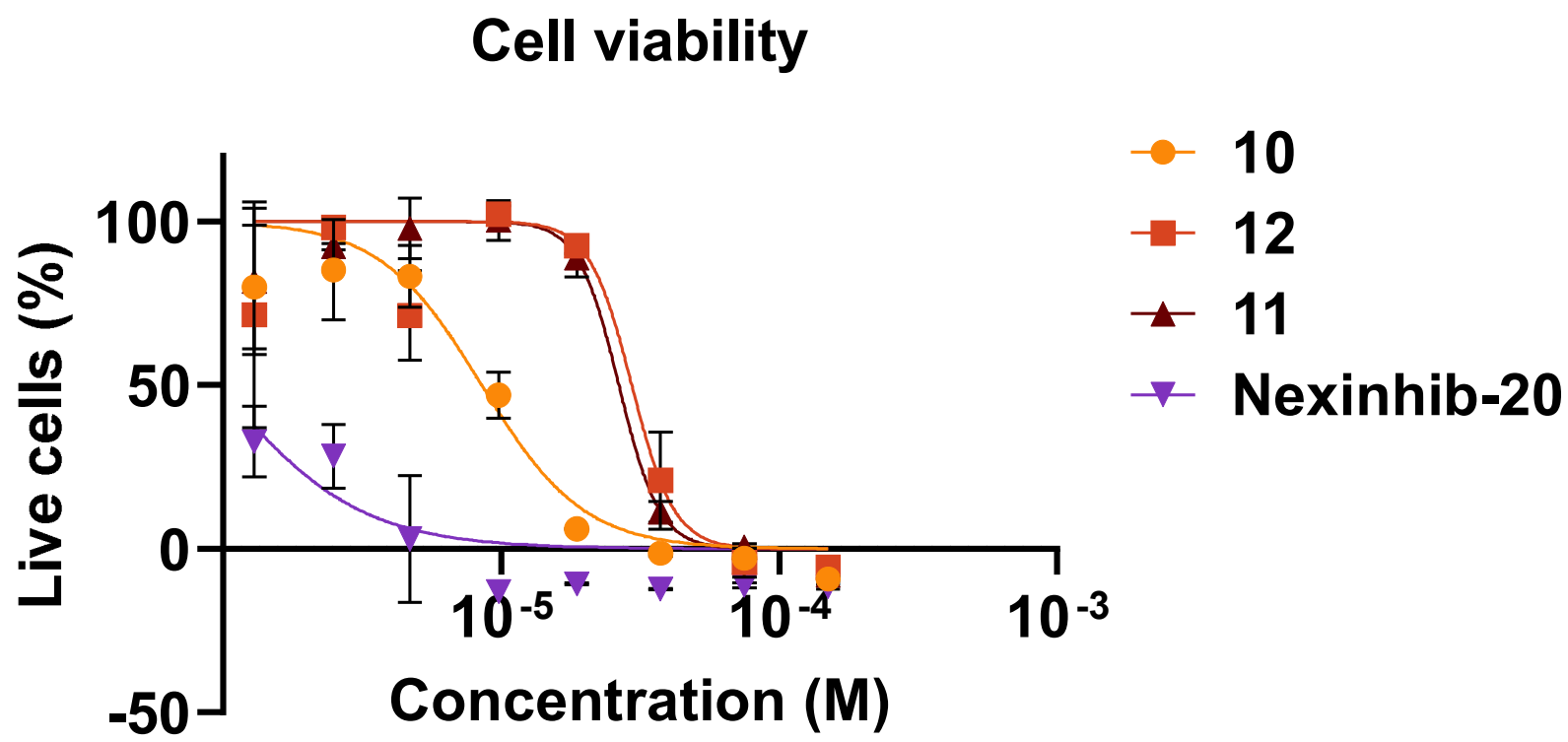

Figure S19. Melan-a cell viability following 24 h treatment with specified compound, as determined by Prestoblue assay (ThermoFisher A13262) according to the manufacturer protocol.

Supplementary Tables

Table S1. Data Processing and Refinement Statistics for fusion hSLP2-A_RAB27A-Cys123 bound to **IMP1704** (**1**) (PDB: 8P3G).

	**8P3G**
**Data collection**	
Wavelength (Å)	0.9795
Space group	$P2_12_12_1$
Unit cell (Å, °)	a = 62.30, b = 76.03, c = 118.85; α = β = γ = 90
Resolution (Å)	55.18–2.13 (2.21–2.13)
Total reflections	64,540
Unique reflections	32,270 (3,162)
Multiplicity	2.0 (2.0)
Completeness (%)	99.72 (99.06)
⟨I/σ(I)⟩	4.2
CC1/2	0.981 (0.344)
Wilson B (Å^2)	26.37
**Refinement**	
Reflections (work/test)	32,207 / 1,614
Rwork / Rfree	0.193 / 0.243 (0.261 / 0.314)
No. atoms	3,833
Protein residues	418
RMS bonds (Å)	0.007
RMS angles (°)	0.86
Ramachandran favored (%)	97.26
Ramachandran allowed (%)	2.74
Ramachandran outliers (%)	0.00
Rotamer outliers (%)	0.28
Clashscore	3.75
**Average B-factors (Å^2)**	
Macromolecules	31.91
Ligands	33.98
Solvent	35.90
Overall	32.36

Values in parentheses correspond to the highest-resolution shell.

Table S2. Data Processing and Refinement Statistics for fusion hSLP2-A_RAB27A-Cys123 bound to **IMP1712** (**2**) (PDB: 8P3H)

	**8P3H**
**Data collection**	
Wavelength (Å)	0.9795 (Diamond I04)
Space group	$P2_12_12_1$
Unit cell (Å, °)	a = 60.1723, b = 77.6959, c = 116.15; α = β = γ = 90
Resolution (Å)	58.08 – 2.76 (2.859 – 2.76)
Total reflections	14,556
Unique reflections	14,556 (1,419)
Multiplicity	2.0 (2.0)
Completeness (%)	99.68 (98.95)
⟨I/σ(I)⟩	5.21
CC1/2	0.972 (0.227)
Wilson B (Å^2)	56.54
**Refinement**	
Reflections (work/test)	14,514 / 723 (1,408 / 71)
Rwork / Rfree	0.227 / 0.313 (0.321 / 0.353)
Non-H atoms	3,459
— Macromolecules	3,264
— Ligands	148
— Solvent	47
Protein residues	409
RMSD bonds (Å)	0.009
RMSD angles (°)	1.06
Ramachandran favored (%)	92.07
Ramachandran allowed (%)	7.16
Ramachandran outliers (%)	0.77
Rotamer outliers (%)	0.00
Clashscore	17.79
**Average B-factors (Å^2)**	
Macromolecules	64.90
Ligands	62.39
Solvent	58.24
Overall	64.70

Values in parentheses correspond to the highest-resolution shell.

Table S3. Data Processing and Refinement Statistics for fusion hSLP2-A_RAB27A-Cys123 bound to **IMP1721** (**14**) (PDB: 8P3I)

	**8P3I**
**Data collection**	
Wavelength (Å)	0.9763 (Diamond I03)
Space group	$P2_12_12_1$
Unit cell (Å, °)	a = 61.3673, b = 76.6009, c = 118.674; α = β = γ = 90
Resolution (Å)	64.36 – 2.00 (2.071 – 2.00)
Total reflections	~73,200* (calculated from redundancy × unique)
Unique reflections	38,565 (3,801)
Multiplicity	1.9 (1.9)
Completeness (%)	87.98 (21.07)
⟨I/σ(I)⟩	3.39
CC1/2	0.997 (0.331)
Wilson B (Å^2)	47.15
**Refinement**	
Reflections (work/test)	33,930 / 1,757 (801 / 48)
Rwork / Rfree	0.2325 / 0.2796 (0.4565 / 0.5171)
Non-H atoms	3,586
— Macromolecules	3,337
— Ligands	192
— Solvent	57
Protein residues	426
RMSD bonds (Å)	0.008
RMSD angles (°)	1.05
Ramachandran favored (%)	96.36
Ramachandran allowed (%)	3.64
Ramachandran outliers (%)	0.00
Rotamer outliers (%)	0.29
Clashscore	7.09
**Average B-factors (Å^2)**	
Macromolecules	58.03
Ligands	57.36
Solvent	55.10
Overall	57.95

Values in parentheses correspond to the highest-resolution shell.

Table S4. Data Processing and Refinement Statistics for fusion hSLP2-A_RAB27A-Ala123 bound to **IMP1704** (**1**) (PDB: 8P3I)

	**8P3J**
**Data collection**	
Wavelength (Å)	0.9795 (Diamond I04)
Space group	$P2_12_12_1$
Unit cell (Å, °)	a = 62.4364, b = 75.8281, c = 118.53; α = β = γ = 90
Resolution (Å)	63.88 – 2.16 (2.237 – 2.16)
Total reflections	30,872
Unique reflections	30,871 (3,056)
Multiplicity	1.9 (1.9)
Completeness (%)	96.39 (76.33)
⟨I/σ(I)⟩	2.8
CC1/2 (overall)	0.972 (0.325)
Wilson B (Å^2)	15.21
**Refinement**	
Reflections (work/test)	29,767 / 1,522 (2,335 / 120)
Rwork / Rfree	0.2847 / 0.3508 (0.6497 / 0.7102)
Non-H atoms	3,686
— Macromolecules	3,429
— Ligands	160
— Solvent	97
Protein residues	426
RMSD bonds (Å)	0.008
RMSD angles (°)	1.02
Ramachandran favored (%)	95.45
Ramachandran allowed (%)	3.59
Ramachandran outliers (%)	0.96
Rotamer outliers (%)	0.00
Clashscore	10.10
**Average B-factors (Å^2)**	
Macromolecules	28.63
Ligands	34.28
Solvent	23.07
Overall	28.73

Values in parentheses correspond to the highest-resolution shell.

Table S5. Data Processing and Refinement Statistics for fusion hSLP2-A_RAB27A-Ala123 bound to **IMP2505** (**13**) (PDB: 8P3K)

	**8P3K**
**Data collection**	
Wavelength (Å)	0.9795 (Diamond I04)
Space group	$P2_12_12_1$
Unit cell (Å, °)	a = 60.8957, b = 76.5361, c = 117.795; α = β = γ = 90
Resolution (Å)	47.65 – 2.58 (2.672 – 2.58)
Total reflections	17,938
Unique reflections	17,938 (1,736)
Multiplicity	6.6 (6.6)
Completeness (%)	98.75 (94.77)
⟨I/σ(I)⟩	4.17
CC1/2 (overall)	0.959 (0.216)
Wilson B (Å^2)	42.54
**Refinement**	
Reflections (work/test)	17,722 / 859 (1,650 / 74)
Rwork / Rfree	0.2410 / 0.3143 (0.3642 / 0.4219)
Non-H atoms	3,565
— Macromolecules	3,340
— Ligands	186
— Solvent	39
Protein residues	421
RMSD bonds (Å)	0.009
RMSD angles (°)	1.06
Ramachandran favored (%)	91.04
Ramachandran allowed (%)	8.23
Ramachandran outliers (%)	0.73
Rotamer outliers (%)	0.00
Clashscore	17.51
**Average B-factors (Å^2)**	
Macromolecules	46.97
Ligands	50.13
Solvent	37.91
Overall	47.03

Values in parentheses correspond to the highest-resolution shell.

Abbreviations

AzB: azidobiotin

BME: β-mercaptoethanol

DIPEA: diisopropylethylenediamine

DMEM: Dulbecco's modified Eagle medium

DMF: dimethylformamide

DMSO: dimethylsulfoxide

DTT: dithiothreol

EDCI: 1-ethyl-3-(3-dimethylaminopropyl)carbodiimide

FP: fluorescence polarization

GAPDH: glyceraldehyde 3-phosphate dehydrogenase

GDP: guanosine diphosphate

GTP: guanosine triphosphate

GppNHp: guanosine 5′-(β,γ-imino)triphosphate

HATU: hexafluorophosphate Azabenzotriazole Tetramethyl Uronium

HBTU: hexafluorophosphate Benzotriazole Tetramethyl Uronium

HEPES: 4-(2-hydroxyethyl)-1-piperazineethanesulfonic acid

HOBt: hydroxybenzotriazole

HRMS: high resolution mass spectrometry

IPTG: isopropyl β-D-1-thiogalactopyranoside

LB: lysogeny broth

LC/Q-TOF: liquid chromatography/quadrupole time-of-flight

LRMS: low resolution mass spectrometry

NTA: nitrilotriacetic acid

MES: 2-(N-morpholino)ethanesulfonic acid

PBS: phosphate-buffered saline

PCR: polymerase chain reaction

qIT: quantitative irreversible tethering

Rab: Ras-related protein in brain

SDS-PAGE: sodium dodecyl sulfate-polyacrylamide gel electrophoresis

TAMRA: carboxytetramethylrhodamine

TCEP: tris(2-carboxyethyl)phosphine

TEV: Tobacco Etch Virus

TIC: total ion chromatograms

THF: tetrahydrofuran

Tris: tris(hydroxymethyl)aminomethane

Biological Procedures

Protein expression and purification

As previously reported,[1] all RAB27A constructs contain the sequence for human RAB27A (P51159, residues 1–192, mutations: Q78L and/or C123S/A or C188S or both as specified), which was cloned into a pET15b vector (Invitrogen) including a N-terminal His-tag followed by a Tobacco Etch Virus (TEV) recognition site (ENLYFQ¦G). Fusion constructs also contain the C-terminus of SLP2-A SHD1 (SFLTEEEQEAIMKVLQRDAALKRAEEER (residues 5–32 in the fusion)) linked to the N-terminus of RAB27A via a flexible poly glycine-serine linker (GSGSGSG). nRAB27B-Cys123 constructs contain the sequence for human RAB27B (O00194, residues 1–193, mutation: C188A), cloned in the same vector.
For protein expression, plasmids were transformed to E. coli BL21 cells and spread on LB agar plates containing 100 mg/L Ampicillin for selection. Single colonies were picked for amplification and incubated overnight into LB media containing 100 mg/L Ampicillin at 37 °C, shaking. Big scale cultures were inoculated using these overnight cultures at 1% v/v and grown at 37 °C until absorbance at 600 nm reached 0.6–0.8. Protein expression was induced using 0.5–1 mM isopropyl β-D-1-thiogalactopyranoside (IPTG) for 3 hours at 37 °C. Subsequently cells were pelleted at 5,000 ×g for 15 min, then re-suspended in lysis buffer containing 500 mM NaCl, 10 mM imidazole, 5 mM $MgCl_2$ and 50 mM Tris at pH 8. Cells were lysed with a cell disruptor at 25K psi and centrifuged at 17,000 ×g for 45 min. The supernatant was loaded on Ni^{2+}-NTA resin equilibrated with lysis buffer, washed extensively and eluted using buffer containing 500 mM NaCl, 300 mM imidazole, 5 mM $MgCl_2$ and 50 mM Tris at pH 8. The protein was buffer exchanged using 2x 5 mL Hitrap columns (Cytiva) in tandem with 100 mM NaCl, 5 mM $MgCl_2$ and 50 mM Tris, pH 8. Afterwards TEV protease (obtained in-house as previously described[2]) was added to the protein solution at a molar ratio of 1/20 in the presence of 1 mM DTT, and the solution was incubated overnight at 4 °C, shaking. The solution was clarified by centrifugation (10 min at 10,000 ×g at 4 °C) and buffer exchanged using 2x 5 mL Hitrap columns (Cytiva) in tandem with 100 mM NaCl, 5 mM MgCl2 and 50 mM Tris, pH 8. The solution was then loaded on Ni^{2+}-NTA resin and the flowthrough was collected, concentrated to 5.5 mg/mL in 150 mM NaCl, 5 mM $MgCl_2$, 20 mM Tris pH 8 buffer. If GppNHp loading was required, a 10X buffer containing 10 mM $ZnCl_2$ and 2 M $(NH_4)_2SO_4$, 4 molar excess of GppNHp and 25 units of Antarctic phosphatase (New England Biolabs) were added to the solution and incubated overnight at 4 °C. Finally, the sample was loaded on a Superdex S-75 gel filtration column at a flow rate of 1 mL/min. The column was pre-equilibrated with 150 mM NaCl, 5 mM $MgCl_2$, and 20 mM Tris at pH 8 for crystallography or 20 mM HEPES at pH 8 for biochemical assays. The peaks corresponding to RAB27A constructs were analysed by SDS-PAGE, pooled, concentrated and flash frozen using liquid nitrogen.

Protein labelling and purification

To a 15 mL falcon tube were added 600 µL of desired construct (100 µM stock), 100 µL of 50% w/v TCEP-agarose beads (ThermoFisher), and 1.8 mL of 100 mM NaCl, 5 mM $MgCl_2$, and 20 mM HEPES pH 8.0. 50 µL of ligand (50 mM stock) were diluted with 2.45 mL of 100 mM NaCl, 5 mM $MgCl_2$, and 20 mM HEPES pH 8.0, followed by centrifugation (2,400 rpm, 5 min). The supernatant was added to the protein mixture and incubated at 4 °C. The labelled protein solution was concentrated to 0.5 mL by using a Vivaspin 20 filter (5,000 MWCO). The protein was diluted with 4.5 mL of 100 mM NaCl, 5 mM $MgCl_2$, and 20 mM HEPES pH 8.0 and concentrated again to 0.5 mL (5x), to remove excess compound, then purified by superdex S-75 gel filtration at a flow rate of 1 mL/min in 150 mM NaCl, 5 mM MgCl2, and 20 mM Tris at pH 8 for crystallography.

Protein crystallisation

Covalent complexes: Pure samples of labelled protein were concentrated between 4–12 mg/mL, in buffer containing 150 mM NaCl, 5 mM $MgCl_2$, and 20 mM Tris at pH 8.0. Crystals were grown at 4 °C using the sitting-drop vapor-diffusion method with a mother liquor containing 150 mM NH_4SO_4, 100 mM MES, and 15% PEG 4K, pH 6.0. Crystals were harvested in 30% glycerol.

Noncovalent soaking (fRAB27A, C123A mutant): Pre-formed fRAB27A-A123 crystals using conditions above were incubated for 2 h at 4 °C with 1 µL of a solution of the covalent ligand at 6 mM in 12% DMSO.

Crystal diffraction, data collection and data processing

Data collections were carried out at Diamond Light Source in Oxford using the i03 or i04 beamline at 100K, using Pilatus detector. Data were collected at 0.4°-0.5° oscillations per image with a total oscillation up to 200°. Data processing was carried out using Dials. Initial phases were calculated using the molecular replacement program phaser. The coordinates of RAB27A from chain A of the RAB27a-SLP2-A complex (PDB:3BC1) without the nucleotide and the magnesium ion were used as the search model. Subsequently, the initial model generated by phaser was refined through an iterative cycle using COOT and REFMAC5. Final model structures were validated using the Molprobity server at http://molprobity.biochem.duke.edu. All structure images were prepared using Pymol. X-ray data collection, processing and refinement statistics are given in Table S1–S5.

qIT assay for screening and hit validation

960 electrophilic acrylamides were screened in the qIT assay adapted from Craven et al[3]. Briefly, the reaction buffer (20 mM HEPES pH 8.0, 100 mM NaCl, 5 mM $MgCl_2$) and quench buffer (20 mM HEPES pH 7.4, 100 mM NaCl, 5 mM $MgCl_2$) were prepared, filtered, de-gassed, and re-gassed with Ar for 15 min on ice. Reaction setup: To each well of a 96-well PCR plate (reaction plate), 8 µL of 50% w/v TCEP-agarose beads in reaction buffer was added, followed by the addition of 92 µL of 10.87 µM protein or glutathione (GSH). In a separate 96-well PCR plate (ligand plate), 3 µL of DMSO or 50 mM ligand in DMSO was added to 147 µL reaction buffer and centrifuged (1k rpm, 5 min, 4 °C). 100 µL of ligand solution or DMSO control from the ligand plate was added to the reaction plate (final concentration: 5 µM protein/GSH and 500 µM ligand). After mixing, the TCEP-agarose beads were pelleted by centrifugation (1k rpm, 5 min, 4 °C) and the plate was kept at 4 °C. At a series of time points (t = 0.25. 1, 2, 4, 7, 24, 48, 72, and 96 h), a 3 µL aliquot in duplicate from the reaction plate was quenched in a black 384-well plate, in which each well was pre-filled with 27 µL of 7-Diethylamino-3-(4'-Maleimidylphenyl)-4-Methylcoumarin (CPM) solution (1.4 µM in quench buffer). The fluorescence plate was spun down (1k rpm, 1 min) and incubated for 60 min at room temperature and then fluorescence intensity (excitation/emission: 384/470 nm) was measured on an EnVision™ plate reader. Data analysis: All analyses were conducted using Prism software (Graphpad). Each fluorescence readout was normalized to the average of the DMSO controls. The normalized fluorescence was plotted against time. A one phase exponential decay was fitted to each plot (constraints: $Y(0) > 0.8$; $0 < \text{plateau} < 0.3$; $k > 0$).

Hit selection

Following the screen, we analyzed the obtained 3,840 kinetic curves to estimate k_{obs} values and used these to determine rate enhancement factors (REFs) by dividing the k_{obs} of each construct

against the k_{obs} for GSH. To further filter for any thiol intrinsic reactivity, REF values for each construct were normalized against the REF value obtained from the geometric mean of k_{obs} values for the specific construct. Compounds with a normalized REF>5 were considered to have significantly enhanced labelling rates for the specific construct. We further filtered the hitlist by excluding compounds that had showed REF>5 for other targets screened in previous campaigns. Overall, 127 acrylamides with enhanced labelling for a specific cysteine mutant RAB27A construct were selected for a validation screening in which we repeated the qIT assay against nRAB27A-Cys123 and nRAB27A-Cys188 at 500 µM (Figure 2A). Four compounds were selected for validation in dose-response analyses (500 µM, 250 µM, 125 µM, 62.5µM, 0 µM) against all thiols (fRAB27A-Cys123, fRAB27A-Cys188, nRAB27A-Cys123, nRAB27A-Cys188, and GSH). Due to best synthetic tractability, we selected hit compound **IMP-1704**.

Intact protein MS analyses

Assays were performed in Agilent InfinityLab 384-well plates where 15 µL of freshly prepared and filtered assay buffer (20 mM HEPES, 100 mM NaCl, 5 mM $MgCl_2$, pH 8.0) with 1 µM protein was mixed with 15 µL of the desired compound (20 µM) in filtered assay buffer + 4% DMSO, to give final assay concentrations of 500 nM protein, 10 µM compound, and 2% DMSO. The reaction plate was shaken (~300 rpm) at rt for 60 min, after which the reaction was quenched by the addition of 7.5 µL of 2% formic acid in MilliQ water. Samples were then submitted to LC-MS analysis on an AdvanceBio 6545XT LC/Q-TOF system (Agilent) fitted with an ACQUITY UPLC Protein BEH C4 Column, 300 Å, 1.7 µm, 2.1 mm x 50 mm (Waters) heated to 45 °C. Data was analysed and processed using BioConfirm (Agilent). The total ion chromatograms (TIC) were extracted (region containing protein) and the summed scans were deconvoluted (using MaxEnt). Protein labelling (%) was calculated by dividing the labelled protein intensity by the sum of the unlabelled and labelled protein intensities. Figures were made using GraphPad Prism 10.0.

Time (min)	A – H_2O+0.1% FA (%)	B – MeCN+0.1% FA (%)	Flow (mL/min)
0.00	80	20	0.6
0.20	80	20	0.6
1.40	35	65	0.6
1.70	5	95	0.6
1.90	5	95	0.6
2.10	80	20	0.6
2.30	80	20	0.6

Fluorescence polarization assay

The assay buffer (20 mM HEPES, 100 mM NaCl, 5 mM $MgCl_2$, pH 8.0) was freshly prepared and filtered (0.2 µm), after which 0.005% Tween was added. 2× Peptide-only buffer was prepared by adding 70 nM A10-TAMRA to the assay buffer. 2× Protein and peptide buffer was prepared by the addition of 70 nM A10-TAMRA and 400 nM nRAB27a-Cys123 to the assay buffer.
Compound plate setup: 2× Compound dilutions (seven-fold 1:3 dilution series in triplicate) in filtered assay buffer +4% DMSO were prepared in a 384-well microplate (Corning CLS4514).
Reaction plate setup: 5 µL of 2× peptide-only buffer or 2× protein and peptide buffer were added to a clear-bottom 384-well microplate (Greiner 788096—black, small volume, lobase, MED binding, µCLEAR). The reaction was started by the addition of 5 µL of 2× compound or 4% DMSO in assay buffer from the compound plate to the reaction plate using an Apricot S2 liquid handler. Final assay concentrations: 35 nM A10-TAMRA, 200 nM nRAB27a-Cys123, desired compound

concentrations (seven-fold 1:3 dilution series in triplicate), and 2% DMSO. Polarisation (mP) was measured every 10 min for 2 h using a Clariostar plate reader (filter set 1-TAMRA FP, ex. 540-20, em. 590-20, 20 flashes per well).
Data analysis was performed using Graphpad Prism software (Version 10.2.3). Raw mP values were normalised by subtracting the mean mP values of the peptide-only wells, followed by normalisation to the mean mP values of the DMSO-containing peptide and protein wells. IC_{50} values were calculated for 30 and 120 mins by plotting normalised data against concentration and fitting to the "[Inhibitor] vs. normalised response -- Variable slope" model using Prism software. The pseudo-first order rate constants (k_{obs}) values were calculated by plotting normalised data against time and fitting to the "one phase exponential association" model (Y=0, Plateau >50) using Prism software. The k_{obs} values with R^2 values > 0.6 were then plotted against concentration and fitted to a linear regression model (Y=0) to generate k_{inact}/K_I values.

Glutathione (GSH) reactivity assay

Compounds (50 µM), fluorescein (internal standard, 25 µM), and GSH (5 mM) or PBS (control) were incubated in PBS containing 10% DMSO at 35 °C. Samples were analysed at 0, 30, 60, 120, and 180 min by LC-MS using an Agilent AdvanceBio 6545XT LC/Q-TOF system equipped with a Poroshell 120 EC-C18 2.1 × 50 mm column (Agilent). Data were processed using MassHunter (Agilent). Peak areas for the parent compound were normalised to the fluorescein internal standard peak area at each time point and subsequently normalised to the 0 h time point. Figures were generated using GraphPad Prism 11.0.

Cell culture

MDA-MB-231 cells were cultivated in Dulbecco's Modified Eagle Medium (DMEM) [high glucose, pyruvate] supplemented with 10% (v/v) fetal bovine serum and grown at 37 °C under a humidified atmosphere supplied with 5% CO2. For all experiments, cells cultivated for ≤ 20 passages were used.

Cell death assay in MDA-MB-231

MDA-MB-231 cells were plated in a 96-well plate (Greiner; clear) at $5*10^3$ cells/well in 100 µL of 10% FBS in DMEM. After 16 h, 100 µL of medium containing a 2x final concentration of treatment were added to each well. Conditions tested were 6x 1:2 dilutions from 50 µM in biological triplicate (final DMSO 0.1%). Sytox green was added to medium to a final concentration of 250 nM. 2 µg/mL of puromycin were used to define 100% cell death. Quantification of green fluorescence area (488) over brightfield area was performed using the Incucyte® software. Data were blank corrected, normalized and plotted using Graphpad Prism.

SDS−PAGE, WB and antibodies.

12% SDS−PAGE gels were freshly prepared prior to use. Gels were run for 15−20 min at 80 V, then for 120 min at 120 V (running buffer: 35 mM Tris, 285 mM glycine, 0.6% (w/v) SDS in Milli-Qwater). The SDS-gel was blotted on a nitrocellulose membrane for 60 min at 100 V (blotting buffer: 25 mM Tris, 192 mM glycine, 20% methanol) using a Bio-Rad Mini Trans-Blot® Cell or Trans-Blot SD semidry transfer cell setup. The membrane was blocked in 5% milk powder with 0.1% Tween-20 TBS (50 mM Tris, 150 mM NaCl, pH 7.4), washed once with 0.1% Tween-20 TBS, and incubated with the specified antibody overnight at 4 °C. The blot was washed 4 × 5 min

with 0.1% Tween-20 TBS, incubated with secondary HRP antibody (rabbit or mouse, Advansta) for 1 h at rt, and washed 4 × 5 min with 0.1% Tween-20 TBS prior to HRP substrate addition and chemiluminescence detection.

Primary antibody	Target	Dilution	Buffer
RAB27A (D7Z9Q) Rabbit mAb #69295 (Cell Signalling)	RAB27A	1:1,000	3% BSA in 1% Tween-20 PBS
Anti-FLAG® (H7425) Rabbit (Aldrich)	FLAG-tag	1:500	1% Tween-20 PBS
Anti-Vinculin antibody [EPR8185] (Abcam)	Vinculin	1:10,000	5% milk in 1% Tween-20 PBS
Anti-GAPDH antibody [ab9485] (abcam)	GAPDH	1:5,000	3% BSA in 1% Tween-20 PBS
Anti-GFP antibody [ab1218] (abcam)	GFP-tag	1:3,000	3% BSA in 1% Tween-20 PBS

Click chemistry protocol

To every 100 µL of the protein sample (0.5–2 mg/mL) was added 6 µL of a mixture containing 1 mM $CuSO_4$, 1 mM tris(2-carboxymethylphosphine) (TCEP), 100 µM tris(1-benzyl-1H-1,2,3-triazol-4-yl)methylamine (TBTA), and 100 µM azide (containing TAMRA (AzT), biotin (AzB) or both (AzTB)), and then the mixture was incubated for 1 h at rt with shaking. The reaction was stopped by protein precipitation with 4 volumes of MeCN. The samples were centrifugated (5 min at 16,000 g) and the supernatant discarded. The obtained pellet was washed with 80% EtOH (3–5x) and sonication, followed by centrifugation (5 min at 16,000 g) and careful removal of the supernatant. Finally, the pellet was dried in a fume hood for 5 min and redissolved stepwise in 0.2% SDS in PBS (starting from 2% SDS in PBS) for further assays (e.g., pull-downs) or directly in 20% β-mercaptoethanol in Laemmli buffer, denatured for 5 min at 95 °C, and subjected to SDS–PAGE analysis.

Streptavidin shift assay

MDA-MB-231 cells were plated in a 12-well plate at 5 × 10^5 cells/well in 1 mL of 10% FBS in DMEM. After 24 h, the cells were treated with the specified compound concentration or DMSO for the specified time at 37 °C. After incubation, the medium was removed and the cells were washed with 2 × 1 mL PBS, lysed with 100 µL ice-cold lysis buffer (1% NP-40 in PBS, containing protease inhibitors [cOmplete™ Protease Inhibitor Cocktail] and benzonase), clarified by centrifugation and protein concentration adjusted to 0.8–1 mg/mL. Cell lysates (20–50 µg protein) were clicked according to the Click Chemistry protocol with AzTB or AzB including precipitation and washing. The dried samples were resuspended in 18–46 µL of 10% BME in Laemmli buffer 1x in PBS. Streptavidin stock solution (100 µM, Jackson Immunoresearch, 016-000-113) was thawed on ice. Each sample was added 2–4 µL Streptavidin (+ Streptavidin samples) or H_2O (– Streptavidin samples) and incubated 10 min at rt before running SDS-PAGE and western blotting according to standard protocol.

Pull-down experiments

MDA-MB-231 cells were plated in a 12-well plate at 5 × 10^5 cells/well in 1 mL of 10% FBS in DMEM. After 24 h, the cells were treated with the specified compound concentration or DMSO

for 2 h at 37 °C. After incubation, the medium was removed and the cells were washed with 2 × 1 mL PBS, lysed with 100 µL ice-cold lysis buffer (1% NP-40 in PBS, containing protease inhibitors [cOmplete™ Protease Inhibitor Cocktail] and benzonase), clarified by centrifugation and protein concentration adjusted to 0.8–1 mg/mL. Cell lysates (50–150 µg protein) were clicked according to the Click Chemistry protocol with AzB including precipitation and washing. The clicked insoluble samples were redissolved in 0.2% SDS in PBS and dincubated with magnetic streptavidin beads (NEB) in 1:3–10 ratios (beads to protein) for 2 h at room temperature. The beads were washed 5 × 0.2% SDS in PBS, then redissolved in 20% β-mercaptoethanol in Laemmli buffer and incubated for 10 min at 95 °C. The supernatant was collected and subjected to SDS–PAGE analysis followed by western blotting for RAB27A.

PPI inhibition

MDA-MB-231 cells were seeded in 6-well plates (6 × 10^5 cells/well) using standard cell culture conditions (2 mL of medium per well). After 24 h each well was transfected with 1.5 µg of purified GFP-Slp2-a plasmid (Addgene #40047) using Lipofectamine 3000 (Invitrogen) in opti-MEM™ medium, according to the manufacturer's instructions. After 6 h, the cells were treated with the specified compound concentration or 0.5% DMSO for 16 h at 37 °C. After incubation, the medium was removed and the cells were washed with 2 × 1 mL ice-cold PBS, lysed with 200 µL ice-cold lysis buffer (20 mM Tris-HCl, 50 mM NaCl, 1 mM EGTA, 1% CHAPS, 1 mM GppNHp, pH 7.5, containing protease inhibitors [cOmplete™ Protease Inhibitor Cocktail]), clarified by centrifugation and protein concentration adjusted to 1 mg/mL.

In lo-bind Eppendorf tubes, magnetic GFP-trap beads (10 µL / well) were washed with 3 × 1 mL ice-cold wash buffer (20 mM Tris-HCl, 50 mM NaCl, 1 mM EGTA, 1% CHAPS, pH 7.5) before the addition of 150 µL of normalized lysate. The samples were rotated for 1 h at 4 °C. Flowthrough was removed, and the magnetic GFP-trap beads were washed with 2 × 1 mL ice-cold wash buffer (20 mM Tris-HCl, 50 mM NaCl, 1 mM EGTA, 1% CHAPS, pH 7.5). Samples were eluted from the beads by the addition of 1× Laemmli buffer with 5% β-mercaptoethanol, and heating at 95 °C for 10 min, followed by centrifugation and collection of the supernatant. Samples were then subject to SDS–PAGE and WB analysis.

Overexpression experiments

MDA-MB-231 cells were seeded in a 12-well plate (5 × 10^5 cells/well) using standard cell culture conditions (1 mL of medium per well) and each well was transfected by addition of 50 µL of a preincubated solution containing 1 µg of the specified plasmid (Figure 6E and S10) and 3 µL of Fugene® HD in opti-MEM™ medium. After 18 h, the cells were treated with the specified probe concentration or DMSO for 24 h at 37 °C. Cells were lysed with 1% NP-40 in PBS containing protease inhibitors (cOmplete™ Protease Inhibitor Cocktail), clarified by centrifugation and protein concentration adjusted to 0.8–1 mg/mL. Samples were processed according to the specified protocol.

Whole proteome proteomics

MDA-MB-231 cells were seeded in a 12-well plate (3 × 10^5 cells/well) using standard cell culture conditions (1 mL of medium per well). After 24 h, the medium was removed and the cells were washed with 2 × 0.5 mL ice-cold PBS, lysed with 100 µL ice-cold lysis buffer (1% NP-40 in PBS containing protease inhibitors [cOmplete™ Protease Inhibitor Cocktail]), clarified by centrifugation and protein concentration adjusted to 1 mg/mL.

Reduction and alkylation of the proteins was carried out by treating with 10 mM tris(2-carboxyethyl)phosphine (TCEP, Sigma-Aldrich) and 40 mM chloroacetamide (CAA, Sigma-Aldrich) for 10 min at rt. Protein was then precipitated by the addition of 4 volumes of acetonitrile (VWR, HPLC grade). The sample was centrifuged, and the supernatant discarded. The pellet was then washed with 3 × 80% ethanol (VWR, HPLC grade). The protein pellet was then treated with trypsin (6.25 ng/µL, Pierce) in a 25 mM ammonium bicarbonate (SigmaAldrich) pH 8.0 buffer and agitated at 37 °C overnight. The samples were dried using a SpeedVac concentrator before being rehydrated in 2% acetonitrile and 0.5% TFA in water (Optima, LC-MS grade).

Activity of RAB27A/B inhibitors in melan-a melanocytes

Melan-a murine melanocytes were seeded into 96-well plates (Merck, UK) at $5x10^3$ cells/well and cultured in standard conditions for 24 h (RPMI 1640 medium supplemented with 10% fetal calf serum, 2 mM glutamine, 100 U/ml penicillin G, 100 mg/ml streptomycin, and 200 nM phorbol 12-myristate 13-acetate (all Sigma-Aldrich, Poole, UK) in a humidified incubator at 37 °C with 10% CO_2). Compounds, diluted in growth medium (final 0.3% DMSO), were added to cells, cultured as above for 24 h and the effects on melanosome dispersion recorded using a Zoe cell imager (Bio-rad UK 1450031). Cellular melanosome distribution was scored as either 'clustered' or 'dispersed', dependent upon whether melanosomes were distributed in the perinuclear cytoplasm or isotropically distributed throughout the cytoplasm as determined by visual inspection.

Viability assay in melan-a melanocytes

Melan-a mouse melanocytes were seeded into 96-well plates (Merck, UK) at 5×10^3 cells/well and cultured in standard conditions for 24 h (RPMI 1640 medium supplemented with 10% fetal calf serum, 2 mM glutamine, 100 U/ml penicillin G, 100 mg/ml streptomycin, and 200 nM phorbol 12-myristate 13-acetate (all Sigma-Aldrich, Poole, UK) in a humidified incubator at 37 °C with 10% CO_2). Compounds, diluted in growth medium, were added to cells, cultured as above for 24 h and the effects on viability were recorded using a Prestoblue assay (ThermoFisher A13262) according to the manufacturer protocol and using an ID3 microplate reader (Spectramax 412379).

Chemical proteomics

MDA-MB-231 cells were seeded in a 12-well plate (3×10^5 cells/well) using standard cell culture conditions (1 mL of medium per well). After 24 h, the cells were treated with the specified compound concentration or 0.5% DMSO for 2 h at 37 °C. For competition experiments, an additional treatment was performed with the specified compound concentration or 0.5% DMSO for 2 h at 37 °C. After incubation, the medium was removed and the cells were washed with 2 × 0.5 mL ice-cold PBS, lysed with 100 µL ice-cold lysis buffer (1% NP-40 in PBS containing protease inhibitors [cOmplete™ Protease Inhibitor Cocktail]), clarified by centrifugation and protein concentration adjusted to 0.8 mg/mL.

In a 96-deep well plate (KingFisher), 100 µL/well of normalised lysate was added, along with 6 µL/well of a mixture containing 1 mM $CuSO_4$, 1 mM tris(2-carboxymethylphosphine) (TCEP), 100 µM tris(1-benzyl-1H-1,2,3-triazol-4-yl)methylamine (TBTA), and 100 µM azide-PEG_3-biotin (AzB). The mixture incubated for 1 h at rt with shaking, before being quenched by the addition of 1 µL EDTA (500 mM) to a final concentration of 5 mM.

To each well, 30 µL of a freshly prepared 1:1 mixture of Cytiva magnetic E3 and E7 SpeedBeads were added, followed by the addition of 591 µL of EtOH and incubation at rt for 15 min with shaking. The plate was then transferred to a Thermo KingFisher system where beads were

washed twice with 90% EtOH (500 µL) before the protein being eluted in 0.2% SDS in 50 mM HEPES, pH 8.0 (150 µL). Pierce Streptavidin magnetic beads (10 µL/well) were added and incubated for 1 h at rt with shaking. The plate was then transferred to a Thermo KingFisher system where beads were washed with RIPA buffer (500 µL), and 1% SDS in 50 mM HEPES pH 8.0 (500 µL). Proteins were then reduced and alkylated using 10 mM TCEP and 40 mM chloroacetamide in 0.2% SDS and 50 mM HEPES pH 8.0 (500 µL), after which the beads were washed twice with 50 mM HEPES pH 8.0 (500 µL), and eluted in 50 mM HEPES pH 8.0 (200 µL). To each well, 0.2 µg of trypsin (Pierce) was added and samples were agitated at 37 °C overnight. Samples were then filtered, acidified to below pH 3 using formic acid, and frozen at -20 °C.

LC-MS/MS analysis

Peptides for proteomics analysis were loaded onto Evotips (Evosep) as per the manufacturer's instructions. For whole proteome analysis, 200 ng peptide was loaded, while for pulldown sample, half of the sample collected was loaded for analysis.

For whole proteome experiments:

Peptides were analysed by nanoLC-MS/MS using an Evosep One (Evosep) coupled with a timsTOF HT (Bruker) equipped with an 8 cm × 150 µm, 1.5 µm analytical column (Evosep). 200 ng peptides were separated by the Evosep 60SPD workflow (Analytical solvents A: 0.1% FA and B: acetonitrile 17 plus 0.1% FA). Column was held at 40 °C. Data were acquired in data-independent acquisition (DIA) PASEF mode with the following settings: *m/z* range from 100 *m/z* to 1700 *m/z*, ion mobility range from 1/K0 = 1.30 to 0.85 Vs/cm^2 using equal ion accumulation and ramp times in the dual TIMS analyser of 100 ms each. Each cycle consisted of 8 PASEF ramps covering 21 mass steps each with 25 Da windows each with 2/3 non-overlapping ion mobility windows covering the 475 to 1000 *m/z* range and 0.85 and 1.26 Vs/cm2 ion mobility range. The collision energy was lowered as a function of increasing ion mobility from 59 eV at 1/K0 = 1.6 Vs/cm^2 to 20 eV at 1/K0 = 0.6 Vs/cm^2.

For pulldown experiments:

Peptides were analysed by nanoLC-MS/MS using an Evosep One (Evosep) coupled with a timsTOF HT (Bruker) equipped with an 8 cm × 150 µm, 1.5 µm analytical column (Evosep). 200 ng peptides were separated by the Evosep 60SPD workflow (Analytical solvents A: 0.1% FA and B: acetonitrile plus 0.1% FA). Column was held at 40 °C. Data were acquired in data-dependent acquisition (DDA) PASEF mode with the following settings: *m/z* range from 100 *m/z* to 1700 *m/z*, ion mobility range from 1/K0 = 1.30 to 0.85 Vs/cm^2 using equal ion accumulation and ramp times in the dual TIMS analyzer of 100 ms each. The collision energy was lowered as a function of increasing ion mobility from 59 eV at 1/K0 = 1.6 Vs/cm^2 to 20 eV at 1/K0 = 0.6 Vs/cm^2. Isolation width was lowered as a function of decreasing m/s from 3 *m/z* at 800 *m/z* to 2 *m/z* at 700 *m/z*. Active exclusion was applied for 0.4 min. Each cycle consisted of 4 PASEF ramps (total cycle time 0.53 s) with 2.75 ms measuring time allowed for each selected precursor.

Data processing and downstream analysis

For whole proteome experiments:

diaPASEF Bruker.d files were processed using library-free analysis in DIA-NN (version 2.3.1) using the following parameters: Human database (Downloaded from UniProt 24 February 2026

containing 20518 proteins and 125 common contaminants); “deep learning-based spectra and RTs prediction” was enabled; trypsin with 1 missed cleavages; N-term Excision, C carbamidomethylation, Oxidation and N terminal Acetylation were enabled with maximum 2 variable modifications; MBR was enabled; quantification strategy set to “QuantUMS (precision)”; protein inference was enabled; Mass and MS1 accuracy set to 0.
Data analysis was carried out using Perseus version 2.1.5.0. Intensities were loaded for analysis. A text filter was applied to remove contaminants and data was Log_{10} transformed. Rows were annotated to group replicates, and data was filtered for valid values in all replicates. Protein intensities were averaged and S-plots generated in GraphPad Prism version 11.
Proteomics data have been deposited on **PRIDE: PXD077793**.

For pulldown experiments:

ddaPASEF Bruker.d files were processed using Fragpipe version 23.0 (Nesvilab). Data was searched against a human reference proteome with isoforms (Uniprot, UP000005640, accessed 23 February 2026, 20464 proteins) with 50% decoys and contaminants added. The built-in label-free quantification-match between runs (LFQ-MBR) workflow was used. Bruker .d files were searched using MSFragger (version 4.4.1) The following parameters were used: strict trypsin digestion; a maximum of 2 missed cleavages allowed; precursor ion tolerance of 20 ppm; oxidation (M) and N terminal acetylation as variable modifications; carbamidomethylation (C) as a fixed modification. MaxLFQ minimum ions was set to 1 and the retention time tolerance for match between runs was set to 1 min. All other default parameters were used for processing. MSFragger search results were processed using Percolator (version 3.7.1) for peptide-spectrum match validation, followed by Philosopher (version 5.1.1) for protein and FDR 18 filtering. Label-free quantification values were calculated using the MaxLFQ algorithm using IonQuant (version 1.11.20) with match between runs enabled and min ions set as 1.
Data analysis was carried out using Perseus version 2.1.5.0. MaxLFQ intensities (competition volcano plots) or iBAQ intensities (target enrichment S-plots) were loaded for analysis. For competition volcano plots, rows were filtered based on combined total peptide >1, a text filter was applied to remove contaminants and data was Log_2 transformed. Rows were annotated to group replicates, and data was filtered for 4/4 valid values in both conditions. Data was normalized by subtracting the median from the columns. Volcano plots were generated by plotting the fold-changes of the protein MaxLFQ intensities against the $-Log_{10}$(p-value). These were calculated by carrying out a two-sample t-test of the intensity values for each protein in the two conditions (permutation-based FDR = 0.05, S0 = 0.1). Data were plotted in GraphPad Prism version 11. For target enrichment S-plots rows were filtered based on combined total peptide >1, a text filter was applied to remove contaminants and data was Log_2 transformed. Rows were annotated to group replicates, and data was filtered for 4/4 valid values at least one condition. Proteins not significantly enriched (fold change >1, $-Log_{10}$(p-value) >1.3) in probe treated samples were removed. Enriched protein iBAQ intensities were averaged and S-plots generated in GraphPad Prism version 11.
Proteomics data have been deposited on **PRIDE: PXD077741**.

File name	Experiment	Inhibitor	Probe
Comp7-1_AMT-029_C57_20_1_reprep_S5-C9_1_17813	AMT-029_Reprep	7 (20 µM)	9 (10 µM)
Comp7-2_AMT-029_C57_20_2_reprep_S5-C10_1_17814	AMT-029_Reprep	7 (20 µM)	9 (10 µM)
Comp7-3_AMT-029_C57_20_3_reprep_S5-C11_1_17815	AMT-029_Reprep	7 (20 µM)	9 (10 µM)
Comp7-4_AMT-029_C57_20_4_reprep_S5-C12_1_17816	AMT-029_Reprep	7 (20 µM)	9 (10 µM)
Comp8-1_AMT-029_C143_20_1_reprep_S5-D1_1_17818	AMT-029_Reprep	8 (20 µM)	9 (10 µM)
Comp8-2_AMT-029_C143_20_2_reprep_S5-D2_1_17819	AMT-029_Reprep	8 (20 µM)	9 (10 µM)
Comp8-3_AMT-029_C143_20_3_reprep_S5-D3_1_17820	AMT-029_Reprep	8 (20 µM)	9 (10 µM)
Comp8-4_AMT-029_C143_20_4_reprep_S5-D4_1_17821	AMT-029_Reprep	8 (20 µM)	9 (10 µM)
Comp9-1_AMT-029_C60_10_1_reprep_S5-C5_1_17808	AMT-029_Reprep	N/A	9 (10 µM)
Comp9-2_AMT-029_C60_10_2_reprep_S5-C6_1_17809	AMT-029_Reprep	N/A	9 (10 µM)
Comp9-3_AMT-029_C60_10_3_reprep_S5-C7_1_17810	AMT-029_Reprep	N/A	9 (10 µM)
Comp9-4_AMT-029_C60_10_4_reprep_S5-C8_1_17811	AMT-029_Reprep	N/A	9 (10 µM)
DMSO-1_AMT-029_DMSO_1_reprep_S5-A1_1_17772	AMT-029_Reprep	N/A	N/A
DMSO-2_AMT-029_DMSO_2_reprep_S5-A2_1_17773	AMT-029_Reprep	N/A	N/A
DMSO-3_AMT-029_DMSO_3_reprep_S5-A3_1_17774	AMT-029_Reprep	N/A	N/A
DMSO-4_AMT-029_DMSO_4_reprep_S5-A4_1_17775	AMT-029_Reprep	N/A	N/A
IMP2552-1_AMT-029_C109_10_1_reprep_S5-A9_1_17782	AMT-029_Reprep	IMP2552 / 10 (10 µM)	IMP2566 / 12 (5 µM)
IMP2552-2_AMT-029_C109_10_2_reprep_S5-A10_1_17783	AMT-029_Reprep	IMP2552 / 10 (10 µM)	IMP2566 / 12 (5 µM)
IMP2552-3_AMT-029_C109_10_3_reprep_S5-A11_1_17784	AMT-029_Reprep	IMP2552 / 10 (10 µM)	IMP2566 / 12 (5 µM)
IMP2552-4_AMT-029_C109_10_4_reprep_S5-A12_1_17785	AMT-029_Reprep	IMP2552 / 10 (10 µM)	IMP2566 / 12 (5 µM)
IMP2566-1_AMT-029_C123_5_1_reprep_S5-A5_1_17777	AMT-029_Reprep	N/A	IMP2566 / 12 (5 µM)
IMP2566-2_AMT-029_C123_5_2_reprep_S5-A6_1_17778	AMT-029_Reprep	N/A	IMP2566 / 12 (5 µM)
IMP2566-3_AMT-029_C123_5_3_reprep_S5-A7_1_17779	AMT-029_Reprep	N/A	IMP2566 / 12 (5 µM)
IMP2566-4_AMT-029_C123_5_4_reprep_S5-A8_1_17780	AMT-029_Reprep	N/A	IMP2566 / 12 (5 µM)
IMP2958-1_AMT-029_C144_10_1_reprep_S5-B1_1_17787	AMT-029_Reprep	IMP2958 / 11 (10 µM)	IMP2566 / 12 (5 µM)
IMP2958-2_AMT-029_C144_10_2_reprep_S5-B2_1_17788	AMT-029_Reprep	IMP2958 / 11 (10 µM)	IMP2566 / 12 (5 µM)
IMP2958-3_AMT-029_C144_10_3_reprep_S5-B3_1_17789	AMT-029_Reprep	IMP2958 / 11 (10 µM)	IMP2566 / 12 (5 µM)
IMP2958-4_AMT-029_C144_10_4_reprep_S5-B4_1_17790	AMT-029_Reprep	IMP2958 / 11 (10 µM)	IMP2566 / 12 (5 µM)

General chemistry experimental

Reagents and solvents were obtained from Acros Organics, Alfa Aesar, Fisher Scientific, Fluorochem, Sigma-Aldrich, Tokyo Chemical Industry, or VWR International Ltd. And were used without further purification. The purity of all biologically tested compounds was ≥94% as determined by analytical HPLC/LC-MS and/or NMR.

Analytical thin layer chromatography (TLC) was carried out using Merck silica gel 60 F254 aluminium-supported thin layer chromatography sheets. Visualization was by absorption of UV light (λmax 254 nm), or thermal development after staining in either an: aqueous basic solution of potassium permanganate, or ethanolic solution of ninhydrin.

Flash column chromatography was carried out using Merck silica gel (40–63 µm), eluting using the indicated solvents under a positive pressure of N_2 gas.

Nuclear magnetic resonance (NMR) spectra were recorded using a 400 MHz Bruker spectrometer (^{1}H NMR 400 MHz, ^{13}C NMR at 101 MHz) and processed using MestReNova 12.0. Chemical shifts (δ) for ^{1}H NMR spectra are reported in parts per million (ppm) relative to the stated solvent as a reference for internal deuterium lock. The spectra are calibrated to residual solvent. Chemical shifts (δ) for ^{13}C NMR spectra are reported in parts per million (ppm) relative to the stated solvent as a reference for internal deuterium lock. The spectra are calibrated to residual solvent. The following abbreviations are used for multiplicities: s─singlet, d─doublet, t─triplet, q─quartet, qi─quintet, m─multiplet, dd─doublet of doublets, br─broad. Coupling constants (*J*) are reported in Hz. All spectra were run at 25 °C unless otherwise stated.

High-resolution mass spectra (HRMS) were recorded on a Micromass Autospec Premier through Imperial College London mass spectrometry services. Mass-to-charge ratios (*m*/*z*) were reported in Daltons, and HRMS was reported with less than 5 ppm error, or analytical HPLC analysis was carried out on an Alliance HPLC 2695 system (Waters, Ireland), equipped with an autosampler and photodiode array detector 2996 (Waters, Ireland). Data acquisition and processing was carried out on MassLynx 4.1 software.

High performance liquid chromatography (HPLC) / Liquid chromatography mass spectroscopy (LC-MS) was carried out according to the below methods.

Method A: Analysis was carried out on an Agilent 1260 Infinity II MSD/XT Single Quad system fitted with a Poroshell 120 EC-C18 2.1 × 50 mm column with a flow rate of 0.6 mL/min. The solvent gradient started at 5% MeCN in water with 0.1% formic, ending at 95% MeCN after 5 min.

Method B: Analysis was carried out on an Agilent 1260 Infinity II MSD/XT Single Quad system fitted with a Poroshell HPH-C18 3.0 × 50 mm column with a flow rate of 0.8 mL/min. The solvent gradient started at 5% MeCN in water with 0.1% formic, ending at 90% MeCN after 7 min.

Synthetic Procedures and Compound Data

General procedure A – Sulfonamide formation

To a solution of the specified amine in CH_2Cl_2 or THF (1 eq., 0.1–0.5 M) cooled to 0 °C, were added DIPEA or Et_3N (2–3 eq.) and the specified sulfonyl chloride (1 eq.). The reaction was allowed to warm to rt and quenched with H_2O after 1–16 h. The aqueous layer was extracted with ethyl acetate (3x, 20–100 mL), washed with brine (20–100 mL), dried ($MgSO_4$), filtered and concentrated *in vacuo*. The crude product was used without further purification unless specified.

General procedure B – Hydrogenation

To a stirring solution of the nitrosulfonamide in EtOAc and/or MeOH (1 eq, 0.05–0.2 M) was added 10% Pd/C (10% w/w). The flask was evacuated and refilled with a H_2 balloon (1 atm) three times and left stirring at rt for 16–72 h. The reaction mixture was filtered through celite with MeOH and the filtrate concentrated *in vacuo*. The crude product was used without further purification unless specified.

General procedure C – Acryloylation

To a solution of the specified aniline in anhydrous CH_2Cl_2 or THF (1 eq., 0.1–0.5 M) cooled to 0 °C, were added Et_3N (1.5–3 eq.) and acryloyl chloride (1.1–3 eq.). The reaction was allowed to warm to rt and quenched with H_2O after 1–16 h. The aqueous layer was extracted with ethyl acetate (3x, 20–100 mL), washed with brine (20–100 mL), dried ($MgSO_4$), filtered and concentrated *in vacuo*. The crude product was purified by liquid chromatography as specified.

General procedure D – Saponification

To a stirring solution of the specified ester in dioxane (1 eq., 0.1–0.5 M) was added a solution of LiOH (1.1–3.0 eq) in H_2O (0.1–0.5 M). The reaction was stirred at rt for 1–16 h, then diluted with H_2O and acidified with 1 M HCl until pH~3. The aqueous layer was extracted with EtOAc (3x, 20–100 mL), dried ($MgSO_4$), filtered and concentrated *in vacuo*. The crude product was used without further purification.

General procedure E – Amide coupling

To a stirring solution of the specified acid in CH_2Cl_2 or DMF (1 eq., 0.1–0.5 M) was added EDCI or HATU or HBTU (1.0–1.5 eq), DIPEA (3.0–4 eq) and the specified amine (1.0–1.5 eq). The reaction was stirred at rt for 1–16 h, then diluted with water (10–100 mL) and extracted with ethyl acetate (3x, 20–100 mL), washed with brine (20–100 mL), dried ($MgSO_4$), filtered and concentrated *in vacuo*. The crude product was purified by liquid chromatography as specified. In the case of parallel syntheses, the reactions were carried out in DMF and directly purified by reverse phase chromatography (5–100% MeCN in H_2O with 0.1% FA) to afford corresponding amides.

1-((4-Nitrophenyl)sulfonyl)pyrrolidine (15)

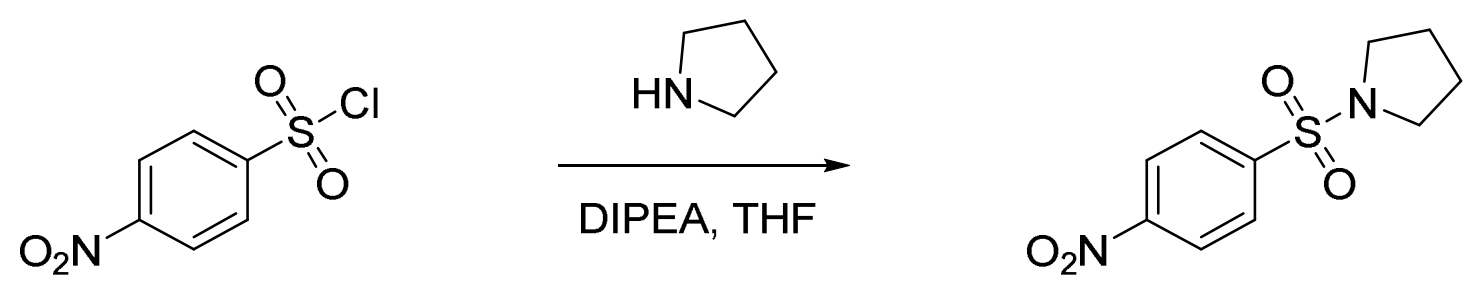

General procedure A, reagents: pyrrolidine (120 µL, 1.4 mmol); 4-nitrophenylsulfonyl chloride (300 mg, 1.4 mmol); DIPEA (470 µL, 2.7 mmol); THF (3.0 mL).
Yield: 350 mg, quantitative, yellow solid.
^{1}H NMR (400 MHz, DMSO-d6) δ: 8.50 – 8.36 (m, 2H), 8.19 – 7.99 (m, 2H), 3.26 – 3.12 (m, 4H), 1.70 – 1.64 (m, 4H) ppm.
^{13}C NMR (101 MHz, DMSO-*d*$_6$) δ: 150.4, 142.3, 129.3, 125.1, 48.4, 25.3 ppm.
LRMS (+ESI) *m/z*: $[M+H]^+$ 257.

4-(Pyrrolidin-1-ylsulfonyl)aniline (16)

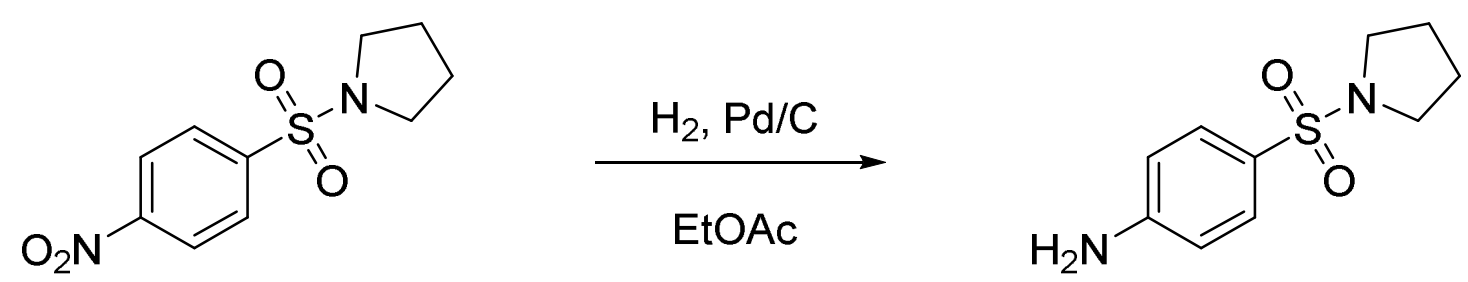

General procedure B, reagents: nitrosulfonamide **15** (150 mg, 0.58 mmol); H_2 (1 atm); 10% Pd/C (18 mg, 10% w/w); EtOAc (4.4 mL).
Yield: 110 mg, 85%, white solid.
^{1}H NMR (400 MHz, Chloroform-*d*) δ: 7.66 – 7.58 (m, 2H), 6.77 – 6.64 (m, 2H), 4.16 (s, 2H), 3.25 – 3.17 (m, 4H), 1.81 – 1.72 (m, 4H) ppm.
^{13}C NMR (101 MHz, Chloroform-*d*) δ: 150.5, 129.7, 125.1, 114.0, 47.9, 25.2 ppm.
LRMS (+ESI) *m/z*: $[M+H]^+$ 227.

***N*-(4-(Pyrrolidin-1-ylsulfonyl)phenyl)acrylamide (1, IMP-1704)**

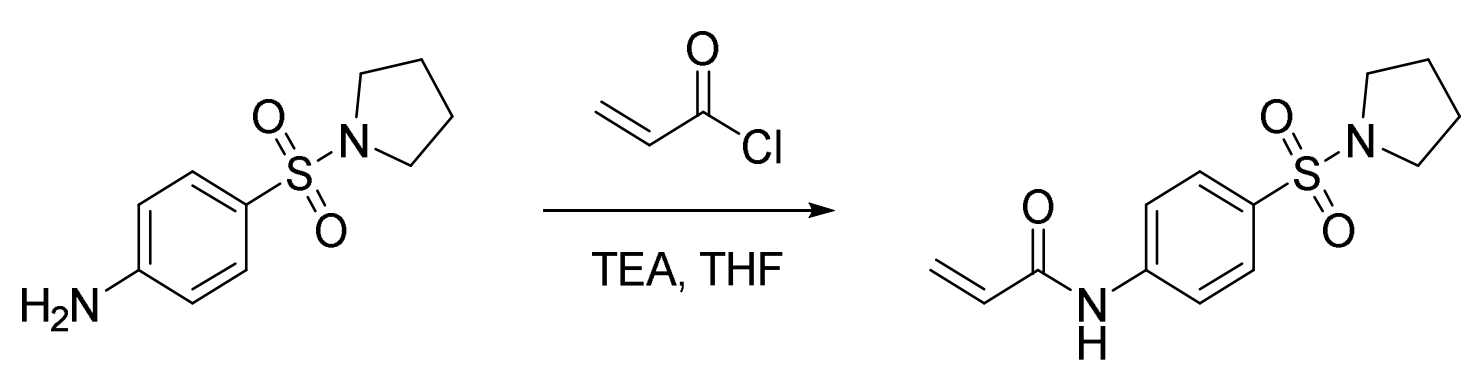

General procedure C, reagents: aniline **16** (100 mg, 0.44 mmol); acryloyl chloride (36 µL, 0.44 mmol); Et_3N (90 µL, 0.65 mmol); THF (3.0 mL); *purification:* LC (2x, 0–5% MeOH in CH_2Cl_2).
Yield: 50 mg, 41%, white solid.
^{1}H NMR (400 MHz, DMSO-*d*$_6$) δ: 10.57 (s, 1H), 7.96 – 7.86 (m, 2H), 7.78 (m, 2H), 6.47 (dd, *J* = 16.9, 10.0 Hz, 1H), 6.32 (dd, *J* = 17.0, 2.0 Hz, 1H), 5.84 (dd, *J* = 10.0, 2.0 Hz, 1H), 3.16 – 3.08 (m, 4H), 1.70 – 1.59 (m, 4H) ppm.
^{13}C NMR (101 MHz, DMSO-*d*$_6$) δ: 164.2, 143.5, 131.9, 130.7, 129.1, 128.6, 119.6, 48.3, 25.1 ppm.
LRMS (+ESI) *m/z*: $[M+H]^+$ 281.
HRMS (ESI) *m/z*: $[M+H]^+$ calcd for $C_{13}H_{17}N_2O_3S$: 281.0960; found: 281.0966.
HPLC retention time 4.0 min, >99.0% (Method A).

(4-Nitrophenyl)(pyrrolidin-1-yl)methanone (17)

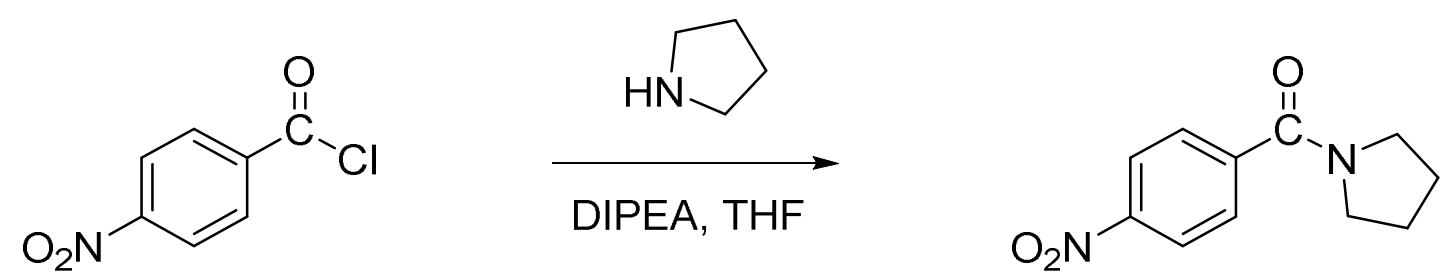

General procedure A, reagents: pyrrolidine (140 µL, 1.6 mmol); 4-nitrobenzoyl chloride (300 mg, 1.4 mmol); DIPEA (560 µL, 3.2 mmol); THF (3.0 mL).
Yield: 340 mg, 95%, orange solid.
^{1}H NMR (400 MHz, Chloroform-*d*) δ: 8.36 – 8.23 (m, 2H), 7.74 – 7.64 (m, 2H), 3.69 (t, J = 6.9 Hz, 2H), 3.40 (t, J = 6.6 Hz, 2H), 2.08 – 1.86 (m, 5H) ppm.
^{13}C NMR (101 MHz, Chloroform-*d*) δ 167.4, 148.4, 143.2, 128.2, 123.7, 49.5, 46.4, 26.4, 24.4 ppm.

(4-Aminophenyl)(pyrrolidin-1-yl)methanone (18)

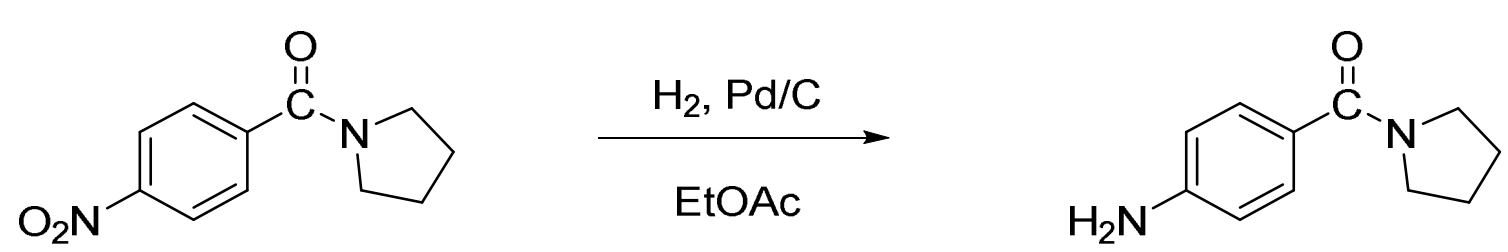

General procedure B, reagents: nitrobenzamide **17** (330 mg, 1.5 mmol); H_2 (1 atm); 10% Pd/C (30 mg, 10% w/w); EtOAc (3.0 mL).
Yield: 290 mg, quantitative, light orange solid.
^{1}H NMR (400 MHz, DMSO-*d*$_6$) δ: 7.32 – 7.20 (m, 2H), 6.59 – 6.46 (m, 2H), 5.51 (s, 2H), 3.43 (t, J = 6.0 Hz, 4H), 1.80 (t, J = 5.5 Hz, 4H) ppm.
^{13}C NMR (101 MHz, DMSO-*d*$_6$) δ: 169.0, 151.0, 129.6, 123.9, 112.9, 49.7, 46.6, 29.4, 24.4 ppm.

***N*-(4-(Pyrrolidine-1-carbonyl)phenyl)acrylamide (2)**

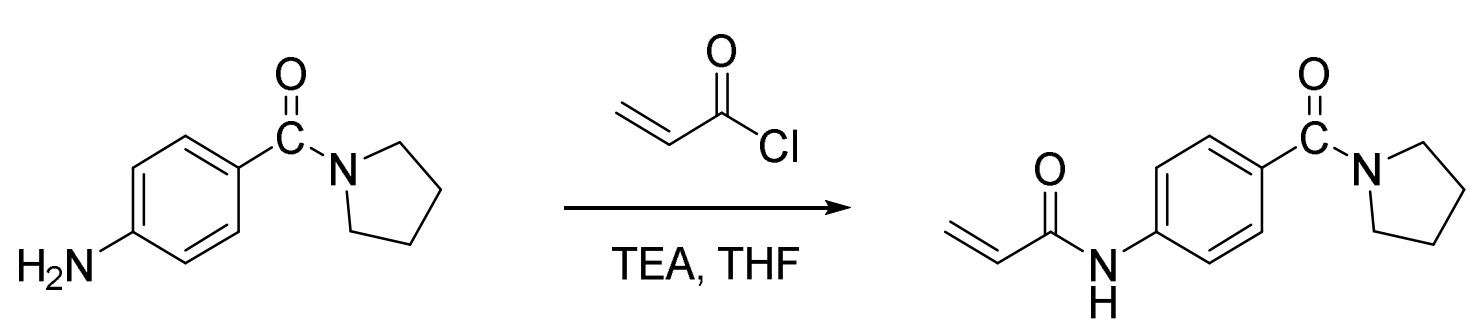

General procedure C, reagents: aniline **18** (100 mg, 0.53 mmol); acryloyl chloride (65 µL, 0.79 mmol); Et_3N (110 µL, 0.79 mmol); THF (1.5 mL); DMF (0.5 mL); *purification:* LC (0–10% MeOH in CH_2Cl_2).
Yield: 54 mg, 42%, white solid.
^{1}H NMR (400 MHz, DMSO-*d*$_6$) δ: 10.33 (s, 1H), 7.77 – 7.67 (m, 2H), 7.57 – 7.46 (m, 2H), 6.45 (dd, J = 17.0, 10.1 Hz, 1H), 6.29 (dd, J = 17.0, 2.1 Hz, 1H), 5.79 (dd, J = 10.1, 2.0 Hz, 1H), 3.43 (dd, J = 10.5, 4.7 Hz, 4H), 1.94 – 1.68 (m, 4H) ppm.
^{13}C NMR (101 MHz, DMSO-*d*$_6$) δ: 168.2, 163.8, 140.8, 132.4, 132.2, 128.6, 127.8, 119.0, 49.5, 46.5, 26.5, 24.4 ppm.
LRMS (+ESI) *m/z*: $[M+H]^+$ 245.
HRMS (ESI) *m/z*: $[M+H]^+$ calcd for $C_{14}H_{17}N_2O_2$: 245.1290; found: 245.1283.
HPLC retention time 3.2 min, >99.0% (Method A).

1-((3-Nitrophenyl)sulfonyl)pyrrolidine (19)

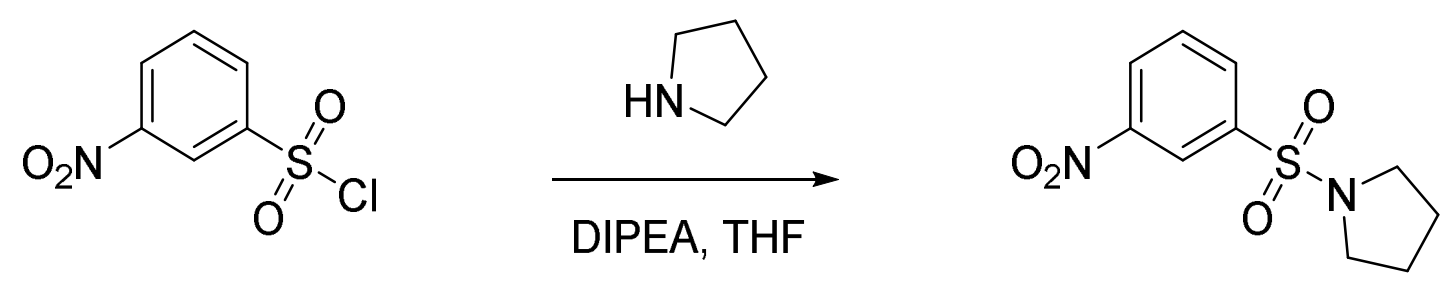

General procedure A, reagents: pyrrolidine (110 µL, 1.4 mmol); 3-nitrophenylsulfonyl chloride (300 mg, 1.4 mmol); DIPEA (470 µL, 2.7 mmol); THF (3.0 mL).
Yield: 260 mg, 75%, pale orange solid.
^{1}H NMR (400 MHz, DMSO-d_6) δ: 8.54 (ddd, *J* = 8.3, 2.3, 1.0 Hz, 1H), 8.42 (t, *J* = 2.0 Hz, 1H), 8.26 (ddd, *J* = 7.8, 1.8, 1.0 Hz, 1H), 7.94 (t, *J* = 8.0 Hz, 1H), 3.24 – 3.15 (m, 4H), 1.74 – 1.61 (m, 4H) ppm.
^{13}C NMR (101 MHz, DMSO-d_6) δ: 148.5, 138.4, 133.6, 132.0, 128.0, 122.4, 48.4, 25.3 ppm.
LRMS (+ESI) *m/z*: $[M+H]^+$ 257.

3-(Pyrrolidin-1-ylsulfonyl)aniline (20)

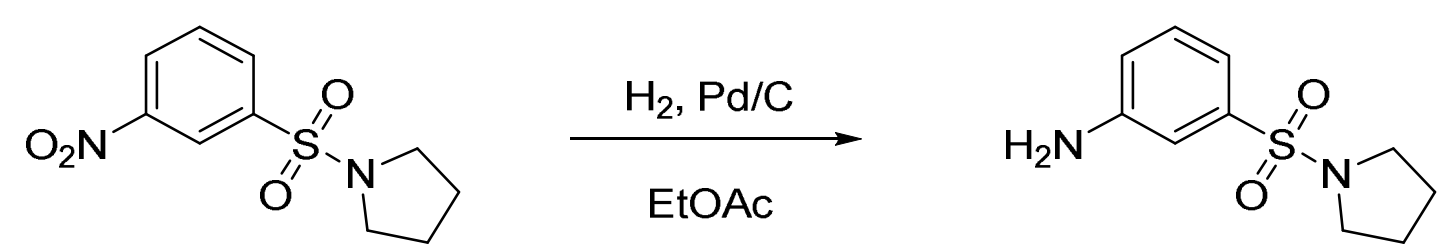

General procedure B, reagents: nitrosulfonamide **20** (250 mg, 0.98 mmol); H_2 (1 atm); 10% Pd/C (25 mg, 10% w/w); EtOAc (6 mL).
Yield: 220 mg, 99%, white solid.
^{1}H NMR (400 MHz, DMSO-d_6) δ: 7.23 (t, *J* = 7.9 Hz, 1H), 6.98 (t, *J* = 2.0 Hz, 1H), 6.85 (ddd, *J* = 7.7, 1.9, 0.9 Hz, 1H), 6.80 (ddd, *J* = 8.2, 2.3, 1.0 Hz, 1H), 3.19 – 3.05 (m, 4H), 1.70 – 1.61 (m, 4H) ppm.
^{13}C NMR (101 MHz, DMSO-d_6) δ 150.0, 136.9, 130.1, 118.2, 114.4, 112.2, 48.2, 25.2 ppm.

***N*-(3-(Pyrrolidin-1-ylsulfonyl)phenyl)acrylamide (3)**

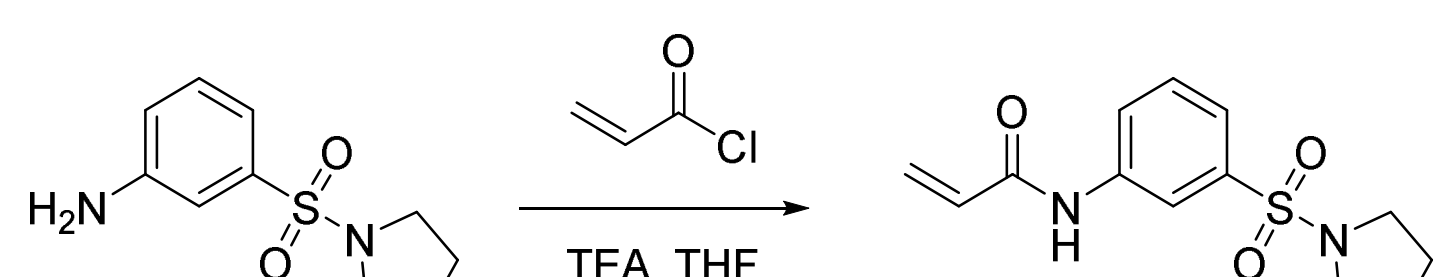

General procedure C, reagents: aniline **20** (100 mg, 0.44 mmol); acryloyl chloride (50 µL, 0.65 mmol); Et_3N (90 µL, 0.65 mmol); THF (1.5 mL).
Yield: 110 mg, 91%, white solid.
^{1}H NMR (400 MHz, DMSO-d_6) δ: 10.51 (s, 1H), 8.22 (t, *J* = 1.9 Hz, 1H), 7.92 (ddd, *J* = 8.2, 2.1, 1.0 Hz, 1H), 7.59 (t, *J* = 8.0 Hz, 1H), 7.49 (ddd, *J* = 7.8, 1.8, 1.0 Hz, 1H), 6.43 (dd, *J* = 17.0, 9.9 Hz, 1H), 6.31 (dd, *J* = 16.9, 2.1 Hz, 1H), 5.82 (dd, *J* = 10.0, 2.1 Hz, 1H), 3.23 – 3.08 (m, 4H), 1.78 – 1.53 (m, 4H) ppm.
^{13}C NMR (101 MHz, DMSO-d_6) δ: 164.1, 140.2, 137.1, 131.9, 130.5, 128.3, 123.6, 122.4, 118.1, 48.3, 25.2 ppm.
LRMS (+ESI) *m/z*: $[M+H]^+$ 281.
HRMS (−ESI) *m/z*: $[M-H]^-$ calcd for $C_{13}H_{15}N_2O_3S$: 279.0803; found: 279.0812.
HPLC retention time 3.6 min, 98.2% (Method A).

Methyl ((4-nitrophenyl)sulfonyl)-*L*-prolinate (21)

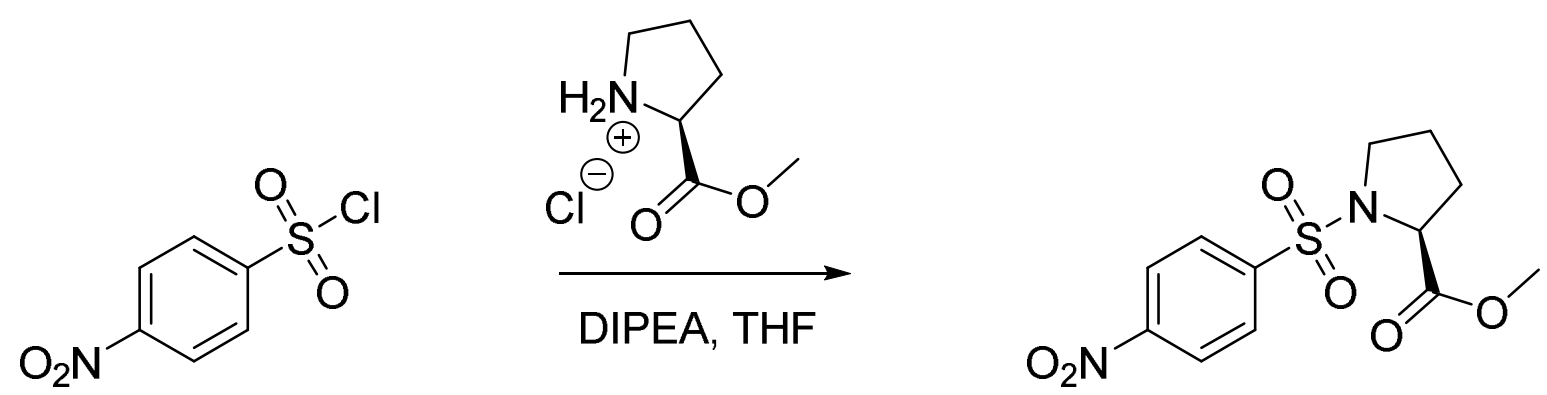

General procedure A, reagents: methyl L-prolinate hydrochloride (750 mg, 4.5 mmol); 4-nitrophenylsulfonyl chloride (1.0 g, 4.5 mmol); DIPEA (2.4 mL, 14 mmol); THF (5.0 mL).
Yield: 1.4 g, 97%, orange oil.
^{1}H NMR (400 MHz, Chloroform-*d*) δ: 8.42 – 8.37 (m, 2H), 8.15 – 8.07 (m, 2H), 4.48 (dd, *J* = 8.5, 3.7 Hz, 1H), 3.73 (s, 3H), 3.47 (dd, *J* = 7.4, 6.0 Hz, 2H), 2.26 – 2.13 (m, 1H), 2.12 – 1.87 (m, 3H) ppm.
^{13}C NMR (101 MHz, Chloroform-*d*) δ: 172.2, 144.7, 128.7, 124.2, 60.6, 52.6, 48.3, 31.0, 24.8 ppm.
LRMS (+ESI) *m/z*: [M+Na]$^+$ 337.

Methyl ((4-aminophenyl)sulfonyl)-*L*-prolinate (22)

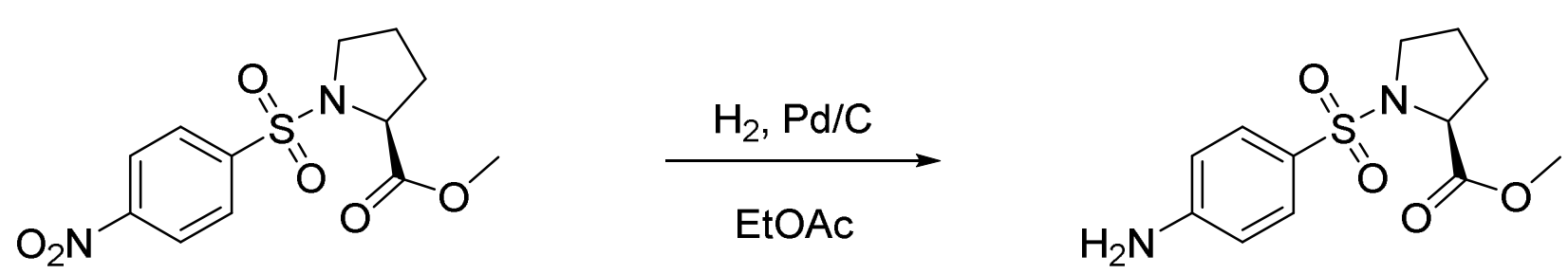

General procedure B, reagents: nitrosulfonamide **21** (1.4 g, 4.3 mmol); H_2 (1 atm); 10% Pd/C (140 mg, 10% w/w); EtOAc (6 mL).
Yield: 1.1 g, 85%, light pink solid.
^{1}H NMR (400 MHz, DMSO-*d*$_6$) δ: 7.48 – 7.40 (m, 2H), 6.69 – 6.57 (m, 2H), 6.09 (s, 2H), 4.05 (dd, *J* = 8.5, 4.4 Hz, 1H), 3.65 (s, 3H), 3.34 – 3.26 (m, 1H), 3.09 (m, 1H), 2.08 – 1.72 (m, 3H), 1.67 – 1.46 (m, 1H) ppm.
^{13}C NMR (101 MHz, DMSO-*d*$_6$) δ: 172.9, 153.7, 129.7, 122.0, 113.3, 60.6, 52.5, 48.9, 30.8, 24.7 ppm.
LRMS (+ESI) *m/z*: [M+H]$^+$ 285.

Methyl ((4-acrylamidophenyl)sulfonyl)-*L*-prolinate (4, IMP1712)

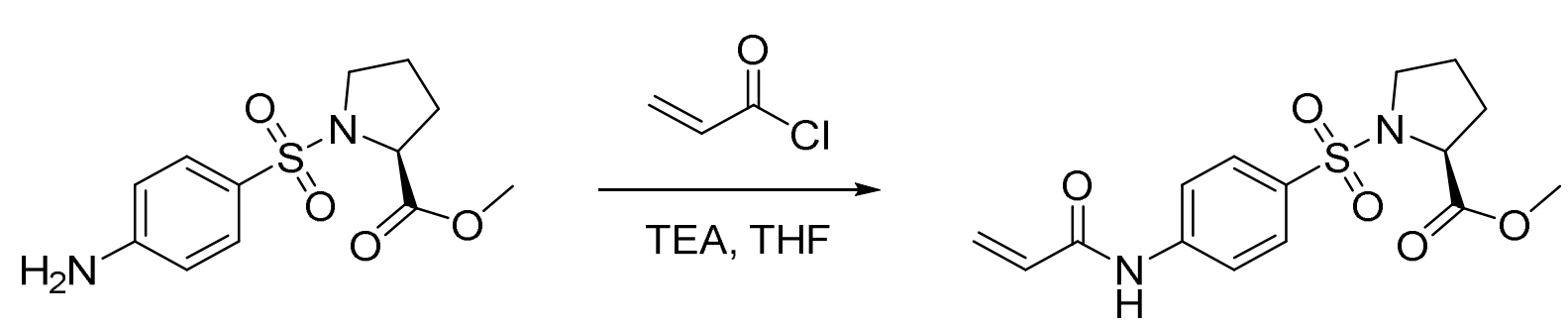

General procedure C, reagents: aniline **22** (710 mg, 2.5 mmol); acryloyl chloride (300 µL, 3.3 mmol); Et_3N (520 µL, 3.8 mmol); THF (5.0 mL); *purification:* LC (10–80% EtOAc in hexane).
Yield: 510 mg, 60%, foamy transparent oil.
^{1}H NMR (400 MHz, Chloroform-*d*) δ: 8.12 (s, 1H), 7.92 – 7.65 (m, 4H), 6.47 (dt, *J* = 16.9, 1.1 Hz, 1H), 6.32 (dd, *J* = 16.8, 10.1 Hz, 1H), 5.82 (dt, *J* = 10.1, 1.2 Hz, 1H), 4.27 (m, 1H), 3.71 (d, *J* = 0.8 Hz, 3H), 3.48 (m, 1H), 3.28 (m, 1H), 2.16 – 1.86 (m, 3H), 1.85 – 1.66 (m, 2H) ppm.
^{13}C NMR (101 MHz, Chloroform-*d*) δ: 172.7, 164.0, 142.3, 132.7, 130.7, 129.1, 128.7, 119.7, 60.5, 52.6, 48.6, 31.0, 24.7 ppm.
LRMS (+ESI) *m/z*: [M+H]$^+$ 339.
HRMS (ESI) *m/z*: [M+H]$^+$ calcd for $C_{15}H_{19}N_2O_5S$: 339.1015; found: 339.1020.
HPLC retention time 4.0 min, 98.9% (Method A).

Methyl ((4-nitrophenyl)sulfonyl)-*D*-prolinate (23)

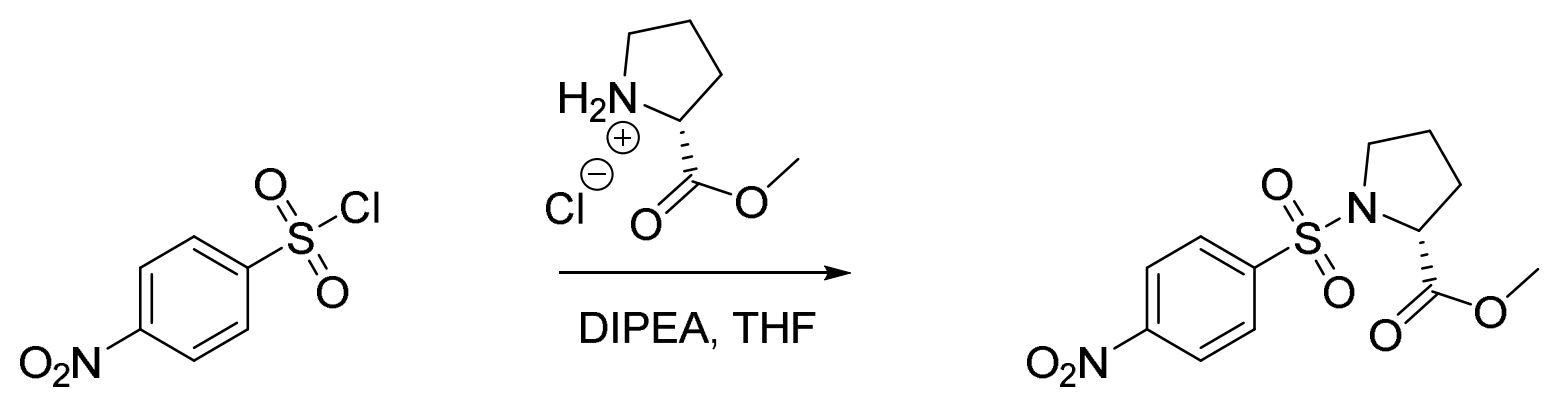

General procedure A, reagents: methyl D-prolinate hydrochloride (75 mg, 0.45 mmol); 4-nitrophenylsulfonyl chloride (100 mg, 0.45 mmol); Et_3N (120 µL, 0.9 mmol); THF (3.0 mL).
Yield: 120 mg, 82%, yellow solid.
^{1}H NMR (400 MHz, Chloroform-*d*) δ: 8.41 – 8.35 (m, 2H), 8.12 – 8.06 (m, 2H), 4.50 – 4.44 (m, 1H), 3.73 (s, 3H), 3.50 – 3.43 (m, 2H), 2.22 – 2.14 (m, 1H), 2.09 – 1.91 (m, 3H) ppm.
^{13}C NMR (101 MHz, Chloroform-*d*) δ: 172.1, 144.6, 128.8, 124.2, 60.5, 52.5, 48.3, 30.9, 24.7 ppm.
LRMS (+ESI) *m/z*: $[M+Na]^+$ 337.

Methyl ((4-aminophenyl)sulfonyl)-*D*-prolinate (24)

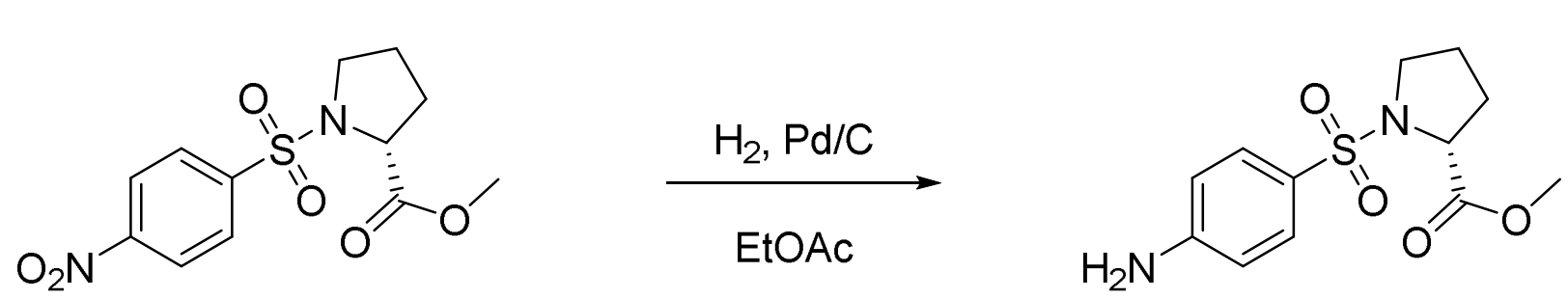

General procedure B, reagents: nitrosulfonamide **23** (120 mg, 0.36 mmol); H_2 (1 atm); 10% Pd/C (12 mg, 10% w/w); EtOAc (6 mL).
Yield: 110 mg, quantitative, yellow solid.
^{1}H NMR (400 MHz, DMSO-*d*$_6$) δ: 7.68 – 7.60 (m, 2H), 6.74-6.67 (m, 2H), 4.27 – 4.21 (m,1H), 3.74 (s, 3H), 3.51 – 3.44 (m, 1H), 3.31 – 3.23 (m, 1H), 2.05 – 1.89 (m, 3H), 1.79 – 1.69 (m, 1H) ppm.
LRMS (+ESI) *m/z*: $[M+H]^+$ 285.

Methyl ((4-acrylamidophenyl)sulfonyl)-*D*-prolinate (5)

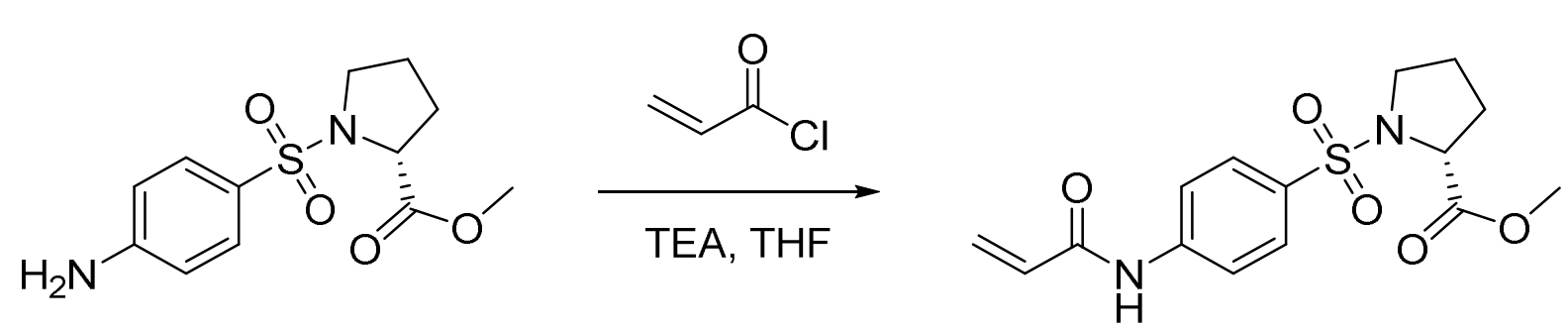

General procedure C, reagents: aniline **24** (110 mg, 0.36 mmol); acryloyl chloride (300 µL, 3.3 mmol); Et_3N (520 µL, 3.8 mmol); THF (5.0 mL); *purification:* LC (20–50% EtOAc in hexane).
Yield: 95 mg, 76%, yellow oil.
^{1}H NMR (400 MHz, Chloroform – *d*) δ: 8.14 (br s, 1H), 7.86 – 7.78 (m, 4H), 6.53 – 6.44 (m, 1H), 6.39 – 6.29 (m, 1H), 5.87 – 5.80 (m, 1H), 4.33 – 4.25 (m, 1H), 3.74 (s, 3H), 3.55 – 3.45 (m, 1H), 3.35 – 3.25 (m, 1H), 2.06 – 1.95 (m, 3H), 1.82 – 1.72 (m, 1H) ppm.
^{13}C NMR (101 MHz, Chloroform-*d*) δ: 172.6, 163.9, 142.2, 132.8, 130.6, 129.0, 128.7, 119.7, 60.4, 52.5, 48.5, 30.9, 24.6 ppm.
LRMS (+ESI) *m/z*: $[M+H]^+$ 339.
HRMS (ESI) *m/z*: $[M+H]^+$ calcd for $C_{15}H_{19}N_2O_5S$: 339.1015; found: 339.1020.
HPLC retention time 3.9 min, 98.1% (Method B).

((4-Acrylamidophenyl)sulfonyl)-*L*-proline (25)

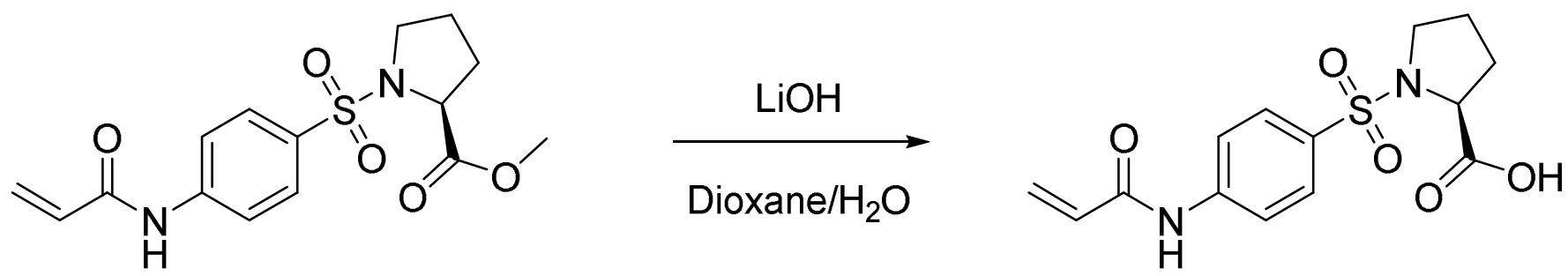

General procedure D, reagents: ester **4** (100 mg, 0.30 mmol); $LiOH.H_2O$ (14 mg, 0.30 mmol); dioxane (0.9 mL); H_2O (0.9 mL).
Yield: 91 mg, 95%, white solid.
^{1}H NMR (400 MHz, DMSO-d_6) δ: 12.76 (s, 1H), 10.58 (s, 1H), 7.97 – 7.86 (m, 2H), 7.85 – 7.73 (m, 2H), 6.47 (dd, J = 16.9, 10.0 Hz, 1H), 6.32 (dd, J = 17.0, 2.0 Hz, 1H), 5.84 (dd, J = 10.0, 2.0 Hz, 1H), 4.08 (dd, J = 8.2, 4.4 Hz, 1H), 3.21 – 3.11 (m, 1H), 1.96 – 1.70 (m, 3H), 1.65 – 1.44 (m, 1H) ppm.
^{13}C NMR (101 MHz, DMSO-d_6) δ: 173.7, 164.2, 143.5, 132.0, 131.9, 128.9, 128.6, 119.6, 60.9, 48.9, 30.9, 24.7 ppm.
LRMS (+ESI) *m/z*: $[M+H]^+$ 325.

(*S*)-1-((4-Acrylamidophenyl)sulfonyl)-*N*-(prop-2-yn-1-yl)pyrrolidine-2-carboxamide (6, C05)

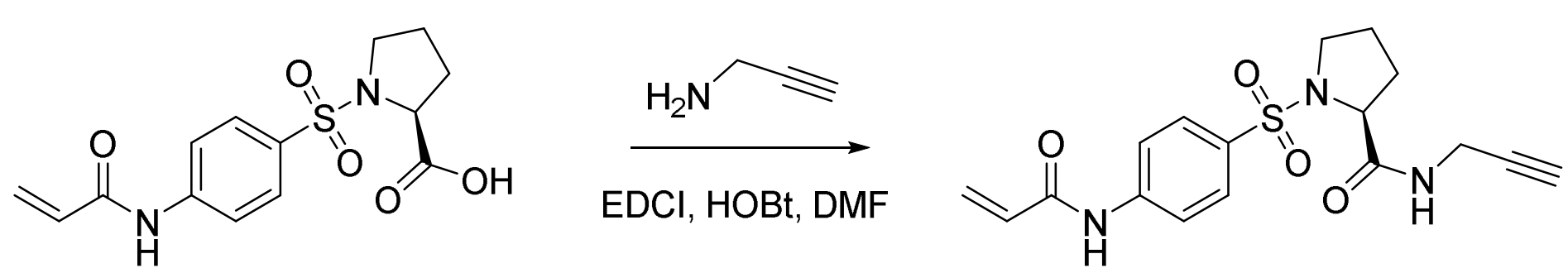

To a stirring solution of acid **25** (85 mg, 0.26 mmol) in anhydrous DMF (1.0 mL) was added HOBt (36 mg, 0.26 mmol) and EDCI.HCl (75 mg, 0.39 mmol). After 10 min, propargylamine (20 µL, 0.31 mmol) was added. After reaction was diluted with water (50 mL) and extracted with ethyl acetate (3x, 10 mL), washed with brine (10 mL), dried ($MgSO_4$), filtered and concentrated *in vacuo*. The crude product was purified by liquid chromatography (0–5% MeOH in CH_2Cl_2).
Yield: 56 mg, 59%, white solid.
^{1}H NMR (400 MHz, DMSO-d_6) δ: 10.58 (s, 1H), 8.43 (t, J = 5.6 Hz, 1H), 7.96 – 7.88 (m, 2H), 7.86 – 7.80 (m, 2H), 6.47 (dd, J = 17.0, 10.0 Hz, 1H), 6.33 (dd, J = 16.9, 2.0 Hz, 1H), 5.84 (dd, J = 10.0, 2.0 Hz, 1H), 4.03 (dd, J = 8.2, 3.5 Hz, 1H), 3.88 (qdd, J = 17.4, 5.6, 2.5 Hz, 2H), 3.42 (ddd, J = 10.1, 6.8, 4.9 Hz, 1H), 3.20 – 3.13 (m, 1H), 3.12 (t, J = 2.5 Hz, 1H), 1.84 – 1.62 (m, 3H), 1.58 – 1.41 (m, 1H) ppm.
^{13}C NMR (101 MHz, DMSO-d_6) δ: 171.5, 164.2, 143.7, 131.9, 131.1, 129.1, 128.6, 119.6, 81.5, 73.4, 61.9, 49.6, 31.1, 28.6, 24.5 ppm.
LRMS (+ESI) *m/z*: $[M+H]^+$ 362.
HRMS (ESI) *m/z*: $[M+H]^+$ calcd for $C_{17}H_{20}N_3O_4S$: 362.1175; found: 362.1185.
HPLC retention time 3.4 min, >99.0% (Method A).

tert-Butyl (S)-2-((4-methoxyphenyl)carbamoyl)pyrrolidine-1-carboxylate (26)

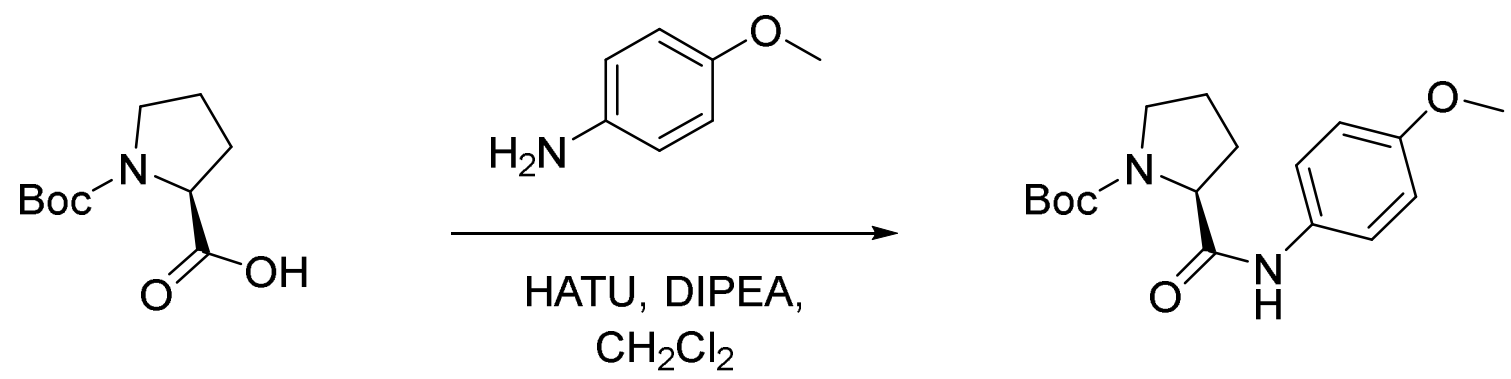

General procedure E, reagents: Boc-proline (2.0 g, 9.3 mmol); 4-methoxyaniline (1.7 g, 14.0 mmol); HATU (4.2 g, 11 mmol); DIPEA (4.8 mL, 28 mmol); CH_2Cl_2 (20 mL); *purification:* LC (2x, 2.5% MeOH in CH_2Cl_2).
Yield: 3.1 g (70% pure), 92%, orange crystalline solid.
^{1}H NMR (400 MHz, Chloroform-*d*) δ: 9.32 (s, 1H), 7.50 – 7.37 (m, 2H), 6.95 – 6.83 (m, 2H), 4.48 (s, 1H), 3.80 (s, 3H), 3.51 – 3.22 (m, 2H), 2.63 – 2.43 (m, 1H), 2.04 – 1.81 (m, 2H), 1.72 – 1.64 (m, 1H), 1.51 (s, 9H) ppm.
^{13}C NMR (101 MHz, Chloroform-*d*) δ: 152.7, 121.4, 121.3, 116.4, 114.8, 114.1, 80.9, 55.8, 55.5, 47.2, 28.4 ppm.

(S)-2-((4-Methoxyphenyl)carbamoyl)pyrrolidin-1-ium chloride (27)

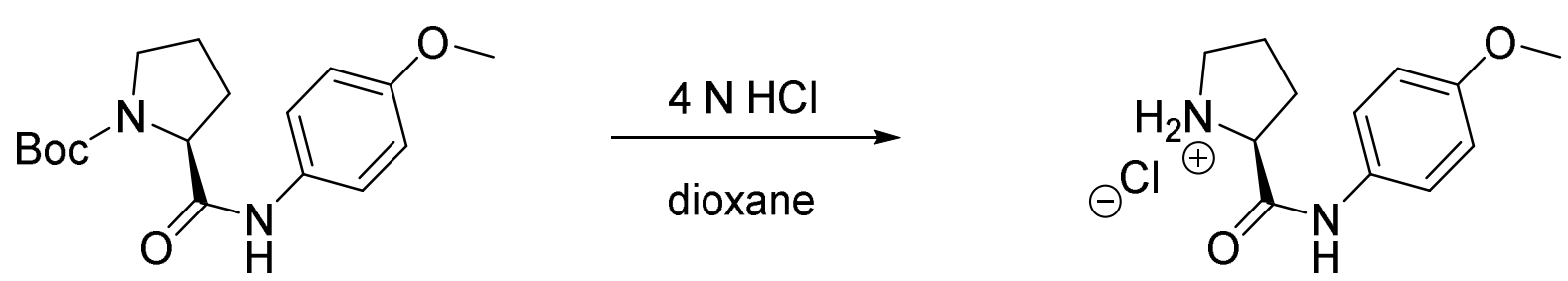

To a stirring solution of amide **26** (70%, 3.1 g, 9.7 mmol) in dioxane (8.0 mL) was added 4 N HCl in dioxane (3.0 mL). After 1 h additional 4 N HCl in dioxane (3.0 mL) and the reaction was stirred for 4 h at rt. The crude suspension was filtered and the precipitate washed with CH_2Cl_2 to afford the desired product which was used without further purification.
Yield: 2.3 g (70% pure), 83%, white solid.
^{1}H NMR (400 MHz, DMSO-d_6) δ: 10.83 (s, 1H), 10.17 (d, J = 45.0 Hz, 2H), 7.66 – 7.49 (m, 2H), 7.00 – 6.88 (m, 2H), 4.37 (s, 1H), 3.74 (s, 3H), 3.26 (d, J = 7.4 Hz, 3H), 2.42 (qt, J = 10.4, 2.5 Hz, 1H), 2.00 – 1.87 (m, 3H) ppm.
^{13}C NMR (101 MHz, DMSO-d_6) δ: 166.7, 156.2, 131.8, 124.9, 124.8, 121.5, 115.3, 114.5, 59.9, 55.7, 46.1, 30.2, 24.1 ppm.

(S)-N-(4-Methoxyphenyl)-1-((4-nitrophenyl)sulfonyl)pyrrolidine-2-carboxamide (28)

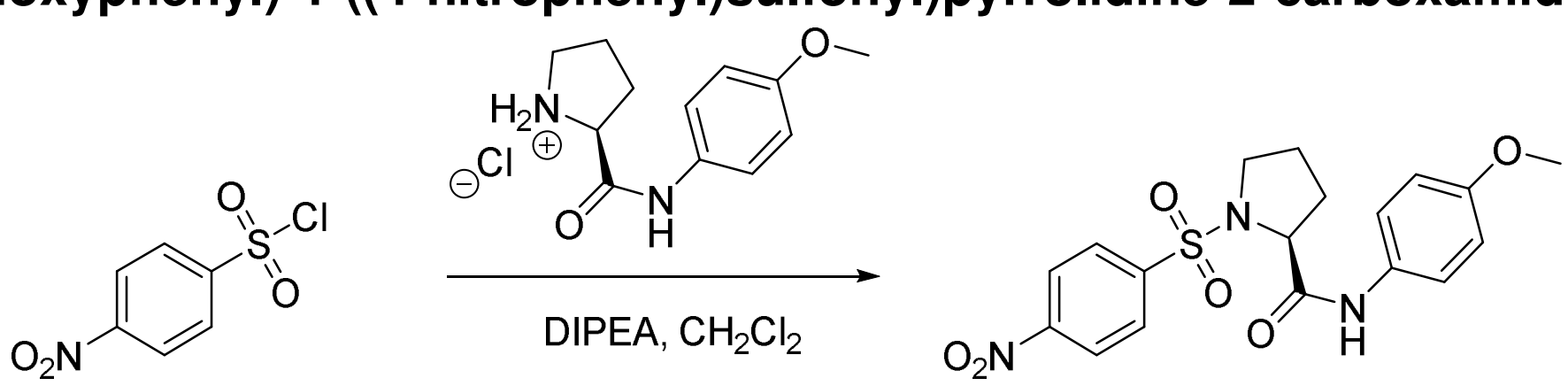

General procedure A, reagents: amine **27** (3.4 g, 13 mmol); 4-nitrophenylsulfonyl chloride (3.0 g, 13 mmol); DIPEA (7.0 mL, 40 mmol); CH_2Cl_2 (10 mL).
Yield: 4.3 g, 80%, orange solid.
^{1}H NMR (400 MHz, Chloroform-*d*) δ: 8.55 – 8.38 (m, 3H), 8.18 – 8.04 (m, 2H), 7.53 – 7.44 (m, 2H), 6.95 – 6.88 (m, 2H), 4.24 (dd, J = 8.6, 2.6 Hz, 1H), 3.72 (ddd, J = 10.4, 7.3, 3.4 Hz, 1H), 3.29 (td, J = 9.7, 6.7 Hz, 1H), 2.40 (ddd, J = 12.7, 6.4, 3.1 Hz, 1H), 1.94 (dddd, J = 15.9, 12.9, 7.2, 4.6 Hz, 1H), 1.84 – 1.54 (m, 2H) ppm.

^{13}**C NMR** (101 MHz, Chloroform-*d*) δ: 168.04, 156.85, 150.6, 141.57, 130.23, 129.1, 124.7, 121.9, 114.2, 63.1, 55.5, 50.2, 30.1, 24.5 ppm.

(*S*)-1-((4-Aminophenyl)sulfonyl)-*N*-(4-methoxyphenyl)pyrrolidine-2-carboxamide (29)

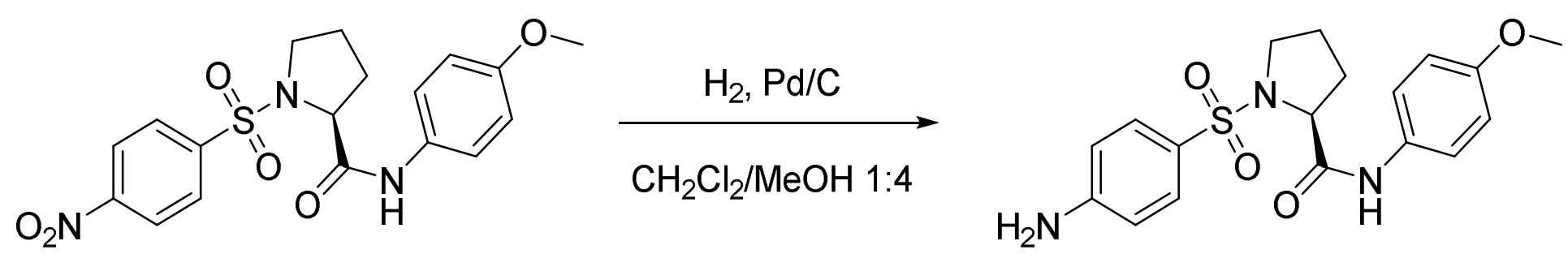

General procedure B, reagents: nitrosulfonamide **28** (9.2 g, 23 mmol); H_2 (1 atm); 10% Pd/C (920 mg, 10% w/w); CH_2Cl_2/MeOH (1:4, 150 mL).
Yield: 3.8 g, 45%, white solid.
^{1}**H NMR** (400 MHz, DMSO-*d*$_6$) δ: 9.69 (s, 1H), 7.62 – 7.40 (m, 4H), 6.95 – 6.78 (m, 2H), 6.71 – 6.57 (m, 2H), 6.08 (d, *J* = 4.9 Hz, 2H), 4.10 – 3.95 (m, 1H), 3.73 (s, 3H), 3.46 – 3.37 (m, 1H), 3.19 – 3.02 (m, 1H), 1.89 – 1.67 (m, 3H), 1.58 – 1.41 (m, 1H) ppm.

(*S*)-1-((4-Acrylamidophenyl)sulfonyl)-*N*-(4-methoxyphenyl)pyrrolidine-2-carboxamide (7)

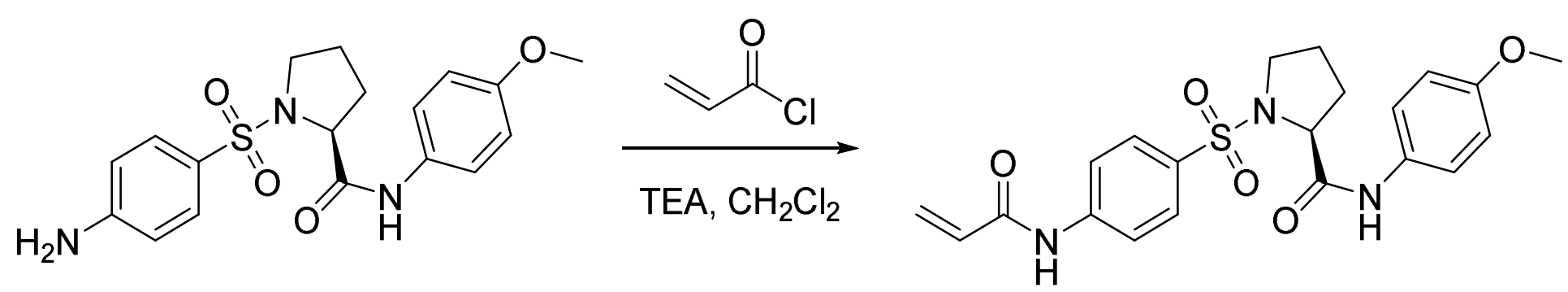

General procedure C, reagents: aniline **29** (60 mg, 0.15 mmol); acryloyl chloride (13 µL, 0.23 mmol, 2x); Et_3N (67 µL, 0.47 mmol, 2x); CH_2Cl_2 (5.0 mL); *purification:* LC (40–100% EtOAc in hexane).
Yield: 31 mg, 44%, white crystalline solid.
^{1}**H NMR** (400 MHz, DMSO-*d*$_6$) δ: 10.59 (s, 1H), 9.81 (s, 1H), 7.95 – 7.88 (m, 2H), 7.88 – 7.77 (m, 2H), 7.56 – 7.47 (m, 2H), 6.93 – 6.84 (m, 2H), 6.47 (dd, *J* = 17.0, 10.0 Hz, 1H), 6.33 (dd, *J* = 16.9, 2.0 Hz, 1H), 5.85 (dd, *J* = 10.1, 2.0 Hz, 1H), 4.20 – 4.10 (m, 1H), 3.73 (s, 3H), 3.53 – 3.42 (m, 1H), 3.29 – 3.15 (m, 1H), 1.95 – 1.75 (m, 3H), 1.59 – 1.39 (m, 1H) ppm.
^{13}**C NMR** (101 MHz, Methanol-*d*$_4$) δ: 169.6, 163.7, 155.4, 143.2, 131.8, 131.4, 131.0, 128.6, 128.2, 121.2, 119.2, 113.8, 61.8, 55.2, 49.2, 31.1, 24.3 ppm.
LRMS (+ESI) *m/z*: $[M+H]^+$ 430.
HRMS (+ESI) *m/z*: $[M+H]^+$ calcd $C_{21}H_{24}N_3O_5S$ 430.1431, found 430.1425.
HPLC retention time 4.2 min, >99.0% (Method A).

((4-Acrylamidophenyl)sulfonyl)-*D*-proline (30)

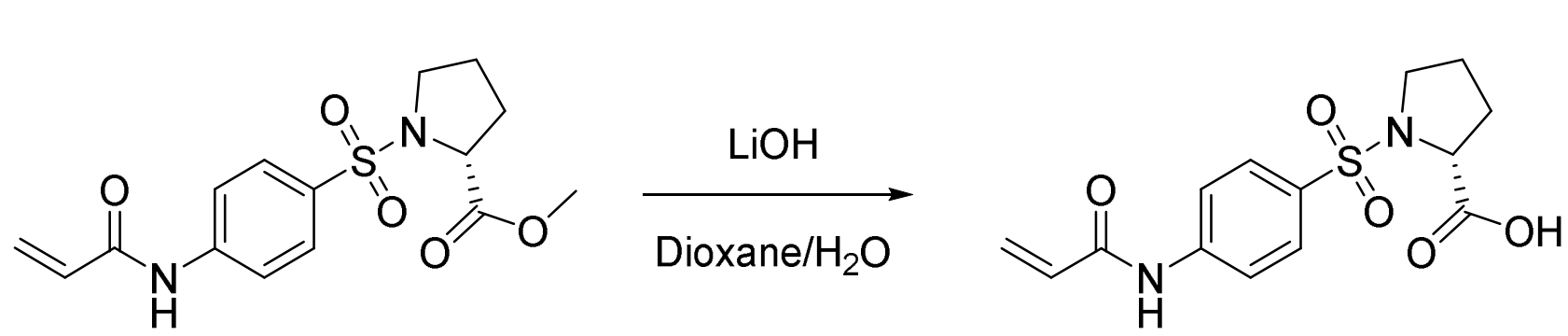

General procedure D, reagents: ester **5** (175 mg, 0.52 mmol); LiOH.H_2O (65 mg, 1.55 mmol); dioxane (5.0 mL); H_2O (2.5 mL).
Yield: 126 mg, 75%, colourless oil.
LRMS (+ESI) *m/z*: $[M+H]^+$ 325.

(*R*)-1-((4-Acrylamidophenyl)sulfonyl)-*N*-(4-methoxyphenyl)pyrrolidine-2-carboxamide (8)

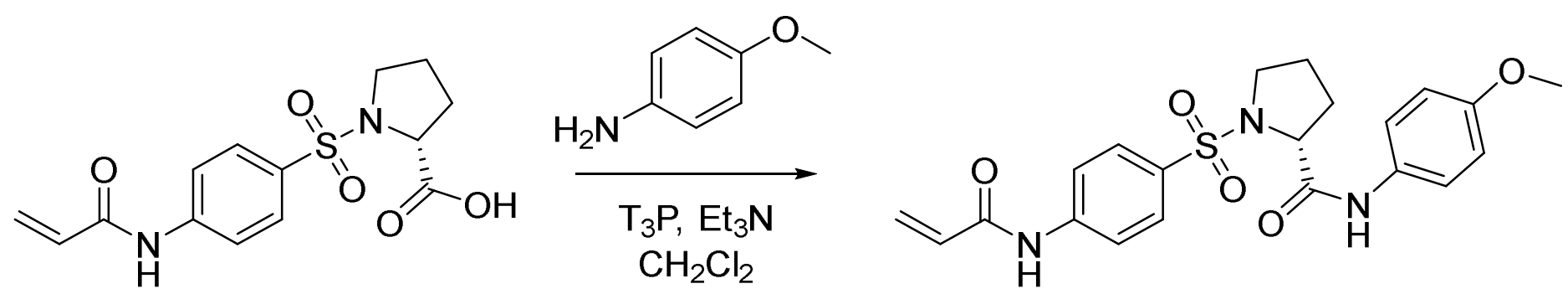

General procedure E, reagents: acid **30** (25 mg, 0.08 mmol); 4-methoxyaniline (14 mg, 0.12 mmol); T_3P (50% in EtOAc, 98 µL, 0.15 mmol); Et_3N (21 µL, 0.15 mmol); CH_2Cl_2 (1.0 mL); *purification:* LC (20–60% EtOAc in hexane).
Yield: 17 mg, 51%, white solid.
^{1}H NMR (400 MHz, DMSO-*d*$_6$) δ: 10.58 (s, 1H), 9.80 (s, 1H), 7.94 – 7.88 (m, 2H), 7.87 – 7.81 (m, 2H), 7.55 – 7.48 (m, 2H), 6.93 – 6.85(m, 2H), 6.46 (dd, J = 17.1, 9.9 Hz, 1H), 6.33 (dd, J = 17.1, 1.8 Hz, 1H), 5.84 (dd, J = 9.9, 1.8 Hz, 1H), 6.14 (dd, J = 7.3, 5.1 Hz, 1H), 3.51 – 3.42 (m, 1H), 3.25 – 3.17 (m, 1H), 1.92 – 1.77 (m, 3H), 1.58 – 1.44 (m, 1H) ppm.
LRMS (+ESI) *m/z*: $[M+H]^+$ 430.
HRMS (+ESI) *m/z*: $[M+H]^+$ calcd $C_{21}H_{24}N_3O_5S$ 430.1431, found 430.1454.
HPLC retention time 4.5 min, >99.0% (Method B).

***tert*-Butyl (4-(prop-2-yn-1-yloxy)phenyl)carbamate (31)**

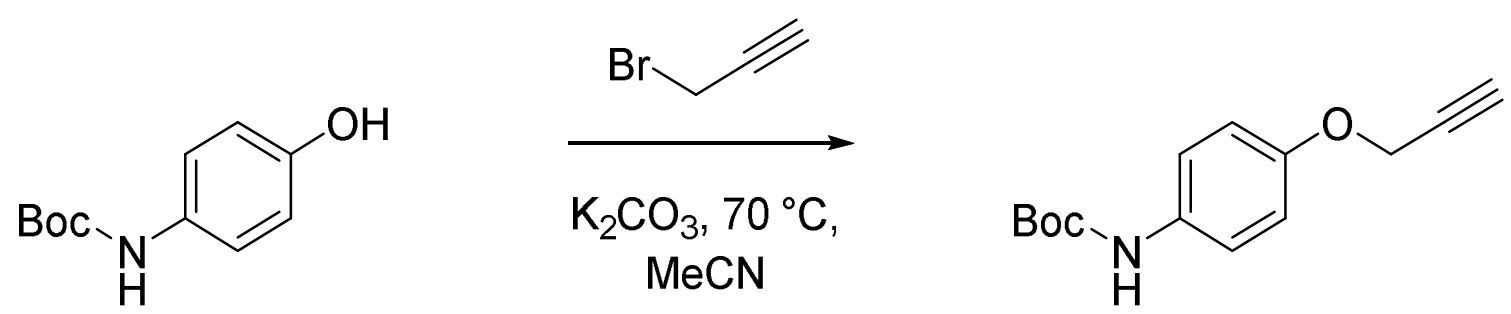

To a stirring solution of tert-butyl (4-hydroxyphenyl)carbamate (250 mg, 1.2 mmol) in MeCN (1.5 mL) in a microwave vial were added K_2CO_3 (250 mg, 1.8 mmol) and propargyl bromide (80% in toluene, 140 µL, 1.3 mmol). The vial was sealed and heated 70 °C, and the reaction was stirred for 14 h. The crude mixture was diluted with EtOAc (20 mL) and washed with H2O (15 mL). The aqueous phase was extracted with EtOAc (2x, 10 mL) and the combined organic phase was washed with brine (10 mL), dried ($MgSO_4$), filtered and concentrated *in vacuo* to afford the desired product which was used without further purification.
Yield: 300 mg, quantitative, orange oil.
^{1}H NMR (400 MHz, Chloroform-*d*) δ: 7.34 – 7.29 (m, 2H), 7.02 – 6.88 (m, 2H), 6.40 (s, 1H), 4.68 (d, *J* = 2.4 Hz, 2H), 2.53 (t, *J* = 2.4 Hz, 1H), 1.54 (s, 9H) ppm
LRMS (+ESI) *m/z*: $[M+H]^+$ 248.

4-(Prop-2-yn-1-yloxy)benzenaminium chloride (32)

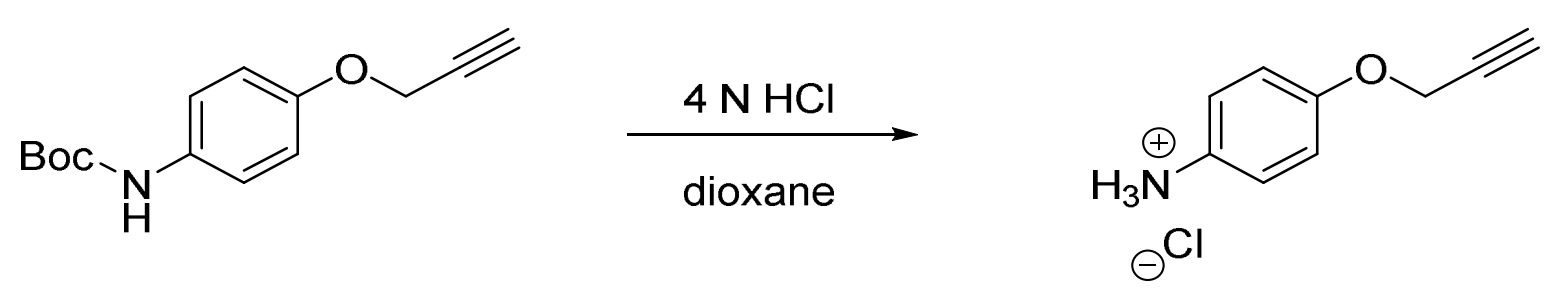

To a stirring solution of alkyne 31 (300 mg, 1.2 mmol) in dioxane (1.0 mL) cooled to 0 °C was added 4 N HCl in dioxane (1.5 mL). After 16 h the crude was concentrated *in vacuo* and azeotroped with MeOH (3x) to remove excess dioxane and afford the desired product which was used without further purification.

Yield: 180 mg, 80%, pale yellow solid.
^{1}H NMR (400 MHz, DMSO-*d*$_6$) δ: 10.22 (s, 3H), 7.40 – 7.29 (m, 2H), 7.14 – 7.00 (m, 2H), 4.83 (d, *J* = 2.3 Hz, 2H), 3.61 (t, *J* = 2.4 Hz, 1H). ppm

(*S*)-1-((4-Acrylamidophenyl)sulfonyl)-*N*-(4-(prop-2-yn-1-yloxy)phenyl)pyrrolidine-2-carboxamide (9)

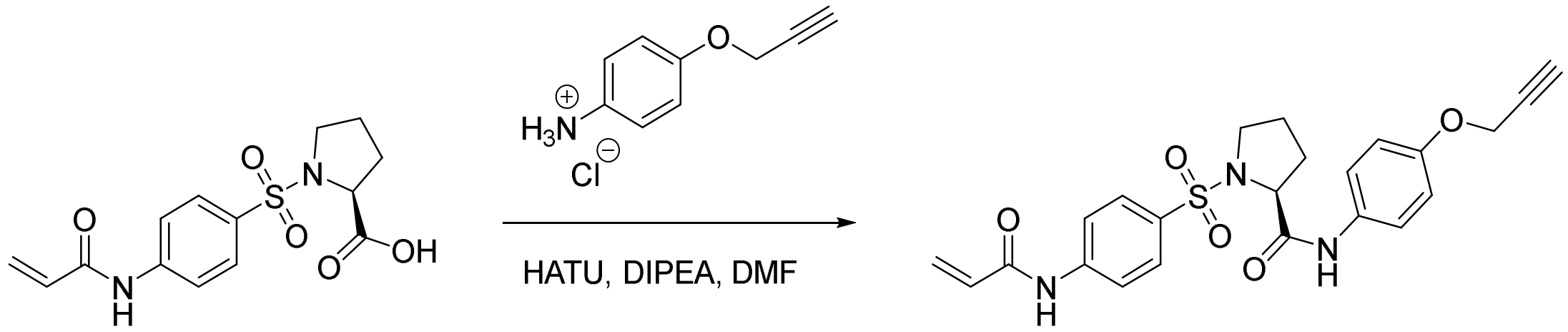

General procedure E, reagents: acid **25** (50 mg, 0.15 mmol); aniline **32** (42 mg, 0.23 mmol); HATU (88 mg, 0.23 mmol); DIPEA (140 µL, 0.75 mmol); DMF (800 µL); *purification:* LC (20–80% EtOAc in hexane).
Yield: 36 mg, 53%, yellow solid.
^{1}H NMR (400 MHz, Chloroform-*d*) δ: 9.22 (s, 1H), 8.78 (s, 1H), 7.91 – 7.84 (m, 2H), 7.84 – 7.75 (m, 2H), 7.56 – 7.45 (m, 2H), 7.01 – 6.86 (m, 2H), 6.49 (dd, *J* = 16.9, 1.4 Hz, 1H), 6.33 (dd, *J* = 16.9, 10.1 Hz, 1H), 5.81 (dd, *J* = 10.2, 1.4 Hz, 1H), 4.68 (d, *J* = 2.4 Hz, 2H), 4.14 (dd, *J* = 8.9, 3.3 Hz, 1H), 3.63 (ddd, *J* = 10.3, 7.0, 3.2 Hz, 1H), 3.24 (td, *J* = 9.8, 6.4 Hz, 1H), 2.54 (t, *J* = 2.4 Hz, 1H), 1.87 – 1.76 (m, 1H), 1.73 – 1.60 (m, 2H), 1.27 – 1.24 (m, 1H) ppm.
^{13}C NMR (101 MHz, Chloroform-*d*) δ: 169.5, 164.4, 154.6, 143.4, 131.1, 130.6, 129.6, 129.1, 129.0, 122.0, 121.9, 119.8, 119.8, 115.3, 78.4, 75.6, 62.9, 56.1, 5.14, 30.1, 24.4 ppm.
LRMS (+ESI) *m/z*: $[M+H]^+$ 454.
HRMS (ESI) *m/z*: $[M+H]^+$ calcd for $C_{23}H_{24}N_3O_5S$: 454.1437; found: 454.1418.
HPLC retention time 4.3 min, 97.7% (Method A).

Ethyl (*S*)-4-(1-((4-Acrylamidophenyl)sulfonyl)pyrrolidine-2-carboxamido)benzoate (33)

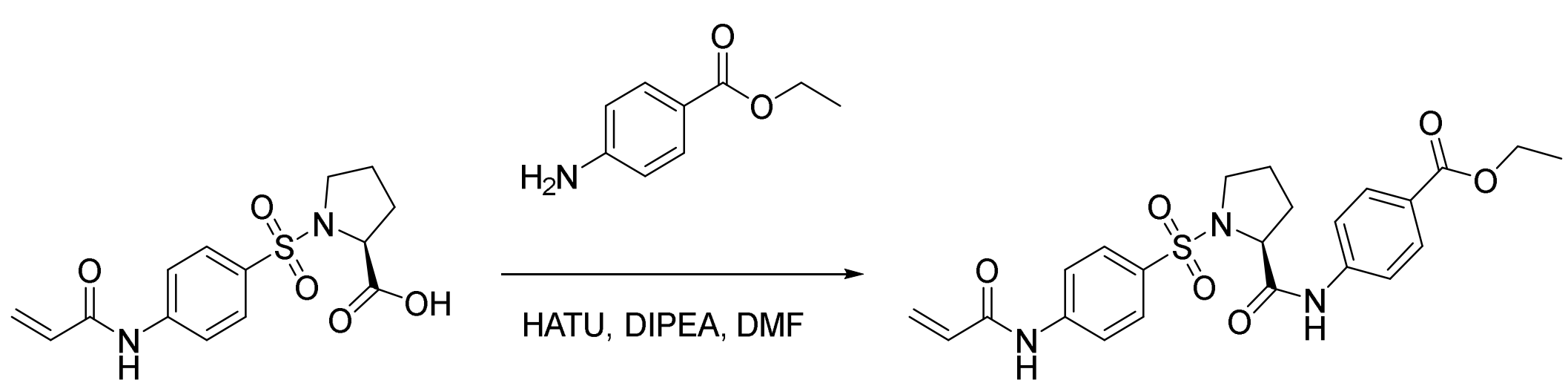

General procedure E, reagents: acid **25** (250 mg, 0.77 mmol); ethyl 4-aminobenzoate (130 mg, 0.77 mmol); HATU (350 mg, 0.92 mmol); DIPEA (400 µL, 2.3 mmol); CH_2Cl_2 (5.0 mL); *purification:* LC (20–60% EtOAc in hexane).
Yield: 250 mg (1:1 mixture with tetramethylurea), 54%, orange foam.
^{1}H NMR (400 MHz, Chloroform-*d*) δ: 9.01 (s, 1H), 8.13 (s, 1H), 8.08 – 8.03 (m, 2H), 7.93 – 7.83 (m, 4H), 7.73 – 7.68 (m, 2H), 6.53 (dd, *J* = 16.8, 1.2 Hz, 1H), 6.33 (dd, *J* = 16.8, 10.2 Hz, 1H), 5.87 (dd, *J* = 10.2, 1.2 Hz, 1H), 4.39 (q, *J* = 7.1 Hz, 2H), 4.18 (dd, *J* = 8.7, 2.7 Hz, 1H), 3.67 (ddd, *J* = 10.3, 7.2, 3.4 Hz, 1H), 3.27 (td, *J* = 9.7, 6.6 Hz, 1H), 2.42 – 2.32 (m, 1H), 1.83 (d, *J* = 18.5 Hz, 3H), 1.72 (ddt, *J* = 12.8, 6.6, 3.3 Hz, 1H), 1.67 – 1.57 (m, 1H), 1.42 (t, *J* = 7.1 Hz, 3H) ppm.

(*S*)-4-(1-((4-Acrylamidophenyl)sulfonyl)pyrrolidine-2-carboxamido)benzoic acid (34)

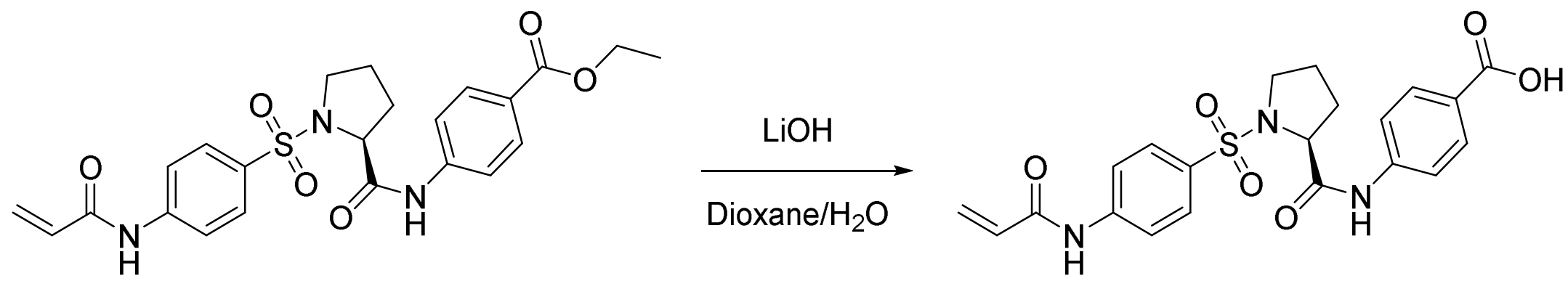

General procedure D, reagents: ester **33** (230 mg, 0.48 mmol, 1:1 mixture with tetramethylurea); LiOH.H_2O (30 mg, 0.72 mmol); dioxane (1.0 mL); H_2O (1.0 mL).
Yield: 200 mg, 92%, white solid.
^{1}H NMR (400 MHz, DMSO-*d*$_6$) δ: 12.77 (s, 1H), 10.60 (s, 1H), 10.28 (s, 1H), 7.98 – 7.89 (m, 4H), 7.89 – 7.83 (m, 2H), 7.78 – 7.72 (m, 2H), 6.47 (dd, *J* = 17.0, 10.0 Hz, 1H), 6.33 (dd, *J* = 17.0, 2.0 Hz, 1H), 5.85 (dd, *J* = 10.0, 2.0 Hz, 1H), 4.21 (t, *J* = 6.1 Hz, 1H), 3.52 – 3.43 (m, 1H), 3.28 – 3.17 (m, 0H), 1.89 (s, 3H), 1.65 – 1.46 (m, 1H) ppm.

(*S*)-1-((4-Acrylamidophenyl)sulfonyl)-*N*-(4-((1-methyl-1H-pyrazol-3-yl)carbamoyl)phenyl)pyrrolidine-2-carboxamide (10, IMP-2552)

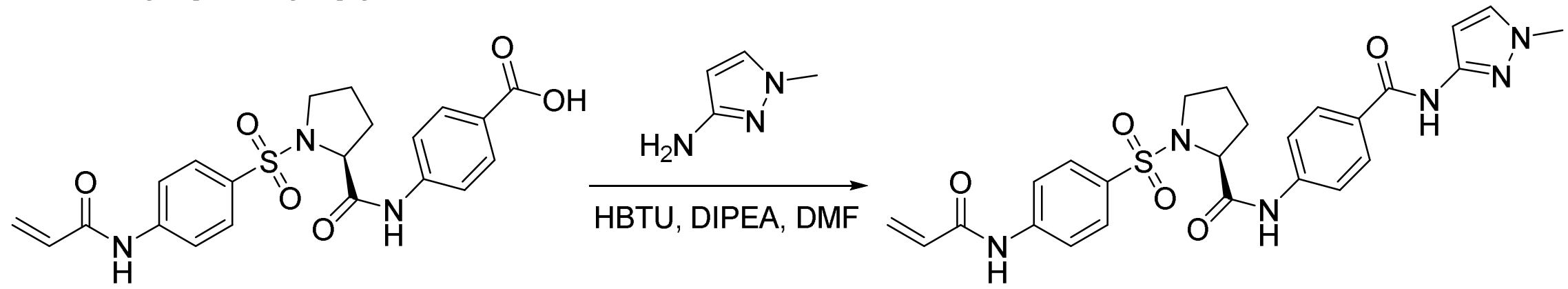

General procedure E, reagents: acid **34** (120 mg, 0.26 mmol); 1-methyl-1H-pyrazol-3-amine (26 mg, 0.26 mmol); HBTU (100 mg, 0.26 mmol); DIPEA (120 µL, 0.66 mmol); CH_2Cl_2 (5.0 mL); *purification:* LC (0–3% MeOH in CH_2Cl_2), then preparative HPLC (20–95% 0.1% formic acid MeCN in H_2O).
Yield: 15 mg, 11%, white solid.
^{1}H NMR (400 MHz, DMSO-*d*$_6$) δ: 10.72 (s, 1H), 10.67 (s, 1H), 10.26 (s, 1H), 8.04 – 7.97 (m, 2H), 7.98 – 7.92 (m, 2H), 7.91 – 7.79 (m, 2H), 7.77 – 7.68 (m, 2H), 7.61 (s, 1H), 6.59 (m, 1H), 6.49 (dd, *J* = 16.9, 10.1 Hz, 1H), 6.33 (d, *J* = 16.9 Hz, 1H), 5.85 (d, *J* = 10.1 Hz, 1H), 4.22 (t, *J* = 6.0 Hz, 1H), 3.79 (s, 3H), 3.49 (dt, *J* = 10.7, 6.0 Hz, 1H), 3.24 (dt, *J* = 10.0, 6.2 Hz, 1H), 1.99 – 1.81 (m, 3H), 1.56 (m, 1H) ppm.
^{13}C NMR (101 MHz, DMSO-*d*$_6$) δ: 171.0, 164.2, 164.2, 147.6, 143.8, 142.1, 131.9, 131.4, 129.3, 129.2, 129.1, 129.1, 128.6, 119.7, 119.2, 97.9, 62.3, 49.6, 38.8, 31.6, 24.8 ppm.
LRMS (+ESI) *m/z*: $[M+H]^+$ 523.
HRMS (+ESI) *m/z*: $[M+H]^+$ calcd for $C_{25}H_{27}N_6O_5S$ 523.1758, found 523.1778.
HPLC retention time 3.6 min, >99.0% (Method A).
Chiral HPLC retention time 5.5 min.

tert-Butyl (4-((1-methyl-1H-pyrazol-3-yl)carbamoyl)phenyl)carbamate (35)

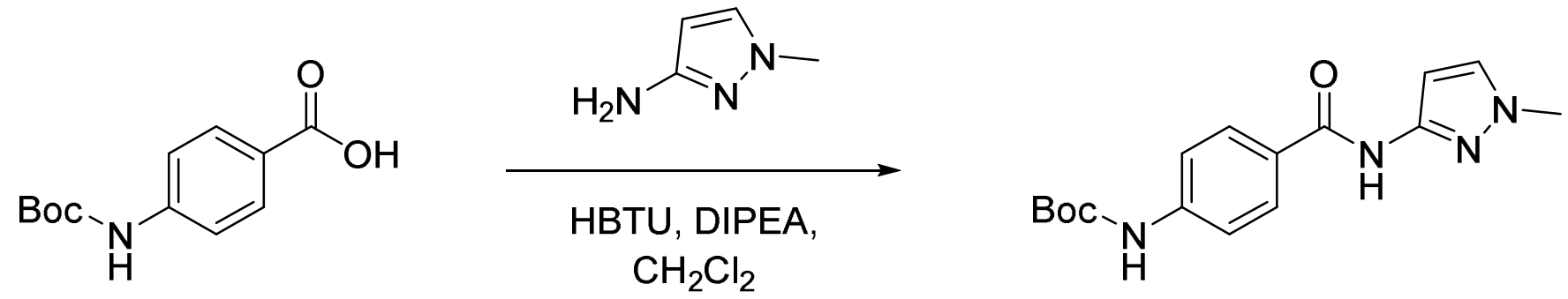

General procedure E, reagents: 4-Boc-aminobenzoic acid (551 mg, 2.3 mmol); 1-methyl-1*H*-pyrazol-3-amine (205 mg, 2.1 mmol); HBTU (961 mg, 2.5 mmol); DIPEA (920 µL, 5.3 mmol); DMF (4 mL); *purification:* LC (10–90% EtOAc in hexane).
Yield: 610 mg, 91%, white solid.
^{1}H NMR (400 MHz, Methanol-d_4) δ: 7.90 – 7.86 (m, 2H), 7.59 – 7.54 (m, 2H), 7.52 (d, *J* = 2.3 Hz, 1H), 6.61 (d, *J* = 2.3 Hz, 1H), 3.85 (s, 3H), 1.55 (s, 9H) ppm.
LRMS (+ESI) *m/z*: $[M+H]^+$ 317.

4-Amino-*N*-(1-methyl-1H-pyrazol-3-yl)benzamide hydrogen chloride (36)

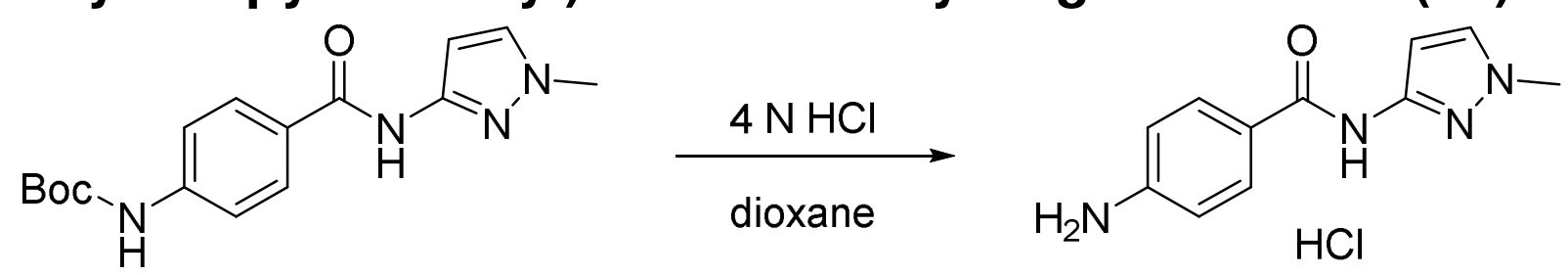

A solution of carbamate **35** (300 mg, 1.0 mmol) in 4 N HCl in dioxane (2.5 mL) was stirred at rt for 23 h. To the crude suspension was added CH_2Cl_2 and filtered *in vacuo*. The precipitate washed with CH_2Cl_2 (20 mL) and hexane (10 mL) to afford the desired product which was used without further purification.
Yield: 240 mg, 99%, white solid.
^{1}H NMR (400 MHz, DMSO-d_6) δ: 10.88 (s, 1H), 8.03 (d, *J* = 8.2 Hz, 1H), 7.38 (d, *J* = 8.2 Hz, 1H), 6.58 (d, *J* = 2.3 Hz, 1H), 4.16 (s, 2H), 3.79 (s, 3H) ppm.
LRMS (+ESI) *m/z*: $[M+H]^+$ 217.

(*R*)-1-((4-Acrylamidophenyl)sulfonyl)-*N*-(4-((1-methyl-1H-pyrazol-3-yl)carbamoyl)phenyl)pyrrolidine-2-carboxamide (11)

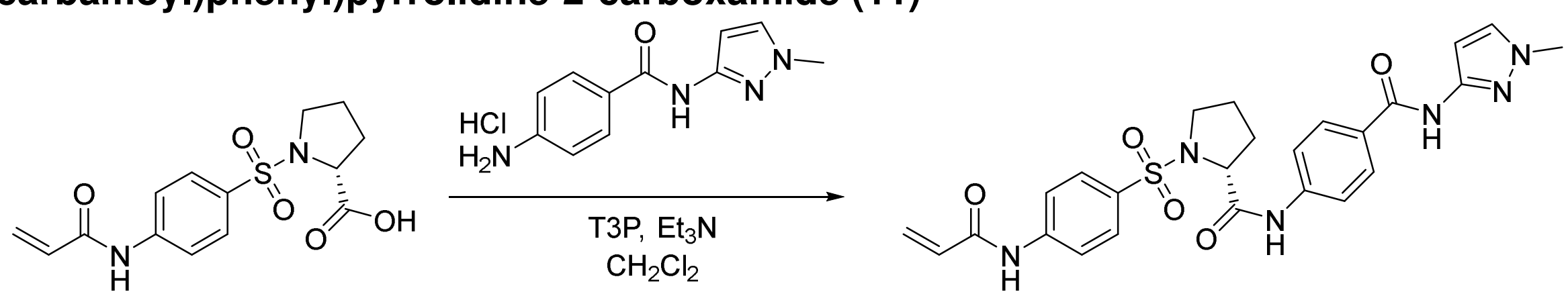

General procedure E, reagents: acid **35** (50 mg, 0.15 mmol); aniline **X** (40 mg, 0.19 mmol); T_3P (50% in EtOAc, 196 µL, 0.31 mmol); Et_3N (86 µL, 0.62 mmol); CH_2Cl_2 (2.0 mL); *purification:* LC (0–10% MeOH in CH_2Cl_2).
Yield: 19 mg, 24%, off-white solid.
^{1}H NMR (400 MHz, DMSO-d_6) δ: 10.71 (s, 1H), 10.59 (s, 1H), 10.21 (s, 1H), 8.03 – 7.96 (m, 2H), 7.95 – 7.89 (m, 2H), 7.88 – 7.82 (m, 2H), 7.74 – 7.69 (m, 2H), 7.60 (d, J = 2.1 Hz, 1H), 6.58 (d, J = 2.1 Hz, 1H), 6.46 (dd, J = 16.9, 10.0 Hz, 1H), 6.33 (dd, J = 16.9, 1.8 Hz, 1H), 5.84 (dd, J = 10.0, 1.8 Hz, 1H), 4.23 – 4.17 (m, 1H), 3.78 (s, 3H), 3.53 – 3.44 (m, 1H), 3.27 – 3.18 (m, 1H), 1.94 – 1.82 (m, 3H), 1.60 – 1.49 (m, 1H) ppm.
LRMS (+ESI) *m/z*: $[M+H]^+$ 523.
HRMS (+ESI) *m/z*: $[M+H]^+$ calcd for $C_{25}H_{27}N_6O_5S$ 523.1758, found 523.1768.
HPLC retention time 4.09 min, 94.4% (Method B).

Chiral HPLC retention time 2.6 min.

4-Nitro-1-(prop-2-yn-1-yl)-1H-pyrazole (37)

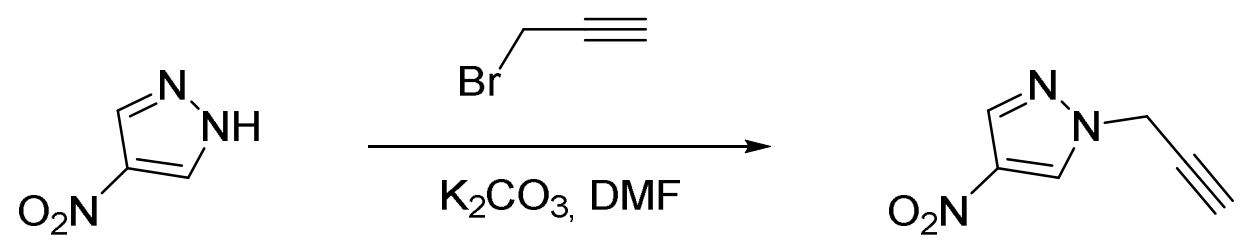

To a transparent stirring solution of 4-nitropyrazole (2.8 g, 18 mmol) in DMF (2.0 mL), were added K_2CO_3 (6.1 g, 28 mmol) and propargyl bromide (80% in toluene, 1.4 mL, 28 mmol). The reaction mixture immediately turned brown, and was stirred at rt for 1 h, before quenching with H_2O (50 mL). The aqueous phase was extracted with EtOAc (10 mL, 3x) and the combined organic phase was washed with brine (10 mL), dried ($MgSO_4$), filtered and concentrated *in vacuo*. The crude mixture was purified by liquid chromatography (0–20% EtOAc in hexane) to afford the desired compound as a pale-yellow oil (2.3 g, 88%).

^{1}H NMR (400 MHz, Chloroform-*d*) δ: 8.44 (s, 1H), 8.12 (s, 1H), 5.01 (d, *J* = 2.6 Hz, 2H), 2.70 (t, *J* = 2.6 Hz, 1H) ppm.

1-(Prop-2-yn-1-yl)-1H-pyrazol-4-amine (38)

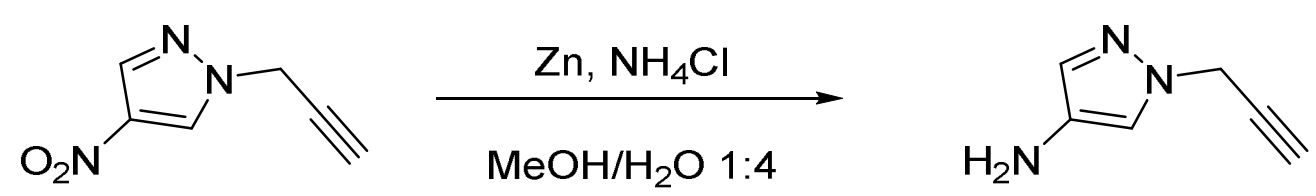

To a clear stirring solution of pyrazole **37** (500 mg, 3.3 mmol) in MeOH (10 mL), were added NH_4Cl (1.8 g, 33 mmol) and zinc (1.1 g, 17 mmol). The reaction mixture slowly turned to dark orange, and after 30 min H_2O (1.0 mL) was added. After 4 h, NH_4Cl (1.8 g, 33 mmol) in H_2O (1.0 mL) and zinc (1.1 g, 17 mmol) were added to the solution. After 16 h, the reaction mixture was filtered through celite and washed with MeOH, then concentrated *in vacuo*. The crude mixture was purified by liquid chromatography (50–100% EtOAc in hexane) to afford the desired compound as a brown oil (60 mg, 15%) which was used immediately in the next step.

^{1}H NMR (400 MHz, DMSO-*d*$_6$) δ: 7.07 (s, 1H), 6.94 (s, 1H), 4.82 (d, *J* = 2.5 Hz, 2H), 3.89 (broad s, 2H), 3.41 (t, *J* = 2.6 Hz, 1H) ppm.

(*S*)-1-((4-Acrylamidophenyl)sulfonyl)-*N*-(4-((1-(prop-2-yn-1-yl)-1H-pyrazol-4-yl)carbamoyl)phenyl)pyrrolidine-2-carboxamide (12, IMP-2566)

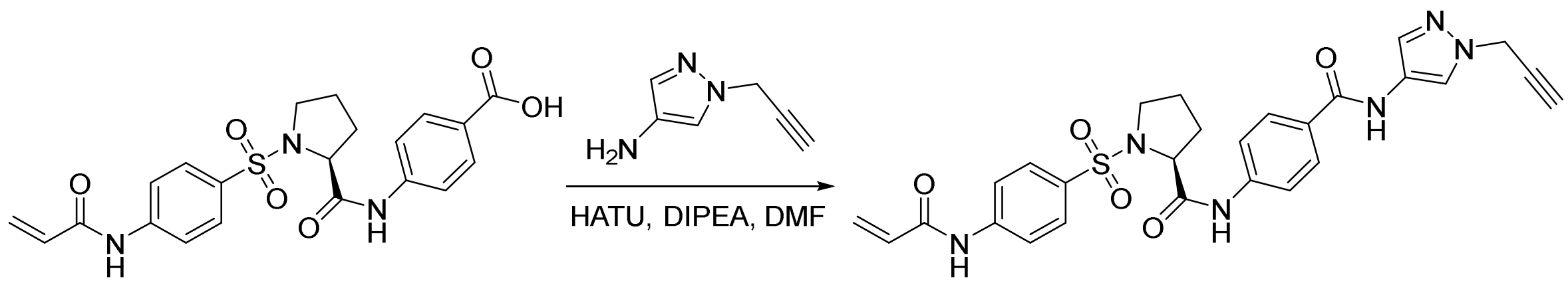

General procedure E, reagents: acid **34** (95 mg, 0.21 mmol); aminopyrazole **X** (52 mg, 0.21 mmol); HATU (81 mg, 0.21 mmol); DIPEA (150 µL, 0.85 mmol); DMF (1.0 mL); *purification:* preparative HPLC (30–95% 0.1% formic acid MeCN in H_2O).
Yield: 60 mg, 51%, pale pink solid.
^{1}H NMR (400 MHz, DMSO-d_6) δ: 10.71 (s, 1H), 10.44 (s, 1H), 10.31 (s, 1H), 8.16 (s, 1H), 8.00 – 7.91 (m, 4H), 7.90 – 7.83 (m, 2H), 7.81 – 7.74 (m, 2H), 7.64 (s, 1H), 6.49 (dd, *J* = 16.9, 10.1 Hz, 1H), 6.33 (dd, *J* = 17.0, 1.9 Hz, 1H), 5.85 (dd, *J* = 9.9, 2.0 Hz, 1H), 5.04 (d, *J* = 2.6 Hz, 2H), 4.23 (t, *J* = 6.0 Hz, 1H), 3.56 – 3.47 (m, 2H), 3.24 (dt, *J* = 10.8, 6.6 Hz, 1H), 1.96 – 1.81 (m, 3H), 1.55 (p, *J* = 7.4, 6.4 Hz, 1H) ppm.
^{13}C NMR (101 MHz, DMSO-d_6) δ: 170.96, 164.20, 163.38, 143.73, 142.02, 131.86, 131.46, 131.34, 129.20, 129.06, 128.73, 128.58, 122.75, 121.01, 119.64, 119.28, 79.15, 76.71, 62.33, 49.63, 41.45, 31.53, 24.81 ppm.
LRMS (+ESI) *m/z*: $[M+H]^+$ 547.
HRMS (+ESI) *m/z*: $[M+H]^+$ calcd for $C_{27}H_{27}N_6O_5S$ 547.1764, found 547.1757.
HPLC retention time 3.6 min, >99.0% (Method B).

(*S*)-1-((4-Acrylamidophenyl)sulfonyl)-*N*-(3-methoxyphenyl)pyrrolidine-2-carboxamide (13, IMP-2505)

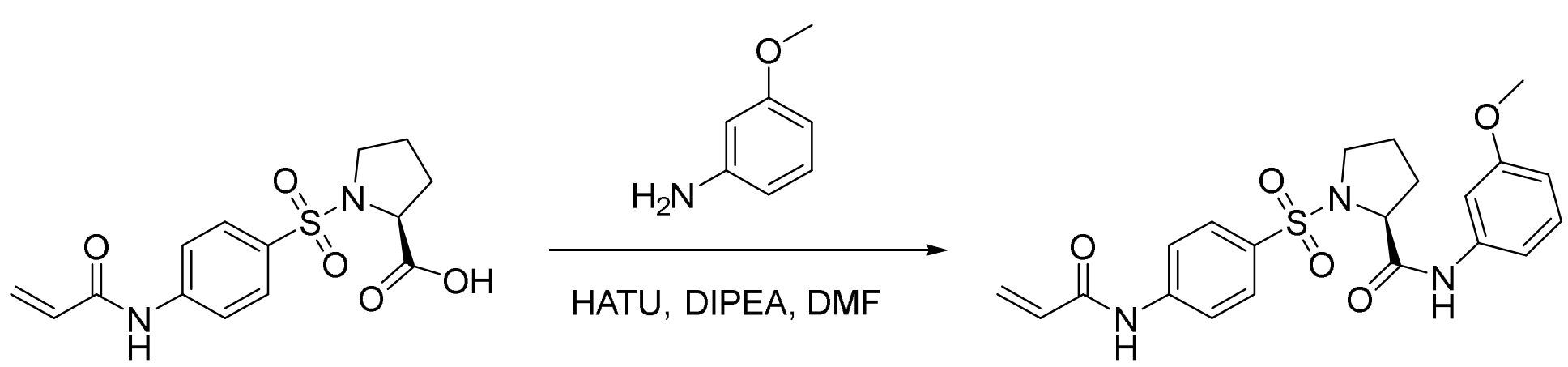

General procedure E, reagents: acid **25** (30 mg, 0.09 mmol); 3-methoxyaniline (17 mg, 0.13 mmol); HATU (51 mg, 0.13 mmol); DIPEA (63 µL, 0.36 mmol); DMF (1.0 mL); *purification:* reverse phase LC (5–100% 0.1% formic acid MeCN in H_2O).
Yield: 26 mg, 67%, off-white solid.
^{1}H NMR (400 MHz, DMSO-d_6) δ: 10.61 (s, 1H), 9.95 (s, 1H), 7.95 – 7.88 (m, 2H), 7.87 – 7.83 (m, 2H), 7.33 (t, *J* = 2.2 Hz, 1H), 7.23 (t, *J* = 8.1 Hz, 1H), 7.17 (ddd, *J* = 8.1, 1.9, 1.1 Hz, 1H), 6.66 (ddd, *J* = 8.2, 2.6, 1.1 Hz, 1H), 6.47 (dd, *J* = 17.0, 10.0 Hz, 1H), 6.33 (dd, *J* = 16.9, 2.0 Hz, 1H), 5.88 – 5.81 (m, 1H), 4.18 (t, *J* = 6.1 Hz, 1H), 3.74 (s, 3H), 3.50 – 3.43 (m, 1H), 3.23 (dt, *J* = 10.1, 6.0 Hz, 1H), 1.87 (ddd, *J* = 5.8, 4.2, 1.9 Hz, 2H), 1.58 – 1.47 (m, 1H), 1.19 (d, *J* = 6.6 Hz, 1H) ppm.
^{13}C NMR (101 MHz, DMSO-d_6) δ: 170.5, 164.1, 143.6, 140.3, 131.8, 129.9, 129.0, 128.5, 119.6, 112.1, 109.6, 105.5, 62.3, 55.4, 49.6, 31.5, 24.7 ppm.
LRMS (+ESI) *m/z*: $[M+H]^+$ 431.
HRMS (+ESI) *m/z*: $[M+H]^+$ calcd for $C_{21}H_{24}N_3O_5S$ 430.1431, found 430.1431.

HPLC retention time 3.9 min, >99.0% (Method A).

1-((2-Methoxy-4-nitrophenyl)sulfonyl)pyrrolidine (39)

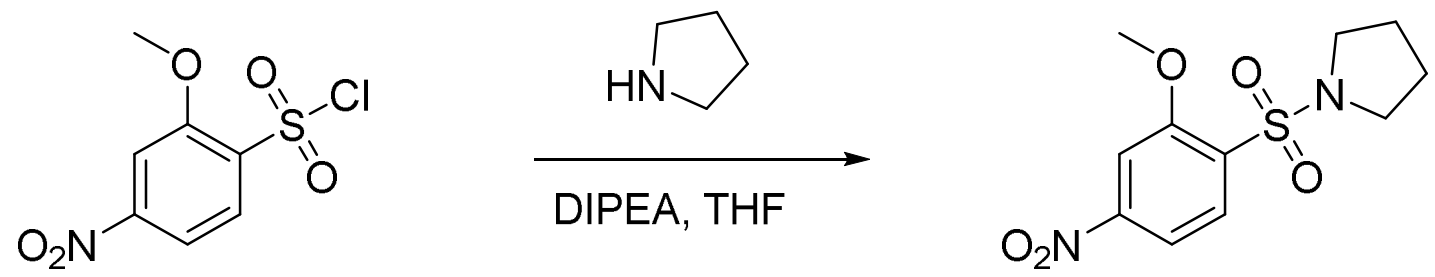

General procedure A, reagents: pyrrolidine (130 µL, 1.6 mmol); 2-methoxy-4-nitrophenylsulfonyl chloride (350 mg, 1.6 mmol); DIPEA (550 µL, 3.2 mmol); THF (4.0 mL).
Yield: 390 mg, 85%, orange solid.
^{1}H NMR (400 MHz, Chloroform-*d*) δ: 8.15 (d, *J* = 8.5 Hz, 1H), 7.92 (dd, *J* = 8.5, 2.1 Hz, 1H), 7.87 (d, *J* = 2.1 Hz, 1H), 4.08 (s, 3H), 3.51 – 3.36 (m, 4H), 1.98 – 1.81 (m, 4H) ppm.
^{13}C NMR (101 MHz, Chloroform-*d*) δ: 157.3, 151.2, 133.3, 132.8, 115.2, 107.3, 56.8, 47.9, 25.9 ppm.

3-Methoxy-4-(pyrrolidin-1-ylsulfonyl)aniline (40)

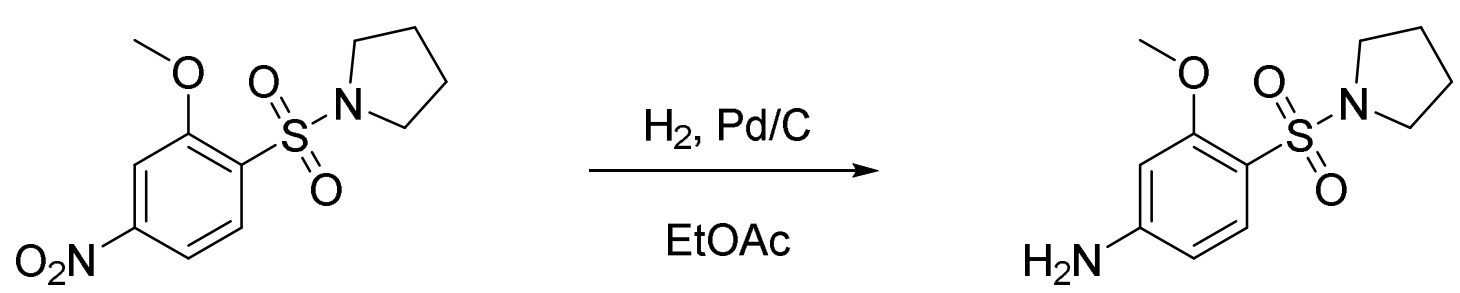

General procedure B, reagents: nitrosulfonamide **39** (380 mg, 1.3 mmol); H_2 (1 atm); 10% Pd/C (38 mg, 10% w/w); EtOAc (20 mL).
Yield: 340 mg, quantitative, brown solid.
^{1}H NMR (400 MHz, Chloroform-*d*) δ: 7.36 (d, *J* = 8.6 Hz, 1H), 6.25 (d, *J* = 2.0 Hz, 1H), 6.15 (dd, *J* = 8.6, 2.0 Hz, 1H), 5.99 (s, 2H), 3.16 – 3.10 (m, 4H), 1.72 – 1.63 (m, 4H) ppm.
^{13}C NMR (101 MHz, Chloroform-*d*) δ: 163.4, 160.0, 138.2, 116.6, 109.9, 101.6, 60.4, 52.6, 30.4 ppm.

***N*-(3-Methoxy-4-(pyrrolidin-1-ylsulfonyl)phenyl)acrylamide (14, IMP1721)**

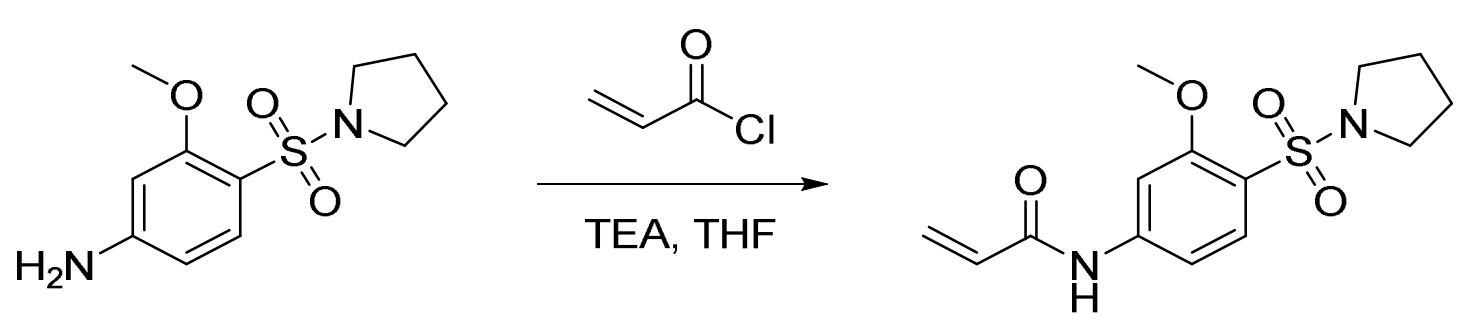

General procedure C, reagents: aniline **40** (100 mg, 0.39 mmol); acryloyl chloride (50 µL, 0.59 mmol); Et_3N (90 µL, 0.65 mmol); THF (2.0 mL); *purification:* LC (20–80% EtOAc in hexane).
Yield: 81 mg, 67%, yellow vetrous solid.
^{1}H NMR (400 MHz, DMSO-d_6) δ: 10.53 (s, 1H), 7.71 (d, *J* = 8.6 Hz, 1H), 7.67 (d, *J* = 1.9 Hz, 1H), 7.31 (dd, *J* = 8.6, 1.9 Hz, 1H), 6.45 (dd, *J* = 17.0, 10.0 Hz, 1H), 6.32 (dd, *J* = 17.0, 2.0 Hz, 1H), 5.84 (dd, *J* = 10.0, 2.0 Hz, 1H), 3.87 (s, 3H), 3.27 – 3.15 (m, 4H), 1.80 – 1.67 (m, 4H) ppm.
^{13}C NMR (101 MHz, DMSO-d_6) δ: 164.2, 157.5, 145.0, 132.7, 131.9, 128.6, 120.8, 110.8, 103.4, 56.2, 48.0, 25.67 ppm.
HRMS (ESI) *m/z*: $[M+H]^+$ calcd for $C_{14}H_{19}N_2O_4S$: 311.1066; found: 311.1052.

NMR Figures

***N*-(4-(Pyrrolidin-1-ylsulfonyl)phenyl)acrylamide (1, IMP-1704)**

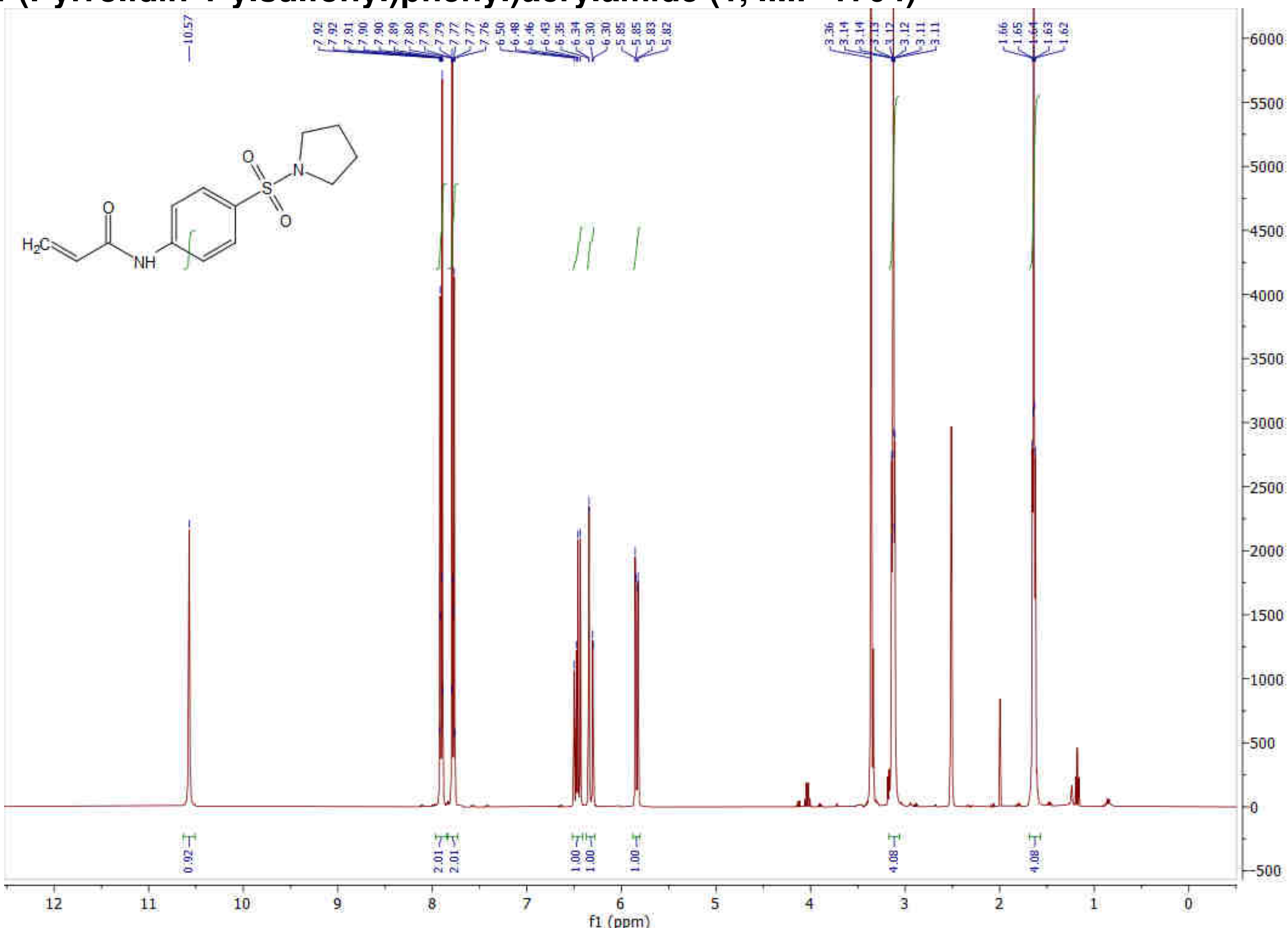

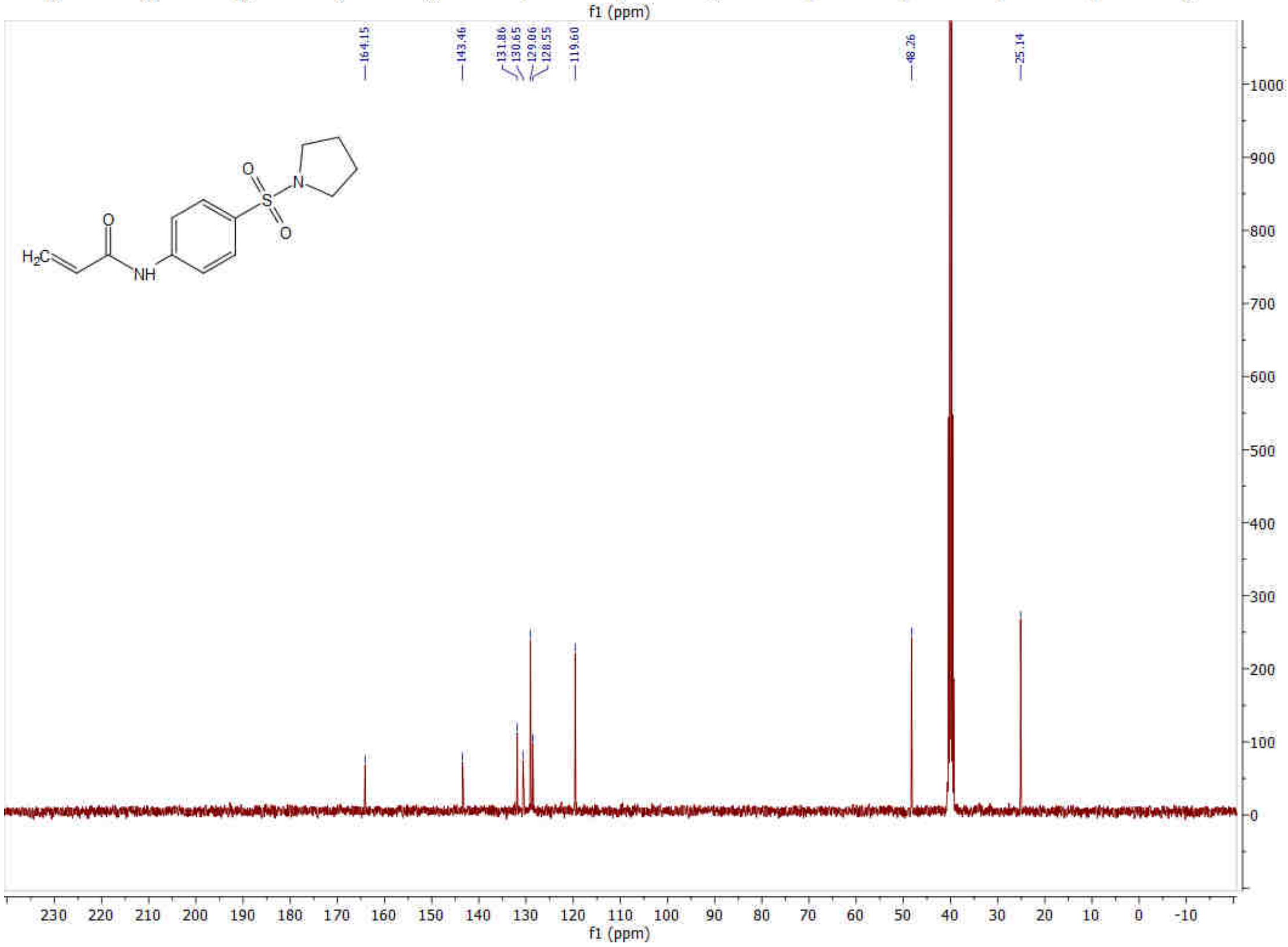

N-(4-(Pyrrolidine-1-carbonyl)phenyl)acrylamide (2)

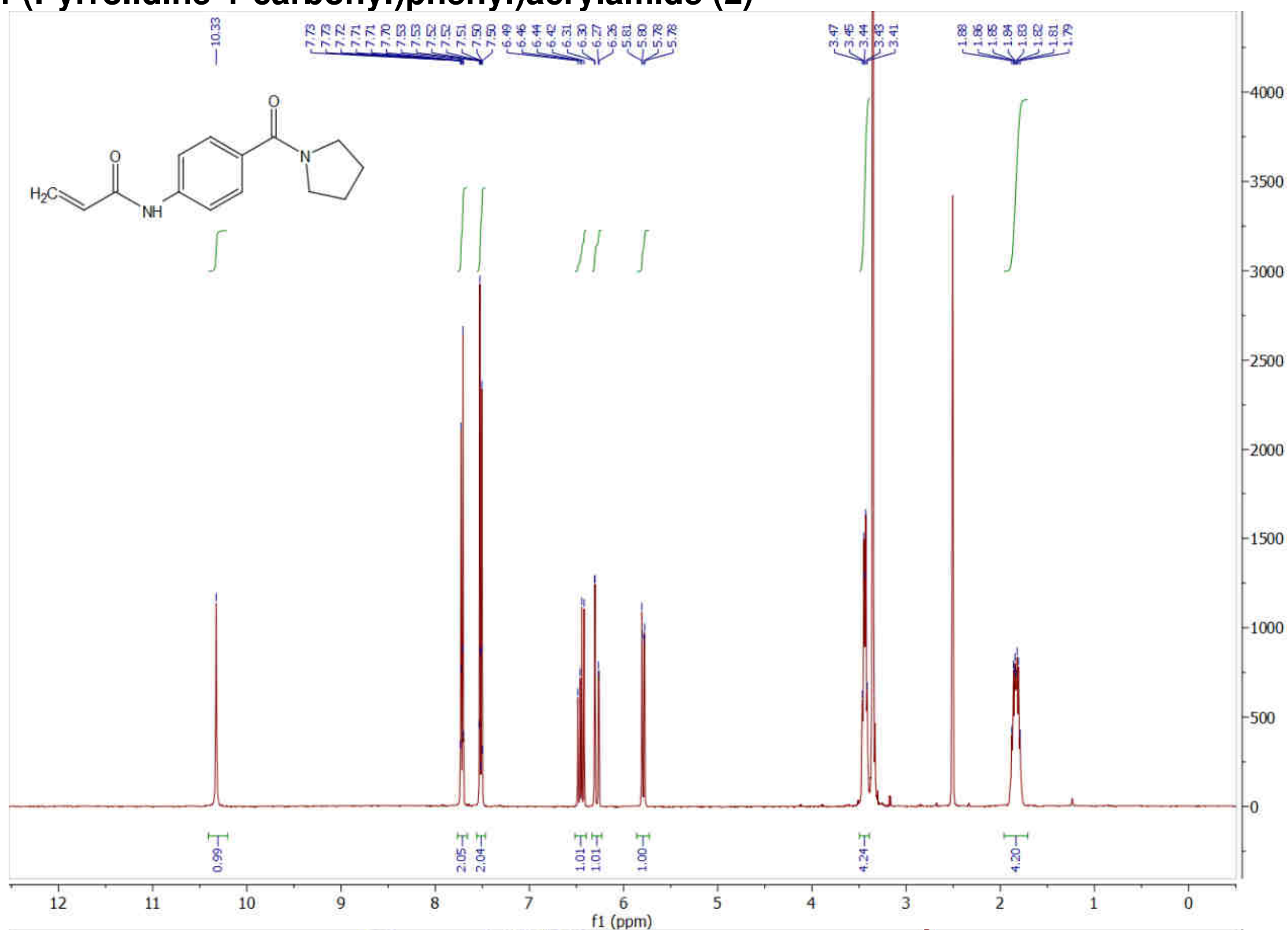

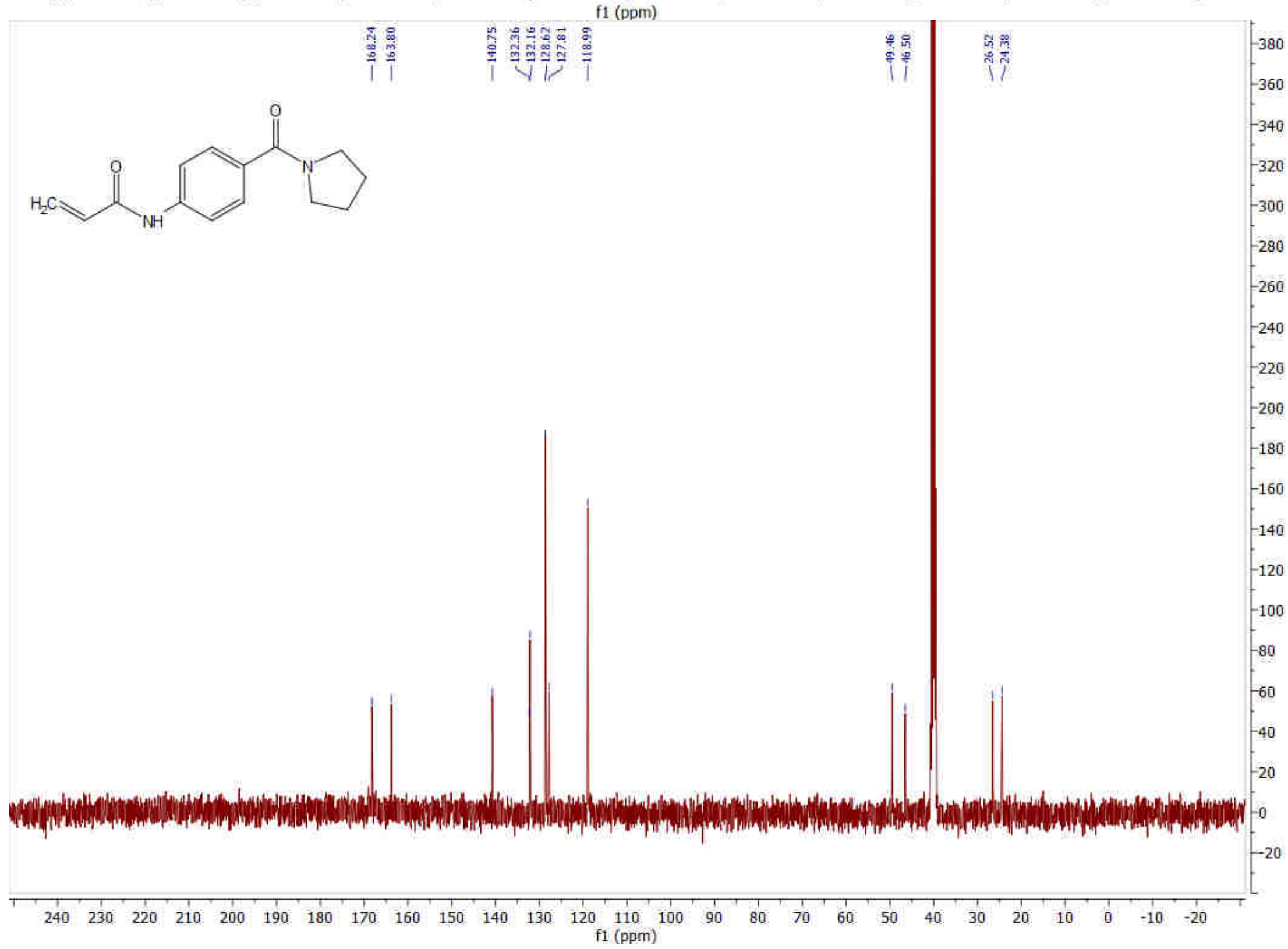

***N*-(3-(Pyrrolidin-1-ylsulfonyl)phenyl)acrylamide (3)**

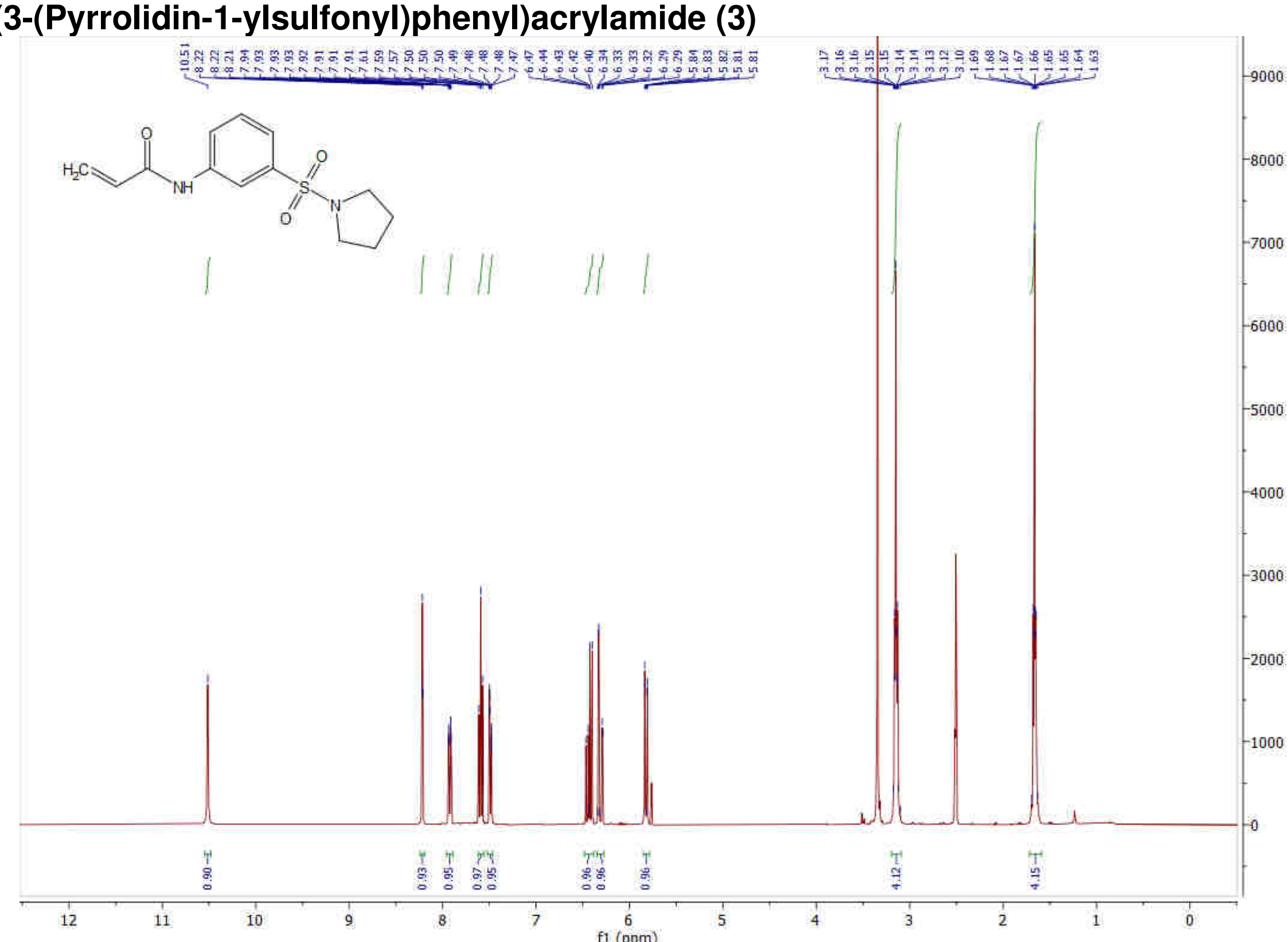

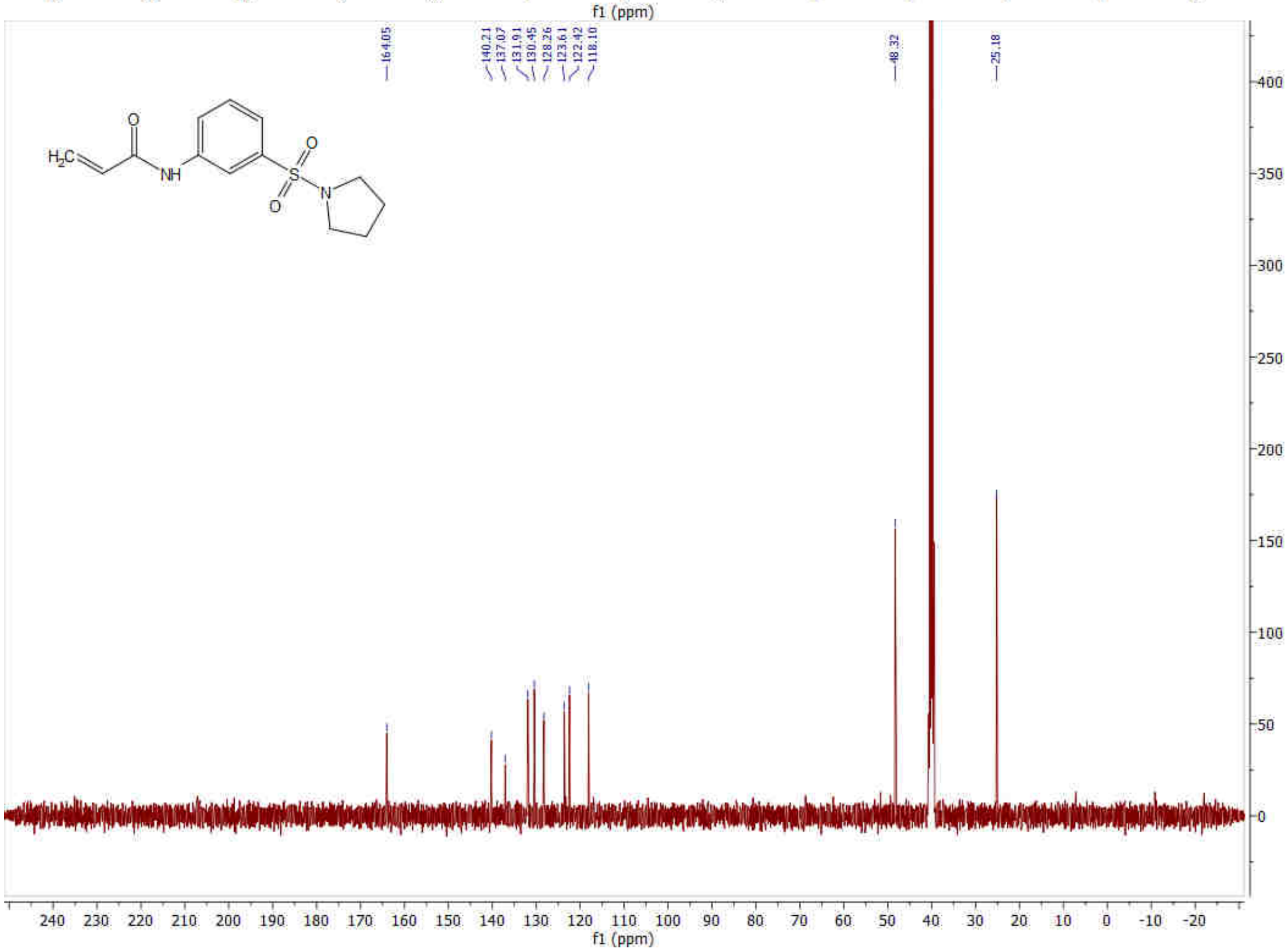

Methyl ((4-acrylamidophenyl)sulfonyl)-*L*-prolinate (4, IMP1712)

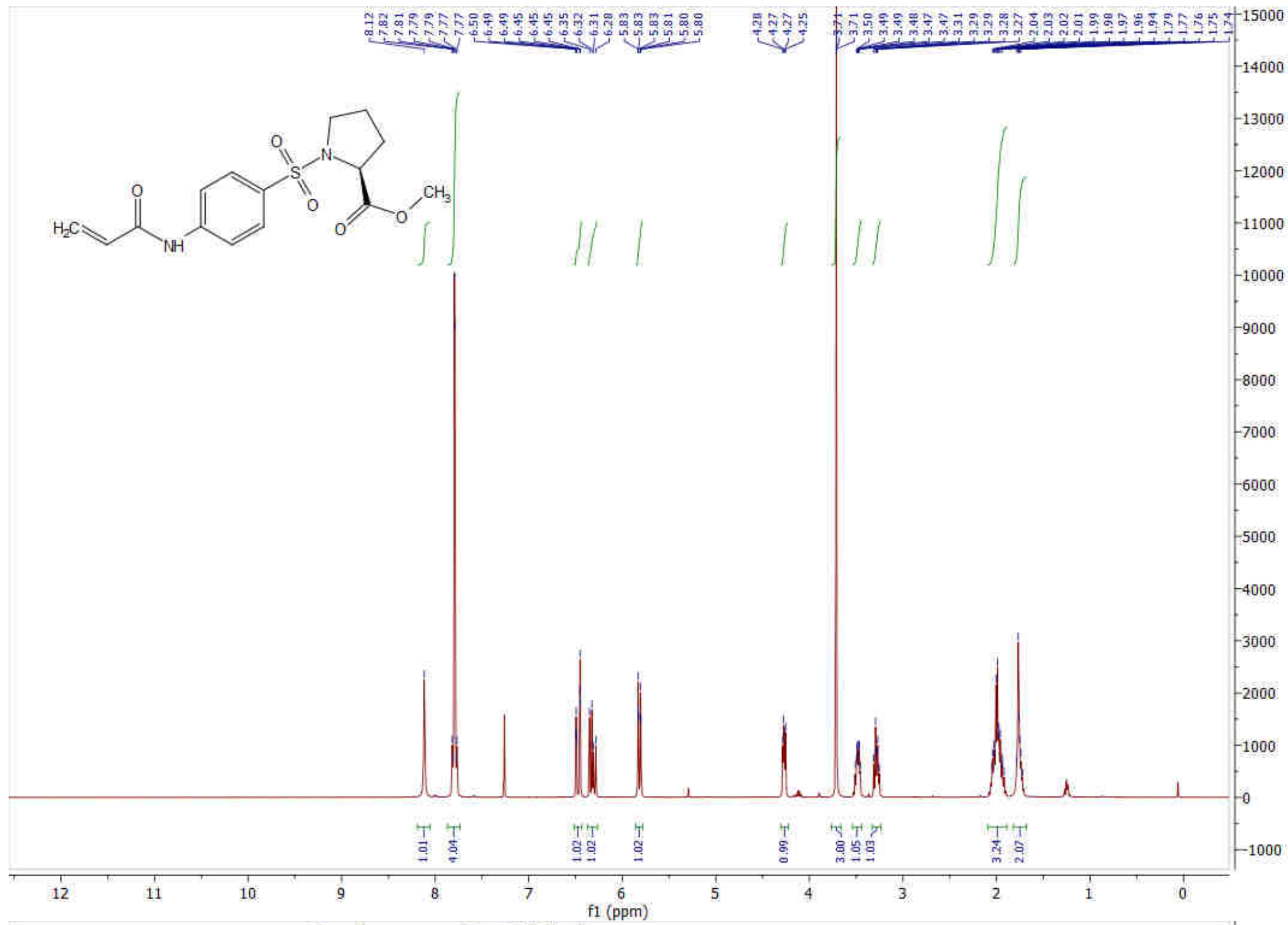

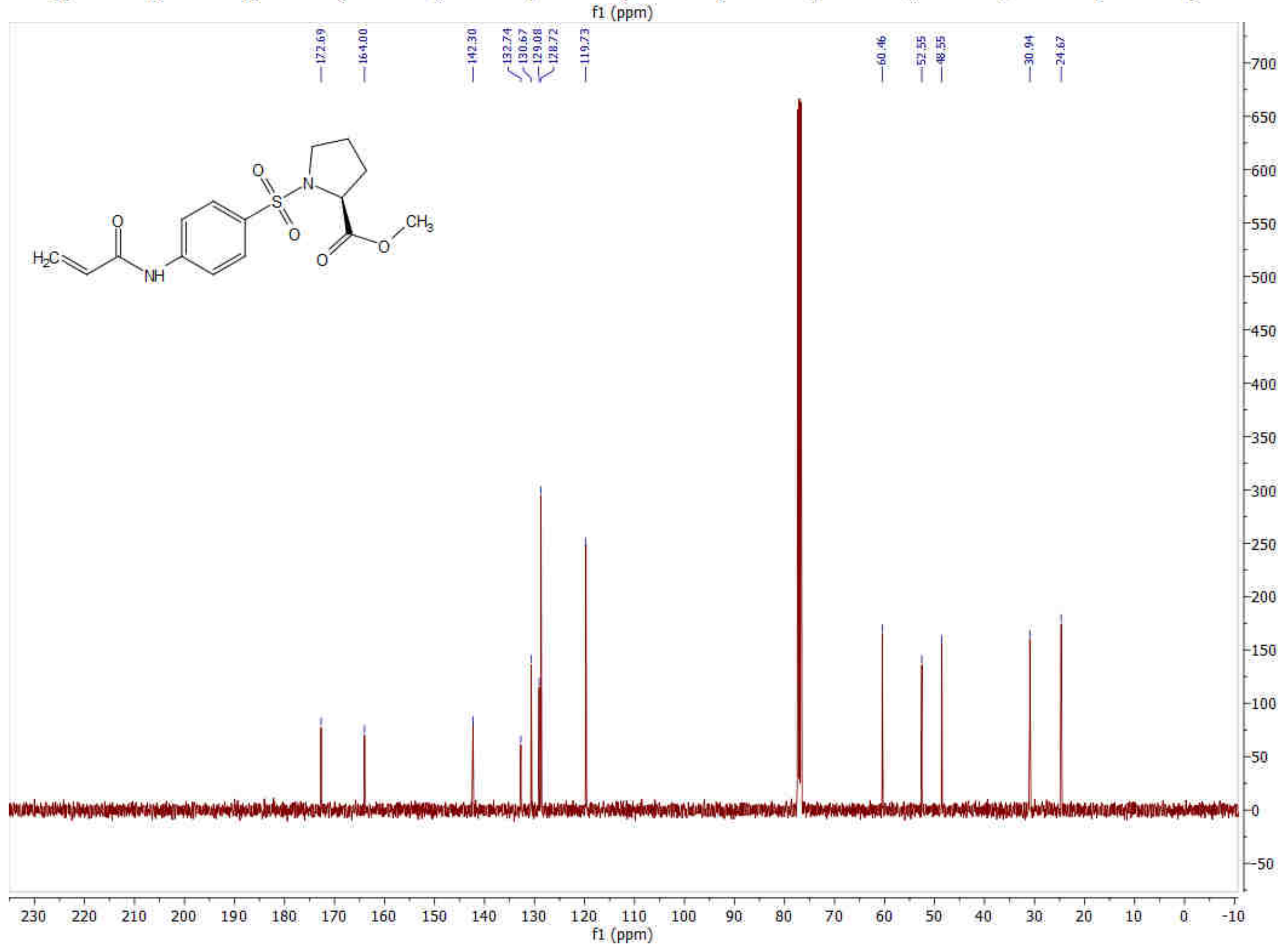

Methyl ((4-acrylamidophenyl)sulfonyl)-*D*-prolinate (5)

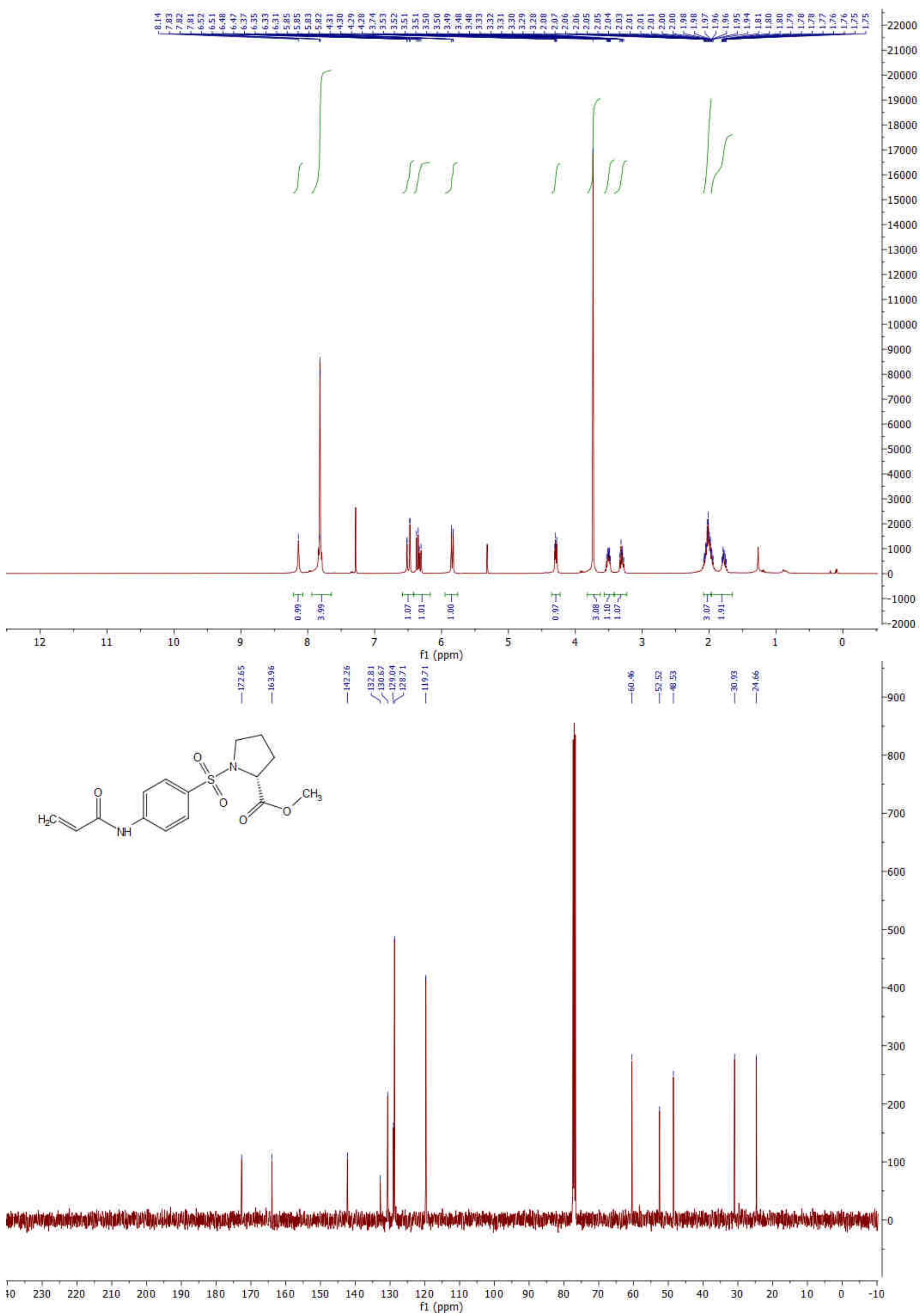

(*S*)-1-((4-Acrylamidophenyl)sulfonyl)-*N*-(prop-2-yn-1-yl)pyrrolidine-2-carboxamide (6)

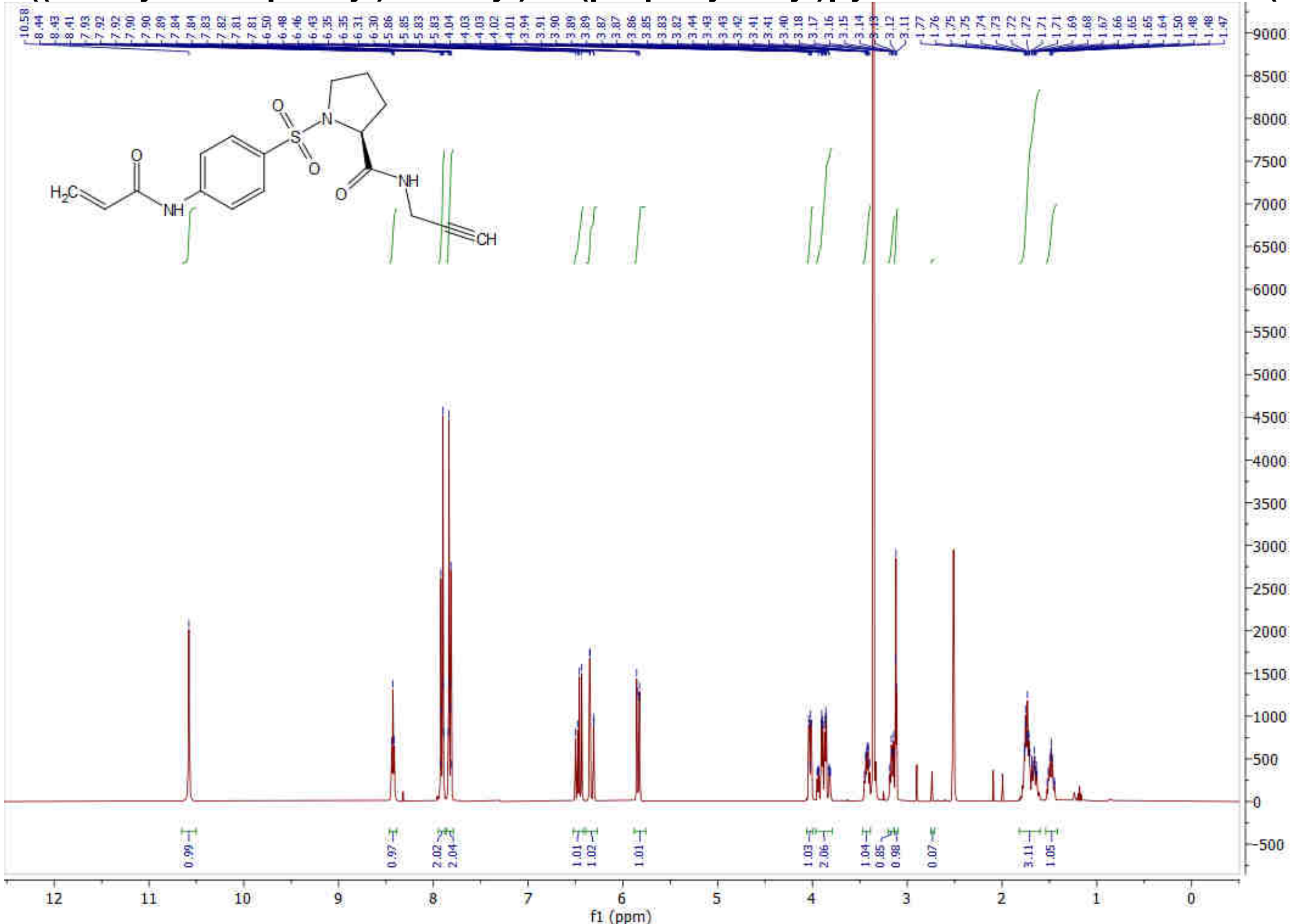

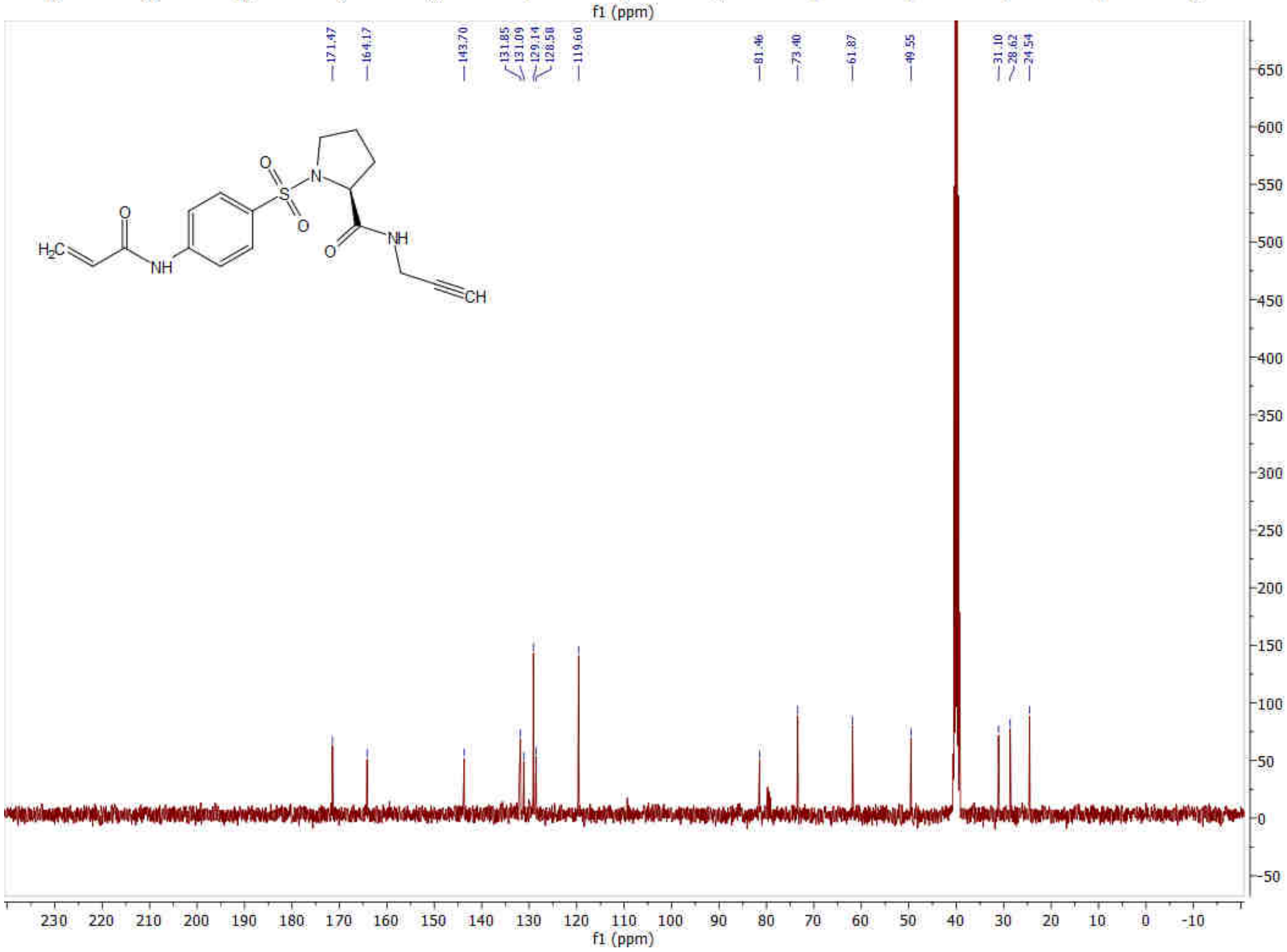

(*S*)-1-((4-Acrylamidophenyl)sulfonyl)-*N*-(4-methoxyphenyl)pyrrolidine-2-carboxamide (7)

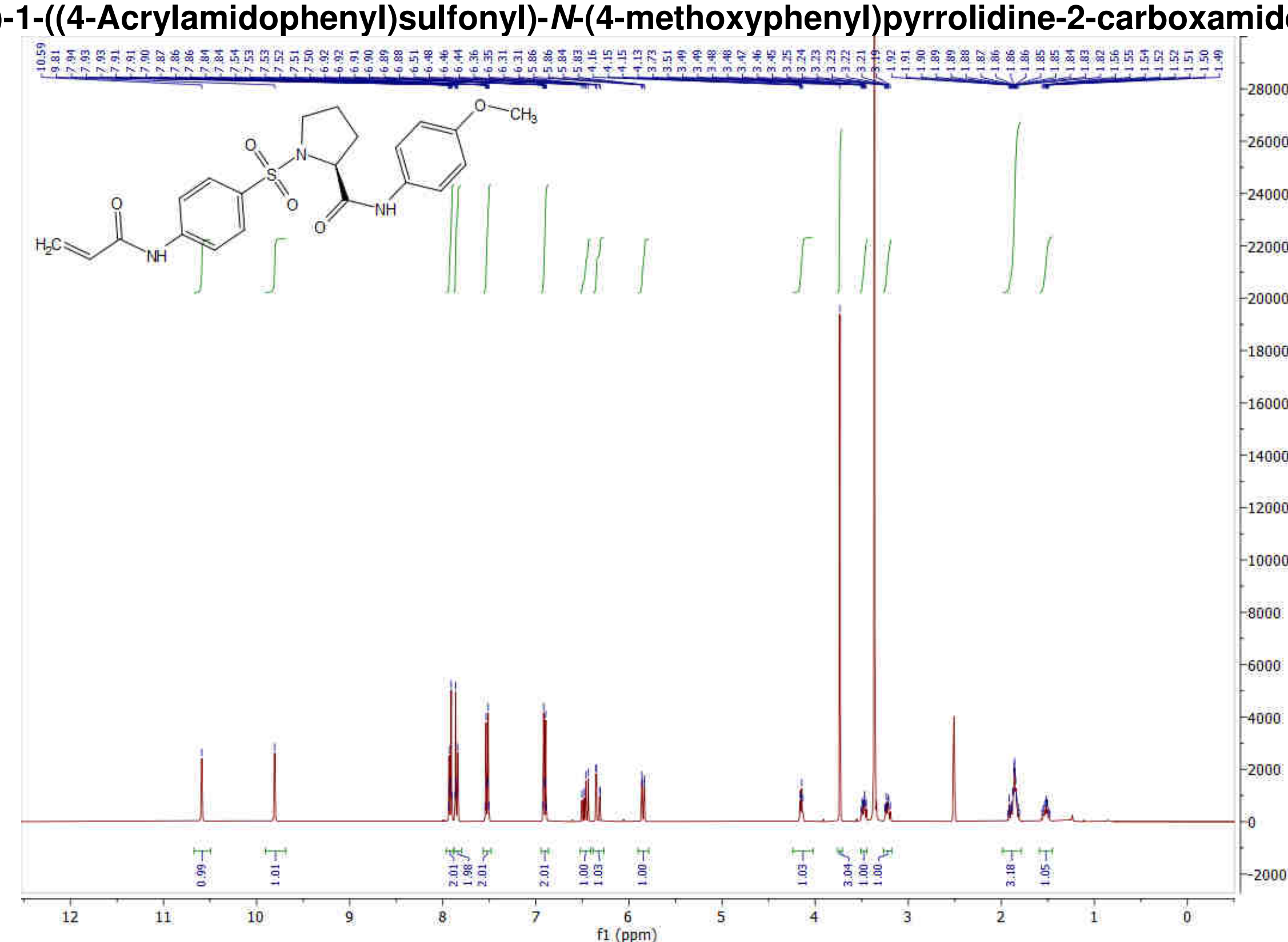

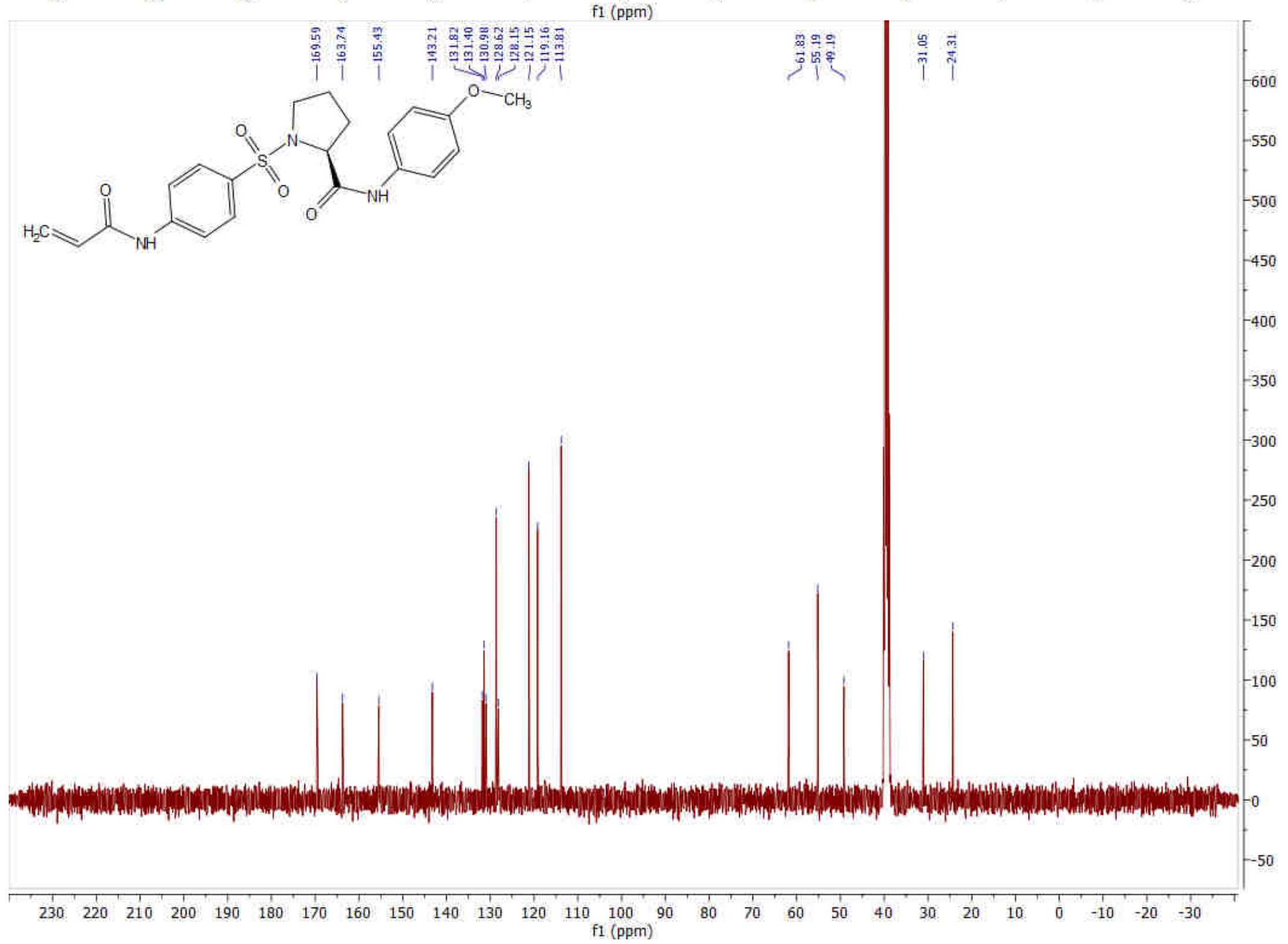

(*R*)-1-((4-Acrylamidophenyl)sulfonyl)-*N*-(4-methoxyphenyl)pyrrolidine-2-carboxamide (8)

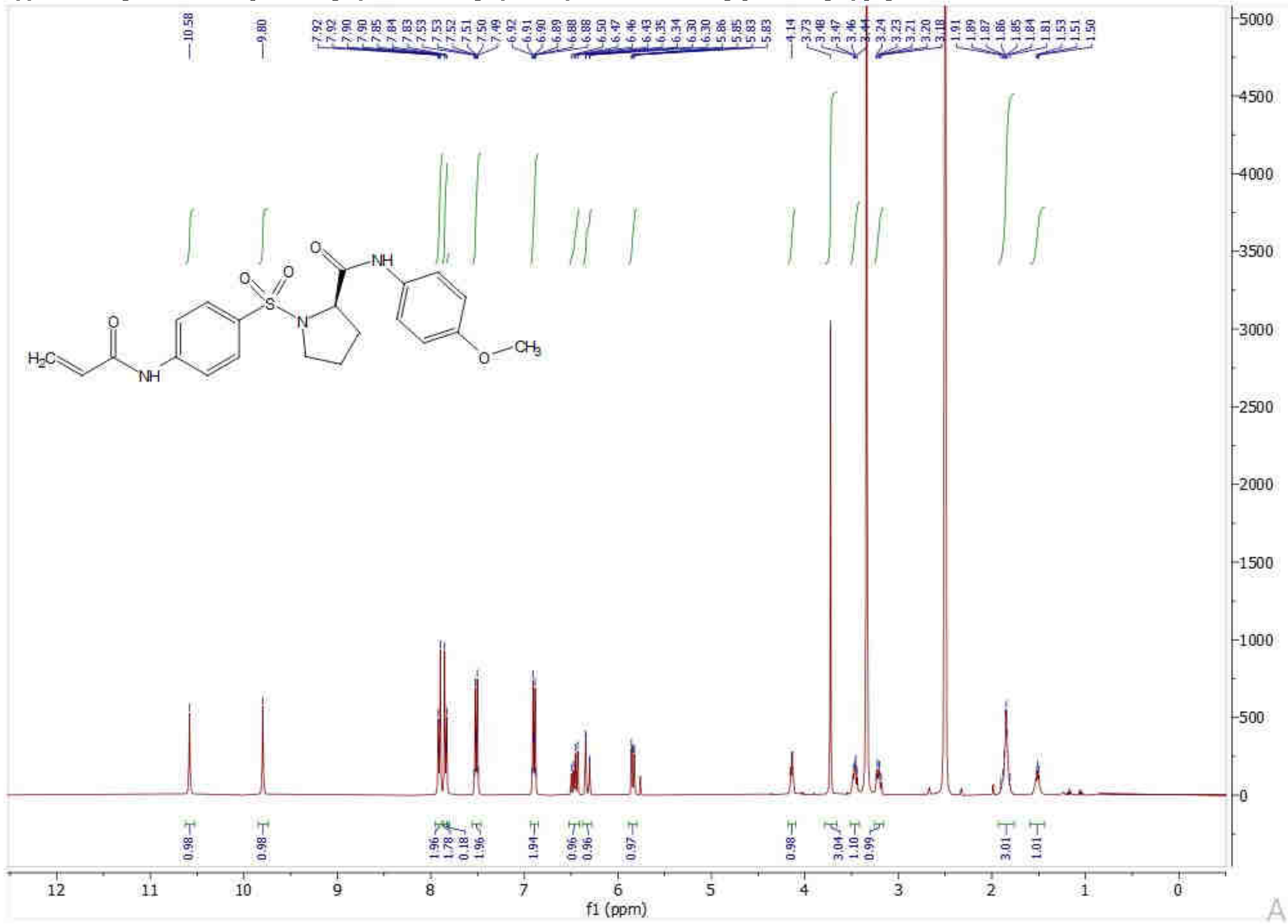

(*S*)-1-((4-Acrylamidophenyl)sulfonyl)-*N*-(4-(prop-2-yn-1-yloxy)phenyl)pyrrolidine-2-carboxamide (9)

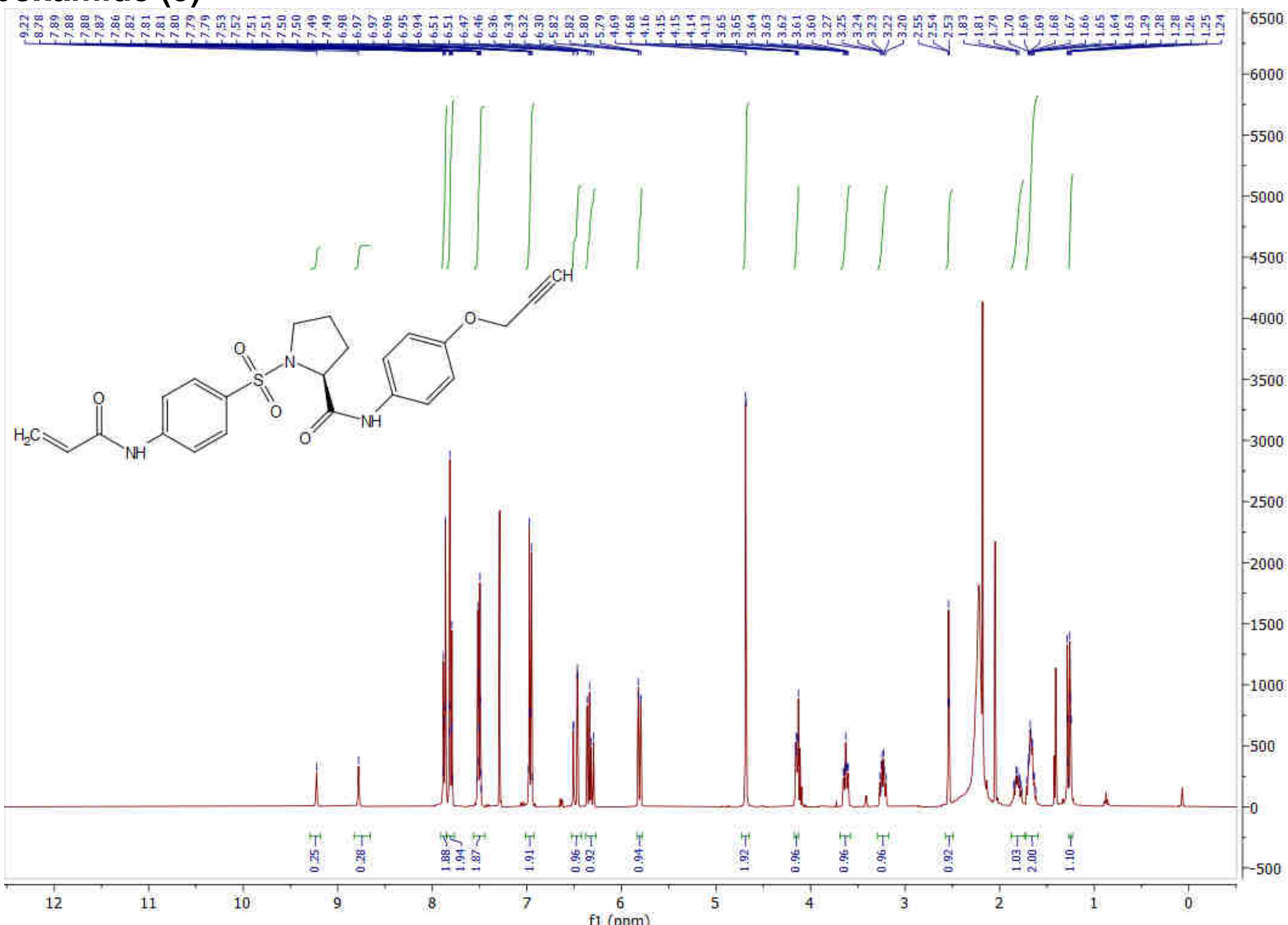

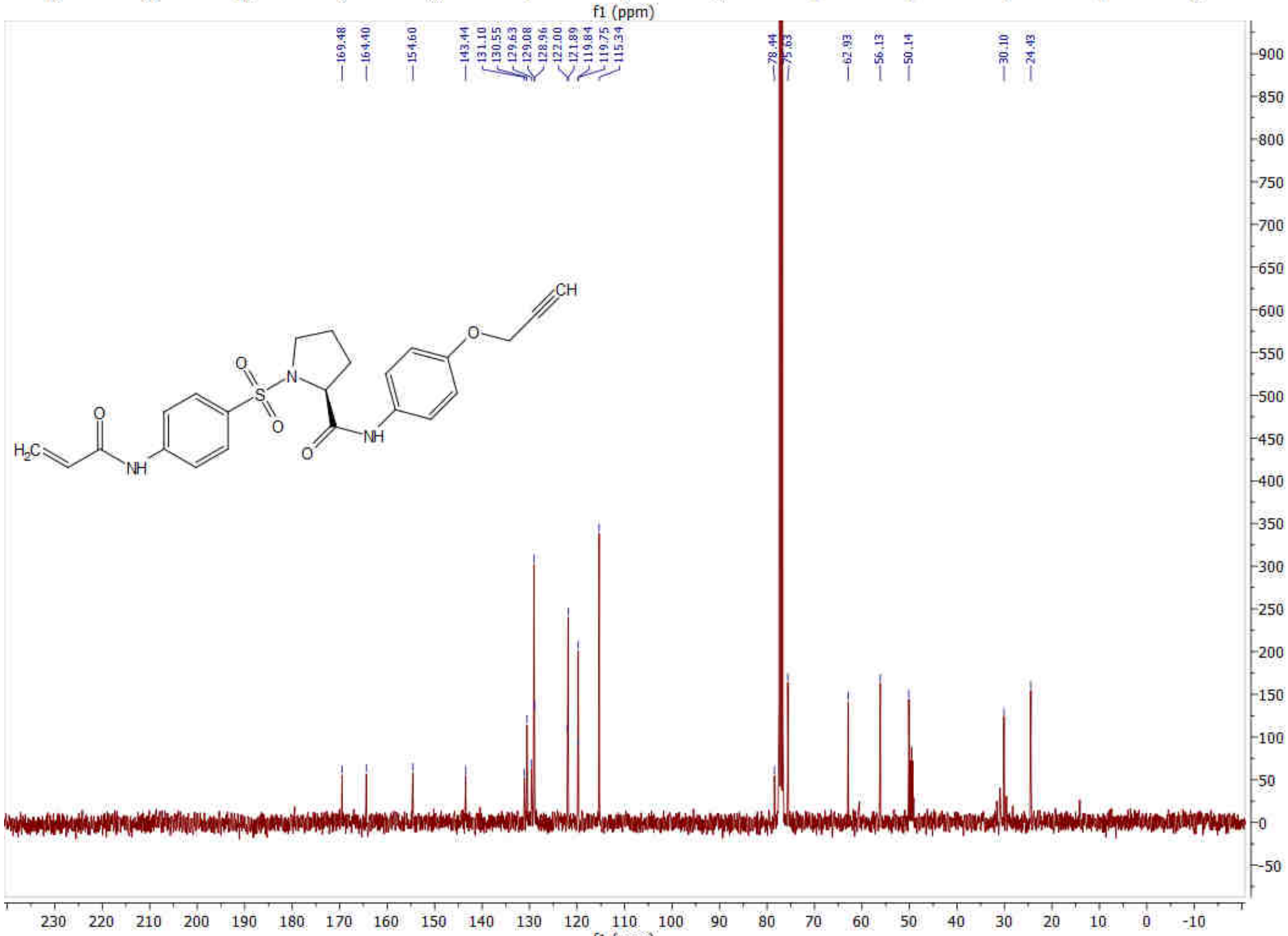

(*S*)-1-((4-Acrylamidophenyl)sulfonyl)-*N*-(4-((1-methyl-1H-pyrazol-3-yl)carbamoyl)phenyl)pyrrolidine-2-carboxamide (10, IMP-2552)

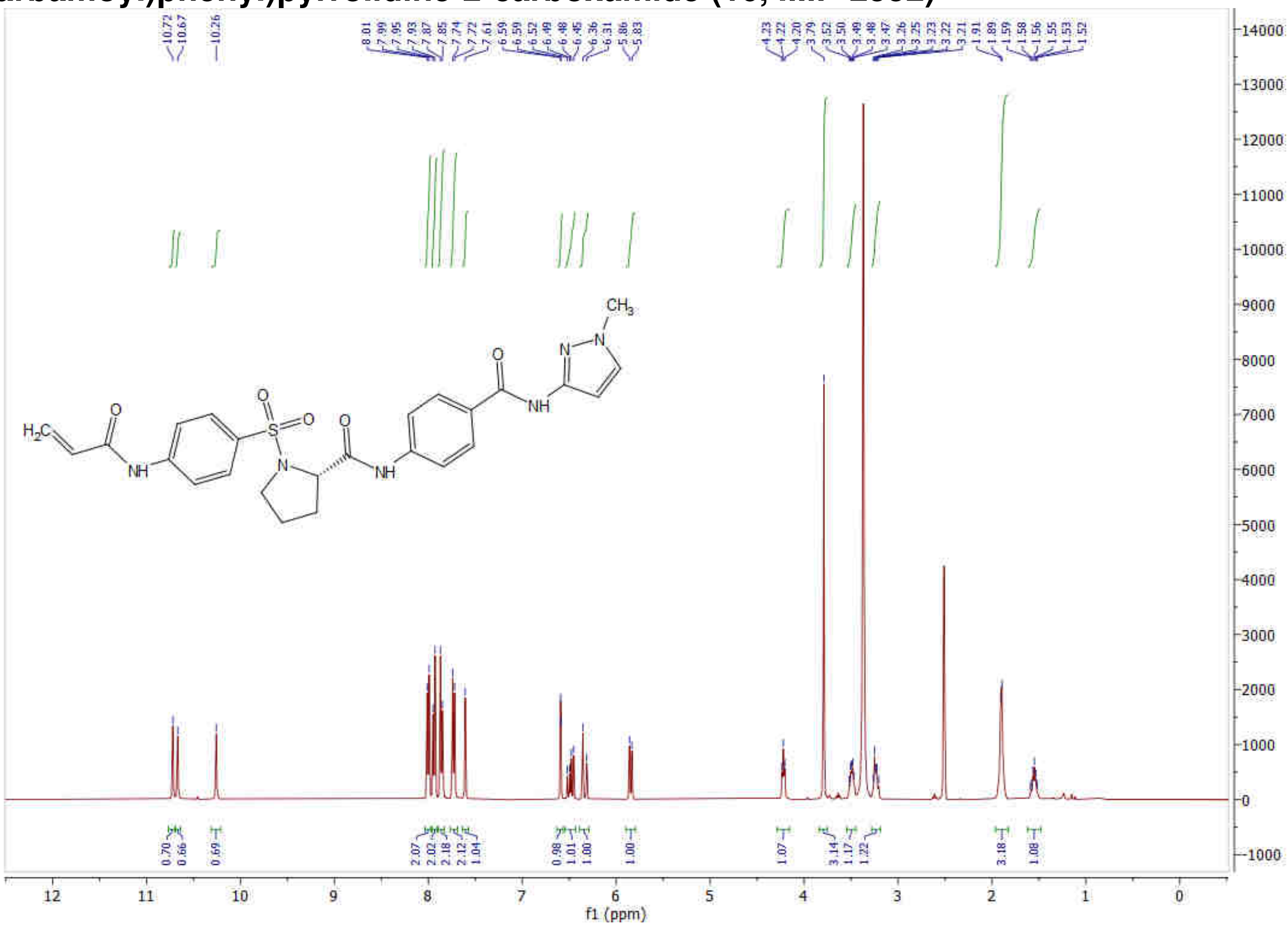

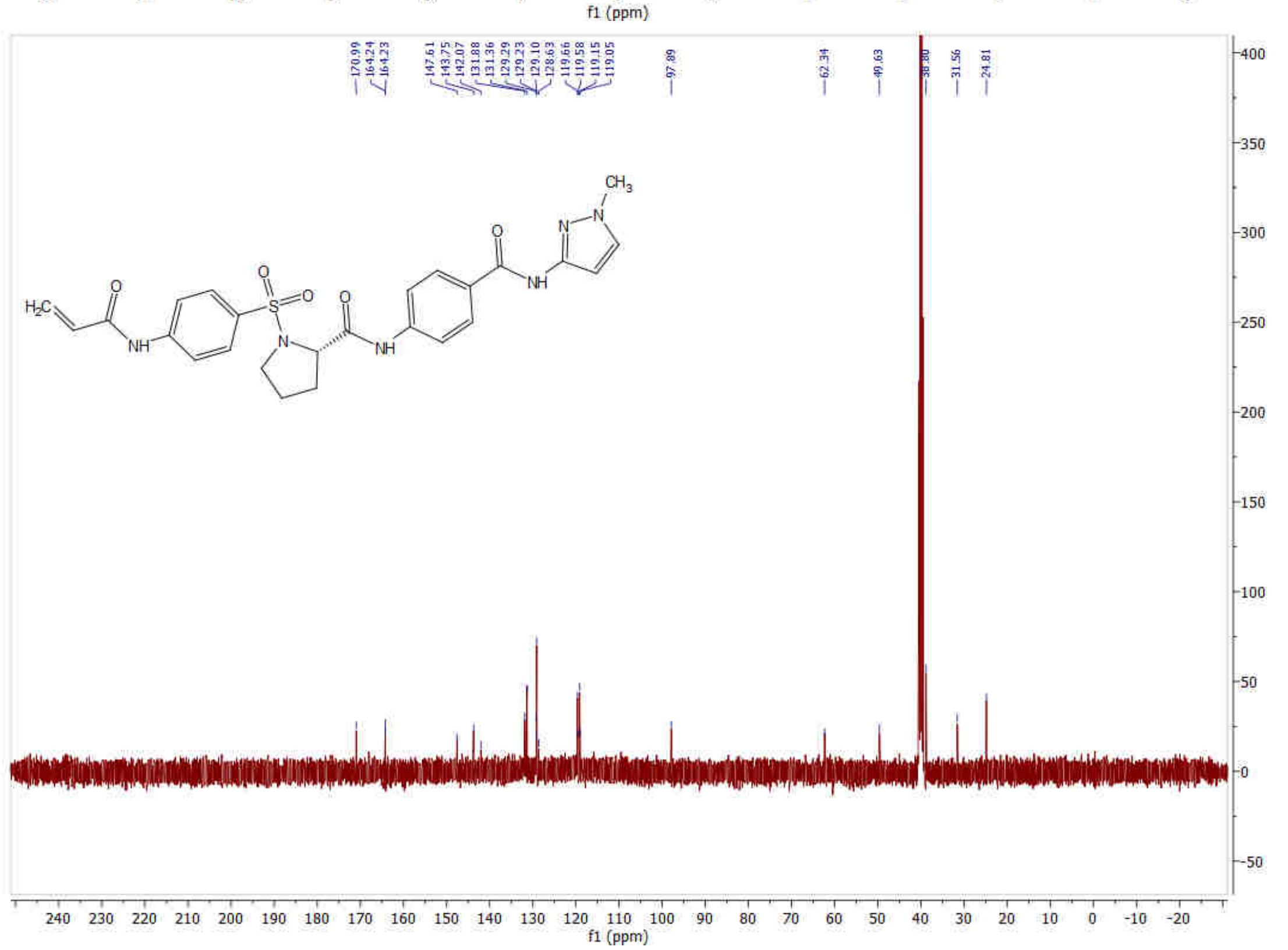

(*R*)-1-((4-Acrylamidophenyl)sulfonyl)-*N*-(4-((1-methyl-1H-pyrazol-3-yl)carbamoyl)phenyl)pyrrolidine-2-carboxamide (11)

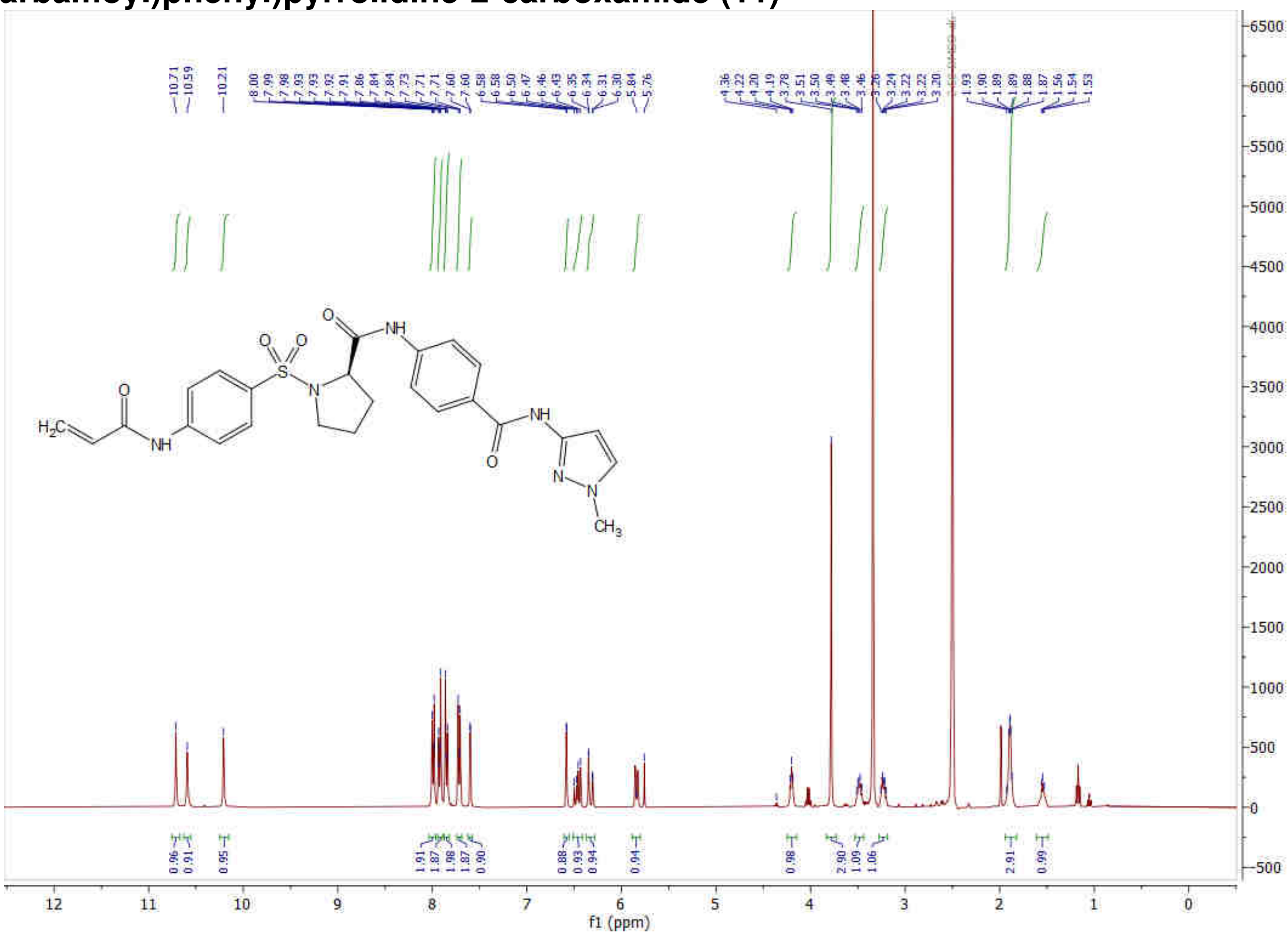

(*S*)-1-((4-Acrylamidophenyl)sulfonyl)-*N*-(4-((1-(prop-2-yn-1-yl)-1H-pyrazol-4-yl)carbamoyl)phenyl)pyrrolidine-2-carboxamide (12, IMP-2566)

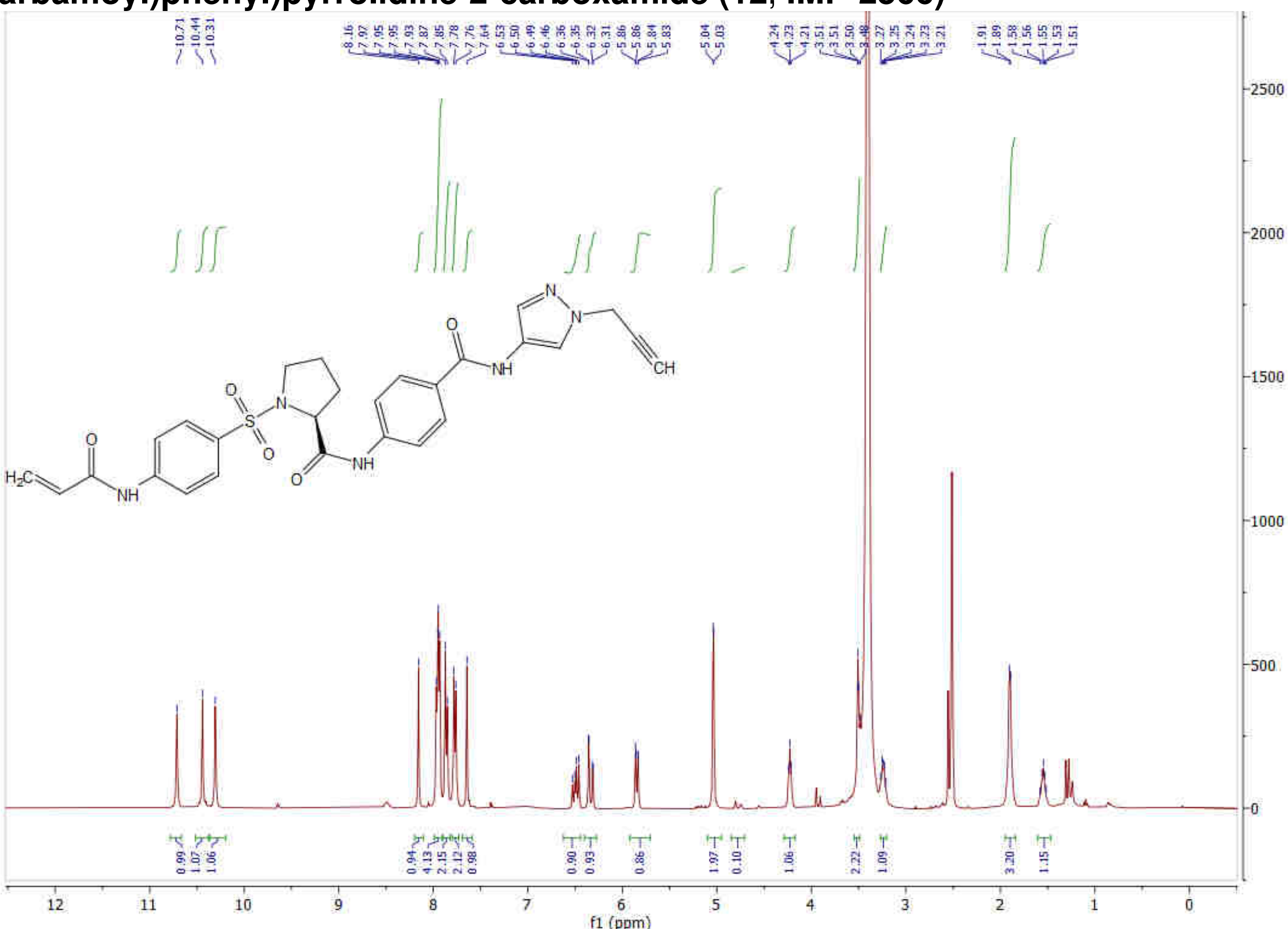

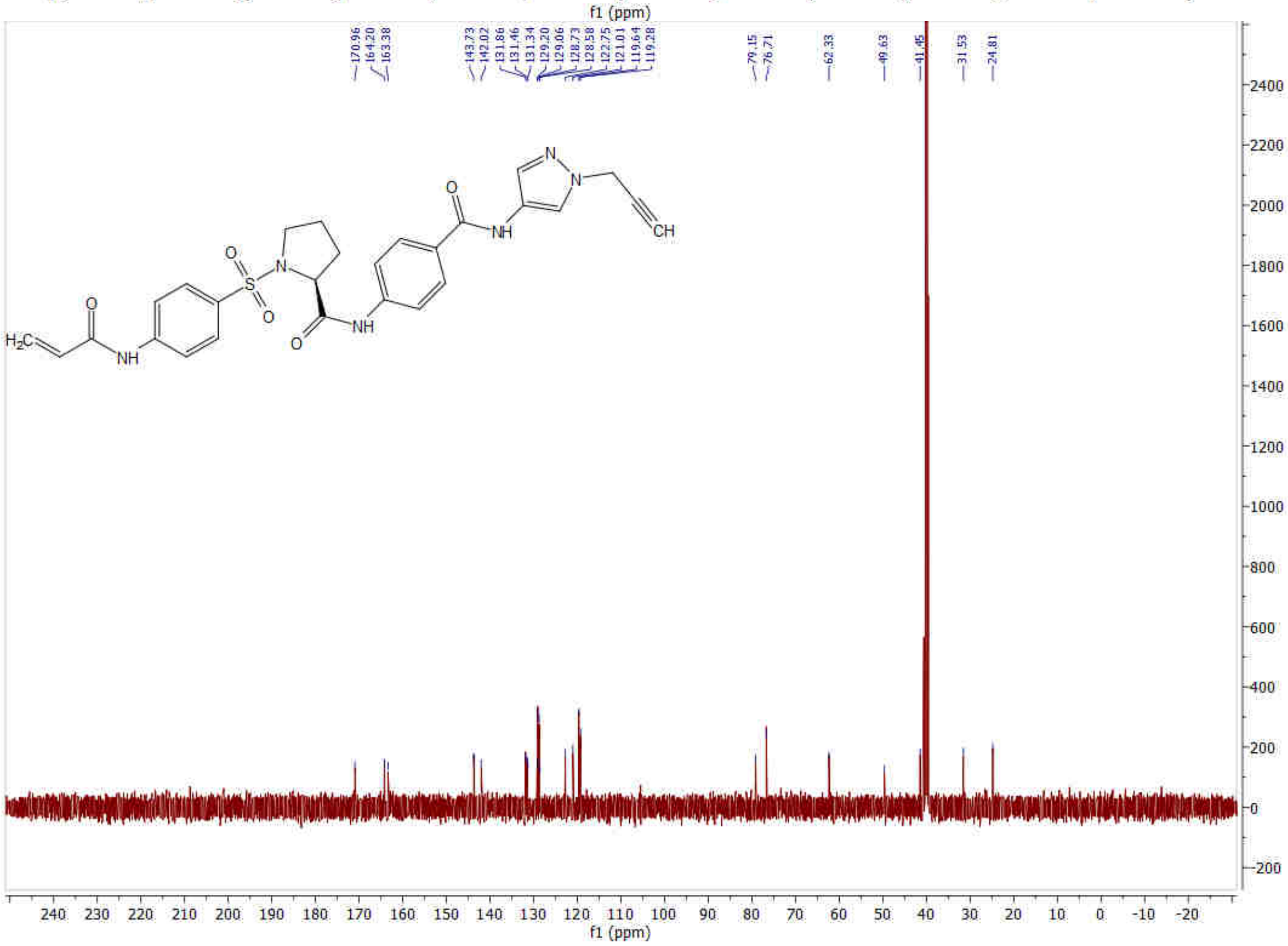

(*S*)-1-((4-Acrylamidophenyl)sulfonyl)-*N*-(3-methoxyphenyl)pyrrolidine-2-carboxamide (13, IMP-2505)

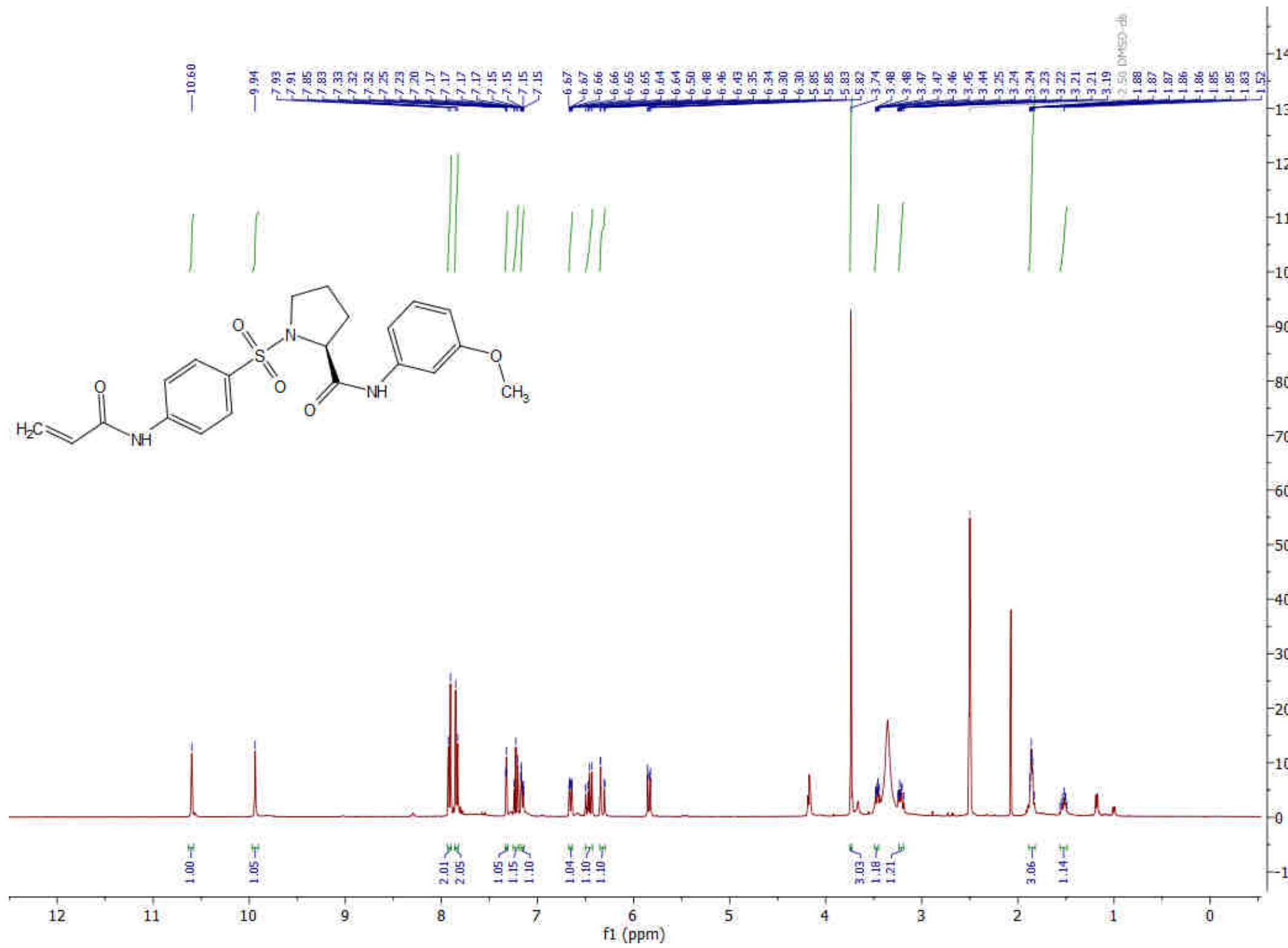

HPLC Traces

***N*-(4-(Pyrrolidin-1-ylsulfonyl)phenyl)acrylamide (1, IMP-1704)**

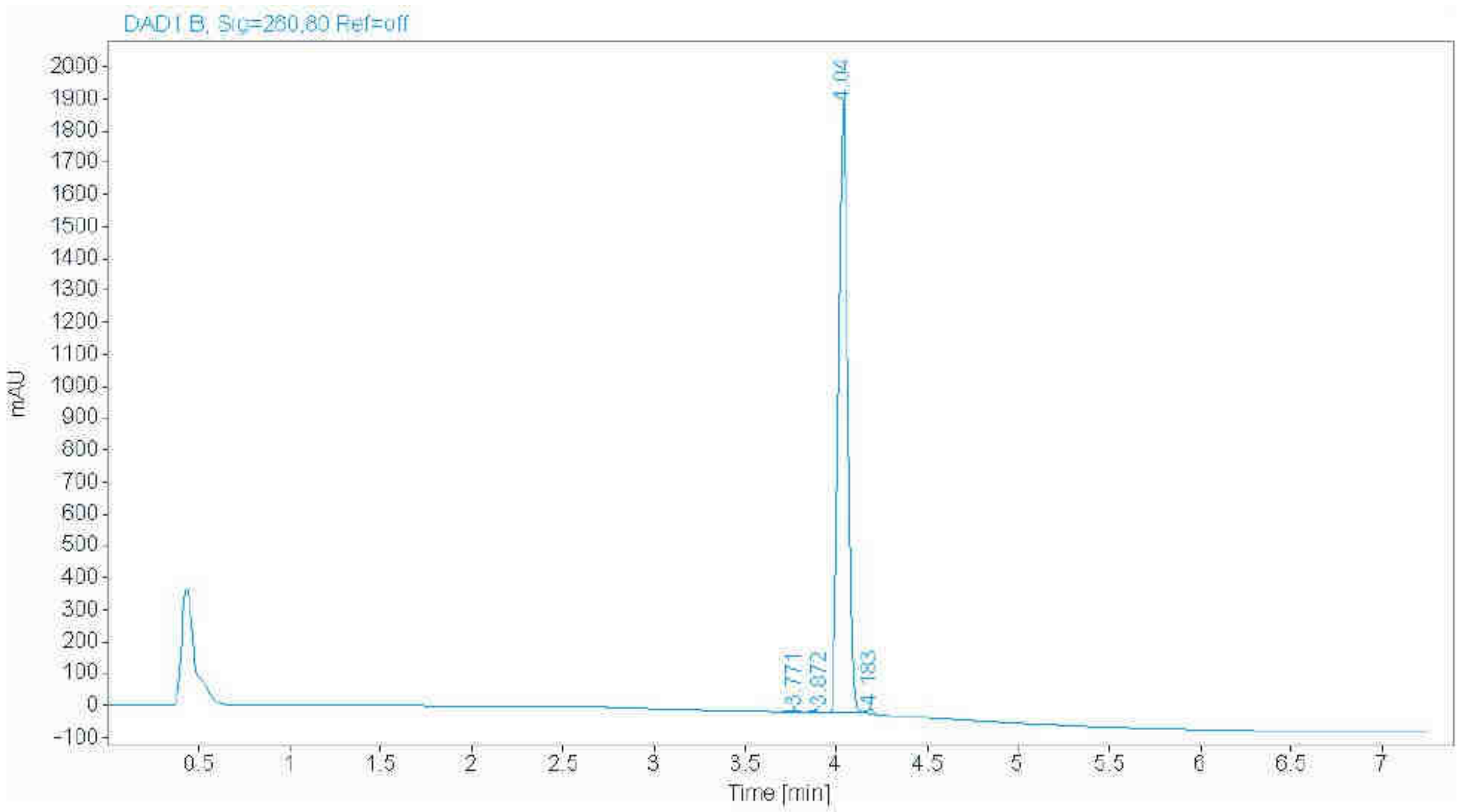

RT [min]	Width [min]	Area	Height	Area%
3.771	0.0572	12.8330	3.4617	0.1859
3.872	0.0503	9.9700	3.2077	0.1444
4.040	0.0577	6848.6528	1913.8254	99.2228
4.183	0.0429	30.8401	11.6413	0.4468
	Sum	6902.2959		

***N*-(4-(Pyrrolidine-1-carbonyl)phenyl)acrylamide (2)**

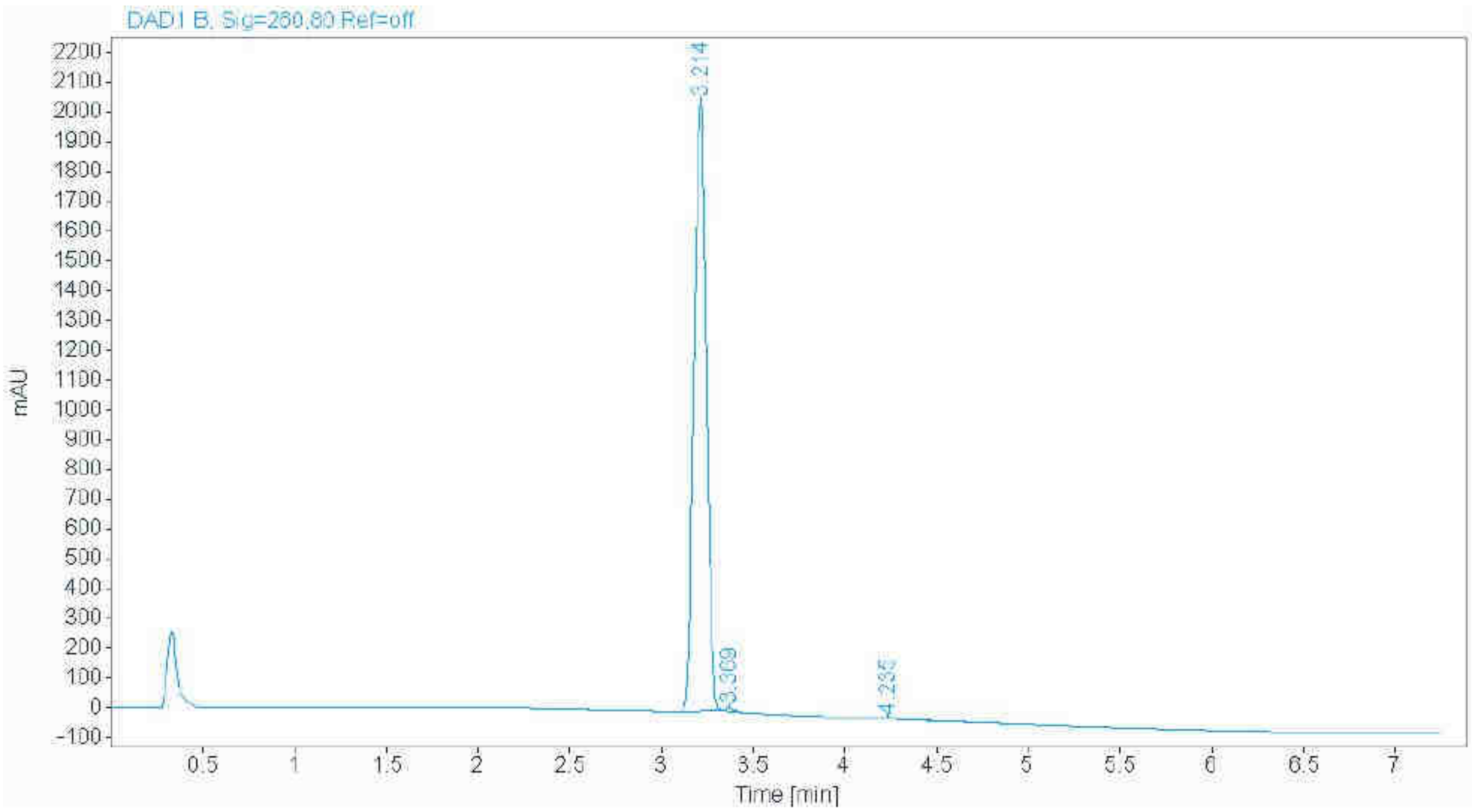

RT [min]	Width [min]	Area	Height	Area%
3.214	0.0750	9658.4990	2049.9414	99.4610
3.369	0.0475	29.8351	9.8111	0.3072
4.235	0.1254	22.5057	2.3158	0.2318
	Sum	9710.8398		

***N*-(3-(Pyrrolidin-1-ylsulfonyl)phenyl)acrylamide (3)**

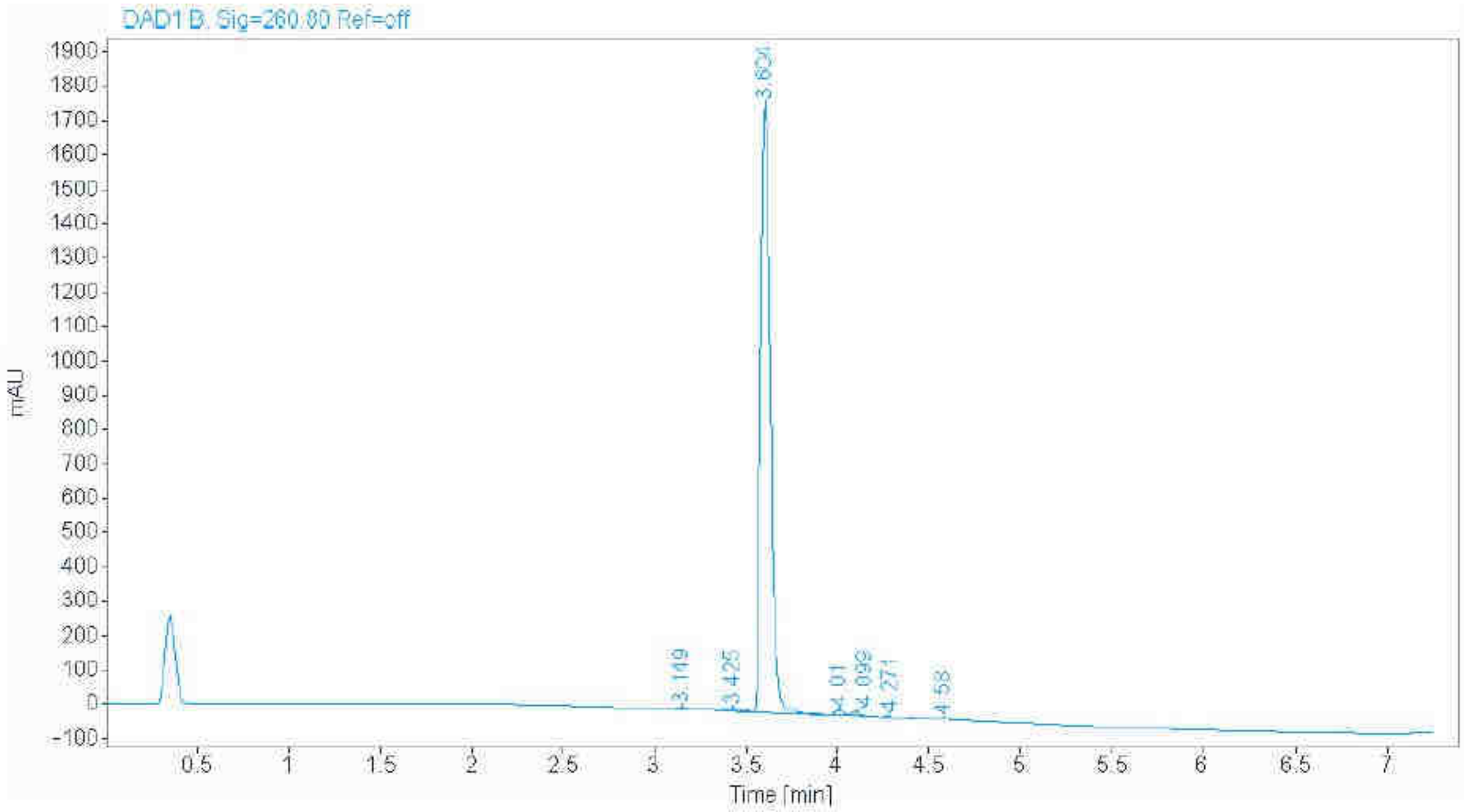

RT [min]	Width [min]	Area	Height	Area%
3.149	0.0594	10.3622	2.5528	0.1588
3.425	0.0845	16.5179	3.1858	0.2531
3.604	0.0539	6406.0527	1782.8141	98.1778
4.010	0.0449	32.4232	11.5093	0.4969
4.099	0.0485	31.4536	10.6448	0.4821
4.271	0.1560	15.8032	1.3554	0.2422
4.580	0.0538	12.3349	3.4389	0.1890
	Sum	6524.9478		

Methyl ((4-acrylamidophenyl)sulfonyl)-*L*-prolinate (4, IMP1712)

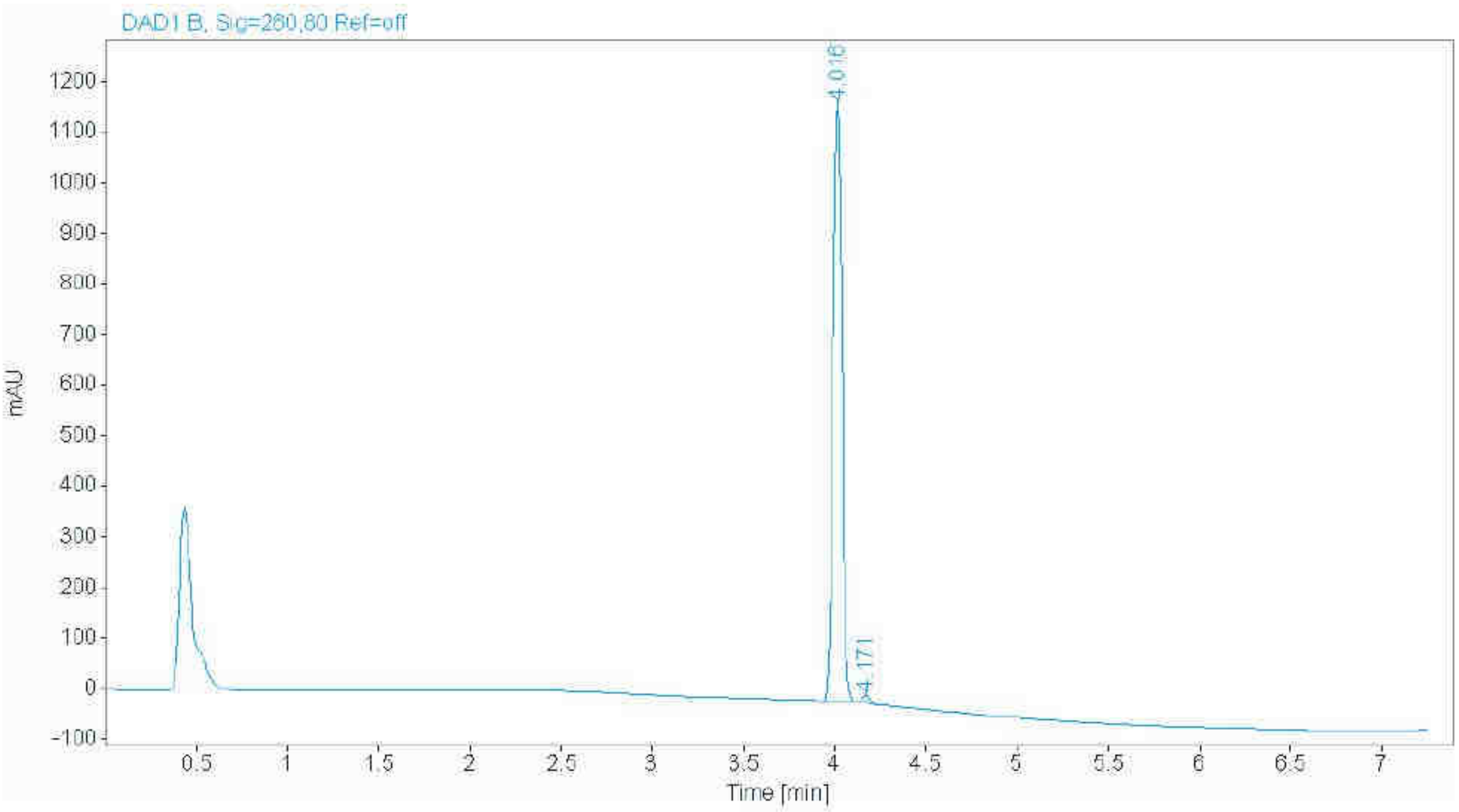

RT [min]	Width [min]	Area	Height	Area%
4.016	0.0529	3951.4736	1185.1375	98.8760
4.171	0.0471	44.9209	14.9492	1.1240
	Sum	3996.3945		

Methyl ((4-acrylamidophenyl)sulfonyl)-*D*-prolinate (5)

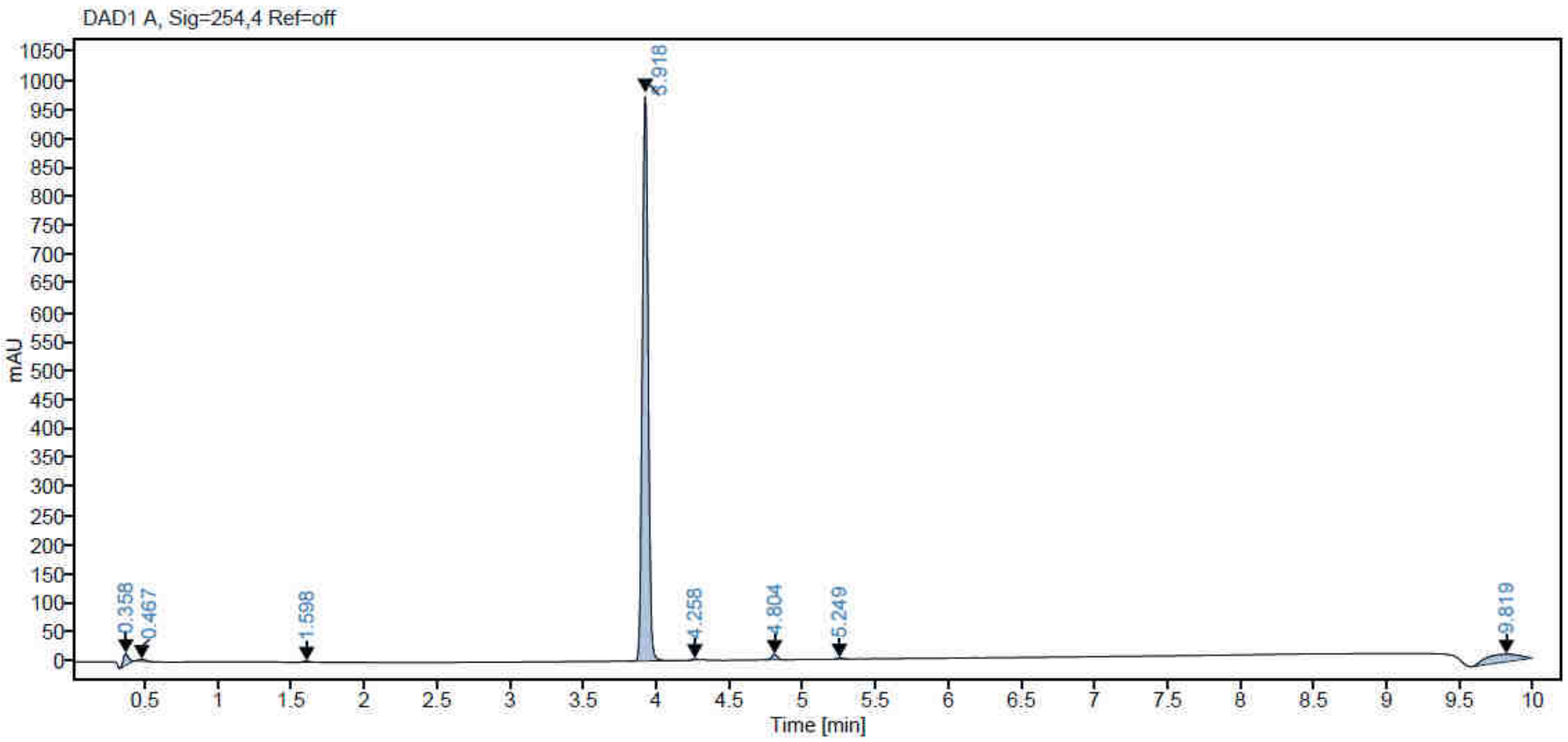

RT [min]	Width [min]	Area	Height	Area%
1.598	0.0946	15.5108	2.3634	0.557
3.918	0.0446	2731.1531	978.4450	98.121
4.258	0.0375	5.4588	2.3184	0.196
4.804	0.0447	25.6539	9.1610	0.922
5.249	0.0467	5.6877	1.9134	0.204
**Sum**		2783.4643		

(*S*)-1-((4-Acrylamidophenyl)sulfonyl)-*N*-(prop-2-yn-1-yl)pyrrolidine-2-carboxamide (6)

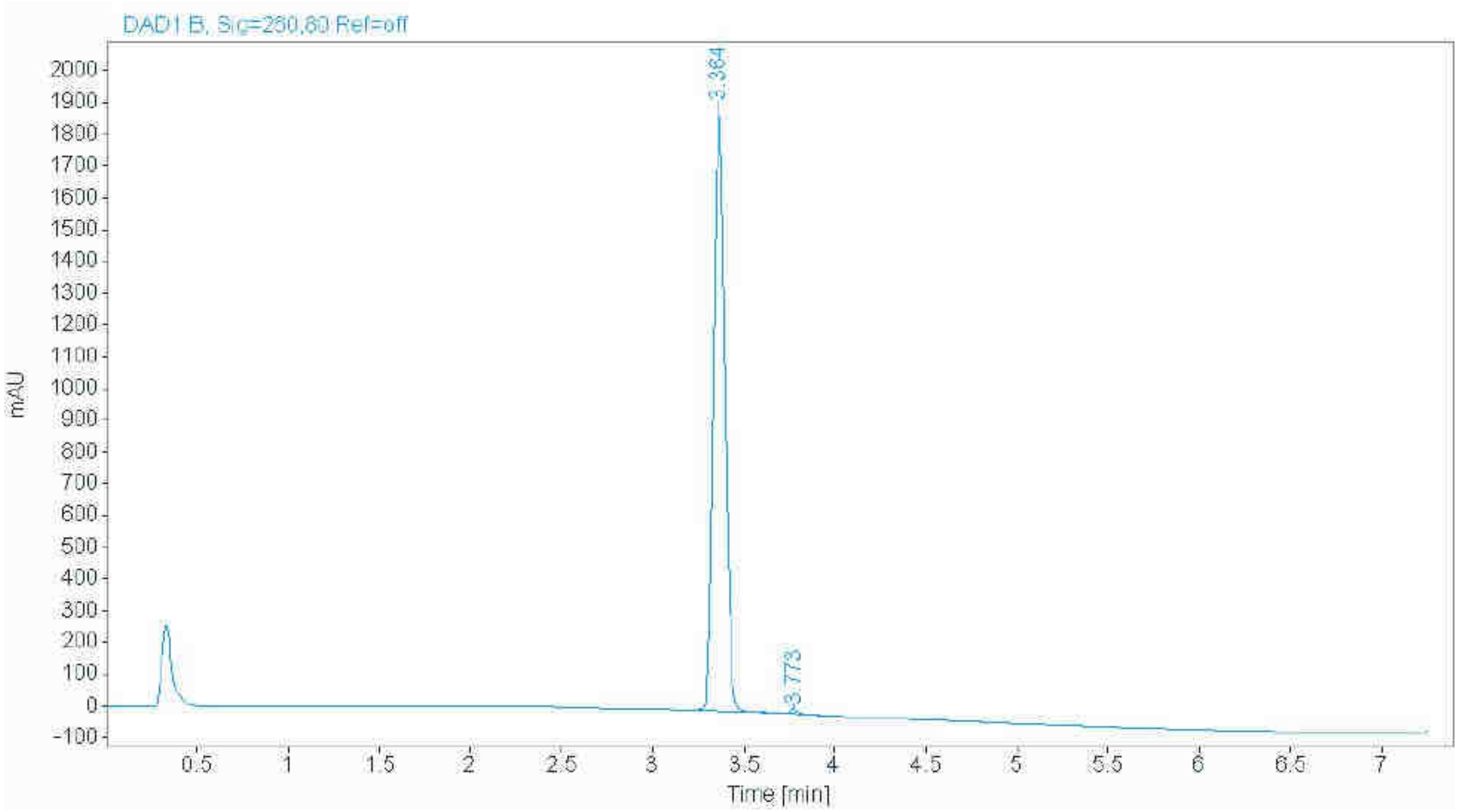

RT [min]	Width [min]	Area	Height	Area%
3.364	0.0691	8398.2217	1918.0364	99.3993
3.773	0.0527	50.7556	14.5393	0.6007
	Sum	8448.9773		

(*S*)-1-((4-Acrylamidophenyl)sulfonyl)-*N*-(4-methoxyphenyl)pyrrolidine-2-carboxamide (7)

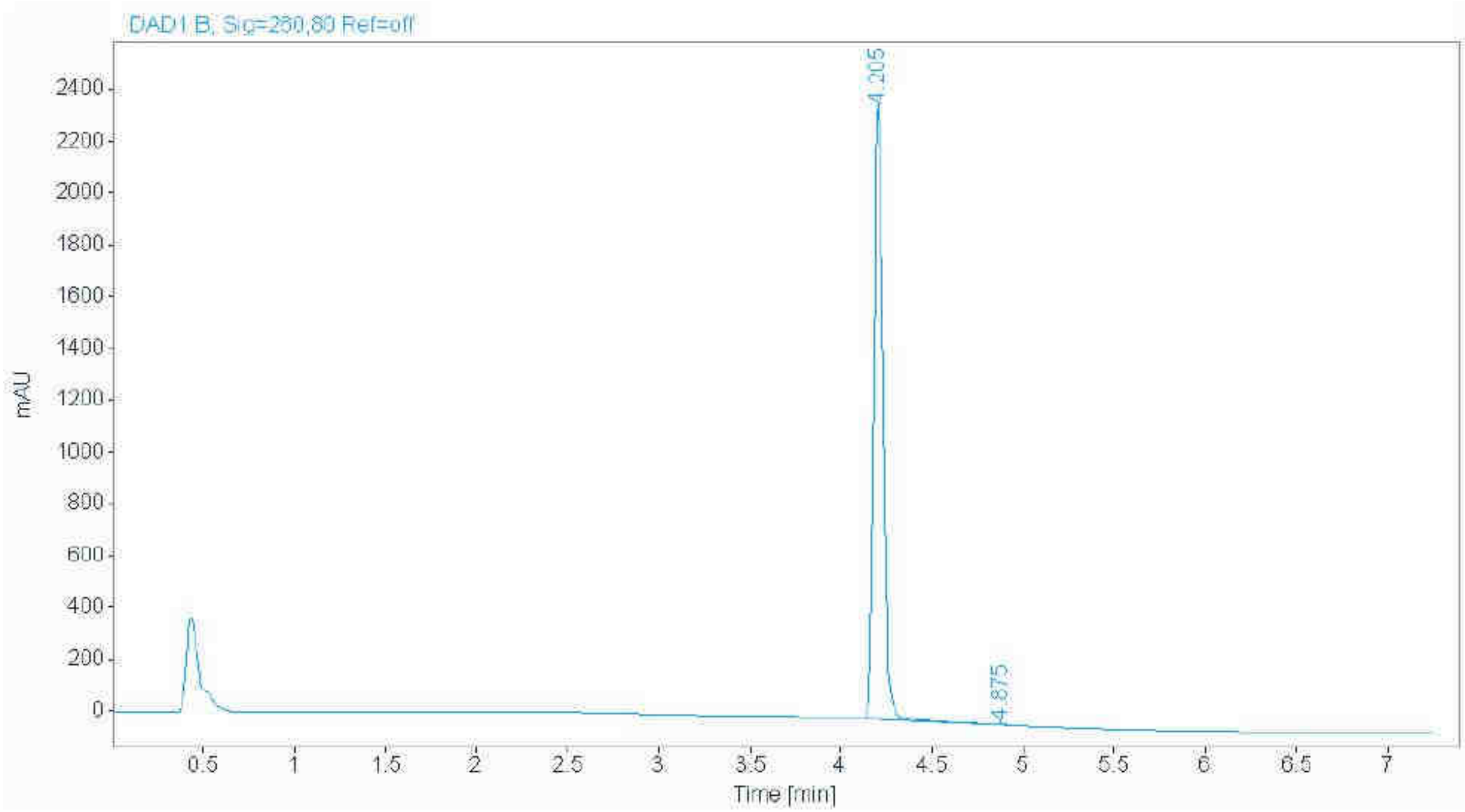

RT [min]	Width [min]	Area	Height	Area%
4.205	0.0583	8623.8184	2378.1069	99.9026
4.875	0.0460	8.4121	2.8891	0.0974
	Sum	8632.2304		

(*R*)-1-((4-Acrylamidophenyl)sulfonyl)-*N*-(4-methoxyphenyl)pyrrolidine-2-carboxamide (8)

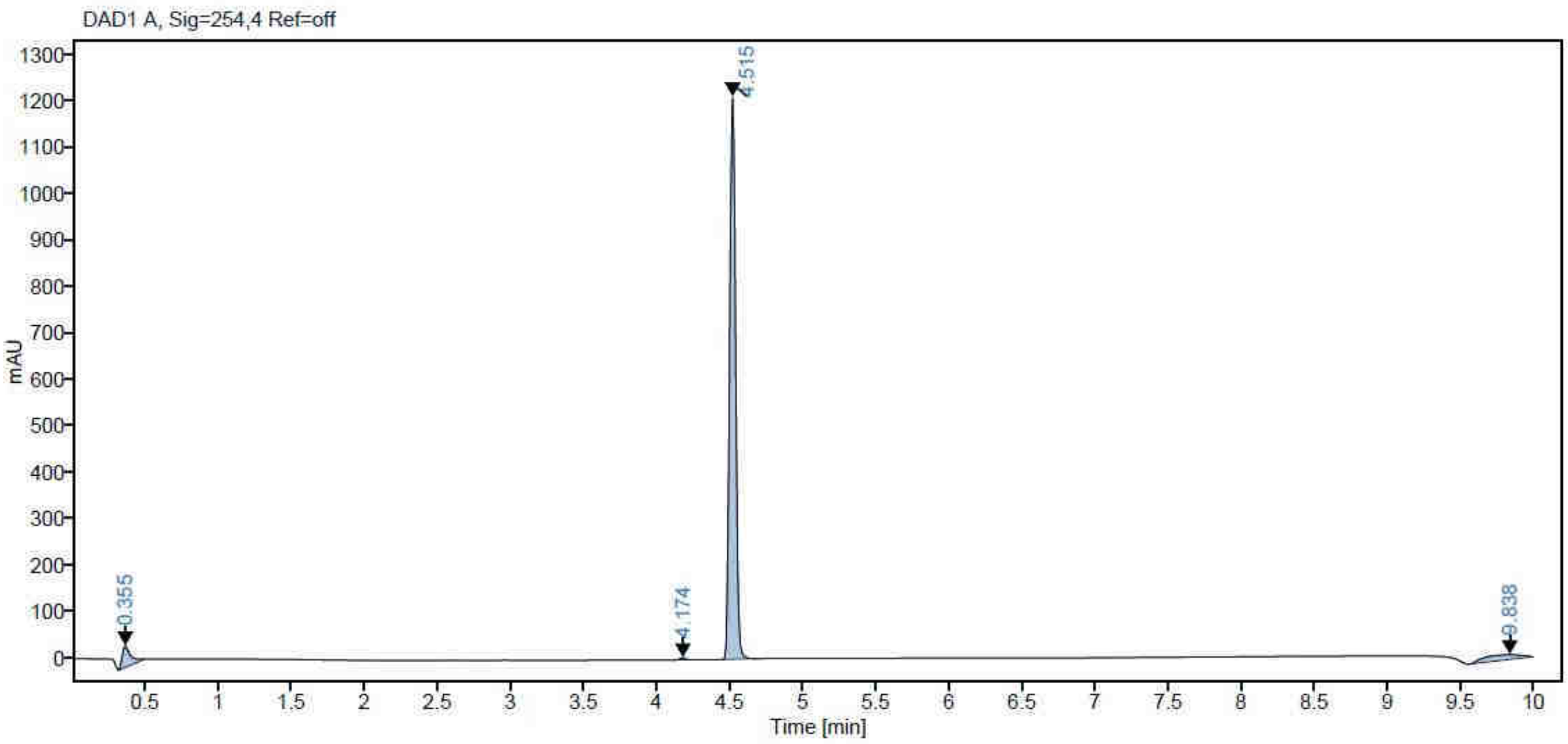

RT [min]	Width [min]	Area	Height	Area%
4.174	0.0439	12.3452	4.5113	0.358
4.515	0.0451	3433.5769	1210.5248	99.642
**Sum**		3445.922		

(*S*)-1-((4-Acrylamidophenyl)sulfonyl)-*N*-(4-(prop-2-yn-1-yloxy)phenyl)pyrrolidine-2-carboxamide (9)

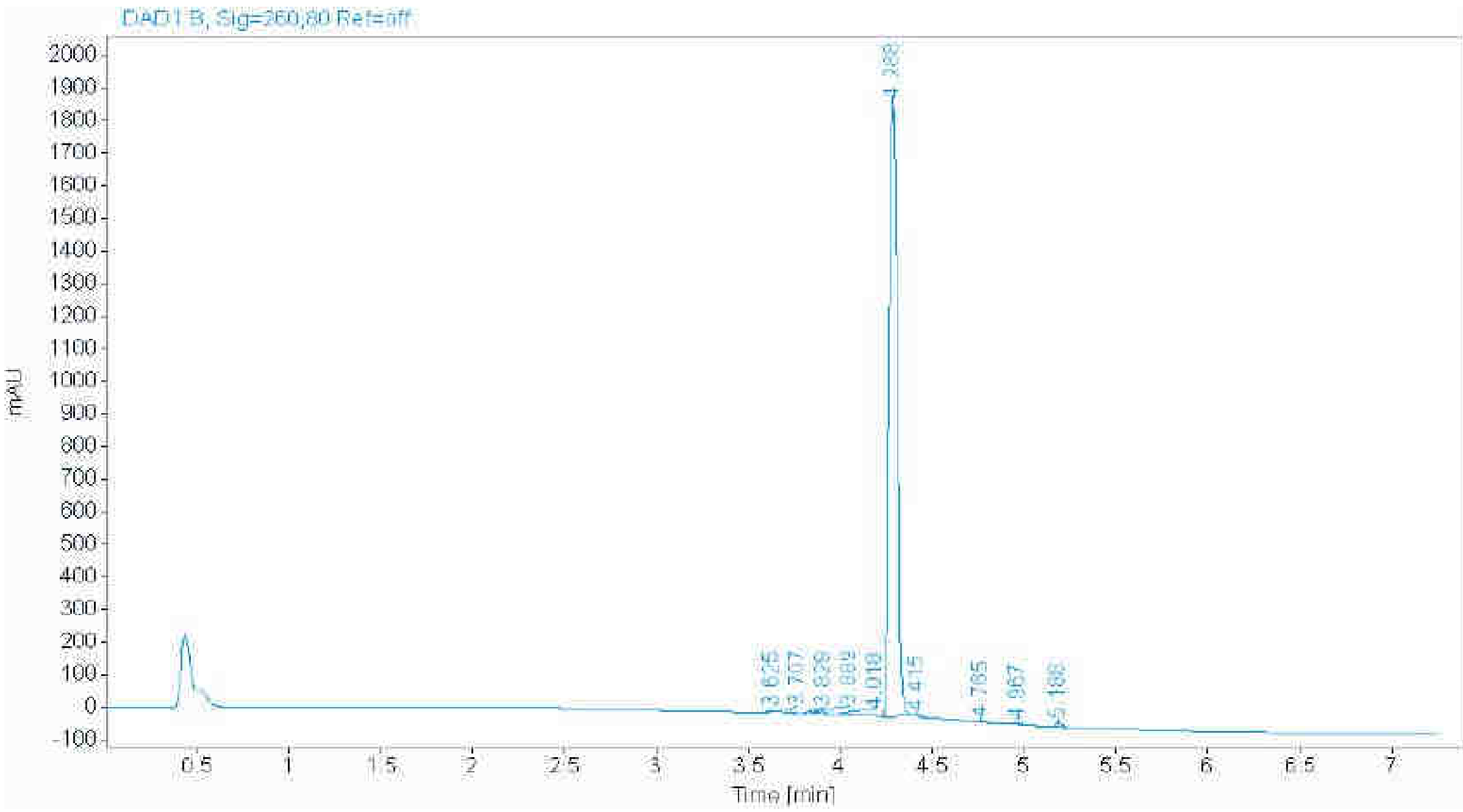

RT [min]	Width [min]	Area	Height	Area%
3.625	0.0428	7.8136	2.9598	0.1355
3.707	0.0489	28.6767	9.5870	0.4975
3.829	0.0350	7.1071	3.3191	0.1233
3.889	0.0414	18.1426	7.1913	0.3147
4.018	0.0471	21.1688	7.0282	0.3672
4.288	0.0487	5632.6465	1892.5148	97.7093
4.415	0.0444	6.8031	2.4480	0.1180
4.765	0.0421	9.6745	3.7456	0.1678
4.967	0.0520	8.4660	2.5991	0.1469
5.188	0.0444	24.1971	8.7102	0.4197
	Sum	5764.6960		

(*S*)-1-((4-Acrylamidophenyl)sulfonyl)-*N*-(4-((1-methyl-1H-pyrazol-3-yl)carbamoyl)phenyl)pyrrolidine-2-carboxamide (10, IMP-2552)

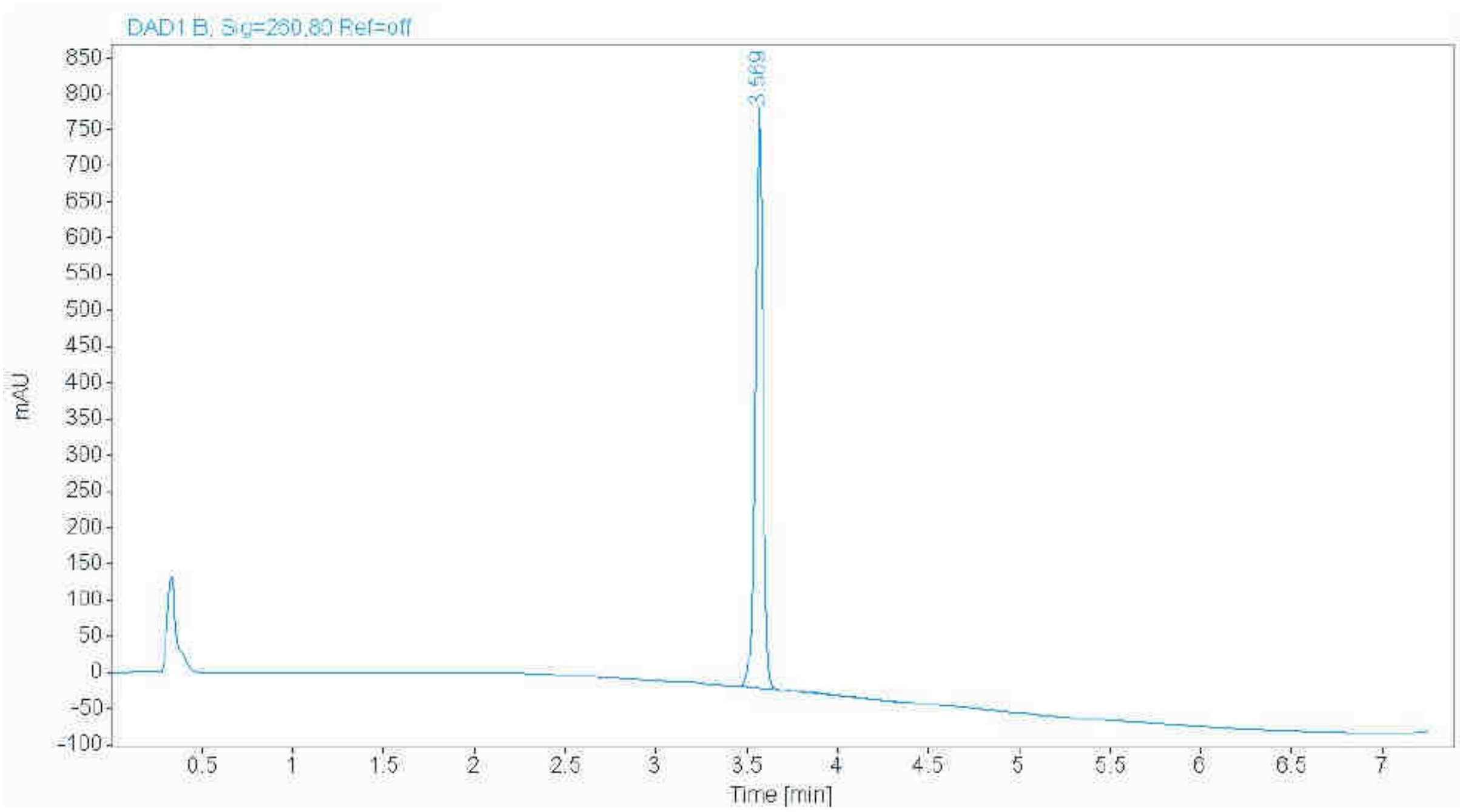

RT [min]	Width [min]	Area	Height	Area%
3.569	0.0465	2379.6851	804.5959	100.0000
	Sum	2379.6851		

(*R*)-1-((4-Acrylamidophenyl)sulfonyl)-*N*-(4-((1-methyl-1H-pyrazol-3-yl)carbamoyl)phenyl)pyrrolidine-2-carboxamide (11)

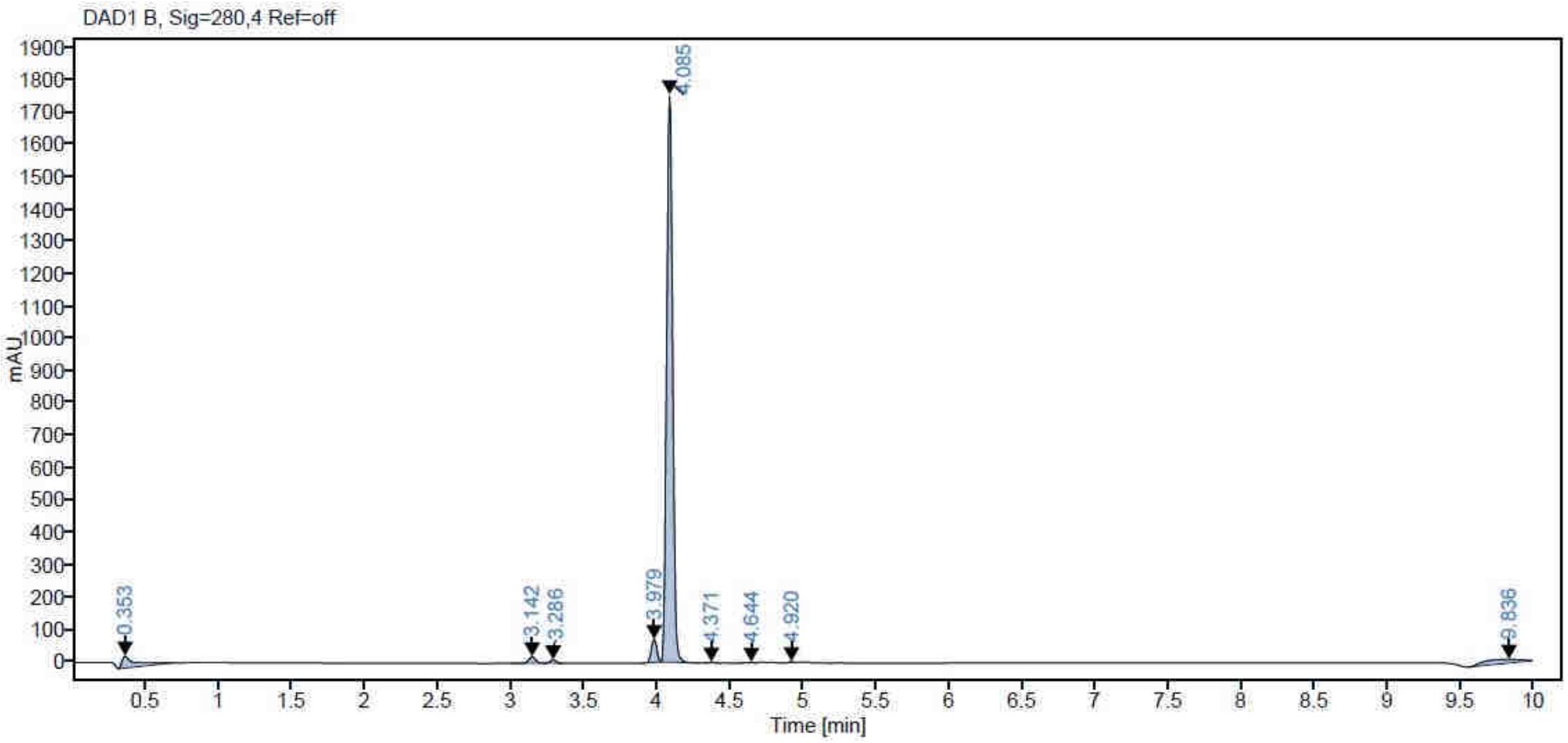

RT [min]	Width [min]	Area	Height	Area%
3.142	0.0529	64.7917	19.4264	1.217
3.286	0.0453	28.8753	10.1085	0.542
3.979	0.0426	185.5625	70.7643	3.484
4.085	0.0454	5026.905	1753.59	94.383
4.371	0.0421	6.7368	2.6095	0.126
4.644	0.0583	6.5666	1.6549	0.123
4.92	0.0423	6.6206	2.5492	0.124
**Sum**		5326.0583		

(*S*)-1-((4-Acrylamidophenyl)sulfonyl)-*N*-(4-((1-(prop-2-yn-1-yl)-1H-pyrazol-4-yl)carbamoyl)phenyl)pyrrolidine-2-carboxamide (12, IMP-2566)

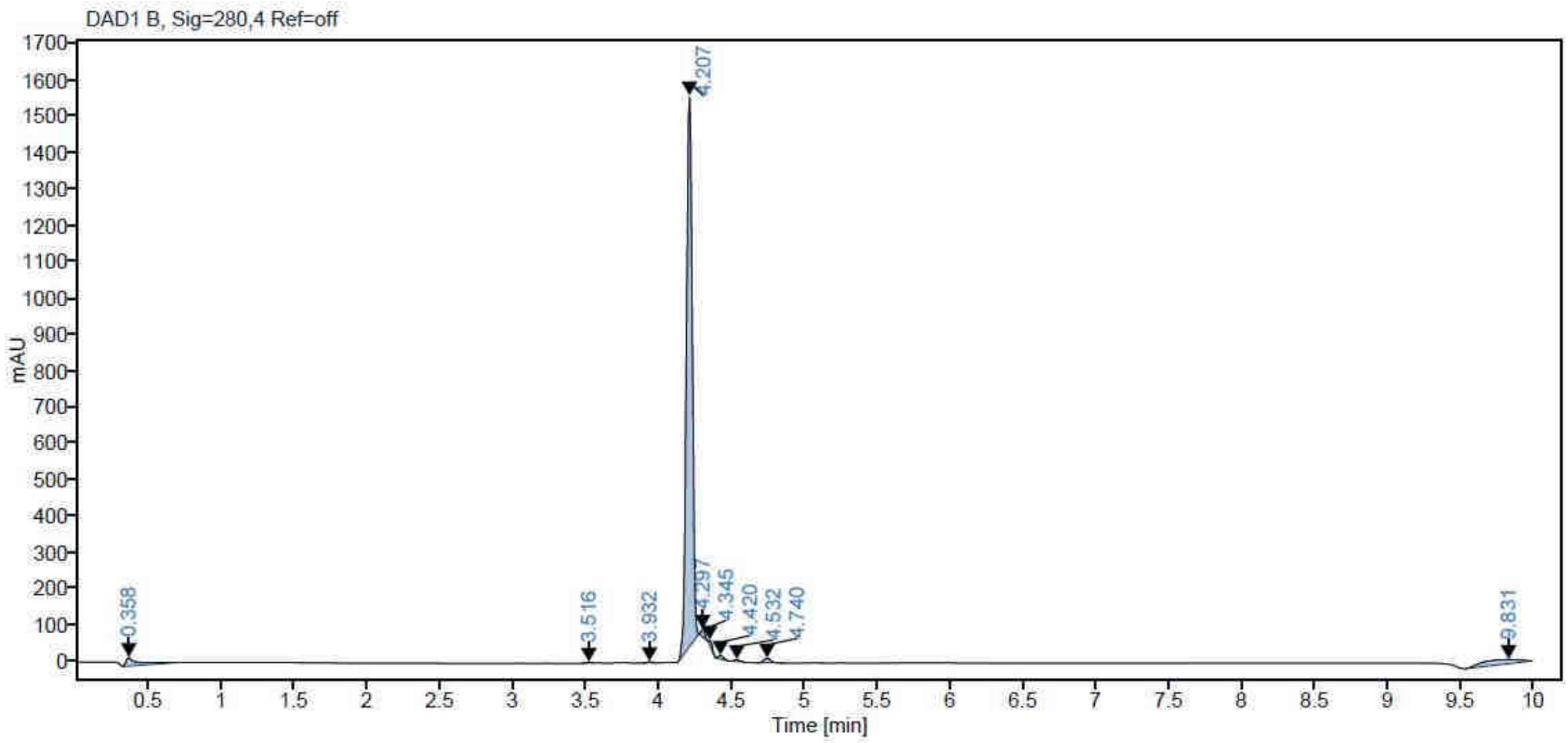

RT [min]	Width [min]	Area	Height	Area%
3.516	0.0401	6.1244	2.5453	0.140
3.932	0.0421	9.0759	3.5205	0.208
4.207	0.0444	4206.396	1514.299	96.242
4.297	0.0293	37.6807	20.6459	0.862
4.345	0.0351	28.3177	13.4499	0.648
4.42	0.0375	24.54	10.4237	0.561
4.532	0.0447	14.7185	5.2542	0.337
4.74	0.0529	43.7828	13.137	1.002
**Sum**		4370.636		

(*S*)-1-((4-Acrylamidophenyl)sulfonyl)-*N*-(3-methoxyphenyl)pyrrolidine-2-carboxamide (13, IMP-2505)

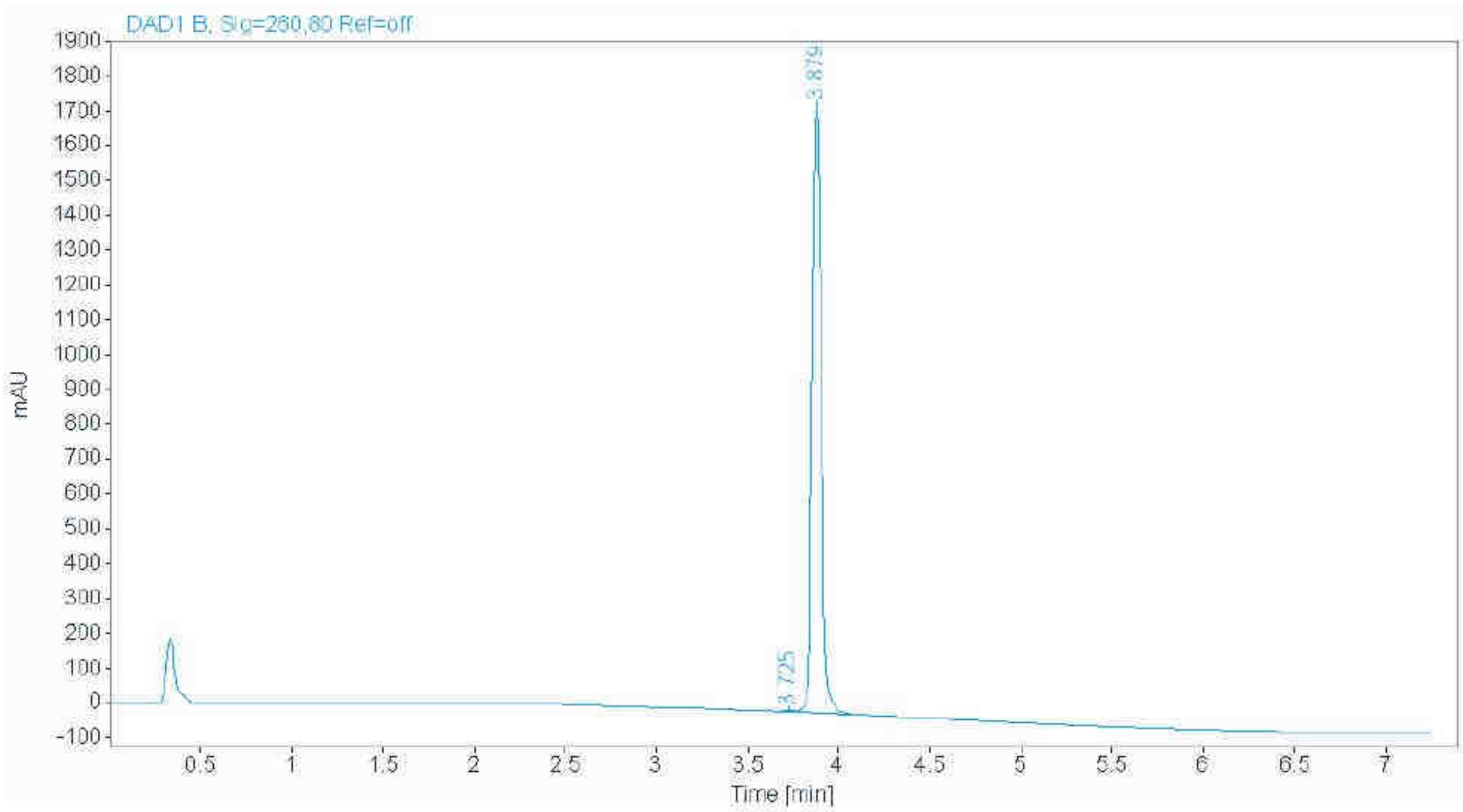

RT [min]	Width [min]	Area	Height	Area%
3.725	0.0420	9.9573	3.8667	0.1740
3.879	0.0521	5711.0508	1746.7648	99.8260
	Sum	5721.0081		

Chiral HPLC Traces

Chiral HPLC was performed on separate enantiomers of **10** and **11**, and a 1:1 racemic mix, using a CHIRALPAK IG-U column (5 µm particle size, column size: 3.0 mm x 100 mm) held at 40 ºC.

Time (min)	A – H_2O+0.1% FA (%)	B – MeCN+0.1% FA (%)	Flow (mL/min)
0.00	35	65	0.6
2.00	35	65	0.6
8.00	10	90	0.6
10.00	10	90	0.6
11.00	35	65	0.6
15.00	35	65	0.6

(*S*)-1-((4-Acrylamidophenyl)sulfonyl)-*N*-(4-((1-methyl-1H-pyrazol-3-yl)carbamoyl)phenyl)pyrrolidine-2-carboxamide (10, IMP-2552)

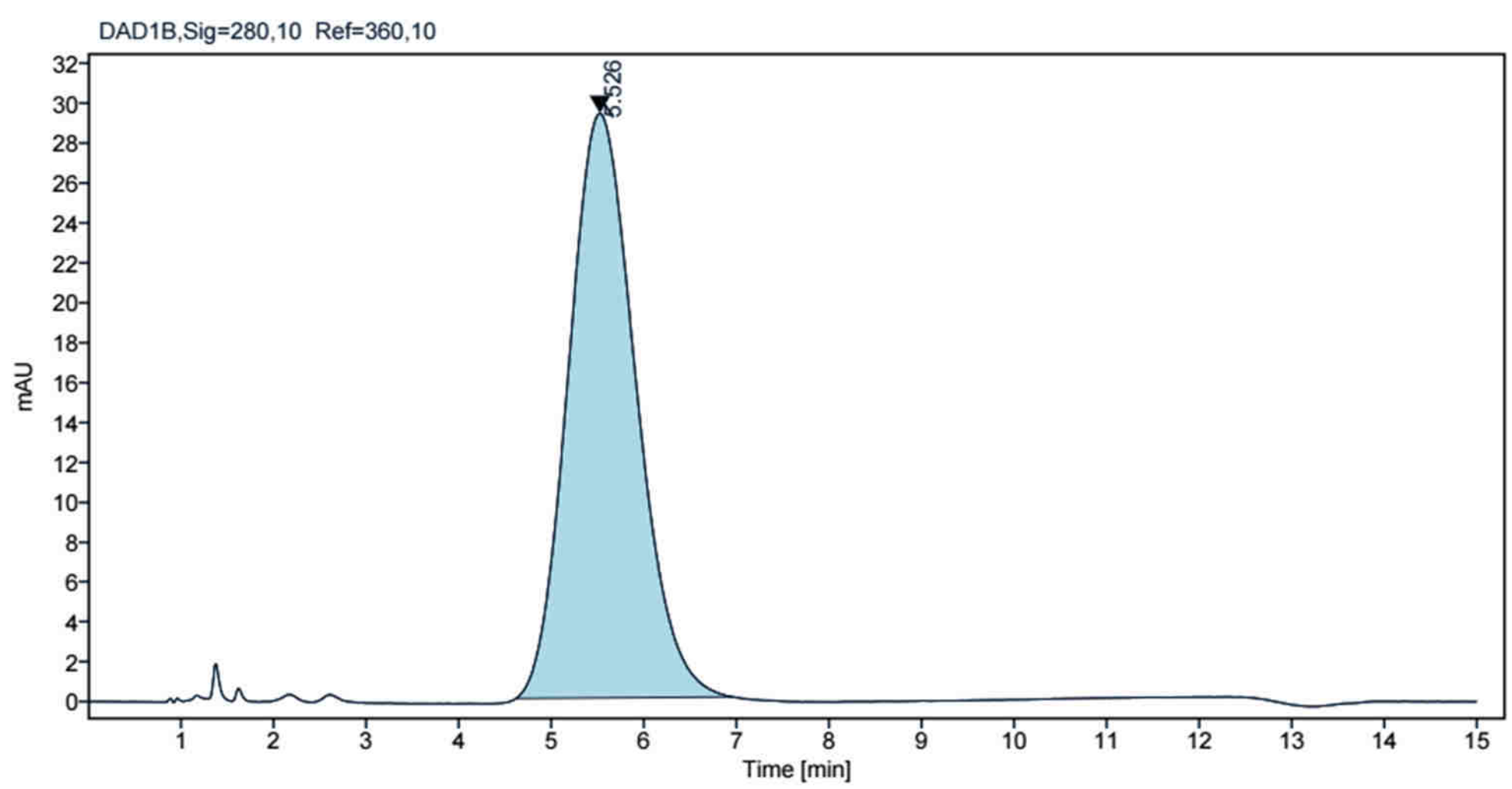

Signal: DAD1B,Sig=280,10 Ref=360,10

RT [min]	Width [min]	Area	Height	Area%
5.526	2.3460	1481.9656	29.2985	100.0000
	**Sum**	**1481.9656**		

(*R*)-1-((4-Acrylamidophenyl)sulfonyl)-*N*-(4-((1-methyl-1H-pyrazol-3-yl)carbamoyl)phenyl)pyrrolidine-2-carboxamide (11)

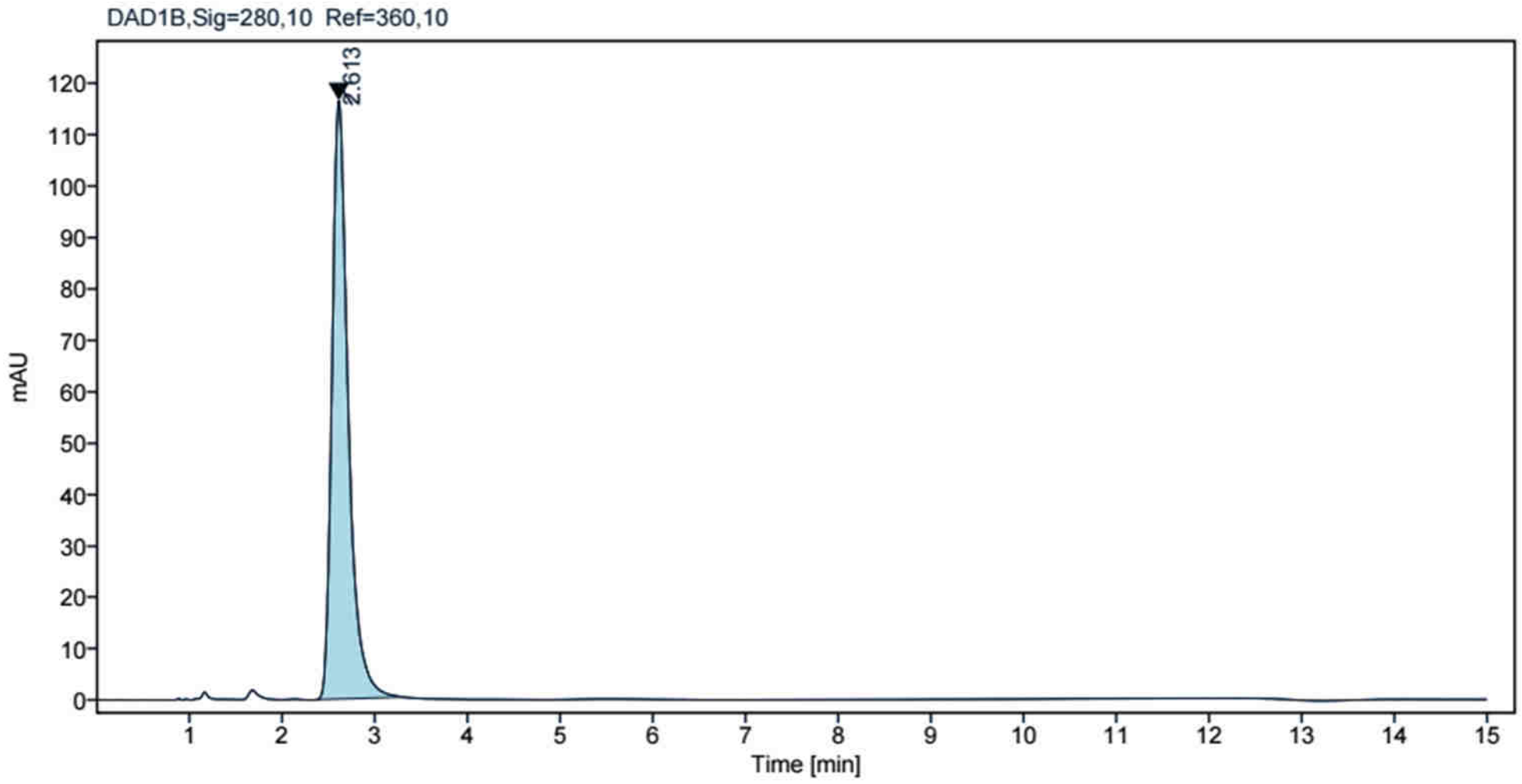

Signal: DAD1B,Sig=280,10 Ref=360,10

RT [min]	Width [min]	Area	Height	Area%
2.613	0.9354	1427.0154	116.3800	100.0000
	Sum	**1427.0154**		

1:1 Mix of Compound 10 and Compound 11

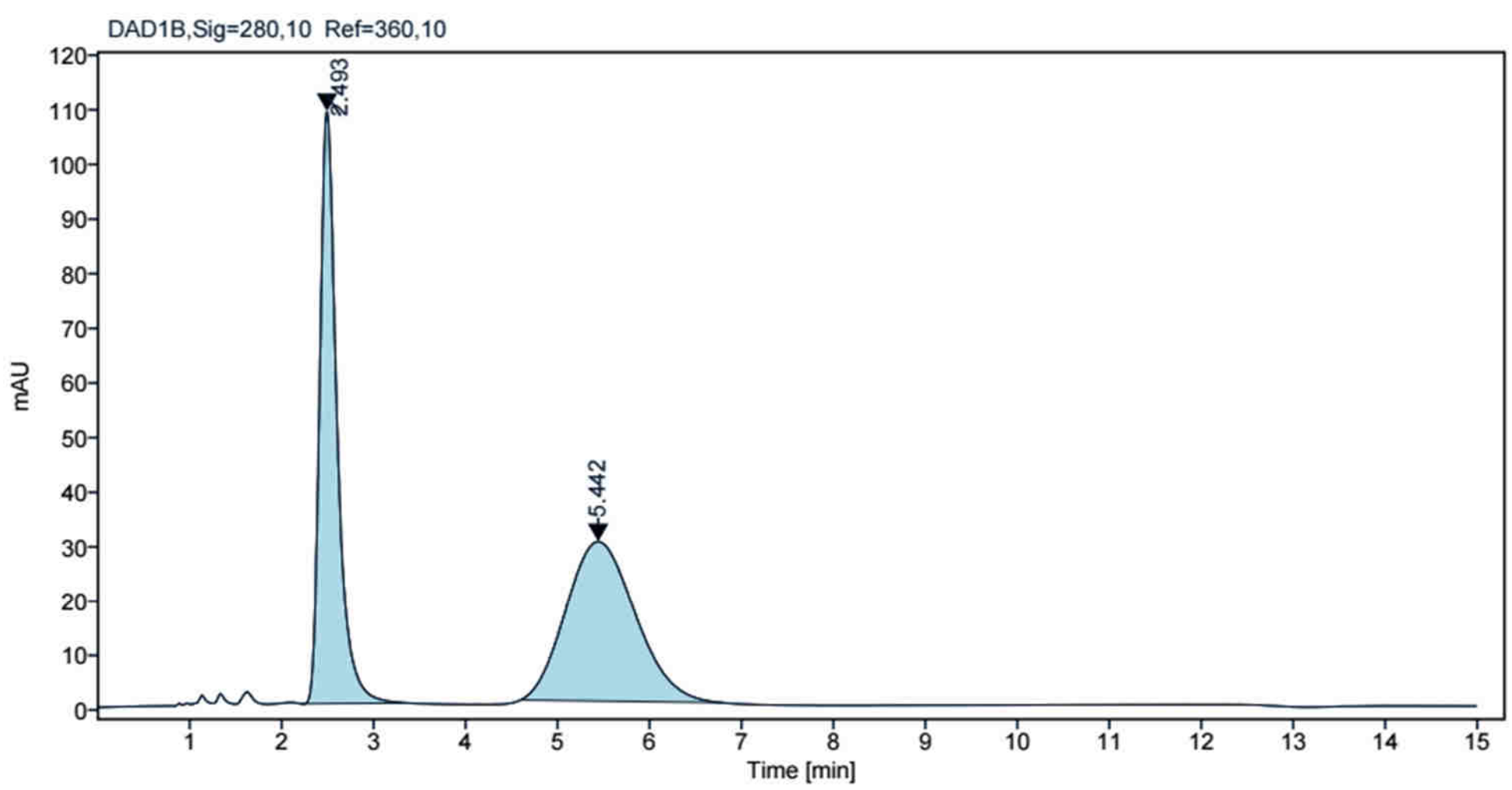

Signal: DAD1B,Sig=280,10 Ref=360,10

RT [min]	Width [min]	Area	Height	Area%
2.493	1.1378	1421.3294	108.5390	48.0071
5.442	2.1886	1539.3338	29.3331	51.9929
	Sum	**2960.6633**		

References

(1) Jamshidiha, M.; Lanyon-Hogg, T.; Sutherell, C. L.; Craven, G. B.; Tersa, M.; De Vita, E.; Brustur, D.; Pérez-Dorado, I.; Hassan, S.; Petracca, R.; Morgan, R. M.; Sanz-Hernández, M.; Norman, J. C.; Armstrong, A.; Mann, D. J.; Cota, E.; Tate, E. W. Identification of the First Structurally Validated Covalent Ligands of the Small GTPase RAB27A. *RSC Med. Chem.* **2022**, *13* (2), 150–155. https://doi.org/10.1039/d1md00225b.

(2) Tropea, J. E.; Cherry, S.; Waugh, D. S. Expression and Purification of Soluble His6-Tagged TEV Protease. *Methods in Molecular Biology* **2009**, *498*, 297–307. https://doi.org/10.1007/978-1-59745-196-3_19.

(3) Craven, G. B.; Affron, D. P.; Allen, C. E.; Matthies, S.; Greener, J. G.; Morgan, R. M. L.; Tate, E. W.; Armstrong, A.; Mann, D. J. High-Throughput Kinetic Analysis for Target-Directed Covalent Ligand Discovery. *Angew. Chem. Int. Ed.* **2018**, *57* (19), 5257–5261. https://doi.org/10.1002/anie.201711825.
